# Asymmetric systematic synthesis, structures, and (chir)optical properties of a series of dihetero[8]helicenes†‡

**DOI:** 10.1039/d1sc00044f

**Published:** 2021-01-29

**Authors:** Tomoyuki Yanagi, Takayuki Tanaka, Hideki Yorimitsu

**Affiliations:** Department of Chemistry, Graduate School of Science, Kyoto University Japan yori@kuchem.kyoto-u.ac.jp

## Abstract

A series of dihetero[8]helicenes have been systematically synthesized in enantiomerically enriched forms by utilizing the characteristic transformations of the organosulfur functionality. The synthetic route begins with assembling a ternaphthyl common synthetic intermediate from 2-naphthol and bissulfinylnaphthalene through an extended Pummerer reaction followed by facile multi-gram-scale resolution. The subsequent cyclization reactions into dioxa- and dithia[8]helicenes take place with excellent axial-to-helical chirality conversion. Dithia[8]helicene is further transformed into the nitrogen and the carbon analogs by replacing the two endocyclic sulfur atoms *via* S_N_Ar-based skeletal reconstruction. The efficient systematic synthesis has enabled comprehensive evaluation of physical properties, which has clarified the effect of the endocyclic atoms on their structures and (chir)optical properties as well as the unexpected conformational stability of the common helical framework.

## Abstract 2

A series of dihetero[8]helicenes have been synthesized in a stereoselective manner through an organosulfur-based synthetic strategy, which has enabled clarifying the effect of the endocyclic atoms on physical properties.

## Graphical abstract

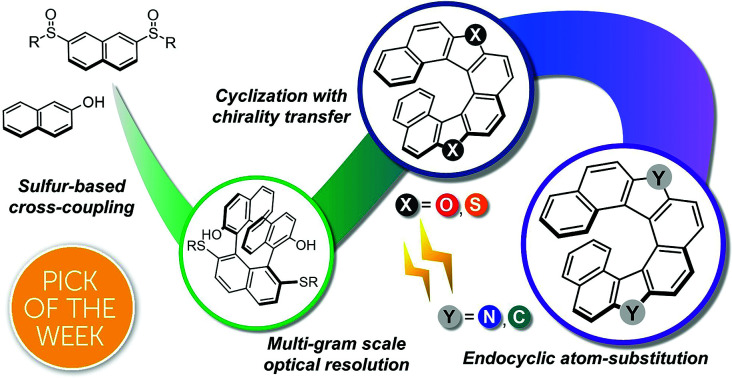

## Introduction

Helicenes are screw-shaped compounds composed of *ortho*-fused benzene rings. Their intriguing properties originating from their stable helical chirality and extended π-systems have received considerable attention and have been applied in a variety of fields^1^ including asymmetric catalysis,^2*a*,*b*^ molecular machines,^2*c*^ polymers,^2*d*,*e*^ molecular recognition,^2*f*^ organic electronics,^2*g*^ and chiroptical devices.^2*h*,*i*^ Hence, many efforts have been made to perform structural modifications of helicenes aiming at changing and improving their physicochemical properties. Among them, introducing a heterocycle in a benzene-based polycyclic helical skeleton is a highly promising approach^3^ because it perturbs the original structure and electronic state to offer new properties and functions.^4^

As challenging but attractive targets, a variety of methodologies have been developed for the synthesis of helicenes including photocyclization, Diels–Alder reactions, transition-metal-catalyzed [2 + 2 + 2] cycloaddition, C–H arylation, and ring-closing metathesis.^1*a*,*e*,5^ Advantageously, the synthesis of heterohelicenes that contain a heterocycle or heteroatom can depend additionally on the characteristic reactivity of the heteroatom, which offers further diversification of synthetic approaches.^3,4,6^ However, the characteristic reactivity of each heteroatom adversely renders systematic synthesis of heterohelicenes that bear different endocyclic heteroatoms more complicated and thus burdensome. In fact, a reported large series of conformationally stable helicenes with different endocyclic atoms are limited to hetero[7]helicenes (diphenanthro[3,4-*b*:4′,3′-*d*′]heteroles). The sulfur-containing hetero[7]helicene (diphenanthro[3,4-*b*:4′,3′-*d*′]thiophene) was synthesized in 1997 by De Lucchi and Smith.^7*a*^ Following this pioneering work, Nozaki and Nakano systematically synthesized its oxygen, nitrogen, phosphorus, silicon, and carbon analogs in sequence during 2005–2016 by elegantly using transition metal-catalyzed bond-forming reactions.^7*b*–*e*^ This steady yet slow progress in the synthesis of this single series of hetero[7]helicenes apparently indicates the immaturity of synthetic protocols and strategies that offer facile accesses to structurally constrained heterohelicenes.

Considering the importance of the elucidation of structure–property relationship for molecular designs to develop useful heterohelicenes, there remains ample room to develop new synthetic approaches to heterohelicenes with higher efficiency and structural diversity including kinds and positions of endocyclic heteroatoms and numbers of constituent rings.^8^

The chirality of helicenes is another key issue. It is important to obtain one enantiomer of a (hetero)helicene in an adequate amount in order to develop practically useful helically chiral functional materials. To this end, numerous efforts have been devoted to stereoselective synthesis of helicenes using chiral auxiliaries and resolving agents.^5*b*^ For the past two decades, transition metal-catalyzed^9^ and organocatalyzed^10^ enantioselective syntheses have also emerged as game-changing tools. Despite these remarkable advances, new methodologies that provide facile systematic access to a variety of enantio-enriched heterohelicenes have been still highly sought after.

Recently, we have been interested in development of new transformations that exploit the characteristic features of organosulfur compounds. Along this line, we reported the metal-free C–H/C–H coupling reaction of aryl sulfoxides with phenols by utilizing the sulfoxide moiety as both an internal oxidant and a directing group.^11,12^ This method provided highly efficient accesses to sterically congested useful 2,2′-disubstituted biaryls. Of particular note is the preparation of a ternaphthalenediol having two dodecylsulfanyl groups, from which dioxa[8]helicene was synthesized as a racemate.

**Scheme 1:**
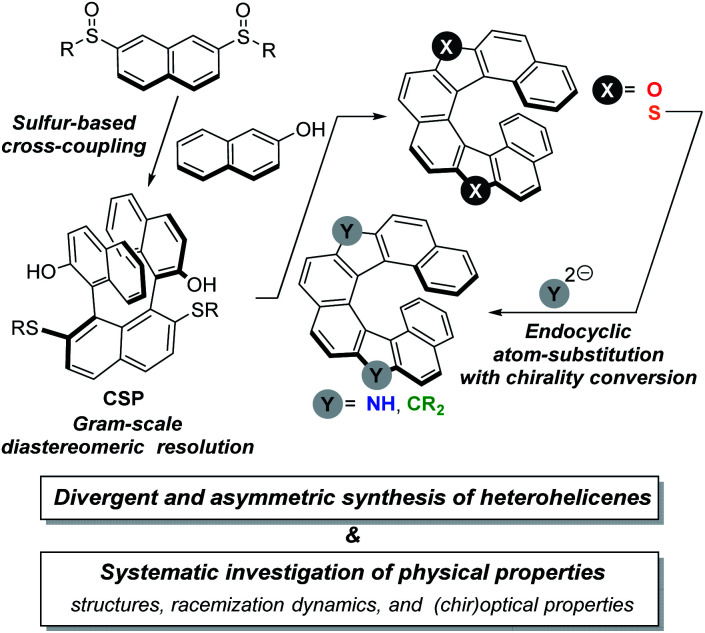
Overview of our synthetic route to chiral dihetero[8]helicenes of structural and photophysical interest.

The concise synthesis of racemic dioxa[8]helicene allowed us to envision that the di(alkylsulfanyl)ternaphthalenediol skeleton would be an excellent platform for the highly efficient and systematic synthesis of enantioenriched dihetero[8]helicenes. Scheme 1 illustrates an overview of our idea. With a proper substituent R on the bissulfinylnaphthalenes, a common synthetic precursor **CSP** is prepared on a multi-gram scale. The common precursor **CSP** is easy to resolve on a preparative scale with a chiral derivatizing agent by using the phenolic hydroxy groups. The asymmetric synthesis of dioxa[8]helicene would be facile according to the previous report. We should choose the R substituents as being readily removable so as to help to synthesize dithia[8]helicene from **CSP**. Once dithia[8]helicene is synthesized, we would be able to take advantage of our synthetic strategy about endocyclic transformations of thiophene compounds, which we have coined ‘aromatic metamorphosis’.^13^ According to our previous reports about the transformations of dibenzothiophene *S*,*S*-dioxides into carbazoles^14^ and fluorenes,^15^ we expected the synthesis of diaza[8]helicene and the carbon analog from the sulfone being feasible, although one concern would lie in the chirality conversion during the aromatic metamorphosis. Here we report that the asymmetric synthesis of a series of dihetero[8]helicenes has been indeed viable. The key to the synthesis is utilization of sulfur functionalities as useful synthetic auxiliaries that exhibit diverse reactivity depending on its oxidation state. The obtained helicenes were subjected to systematic investigation of their physical properties including structural features, racemization dynamics, and (chir)optical properties, which has unraveled the electronic and structural effects of embedded atoms in the common skeleton, unexpected conformational stability, and (chir)optical properties.

## Results and discussion

### Synthesis

**Scheme 2:**
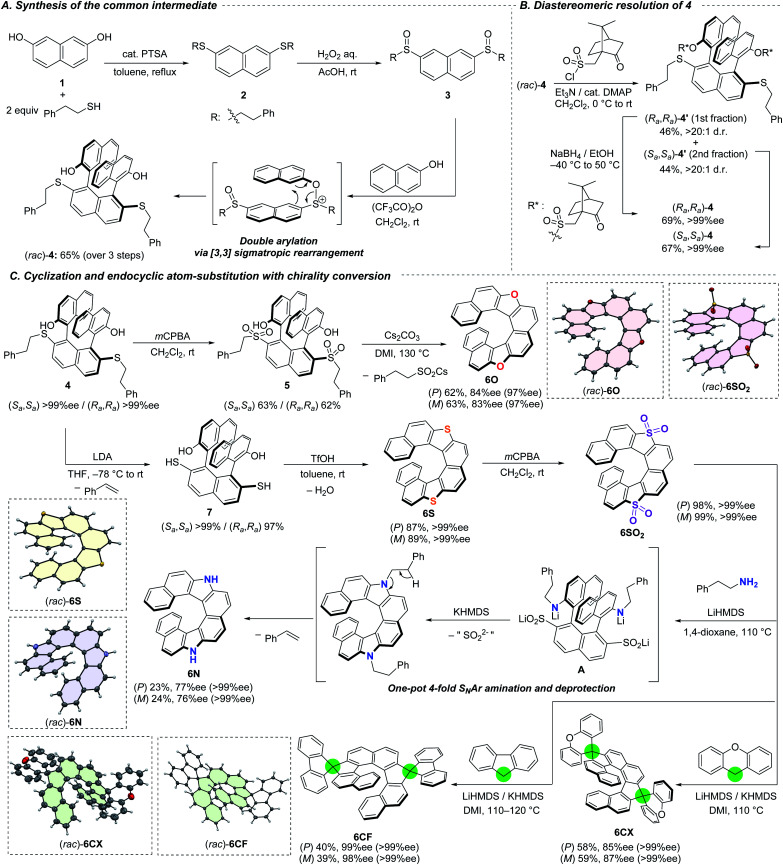
Synthetic routes to a series of dihetero[8]helicenes. (A) Synthesis of common intermediate (*rac*)-**4**. (B) Resolution of **4**. (C) Conversions of enantiopure **4** to dihetero[8]helicenes. Structures in dashed boxes were obtained by X-ray single crystal diffraction analysis, and all thermal ellipsoids are set at a 50% probability level. Solvent molecules in (*rac*)-**6CX** and (*rac*)-**6CF** were omitted for clarity. Values of ee in parenthesis are those after recrystallization.

The series of dihetero[8]helicenes were prepared from commercially available 2,7-naphthalenediol (**1**) and 2-phenylethanethiol (Scheme 2A). The first step is acid-catalyzed condensation between the phenolic hydroxy groups and the thiol, resulting in the formation of bissulfide **2**. The choice of the thiol was planned in order that the 2-phenylethyl moiety serves as a protecting group for a SH group which is fairly stable but undergoes easy deprotection under basic conditions (*vide infra*). Subsequent oxidation with aqueous H_2_O_2_ afforded bissulfoxide **3** in nearly quantitative yield. The key coupling reaction with two 2-naphthols smoothly proceeded with the aid of trifluoroacetic anhydride through transient S–O bond formation and subsequent [3,3] sigmatropic rearrangement.^11*a*^ To our delight, two naphthol moieties were introduced regioselectively to the 1- and 8-positions and stereoselectively to afford the *trans* isomer where the two hydroxy groups are located toward the opposite directions. Notably, our protocol tolerates a large-scale synthesis; 58 mmol (40 g) of (*rac*)-**4** was obtained from 90 mmol of **1** (65% yield) without intermediate purification. In order to construct a ternaphthyl motif, direct oxidative coupling of **1** with 2-naphthol would be a more straightforward alternative.^9*b*,16^ However, it suffers from undesired homo-coupling, oligomerization, and/or overoxidation. Advantageously, our three-step synthesis is highly efficient and selective by utilizing the sulfoxide moiety as a directing group. Moreover, overreactions could be suppressed because the sulfoxide moieties are reduced to sulfides to lose the directing ability.^11*c*^

For the streamlined asymmetric synthesis of a series of dihetero[8]helicenes, we next investigated optical resolution of common synthetic intermediate **4** (Scheme 2B) to avoid troublesome compound-dependent condition screening for optical resolution of every dihetero[8]helicene. Thanks to the 2-naphthol-derived hydroxy groups, racemic ternaphthalene **4** could be converted into a pair of diastereomers by using (+)-10-camphorsulfonyl chloride.^7*b*,17^ The diastereomeric mixture of bissulfonyl ester **4′** was successfully separated by silica-gel column chromatography on a gram-scale (*R*_f_ = 0.21 and 0.31 with toluene/*n*-hexane/EtOAc = 10/10/3 as an eluent). The sulfonyl group in each diastereomer can be removed with the aid of sodium borohydride^18^ without any loss of the axial chirality. Both of the diastereomers were obtained in isomerically pure forms in good yields. The absolute configuration of **4** derived from the latter eluting fraction of **4′** was determined to be *S*_*a*_,*S*_*a*_ (*vide infra*).

For the access to dioxa[8]helicene **6O**, namely dinaphtho[1,2-*d*:1′,2′-*d*′]naphtho[2,1-*b*:7,8-*b*′]furan, we employed intramolecular cyclization under basic conditions using the sulfur functionality as a leaving group (Scheme 2C). To improve the leaving group ability, the sulfanyl moieties of (*S*_*a*_,*S*_*a*_)-**4** were oxidized to the sulfones. The bis-sulfone **5** was successfully cyclized into (*P*)-**6O** in 62% yield with a slight decrease of optical purity (84% ee).^11*a*^ The optical purity could be improved by recrystallization from CH_2_Cl_2_/*n*-hexane, where crystals were precipitated predominantly in a racemic form and the ee of **6O** in the filtrate was increased to 97% ee. The other isomer, (*M*)-**6O**, was obtained in the same way from (*R*_*a*_,*R*_*a*_)-**4**.

The sulfanyl moieties of **4** can be used for the construction of thiophene segment to synthesize dithia[8]helicene **6S**. Treatment of (*S*_*a*_,*S*_*a*_)-**4** with LDA invoked E2 elimination to form the corresponding bisthiol **7** efficiently. Subsequent intramolecular condensation of the thiol moieties with the hydroxy groups of **7** was facile to afford (*P*)-**6S** with the aid of triflic acid. The same protocol was applicable to the synthesis of (*M*)-**6S** from (*R*_*a*_,*R*_*a*_)-**4**. Because the elimination and condensation proceeded at ambient temperature, no degradation of the enantiomeric excess was observed.

Each enantiomer of the obtained **6S** was oxidized to the corresponding tetraoxide **6SO2** quantitatively. The tetraoxide **6SO2** were subjected to a series of endocyclic substitution reactions based on our aromatic metamorphosis methodology.

Diaza[8]helicenes (*P*)- and (*M*)-**6N** were synthesized from (*P*)- and (*M*)-**6SO2**, respectively, by treatment with 2-phenylethylamine in the presence of strong bases in a one-pot process.^14*b*,19^ The transformation includes multiple steps and began with ring-opening amination with 2-phenylethylamine mediated by LiHMDS to form intermediate **A** and its two other regioisomers in terms of the positions of the C–S bond cleavage. Subsequent addition of KHMDS to the reaction mixture invoked cyclization with concomitant elimination of the sulfur moieties. Finally, base-mediated E2 elimination across the 2-phenylethyl groups furnished enantiomerically enriched **6N**. The sequential additions of LiHMDS and KHMDS were crucial because **6SO2** readily decomposed in the presence of more basic KHMDS while LiHMDS was not basic enough to mediate the ring-closing amination. Slight decreases of the optical purity of **6N** to 77 and 76% ee were observed during the substitution because of the reaction temperature as high as 110 °C. Recrystallizations from *n*-hexane/dichloromethane successfully afforded crystalline (*P*)- and (*M*)-**6N** in enantiomerically pure forms.

Carbon nucleophiles possessing appropriate acidity such as xanthene and fluorene could also be applied for the endocyclic substitution reaction.^15^ The LiHMDS/KHMDS-mediated reaction of (*P*)-**6SO2** with xanthene at 110 °C afforded double spiro-shaped helicenes (*P*)-**6CX** in good yield with slight decrease of the optical purity to 85% ee.^7*e*,9*a*^ Subsequent recrystallization from *n*-hexane/dichloromethane provided the enantiomerically pure (*P*)-**6CX**. By using fluorene as a different nucleophile, almost optically pure (*P*)-**6CF** was obtained despite a lower yield (40%, 99% ee) in comparison with **6CX**. The lower yield is probably attributed to the low nucleophilicity of aromatic fluorenyl anion.

The structures of all racemic helicenes were unambiguously revealed by X-ray diffraction analysis (XRD) of single crystals. The absolute configuration of (*P*)-**6S** was determined by XRD (Fig. S14‡), and those of all the other chiral compounds were estimated from the structure of (*P*)-**6S** and CD spectra (*vide infra*).

### Structure and racemization dynamics

**Fig. 1:**
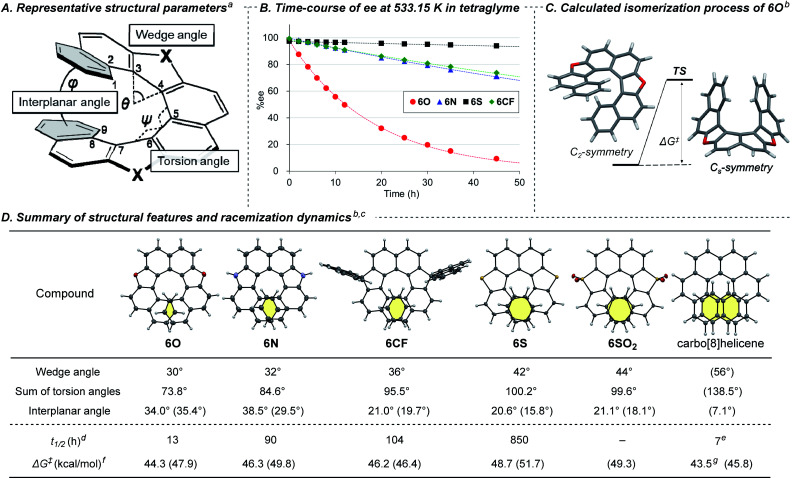
(A) Representative structural parameters of dihetero[8]helicenes. (B) Time-course of ee of dihetero[8]helicenes at 533.15 K. (C) Calculated isomerization process of **6O**. (D) Summary of structural features and racemization dynamics. ^*a*^See Fig. S21‡ for the detailed definition for the wedge angle. ^*b*^DFT calculation was conducted at the B3LYP-D3(BJ)/6-311G(d,p) level of theory in the gas phase. ^*c*^The structural parameters were obtained by X-ray single crystal diffraction analysis of the racemates. Estimated values based on DFT calculations are shown in parentheses. The structure of carbo[8]helicene shown in the first row was obtained by DFT calculation. ^*d*^Experimental half-life time of ee at 533.15 K in tetraglyme. ^*e*^Experimental value at 531.76 K taken from ref. 22. ^*f*^Activation free energies for racemization at 533.15 K. ^*g*^Estimated by experimentally obtained values of Δ*H*^‡^ and Δ*S*^‡^ taken from ref. 22.

It has been widely accepted that replacement of a benzene ring with a five-membered ring in a chiral helicene makes its racemization more facile because the smaller five-membered ring reduces the overlapping area of the two terminal benzene rings.^3*a*^ However, there has been few quantitative and systematic investigations of the effect of endocyclic atoms because of synthetic difficulty.^7,8^ With the series of dihetero[8]helicenes in our hands, we conducted systematic investigations using **6O**, **6N**, **6S**, **6SO2**, **6CF**, and carbo[8]helicene as a reference compound to reveal the effects of the endocyclic atoms on the structure and racemization dynamics. For the evaluation of the structural features of these helical molecules, we selected three parameters (Fig. 1A):^1*a*,3*a*^ (1) the wedge angle defined as the angle between the two formal C=C bonds of the five-membered ring, reflecting the ring size of heterocycles;^20^ (2) the sum of the torsion angles of the inner rims (from ∠C1–C2–C3–C4 to ∠C6–C7–C8–C9), which represents the degree of the twist of a helix;^7*c*^ (3) the interplanar angle between the mean planes of the terminal benzene rings related to the degree of compression of helical molecules. The helicenes were horizontally listed in Fig. 1D in the order of increasing the wedge angles (**6O** < **6N** < **6CF** < **6S** < **6SO2** ≪ carbo[8]helicene). There is a positive correlation between the wedge angles, the sums of the torsional angles, and the overlapping areas of the terminal rings highlighted in yellow. Although the interplanar angles based on the crystal structure do not show any noticeable trends, energy-minimized structures obtained by DFT calculations at the B3LYP-D3(BJ)/6-311G(d,p) level clearly show a negative correlation between the calculated interplanar angles (shown in parentheses in Fig. 1D) and the wedge angles. The irregularity observed in the solid state would be attributable to crystal packing forces distorting the molecular springs.^21^

The pattern of crystal packing depends on the embedded elements (Fig. S8–S20‡): (*rac*)-**6O** and (*rac*)-**6S** are filled in a herringbone pattern, whereas (*rac*)-**6N** is arranged to be orthogonal to each other due to participation of NH–π interaction. (*rac*)-**6SO2** showed a one-dimensional columnar arrangement in which *P* and *M* isomers are stacked alternatively. Of note is that spontaneous resolution took place in the case of crystallization from (*rac*)-**6CX** and (*rac*)-**6CF**.

Next, we measured the rate constant of racemization of a series of helicenes at several temperatures and determined the activation free energies Δ*G*^‡^ for the inversion of helicity by Eyring plots (Fig. 1B, D and Table S5‡). The rate constants for racemization were found to be quite sensitive to the embedded atoms in the five-membered rings. The half-life time (*t*_1/2_) of the enantiomeric excess of **6O** at 260 °C (533.15 K) was calculated to be 13 h, and those of **6N** and **6S** were 7-times (90 h) and 65-times (850 h) longer, respectively, which is consistent with the trend of the overlapping area of the terminal benzene rings. Unfortunately, the rate constant for **6SO2** could not be determined because thermal decomposition was more dominant than racemization. Surprisingly, all the dihetero[8]helicenes synthesized in this study were found to be conformationally more stable than carbo[8]helicene, by judging from the experimentally obtained activation free energies.^22^ In particular, **6S** showed 120-times longer half-life time of ee in comparison with carbo[8]helicene (850 h *vs.* 7 h). The counterintuitive conformational stability was also supported by DFT calculations (shown in parentheses in Fig. 1D. Also see Tables S5 and S6‡). According to the theoretical calculations, the isomerizations of the dihetero[8]helicenes proceed in concerted manners without any intermediates *via C*_s_-like transition states (Fig. 1C) whilst the isomerization of carbo[8]helicene is known to be a multistep process (Fig. S25‡).^23^

The observed conformational stability contradicts the conventional understanding that heterohelicenes are conformationally less stable than the corresponding carbohelicene due to the smaller overlap of the terminal rings,^3*a*^ even in the absence of additional stabilizing factors like π-extension of helical framework.^24^ For carbo[*n*]helicenes, the resistance to racemization increases dramatically as the number of aromatic rings increases (Δ*G*^‡^ = 24.1, 36.2 and 41.7 kcal mol^−1^ for *n* = 5, 6, and 7) to reach a plateau (Δ*G*^‡^ = 42.4 and 43.5 kcal mol^−1^ for *n* = 8 and 9).^22^ Given this tendency, factors other than the number of aromatic rings could be determinants for Δ*G*^‡^ for the inversion of helicity of medium-sized helicenes. By applying this hypothesis to our dihetero[8]helicenes, we assume that the substitutions of the two six-membered benzene rings with five-membered heteroaromatic rings lower the freedom of conformation, or flexibility, of molecules, forcing highly strained transition state structures for the inversion of helicity. Contributions of other factors, such as π–π repulsive interaction enhanced by the electron-donating ability of heteroatoms,^25^ cannot be ruled out, and further quantitative study is needed.

**Fig. 2:**
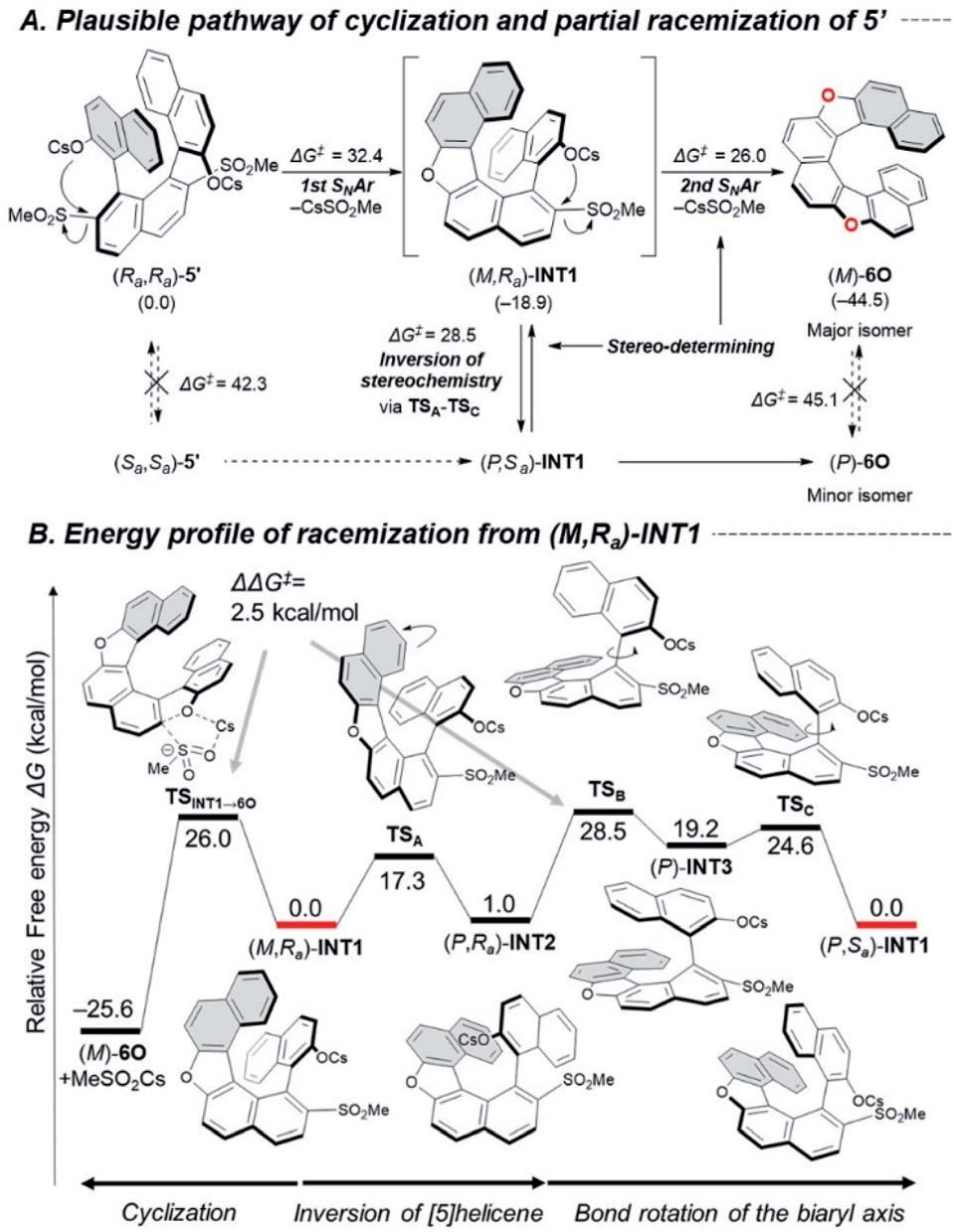
(A) Plausible pathway for the cyclization and partial racemization of **5′**. (B) Detailed energy profile of racemization of (*M*,*R*_a_)-**INT1**. Calculations were conducted at B3LYP-D3(BJ)/6-311+G(2df,p)//B3LYP-D3(BJ)/6-31+G(d) for C, H, O, S and SDD for Cs with SMD (DMF). Δ*G*^‡^ values represent the activation free energies (kcal mol^−1^) of the forward reaction. The numbers in parentheses are the relative free energies (kcal mol^−1^).

These racemization studies revealed the series of dihetero[8]helicenes in this study are sufficiently conformationally stable under synthetic conditions although non-negligible decreases of optical purity were observed in the cyclizations into **6O**, **6N** and **6CX**. These phenomena brought up a question, “in which step does undesired inversion of the axial or helical chirality occur?” To answer this question, we chose cyclization of bissulfone (*R*_*a*_,*R*_*a*_)-**5′** into **6O** as a simplified model reaction and explored possible pathways for the cyclization and competitive stereo-inversion computationally (Fig. 2A).

The pathway for the major product (*M*)-**6O** consists of two sequential S_N_Ar-type cyclizations of (*R*_*a*_,*R*_*a*_)-**5′***via* oxa[5]helicene (*M*,*R*_*a*_)-**INT1**. These steps proceed in concerted manners without formation of Meisenheimer complexes,^26^ and the activation free energies were calculated to be 32.4 and 26.0 kcal mol^−1^, respectively. The stereo-inversion events from starting material (*R*_*a*_,*R*_*a*_)-**5′** to (*S*_*a*_,*S*_*a*_)-**5′** and from product (*M*)-**6O** to (*P*)-**6O** turned out to be unlikely to occur at 130 °C (Δ*G*^‡^ = 42.3 and 45.1 kcal mol^−1^, respectively, at the same level of theory), which indicates that the reaction intermediate (*M*,*R*_*a*_)-**INT1** would be responsible for the partial racemization. The racemization process is composed of three steps (Fig. 2B): ring flipping of the [5]helicene moiety (**TSA**: Δ*G*^‡^ = 17.3 kcal mol^−1^), and a couple of rotations around the biaryl axis (**TSB** and **TSC**: Δ*G*^‡^ = 28.5 and 24.6 kcal mol^−1^, respectively).^27^ Due to the small difference of the calculated activation free energy between the second S_N_Ar-type cyclization (**TSINT1**_→_**6O**, Δ*G*^‡^ = 26.0 kcal mol^−1^) and the stereo-inversion (**TSB**, Δ*G*^‡^ = 28.5 kcal mol^−1^), these processes would be competitive to result in the partial degradation of the optical purity.

To approve the mechanistic scenario experimentally, we monitored the time-course of the optical purities during the cyclization of (*R*_*a*_,*R*_*a*_)-**5** into (*M*)-**6O** (Scheme S2‡). As the result, an **INT1**-like [5]helicene intermediate was not observed even at the early stage of the reaction, which suggests the first cyclization is certainly the rate-determining step for the whole process. We also confirmed that the optical purities of (*R*_*a*_,*R*_*a*_)-**5** and (*M*)-**6O** were almost constant during the reaction ((*R*_*a*_,*R*_*a*_)-**5**: >99% ee, (*M*)-**6O**: 84% ee), which indicates neither (*R*_*a*_,*R*_*a*_)-**5** nor (*M*)-**6O** were the active species for the stereo-inversion, which supports our computational study.

As in the case of **6O**, **INT1**-like [5]helicene intermediates should be formed in the course of the cyclization into **6N** and **6CX**. Therefore, similar racemization pathways can exist in the partial racemization of these compounds. The almost negligible racemization in the synthesis of **6CF** may be due to the rigid structure of the fluorene units that inhibit the axial rotation process.

### Electronic and optical properties

**Fig. 3:**
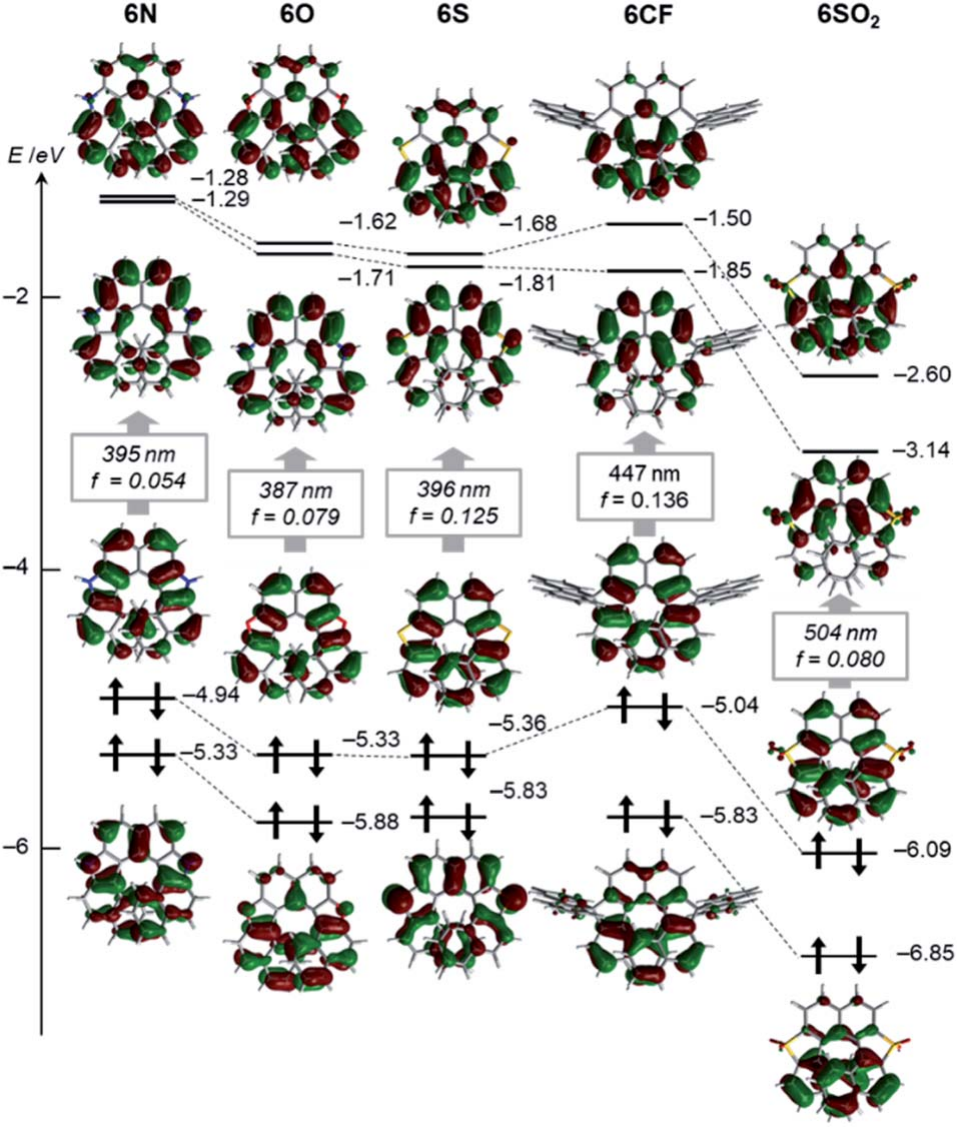
Kohn–Sham orbital representations of a series of helicenes (isovalue: 0.03). The wavelengths and oscillator strengths of the lowest energy electronic transitions obtained by TD-DFT calculations are shown in the box.

Effects of the endocyclic atoms on the electronic states of the dihetero[8]helicenes were investigated. The HOMOs and LUMOs shown in Fig. 3 are well delocalized over the whole helices and have similar orbital coefficients regardless of the embedded atoms and the presence or absence of local aromaticity of the five-membered rings. The relative orbital energy levels of HOMOs and LUMOs of the dihetero[8]helicenes show a clear dependence on the endocyclic atoms while no reversal of the frontier orbitals occurred. As can be inferred from the HOMO and LUMO levels of the corresponding isolated five-membered ring, diazahelicene **6N**, containing the most electron-rich pyrrole moiety, shows the highest HOMO and LUMO energies while those of **6SO2** are strongly stabilized.

**Fig. 4:**
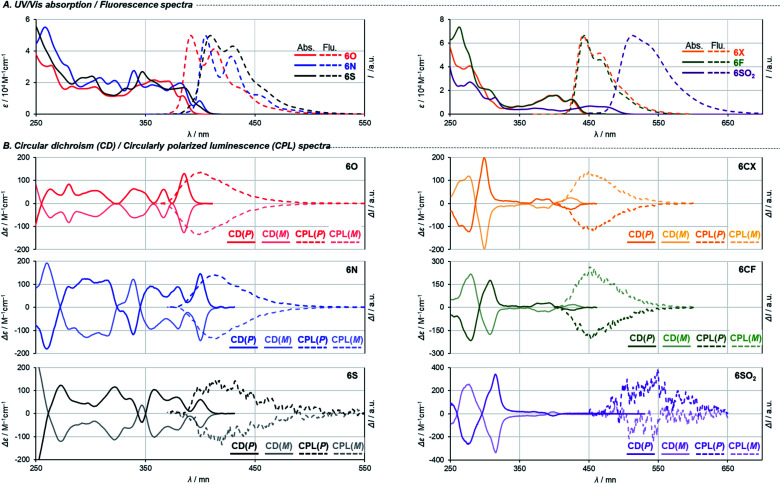
(A) UV/Vis and fluorescence spectra in CH_2_Cl_2_. (B) CD and CPL spectra in CH_2_Cl_2_.

UV/Vis absorption and fluorescence spectra recorded in CH_2_Cl_2_ are shown in Fig. 4A, and (chir)optical properties are summarized in Table 1. Fully aromatic helicenes **6O**, **6N**, and **6S** display absorption bands up to 420 nm that are similar in shape, and the spectra of **6N** and **6S** are slightly red-shifted compared to that of **6O**. The significantly destabilized HOMO of **6N** rather than the destabilization of the LUMO would be the main cause of the red shift. On the other hand, the LUMO and LUMO+1 of **6S** are comparatively stabilized relative to those of **6O**. The helicenes containing non-aromatic rings **6CF**, **6CX**, **6SO2** show absorption bands in low energy regions as expected from their narrower HOMO–LUMO gaps. The trend of the optical band gaps estimated from the absorption spectra was well reproduced by TD-DFT calculation. According to the calculations, the lowest energy absorptions of **6CF**, **6CX**, and **6SO2** are assigned to HOMO → LUMO transition (Tables S10–S12‡).

**Table 1:** Summary of optical properties^a^

Compound	*λ*_abs_ (nm)	*λ*_flu_ b (nm)	Stokes shift (cm^−1^)	*Φ*_F_ c	*τ*_F_ (ns)	*k*_r_ (10^−8^ s^−1^)	*k*_nr_ (10^−8^ s^−1^)	|*g*_abs_|	|*g*_lum_|^b^
**6O**	384	391	466	39%	4.7	0.82	1.3	1.2 × 10^−2^ (390 nm)	6.1 × 10^−3^ (385 nm)
**6N**	399	405	371	13%	3.2	0.40	2.7	1.9 × 10^−2^ (403 nm)	9.5 × 10^−3^ (403 nm)
**6S**	400	408	490	0.6%	0.22	0.27	45	1.7 × 10^−2^ (404 nm)	4.4 × 10^−3^ (414 nm)
**6CX**	424	442	935	62%	6.3	0.99	0.61	9.8 × 10^−3^ (301 nm)	1.3 × 10^−3^ (432 nm)
**6CF**	425	444	981	66% (40%)^d^	6.4	1.03	0.53	8.6 × 10^−3^ (307 nm)	8.6 × 10^−4^ (454 nm)
**6SO2**	470	513	1780	10%	2.6	0.39	3.4	2.4 × 10^−2^ (316 nm)	7.6 × 10^−4^ (545 nm)

a *λ*_abs_: wavelength of the longest absorption maximum, *λ*_flu_: wavelength of the maximum of fluorescence intensity, *Φ*_F_: fluorescence quantum yield (excited at 365 nm), *τ*_F_: fluorescence lifetime, *k*_r_: radiative rate constant, *k*_nr_: non-radiative rate constant, *g*_abs_: absorption dissymmetry factor (Δ*ε*/*ε*), *g*_lum_: luminescence dissymmetry factor (2(*I*_L_ − *I*_R_)/(*I*_L_ + *I*_R_)). Racemic samples were used except for CD and CPL measurements. The values of |*g*_abs_| and |*g*_lum_| shown in Table 1 are the averages of those of two enantiomers.

b Excited at 300 nm.

c Excited at 365 nm.

d In the solid state.

Regarding **6O**, **6N**, **6S**, two different transitions (mixed transitions mainly contributed by HOMO → LUMO or HOMO → LUMO+1 components) are involved in the longest absorption bands due to almost energetically degenerated LUMOs (Tables S7–S9‡). Spiro-shaped helicenes **6CX** and **6CF** show almost identical absorptions in the 350–450 nm region, which indicates the exocyclic fluorene or xanthene moieties do not participate in low energy electronic transitions. This is also supported by almost no contributions of the exocyclic fluorenes for HOMOs and LUMOs (Fig. 3 and S33‡). The fluorescence spectra of these helicenes are characterized by small Stokes shifts and clear vibrational structures, which would reflect their conformational rigidity and small structural deviations from the ground state.

The embedded heteroatoms in each helical skeleton also have a great impact on the efficiency of fluorescence. Dioxa[8]helicene **6O** exhibits a high quantum fluorescence yield of 39% (*cf.* carbo[8]helicene: 1.4% (ref. 28)) while that of **6S** is quite low (0.6%). Interestingly, the oxidation of the sulfide moieties improved fluorescence efficiency (10% for **6SO2**), which is in agreement with earlier reports.^29^ Among helicenes in this study, **6CX** and **6CF** show the highest fluorescence quantum yields of 62% and 66%, respectively. Notably, these are also the highest values among the reported fluorene-based helicene-like compounds.^7*e*,9*a*^ Moreover, **6CF** emitted light blue fluorescence in the solid state (*Φ*_F_ = 40%). To gain further understanding of the element-dependent behaviors in the excited states, we have measured fluorescence lifetimes *τ*_F_ in dichloromethane by using a time-correlated single-photon counting (TCSPC) technique (Table 1 and Fig. S34‡). The fluorescence lifetimes of the series of helicenes in Table 1 are fitted by a single exponential decay component. We found that the low fluorescence efficiency of **6S** is attributed to its incomparably faster non-radiative decay, which may be due to a rapid intersystem crossing process. This process is significantly suppressed by the oxidation of the sulfide moieties, which would be responsible for the increase in the quantum fluorescence yield of **6SO2**.^29^ Spiro-shaped helicene **6CF** exhibits the largest radiative rate constant and the smallest non-radiative rate constant among the series of helicenes, resulting in high fluorescence efficiency.

Finally, we measured circular dichroism (CD) and circularly polarized luminescence (CPL) spectra^1*d*,30^ to examine chiroptical properties and to verify their absolute configurations. Fig. 4B shows the spectra of the helicenes derived from both the first and the second fractions of **4′**. The pairs of enantiomers exhibit sets of mirror-imaged CD and CPL spectra. The three fully conjugated dihetero[8]helicenes **6O**, **6N**, and **6S** show similar CD spectra in terms of their shapes and signs. This is also the case for the other three helicenes **6CX**, **6CF**, and **6SO2**. Judging from the similarities of these spectra, all the chirality conversion events, including axial to helical and helical to helical, certainly proceed with retention of the original configuration of common synthetic intermediate **4**. Comparisons of experimental spectra with simulated ones based on TD-DFT calculation suggest that the absolute configurations of helicenes derived from the second fraction of **4′** (darker line in Fig. 4B) are *P*, which is consistent with the assignment based on the X-ray single crystal diffraction analysis (Fig. S14‡). Regarding dissymmetric factors of absorption *g*_abs_ = Δ*ε*/*ε* (Table 1 and Fig. S36‡), fully conjugated dihetero[8]helicenes **6O**, **6N**, and **6S** exhibit high values (10^−2^-order) compared to values of usual small molecules (10^−3^-order) around the longest absorption maxima. In contrast, |*g*_abs_| values of the other helicenes **6CX**, **6CF**, and **6SO2** reach maxima around 300 nm, and **6SO2** showed the highest value (2.4 × 10^−2^) among the series of helicenes in this study. Interestingly, only carbon-embedded **6CX** and **6CF** showed the opposite Cotton effect around the lowest energy absorption band due to electronic perturbations (Table S13‡). Reflecting the small structural change in the excited states from the ground states, the sign and scale of dissymmetric factors of fluorescence *g*_lum_ = 2(*I*_L_ − *I*_R_)/(*I*_L_ + *I*_R_) show trends similar to those of *g*_abs_ at the lowest energy electronic transitions (Table 1 and Fig. S37‡).^30^ In particular, **6O** and **6N** show both high fluorescence efficiency and |*g*_lum_| values (6.1 × 10^−3^ for **6O** and 9.5 × 10^−3^ for **6N**) among reported helicenes.^1*d*^

## Conclusions

We have achieved asymmetric syntheses of a series of hetero[8]helicenes, containing C, N, O, S, SO_2_ in their helical skeletons. A cascade of sequential interrupted Pummerer reaction/sigmatropic rearrangement that we developed has enabled the facile assembly of highly crowded ternaphthyl common synthetic intermediate **4** for the syntheses of **6O** and **6S**. Oxidations of **6S** afforded another synthetic intermediate **6SO2**, from which **6N**, **6CX**, and **6CF** were derived through replacing the two SO_2_ units with nitrogen and carbon atoms without significant erosion of optical purity.

The obtained dihetero[8]helicenes were subjected to systematic investigations of the structural properties to reveal the influence of the embedded atoms in the common framework. The kinetic studies for racemization dynamics have revealed that the dinaphtho[1,2-*d*:1′,2′-*d*′]naphtho[2,1-*b*:7,8-*b*′]diheterole skeleton exhibits unexpectedly high thermal conformational stability in comparison with carbo[8]helicene. This observation offers a seemingly contradictory viewpoint in designing helicenes: introduction of a smaller 5-membered heterole ring to stabilize helicity.

A series of photophysical measurements have elucidated endocyclic atom-dependent behaviors of (chir)optical properties. The potential utility of our systematic synthetic strategy will be applicable to efficient generations of chemical libraries of π-extended compounds to find ‘hit’ molecules.

## Conflicts of interest

There are no conflicts to declare.

## Supplementary Material

SC-012-D1SC00044F-s001

SC-012-D1SC00044F-s002
