# Supplementary material for: Asymmetric systematic synthesis, structures, and (chir)optical properties of a series of dihetero[8]helicenes
Source: Chem Sci. 2021 Jan 29;12(8):2784–93. doi: 10.1039/d1sc00044f (PMC8179410; doi:10.1039/d1sc00044f)
Supplement: SC-012-D1SC00044F-s001 [file SC-012-D1SC00044F-s001.pdf]

Electronic Supplementary Material (ESI) for Chemical Science.
This journal is © The Royal Society of Chemistry 2021

Asymmetric Systematic Synthesis, Structures, and (Chir)optical Properties of a Series of Dihetero[8]helicenes

Tomoyuki Yanagi, Takayuki Tanaka, Hideki Yorimitsu

Department of Chemistry, Graduate School of Science, Kyoto University, Sakyo-ku, Kyoto 606-8502, Japan.

yori@kuchem.kyoto-u.ac.jp

Note added after first publication: 23/02/2021. On page S27, the value of b [Å] for structure (*P*)-**6S** has been corrected.

Table of Contents

1. Instrumentation and Chemicals S2–3
2. Synthetic Procedure and Characterization S3–15
3. HPLC Trace S15–20
4. X-Ray Crystallographic Details S21–28
5. Racemization Experiments and Theoretical Calculation S29–35
6. Optical Measurement S36–44
7. Reference S45
8. XYZ Coordinates and Energies S46–65
9. NMR Spectra S66–89

1. Instrumentation and Chemicals

^{1}H NMR (600 MHz) and ^{13}C NMR (151 MHz) spectra were recorded on a JEOL ECZ-600 spectrometer. Chemical shifts in ^{1}H NMR spectra were recorded in delta (δ) units, parts per million (ppm) relative to residual $CHCl_3$ (δ = 7.26 ppm), CD_3COCHD_2 (δ = 2.05 ppm), and CD_3SOCHD_2 (δ = 2.50 ppm). Chemical shifts in ^{13}C NMR spectra were recorded in delta (δ) units, parts per million (ppm) relative to $CDCl_3$ (δ = 77.16 ppm), CD_3COCD_3 (δ = 29.84 ppm), and CD_3SOCD_3 (δ = 39.52 ppm). The following abbreviations are used for spin multiplicity: s = singlet, d = doublet, t = triplet, q = quartet, m = multiplet, br = broad. High resolution mass spectra (HRMS) were obtained on a Bruker micrOTOF II-KR spectrometer in Atmospheric Pressure Chemical Ionization (APCI) method using "LC/MS tuning mix, for APCI, low concentration" (Agilent Technologies, Inc.) as the internal standard and Electrospray Ionization (ESI) method using "LC/MS tuning mix, for ESI, low concentration" (Agilent Technologies, Inc.) as the internal standard. UV/Vis absorption spectra were recorded on a Shimadzu UV-1800 spectrometer. Fluorescence spectra were measured on a Shimadzu RF-5300PC spectrometer. Absolute fluorescence quantum yields were determined by a photon-counting method by using an integration sphere on Hamamatsu Photonics C9920-02 spectrometer. Fluorescence lifetime was recorded on a Hamamatsu Photonics Quantaurus-Tau C11367. CD (Circular Dichroism) spectra were measured on a JASCO J-1500 CD Spectrometer. CPL (Circular polarized luminescence) spectra were measured on a JASCO CPL200 Spectrophotometer. For all spectroscopic studies, spectroscopic grade solvents were used as purchased unless otherwise noted. Optical rotation was measured on a JASCO DIP-1000 digital polarimeter. HPLC analysis was performed on a Shimadzu HPLC unit (LC-20AD, SIL-20A, CTO-20A, SPD M20A). X-ray data were taken at –180 ˚C with a Rigaku XtaLAB P-200 system by using graphite monochromatic Cu-Kα radiation (λ = 1.54187 Å). The structures were solved by using direct method SIR-97 and refined by SHELXL-2014/7 program.[1]

All non-aqueous reactions were carried out under an inert atmosphere of N_2 gas in oven-dried glassware unless otherwise noted. Dehydrated solvents, 1,4-dioxane, dichloromethane, and toluene, were purchased from FUJIFILM Wako Pure Chemical Corporation and stored under nitrogen atmosphere. Dehydrated THF was purchased from Kanto Chemical Co., Inc. and stored under nitrogen atmosphere. 1,3-Dimethyl-2-imidazolidinone (DMI) was purchased from common commercial suppliers and distilled before use. Tetraglyme was purchased from FUJIFILM Wako Pure Chemical Corporation and stored under nitrogen atmosphere in the presence of MS4A after removal of oxygen by nitrogen bubbling. All other reagents were commercially available and used without further purification unless otherwise noted. Analytical

thin layer chromatography (TLC) was performed on Merck precoated analytical plates, 0.25-mm thick, silica gel 60 F_{254}. Preparative flash chromatography was performed using Silica Gel (Wakosil® C-300 purchased from FUJIFILM Wako Pure Chemical Corporation and Alumina (activated 200 purchased from Nacalai Tesque, Inc.).

All calculations were carried out using the Gaussian 16 program[2] and GRRM 17 program[3] associated with Gaussian16 program. Geometry optimizations and frequency calculations were conducted using B3LYP-D3(BJ) as a functional. Calculated frequencies were used to verify the nature of all stationary points as either minima (no imaginary frequencies) or transition states (one imaginary frequency). IRC calculations were performed to further confirm whether the transition state is connected to the corresponding intermediates. Images were created using the CYLview software.[4]

2. Synthetic Procedure and Characterization

Synthesis of bissulfide 2: Acid-catalyzed dehydrative sulfanylation

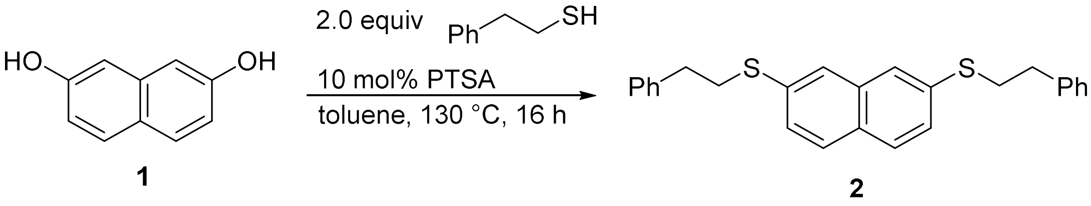

A modified procedure of the reported method was used.[5] A 100-mL Schlenk flask was charged with 2,7-dihydroxynaphthalene (14.4 g, 90 mmol), *p*-toluenesulfonic acid monohydrate (1.70 g, 9.0 mmol), 2-phenylethanethiol (25.0 mL, 180 mmol), and toluene (90 mL). The mixture was stirred at 130 °C for 27 h and cooled to room temperature. The reaction mixture was diluted with dichloromethane (50 mL), passed through a pad of neutral alumina (eluent: dichloromethane), and concentrated under reduced pressure to give 2,7-bis(2-phenylethylsulfanyl)naphthalene **2** as an orange solid with little impurities. This crude sulfide was used for the next step without further purification.

^{1}H NMR ($CDCl_3$): δ 7.71 (d, J = 7.5 Hz, 2H), 7.64 (d, J = 1.4 Hz, 2H), 7.39 (dd, J = 7.5, 1.4 Hz, 2H), 7.32 (t, J = 7.5 Hz, 4H), 7.24 (t, J = 7.5 Hz, 6H), 3.28 (t, J = 7.5 Hz, 4H), 2.99 (t, J = 7.5 Hz, 4H).

^{13}C NMR ($CDCl_3$): δ 140.2, 135.1, 134.3, 130.1, 128.7, 128.7, 128.4, 127.0, 126.7, 125.7, 35.7, 35.0

HRMS (APCI-MS, positive): *m/z* [*M*]$^+$ Calcd for $C_{26}H_{24}S_2$: 400.1314; Found: 400.1331.

Synthesis of bissulfoxide 3: Oxidation of bissulfide 2

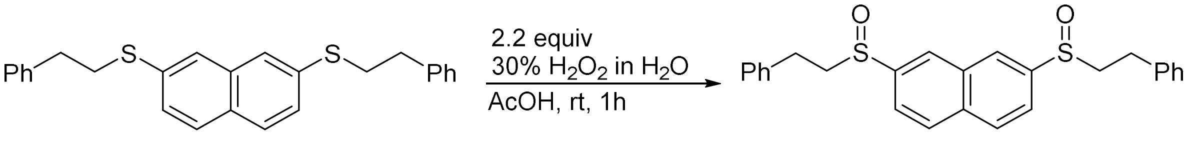

2 3

The crude sulfide **2** obtained above (ca. 90 mmol) was dissolved in AcOH (180 mL), and aqueous 30% H_2O_2 (22.4 mL, 198 mmol) was added to the solution slowly via a dropping funnel. The resulting solution was stirred at room temperature, and the progress of the oxidation was checked by TLC. After completion of the reaction, the mixture was poured into water (300 mL) and extracted with CH_2Cl_2 (100 mL × 3). The combined organic layer was washed with water (300 mL). To the organic layer, water (100 mL) was added again. Na_2CO_3 (solid) were then added until AcOH in the organic layer was completely neutralized, and remaining H_2O_2 was quenched by sat. aqueous $Na_2S_2O_3$ (30 mL). After the phase separation, the organic layer was dried over Na_2SO_4 and concentrated under reduced pressure to give 2,7-bis(phenethylsulfinyl)naphthalene **3** (37 g, ca. 85 mmol) as an orange solid with a little impurities. This crude sulfoxide was used for the next step without further purification.

^{1}H NMR ($CDCl_3$): δ 8.23 (s, 2H), 8.00 (d, *J* = 8.2 Hz, 2H), 7.68 (d, *J* = 8.2 Hz, 2H), 7.22 (t, *J* = 7.5 Hz, 4H), 7.16–7.12 (m, 6H), 3.18–3.05 (m, 6H), 2.88–2.84 (m, 2H)
^{13}C NMR ($CDCl_3$): δ 142.6, 138.3, 135.1, 132.2, 129.5, 128.6, 128.4, 126.6, 124.9, 121.6, 57.7, 27.9.
Although bissulfoxide **3** was obtained as a mixture of diastereomers (*cis*- and *trans*-isomer), they are almost not distinguishable by ^{1}H and ^{13}C NMR due to overlapping.
HRMS (APCI-MS, positive): *m/z* $[M+H]^+$ Calcd for $C_{26}H_{25}O_2S_2$: 433.1290; Found: 433.1284.

Synthesis of ternaphthalene 4: Double arylation of bissulfoxide 3

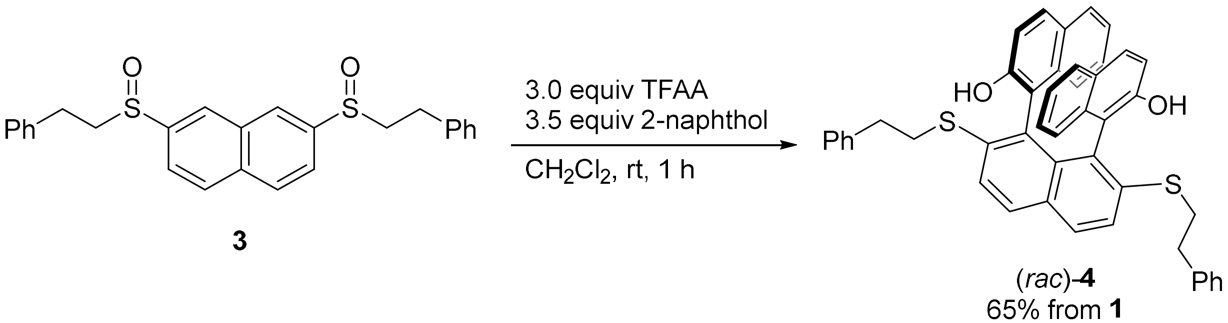

3

(*rac*)-**4**
65% from **1**

A modified procedure of the reported method was used.[5] A 2-L flask was charged with the obtained crude sulfoxide **3** (37 g, ca. 85 mmol), 2-naphthol (42.8 g, 298 mmol), and CH_2Cl_2

(1.7 L, normal grade). Trifluoroacetic anhydride (35.7 mL, 255 mmol) was then slowly added to the solution at 25 °C via a drop funnel, and the resulting mixture was stirred for 1 h at room temperature. After completion of the reaction, ca. 1.3 L of dichloromethane was removed by a rotary evaporator. *n*-Hexane (200 mL) was added to result in formation of white precipitation of **4**. The precipitation was filtered, washed with *n*-hexane, and dried under reduced pressure at 80 °C to give ternaphthalene **4** (39.9 g, 58.2 mmol, 65% from **1**) as a white solid.

^{1}H NMR ($CDCl_3$): δ 8.06 (d, J = 8.2 Hz, 2H), 7.61 (d, J = 8.2 Hz, 4H), 7.28 (t, J = 8.2 Hz, 2H), 7.25-7.23 (m, 4H), 7.21-7.15 (m, 6H), 7.08 (d, J = 8.2 Hz, 4H), 6.97 (d, J = 8.2 Hz, 2H), 6.18 (d, J = 8.2 Hz, 2H), 4.03 (s, 2H), 3.03–2.99 (m, 4H), 2.73–2.70 (m, 4H).
^{13}C NMR ($CDCl_3$): 150.2, 141.9, 140.3, 135.2, 132.9, 130.8, 130.7, 129.3, 128.8, 128.6, 128.5, 127.9, 126.8, 126.6, 125.9, 124.5, 123.2, 122.9, 119.0, 116.7, 35.5, 33.6.
HRMS (APCI-MS, positive): *m/z* $[M]^+$ Calcd for $C_{46}H_{36}O_2S_2$: 684.2151; Found: 684.2131.

Synthesis of bissulfonate ester 4': Introduction of chiral auxiliaries

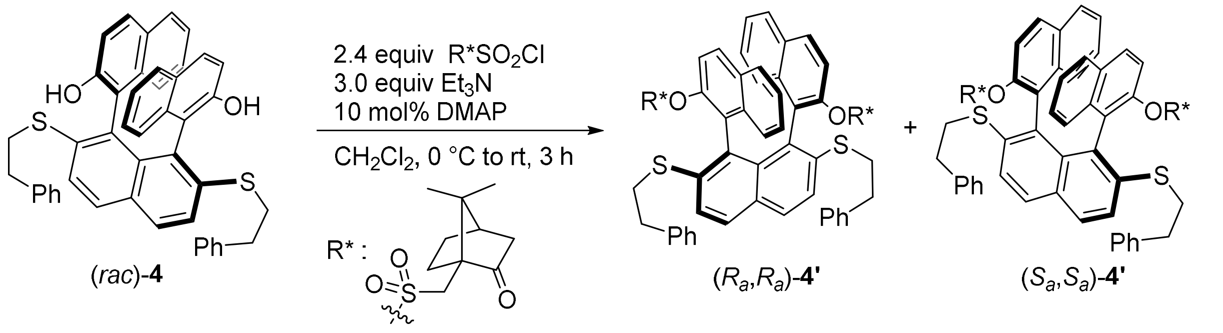

A 100-mL flask was charged with **4** (4.10 g, 6.00 mmol), Et_3N (2.49 mL, 18.0 mmol), DMAP (73.3 mg, 0.60 mmol), and CH_2Cl_2 (100 mL). To the solution, (*1S*)-(+)-10-camphorsulfonyl chloride (3.60 g, 12 mmol) in CH_2Cl_2 (20 mL) was slowly added at 0 °C. The resulting mixture was allowed to warm up to room temperature and stirred for 3 h. Aqueous HCl (3 M, 100 mL) was then added, and the resulting biphasic solution was extracted with CH_2Cl_2 (50 mL × 3). The combined organic layer was dried over Na_2SO_4 and concentrated under reduced pressure. The crude mixture was purified by column chromatography on silica gel (eluent: toluene : *n*-*n*-hexane : EtOAc = 10:10:3) to provide (R_a,R_a)-**4'** (R_f: 0.31, 3.05 g, 2.74 mmol , 46%, d.r. > 20:1, $[\alpha]_D^{26}$ = +92.0 (c = 0.39, $CHCl_3$)) as a white solid and (S_a,S_a)-**4'** (R_f: 0.21, 2.93 g, 2.63 mmol , 44%, d.r. > 20:1, $[\alpha]_D^{26}$ = –63.4 (c = 0.45, $CHCl_3$)) as a white solid.

(R_a,R_a)-**4'**

^{1}H NMR (CDCl$_3$):δ 7.94 (d, J = 8.2 Hz, 2H), 7.69–7.67 (m, 2H), 7.56 (d, J = 8.9 Hz, 2H), 7.52–7.50 (m, 2H), 7.40–7.36 (m, 4H), 7.22 (t, J = 7.2 Hz, 4H), 7.16 (t, J = 7.2 Hz, 2H), 7.10 (d, J = 6.9 Hz, 4H), 6.95 (d, J = 8.9 Hz, 2H), 6.31 (d, J = 8.9 Hz, 2H), 3.23 (d, J = 15.1 Hz, 2H), 3.18–3.13 (m, 2H), 3.03–2.98 (m, 2H), 2.77–2.72 (m, 4H), 2.32 (d, J = 15.1 Hz, 2H), 2.28 (dt, J = 18.5, 3.8 Hz, 2H), 2.09–2.04 (m, 2H), 1.99 (t, J = 4.5 Hz, 2H), 1.90–1.86 (m, 2H), 1.80 (d, J = 18.5 Hz, 2H), 1.27–1.21 (m, 4H), 0.94 (s, 6H), 0.73 (s, 6H).

^{13}C NMR (CDCl$_3$):δ 213.9, 144.9, 140.6, 140.3, 135.7, 132.1, 130.4, 130.3, 129.8, 128.6, 128.6, 128.1, 127.9, 127.8, 127.7, 127.5, 126.5, 126.4, 126.0, 123.0, 119.8, 58.2, 48.9, 47.7, 43.1, 42.5, 35.4, 34.2, 27.0, 25.1, 20.1, 19.7.

HRMS (APCI-MS, positive): m/z $[M]^+$ Calcd for $C_{66}H_{64}O_8S_4$: 1112.3479; Found: 1112.3466.

(S_a,S_a)-**4'**

^{1}H NMR (CDCl$_3$): δ 8.01 (d, J = 8.9 Hz, 2H), 7.73 (t, J = 4.8 Hz, 2H), 7.56 (d, J = 8.9 Hz, 2H), 7.53–7.52 (m, 2H), 7.40–7.37 (m, 4H), 7.24 (t, J = 7.5 Hz, 4H), 7.18 (t, J = 7.2 Hz, 2H), 7.11 (d, J = 7.5 Hz, 4H), 6.96 (d, J = 9.6 Hz, 2H), 6.28 (d, J = 8.9 Hz, 2H), 3.16 (td, J = 11.8, 5.5 Hz, 2H), 2.94 (td, J = 11.8, 5.7 Hz, 2H), 2.84–2.79 (m, 4H), 2.72–2.68 (m, 2H), 2.64 (d, J = 14.4 Hz, 2H), 2.29 (dt, J = 18.3, 3.9 Hz, 2H), 2.26–2.21 (m, 2H), 2.00 (t, J = 4.5 Hz, 2H), 1.95–1.90 (m, 2H), 1.85 (d, J = 18.5 Hz, 2H), 1.38–1.29 (m, 4H), 0.95 (s, 6H), 0.74 (s, 6H).

^{13}C NMR (CDCl$_3$): δ 214.0, 145.3, 140.3, 139.6, 135.8, 131.8, 130.5, 130.4, 130.2, 128.6, 128.5, 128.1, 128.0, 127.9, 127.5, 127.5, 126.6, 126.4, 126.0, 123.0, 120.0, 58.0, 48.6, 47.8, 42.8, 42.5, 35.5, 34.1, 27.0, 24.8, 19.9, 19.6.

HRMS (APCI-MS, positive): m/z $[M]^+$ Calcd for $C_{66}H_{64}O_8S_4$: 1112.3479; Found: 1112.3468.

Synthesis of enantio-enriched 4: Removal of chiral auxiliaries of 4'

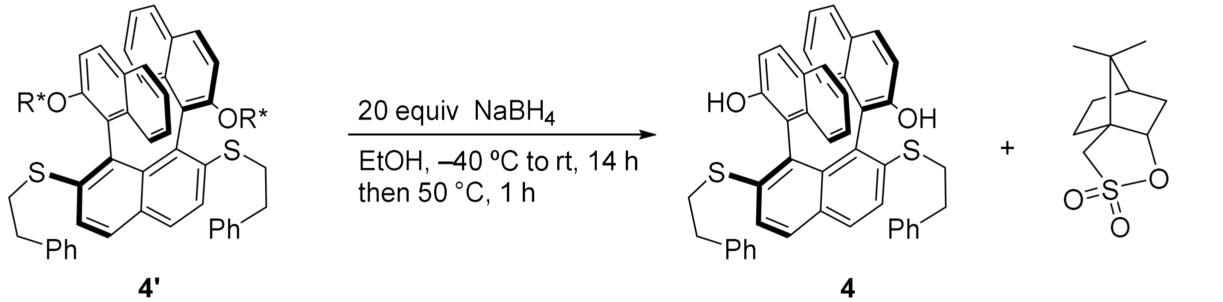

4' **4**

Synthesis of (S_a,S_a)-**4** is representative. A modified procedure of the reported method was used.[6] A 50-mL flask was charged with (S_a,S_a)-**4'** (3.52 g, 3.17 mmol) and EtOH (60 mL). To the solution, $NaBH_4$ (2.40 g, 63 mmol) was added slowly at –40 °C. The resulting mixture was allowed to warm up to room temperature and stirred for 14 h at room temperature and 1 h at

50 °C. Aqueous sat. NH_4Cl (10 mL) and water (10 mL) were then added. The precipitates were filtered off and washed by water (10 mL) and *n*-hexane (10 mL). The crude mixture was purified by column chromatography on silica gel (eluent: toluene/EtOAc = 10:1) to provide (*S*,*S*)-**4** (1.45 g, 2.1 mmol, 67%, >99%ee, $[\alpha]_D^{25}$ = –123 (c = 0.21, $CHCl_3$)) as a white solid. The ^{1}H NMR spectrum of (S_a,S_a)-**4** is identical to that of (*rac*)-**4**.

Desulfonylation of (*R*,*R*)-**4'** was also performed according to the same procedure to afford (R_a,R_a)-**4** (820 mg, 1.20 mmol, 69%, >99%ee, $[\alpha]_D^{25}$ = +95.0 (c = 0.20, $CHCl_3$)) from (R_a,R_a)-**4'** (1.94 g, 1.74 mmol).

Synthesis of 5: Oxidation of sulfanyl moieties of 4

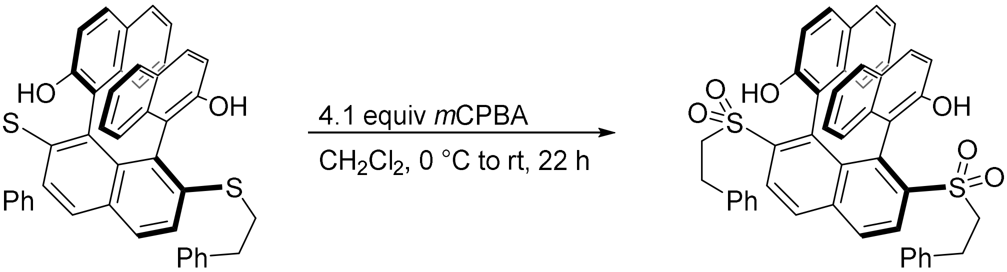

(S_a, S_a)-**4**　　　　　(S_a, S_a)-**5**

Synthesis of (S_a,S_a)-**5** is representative. A 100-mL flask was charged with (S_a,S_a)-**4** (1.03 g, 1.5 mmol) and CH_2Cl_2 (30 mL). To the solution, *m*CPBA (ca. 70%, stabilized by water, 1.50 g, ca. 6.1 mmol) was added slowly at 0 °C. The resulting solution was stirred at the same temperature for 10 min and at room temperature for 22 h. After completion of the reaction, sat. aqueous $NaHCO_3$ (60 mL) and sat. aqueous $Na_2S_2O_3$ (20 mL) were added. The resulting biphasic mixture was extracted with CH_2Cl_2 (50 mL × 3). The combined organic layer was washed with brine and dried over Na_2SO_4. The solution was concentrated under reduced pressure. The crude mixture was purified by column chromatography on silica gel (eluent: CH_2Cl_2: EtOAc=10:1) to afford (S_a, S_a)-**5** as a pale yellow solid (711 mg 0.95 mmol, 63%, $[\alpha]_D^{25}$ = +181 (c = 0.22, $CHCl_3$)).

(R_a,R_a)-**5** and (*rac*)-**5** were synthesized according to the same procedure.

(R_a,R_a)-**5** was obtained as a pale yellow solid (698 mg, 0.93 mmol, 62%, $[\alpha]_D^{25}$ = –183 (c = 0.18, $CHCl_3$)) from (S_a,S_a)-**4** (1.03 g, 1.5 mmol).

(*rac*)-**5** was obtained as a pale yellow solid (1.10 g, 1.46 mmol, 73%) from (*rac*)-**4** (1.37 g, 2.0 mmol).

^{1}H NMR ($CDCl_3$): δ 8.67 (d, J = 8.9 Hz, 2H), 8.42 (d, J = 8.9 Hz, 2H), 7.72 (d, J = 8.9 Hz, 2H), 7.43 (d, J = 8.9 Hz, 2H), 7.28 (d, J = 8.9 Hz, 2H), 7.07–7.01 (m, 8H), 6.36–6.34 (m, 6H), 6.31 (d, J = 8.9 Hz, 2H), 4.55 (s, 2H), 2.61 (td, J = 13.0, 4.8 Hz, 2H), 2.42 (td, J = 13.0, 4.8 Hz, 2H), 2.34 (td, J = 13.0, 4.8 Hz, 2H), 2.18 (td, J = 13.0, 4.8 Hz, 2H).
^{13}C NMR ($CDCl_3$): δ 151.0, 142.4, 139.1, 138.5, 136.9, 135.9, 134.4, 131.1, 131.0, 129.2, 128.8, 128.5, 128.4, 127.9, 126.8, 126.7, 124.4, 124.1, 119.9, 118.0, 55.1, 28.0.
HRMS (APCI-MS, positive): m/z $[M]^+$ Calcd for $C_{46}H_{36}O_6S_2$: 748.1948; Found: 748.1945.

Synthesis of 6O: Base-mediated cyclization of 5

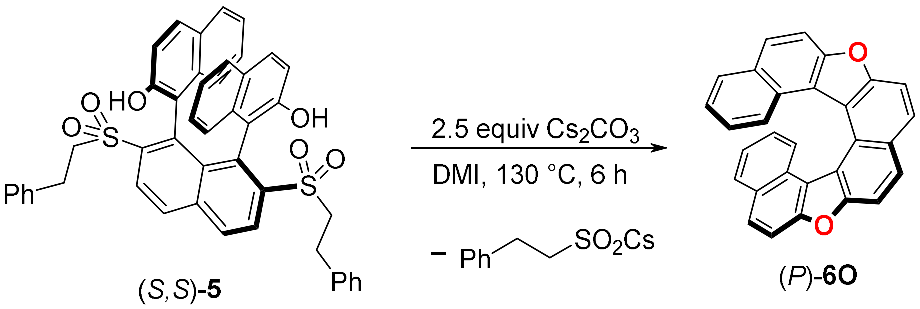

(S,S)-**5** (P)-**6O**

Synthesis of (P)-**6O** is a representative. A modified procedure of the reported method was used.[5] A 20-mL Schlenk tube was charged with (S_a,S_a)-**5** (449 mg, 0.60 mmol), Cs_2CO_3 (487 mg, 1.5 mmol), and DMI (12 mL). The resulting solution was stirred at room temperature for 10 min and at 130 °C for 6 h. After completion of the reaction, aqueous sat. NH_4Cl (10 mL) and water (100 mL) were added, and the resulting mixture was extracted with EtOAc/ n-hexane (ratio ca. 5:1, 30 mL × 3). The combined organic layer was washed with water and brine, dried over Na_2SO_4, and concentrated under reduced pressure. The crude mixture was purified by column chromatography on silica gel (eluent: n-hexane: CH_2Cl_2 = 10:1) to afford (P)-**6O** as a white solid (151 mg, 0.37 mmol, 62%, 84%ee), which showed ^{1}H NMR and ^{13}C NMR spectra identical to the reported ones.[5] Recrystallization from CH_2Cl_2/ n-hexane increased the optical purity of the filtrate (125 mg, 97%ee, $[\alpha]_D^{22}$ = +1560 (c =0.10, $CHCl_3$)) due to higher crystallinity of the racemate (The precipitate: 27 mg, 21%ee).

(R_a,R_a)-**5** and (rac)-**5** were synthesized according to the same procedure.
(M)-**6O** was obtained as a white solid (154 mg, 0.38 mmol, 63%, 83%ee from with (S_a,S_a)-**5** (449 mg, 0.60 mmol). Recrystallization from CH_2Cl_2/ n-hexane increased the optical purity of the filtrate (106 mg, 97%ee, $[\alpha]_D^{22}$ = –1495 (c = 0.10, $CHCl_3$)).
(rac)-**6O** was obtained as a white solid (30.5 mg, 0.074 mmol, 56%) from (rac)-**6SO₂** (100 mg, 0.13 mmol).

For the chiroptical measurements, the enantiopure sample was prepared by preparative chiral HPLC. (Column: DAICEL CHIRALPAK IA (20 mm × 250 mm), Eluent: *n*-Hexane/dichloromethane= 4:1, Flow rate: 6.0 mL/min, Detection: 254 nm, Retention time: t_1=15.3 min, t_2=17.8 min.)

Synthesis of 7: Deprotection of the thiol moieties of 4

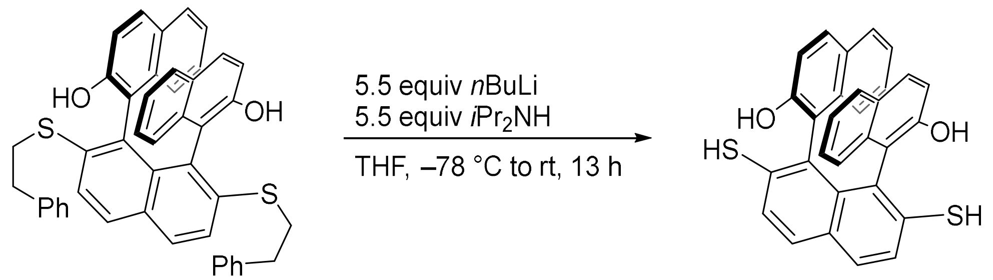

(*rac*)-**4** (*rac*)-**7**

Synthesis of (*rac*)-**7** is representative. A 200-mL two-necked flask was charged with diisopropylamine (7.7 mL, 55 mmol) and THF (55 mL). To the solution, *n*BuLi (1.6 M in *n*-hexane, 34 mL, 55 mmol) was added slowly at –78 °C. The resulting solution was stirred at the same temperature for 10 min and at 0 °C for 10 min. Ternaphthalene **4** (6.84 g, 10.0 mmol) was then added portionwise at –78 °C. The resulting mixture was stirred for 10 min at the same temperature and for 13 h at room temperature. After completion of the reaction, sat. aqueous NH_4Cl (60 mL) was added, and the resulting biphasic solution was extracted with EtOAc (100 mL × 3). The combined organic layer was washed with brine, dried over Na_2SO_4. The solution was concentrated under reduced pressure to ca. 30 mL, and precipitation was then filtered and washed with diethyl ether to afford bisthiol **7** (4.34 g, 9.1 mmol, 91%) as a pale yellow solid.

(R_a,R_a)-**7** and (S_a,S_a)-**7** were synthesized according to the same procedure.

(*S*,*S*)-**7** was obtained as a pale yellow solid (1.06 g, quantitative yield, $[\alpha]_D^{19}$ = +121 (c = 0.20, $CHCl_3$)) from (S_a,S_a)-**4** (1.43 g, 2.12 mmol).

(*R*,*R*)-**7** was obtained as a pale yellow solid (552 mg, 1.16 mmol, 97%, $[\alpha]_D^{26}$ = –132 (c = 0.20, $CHCl_3$)) from (R_a,R_a)-**4** (821 mg, 1.20 mmol).

^{1}H NMR ($CDCl_3$): δ 7.90 (d, *J* = 8.9 Hz, 2H), 7.57–7.54 (m, 4H), 7.26 (t, *J* = 6.9 Hz, 2H), 7.20 (t, *J* = 7.5 Hz, 2H), 7.10 (d, *J* = 8.9 Hz, 2H), 6.98 (d, *J* = 8.2 Hz, 2H), 6.13 (d, *J* = 8.9 Hz, 2H), 4.02 (s, 2H), 3.16 (s, 2H).

^{13}C NMR ($CDCl_3$): δ 150.0, 138.4, 135.6, 132.2, 131.7, 130.6, 129.5, 128.8, 127.9, 127.0, 126.3, 125.6, 124.3, 123.5, 119.2, 116.8.

HRMS (APCI-MS, positive): *m/z* $[M]^+$ Calcd for $C_{30}H_{20}O_2S_2$: 476.0899; Found: 476.0901.

Synthesis of 6S: Acid-mediated cyclization of bisthiol of 7

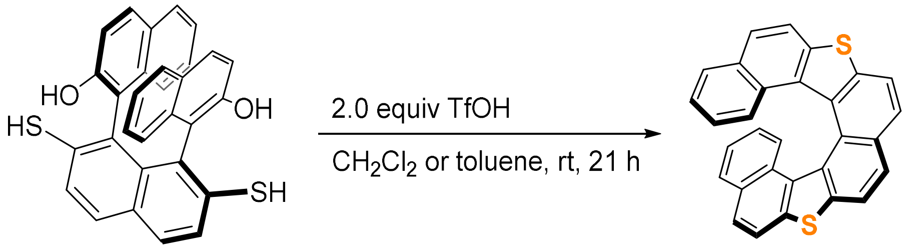

(*rac*)-**7** (*rac*)-**6S**

Synthesis of (*rac*)-**6S** is representative. A 200-mL two-necked flask was charged with bisthiol **7** (4.34 g, 9.1 mmol) and CH_2Cl_2 (90 mL). TfOH (1.6 mL, 18 mmol) was then added at 0 °C, and the resulting mixture was stirred for 21 h at room temperature. The reaction mixture was then diluted with CH_2Cl_2 (100 mL), passed through a pad of neutral alumina (eluent: CH_2Cl_2), and concentrated under reduced pressure to afford (*rac*)-**6S** as a pale yellow solid (3.10 g, 7.0 mmol, 77%). Samples for optical measurements were purified by recrystallization from CH_2Cl_2/ *n*-hexane.

(*P*)-**6S** and (*M*)-**6S** were synthesized according to the same procedure using toluene as a solvent instead of CH_2Cl_2.

(*P*)-**6S** was obtained as a pale yellow solid (767 mg, 1.74 mmol, 87%, >99%ee, $[\alpha]_D^{33}$ = +1940 (c = 0.10, $CHCl_3$)) from (S_a,S_a)-**7** (952 mg, 2.0 mmol).

(*M*)-**6S** was obtained as a pale yellow solid (393 mg, 0.89 mmol, 89%, >99%ee, $[\alpha]_D^{33}$ = –1840 (c = 0.11, $CHCl_3$)) from (R_a,R_a)-**7** (476 mg, 1.0 mmol).

^{1}H NMR (DMSO-*d*$_6$): δ 8.48 (d, *J* = 8.2 Hz, 2H), 8.41 (d, *J* = 8.2 Hz, 2H), 8.06 (d, *J* = 8.2 Hz, 2H), 7.62 (d, *J* = 8.2 Hz, 2H), 7.43 (d, *J* = 8.2 Hz, 2H), 6.92 (t, *J* = 8.2 Hz, 2H), 6.40 (d, *J* = 8.2 Hz, 2H), 6.19 (t, *J* = 8.2 Hz, 2H).

^{13}C NMR (DMSO-*d*$_6$): δ 138.0, 135.6, 132.5, 130.5, 129.5, 129.1, 128.2, 127.3, 127.1, 126.4, 124.6, 123.9, 122.9, 122.7, 120.6, 120.1.

HRMS (APCI-MS, positive): *m/z* $[M]^+$ Calcd for $C_{30}H_{16}S_2$: 440.0688; Found: 440.0710.

Synthesis of 6SO$_2$: Oxidation of 6S

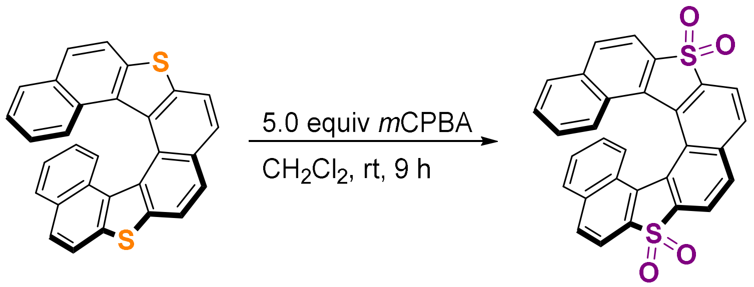

(*P*)-**6S** (*P*)-**6SO2**

Synthesis of (*P*)-**6SO2** is representative. A 100-mL flask was charged with (*P*)-**6S** (739 mg, 1.73 mmol) and CH_2Cl_2 (70 mL). To the solution, *m*CPBA (ca. 70%, stabilized by water, 2.13 g, ca. 8.7 mmol) was added slowly at 0 °C. The resulting solution was stirred at the same temperature for 10 min and at room temperature for 9 h. After completion of the reaction, sat. aqueous $NaHCO_3$ (60 mL) and sat. aqueous $Na_2S_2O_3$ (20 mL) were added. The resulting biphasic mixture was extracted with CH_2Cl_2 (50 mL × 3). The combined organic layer was washed with brine and dried over Na_2SO_4. The solution was passed through a pad of neutral alumina and silica gel (eluent CH_2Cl_2) and concentrated under reduced pressure to afford (*P*)-**6SO2** as an orange solid (855 mg 1.69 mmol, 98%, >99%ee, $[\alpha]_D^{33}$ = +858 (c = 0.098, $CHCl_3$)). Samples for optical measurement were purified by recrystallization (CH_2Cl_2/ EtOAc).
Dithiahelicene tetraoxide (*M*)-**6SO2** was synthesized according to the same procedure and obtained as an orange solid (552 mg, 1.10 mmol, 99%, >99%ee, $[\alpha]_D^{33}$ = –964 (c = 0.11, $CHCl_3$)) from (*M*)-**6S** (480 mg, 1.09 mmol).

(*rac*)-**6SO2** was synthesized according to the procedure shown below.

A 200-mL flask equipped with a reflux condenser was charged with (*rac*)-**6S** (2.2 g, 5.0 mmol) and AcOH (40 mL). 30% aqueous H_2O_2 (6.0 mL, ca. 53 mmol) was added slowly, and the resulting mixture was stirred at 120 °C (bath temp.) for 16 h. After completion of the reaction, the mixture was cooled to room temperature. The formed precipitate was filtered, washed with Et_2O, and dried under reduced pressure to afford (*rac*)-**6SO2** (2.34 g, 4.6 mmol, 93%) as an orange solid. The obtained product contains little impurity, which was used for the next steps without further purification due to very poor solubility (enantio-enriched **6SO2** showed much better solubility than racemic **6SO2**). For optical measurements, a part of the obtained product was purified by column chromatography on silica gel (eluent: CH_2Cl_2).

^{1}H NMR (DMSO-d_6): δ 8.59 (d, J = 8.2 Hz, 2H), 8.39 (d, J = 8.2 Hz, 2H), 7.84 (d, J = 8.2 Hz, 2H), 7.80 (d, J = 8.2 Hz, 2H), 7.62 (d, J = 8.2 Hz, 2H), 7.54 (d, J = 8.2 Hz, 2H), 7.21 (t, J = 8.2 Hz, 2H), 6.82 (t, J = 8.2, 2H).

^{13}C NMR (DMSO-d_6): δ 140.1, 138.5, 134.9, 133.4, 133.0, 132.3, 131.2, 130.4, 127.8, 127.6, 127.2, 126.6, 124.4, 124.0, 119.3, 116.1.

HRMS (APCI-MS, positive): m/z $[M]^+$ Calcd for $C_{30}H_{16}O_4S_2$: 504.0485; Found: 504.0494.

Synthesis of 6N: Atom-substitution by amine via sequential 4-fold S$_N$Ar reactions

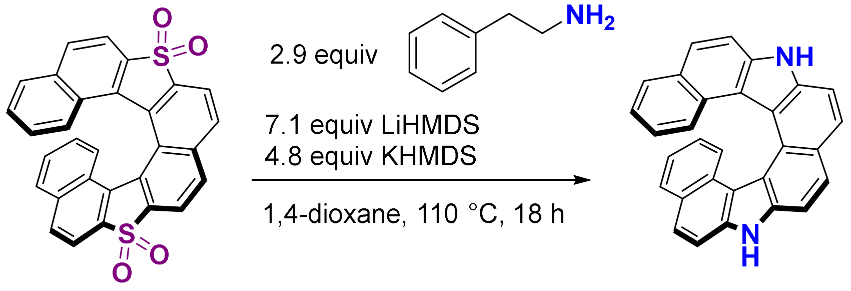

(*P*)-**6SO$_2$** (*P*)-**6N**

Synthesis of (*P*)-**6N** is representative. A modified procedure of the reported method was used.[7] A 10-mL Schlenk tube was charged with (*P*)-**6SO$_2$** (212 mg, 0.42 mmol), 2-phenylethylamine (0.15 mL, 1.2 mmol), LiHMDS (498 mg, 3.0 mmol), and 1,4-dioxane (5.0 mL). The mixture was stirred at 110 °C for 6 h. KHMDS (398 mg, 2.0 mmol) was then added, and the mixture was stirred for an additional 12 h. The reaction was terminated by addition of sat. aqueous NH_4Cl (5 mL) at room temperature, and the resulting biphasic solution was extracted with CH_2Cl_2 (5 mL × 3). The combined organic layer was washed with brine, dried over Na_2SO_4, and concentrated under reduced pressure. The residue was purified by column chromatography on silica gel (eluent: *n*-hexane: EtOAc = 3:1) to provide (*P*)-**6N** (39.1 mg, 0.096 mmol, 23%, 77%ee) as a pale yellow solid. Recrystallization from CH_2Cl_2/ *n*-hexane afforded the enantiopure product (16.2 mg, >99%ee, $[\alpha]_D^{22}$ = +1670 (c = 0.030, $CHCl_3$)).

(*M*)-**6N** and (*rac*)-**6N** were synthesized according to the same procedure.

(*M*)-**6N** was obtained as a pale yellow solid (40.7 mg, 0.10 mmol, 24%, 76%ee) from (*M*)-**6SO$_2$** (512 mg, 0.42 mmol). Recrystallization from CH_2Cl_2/ *n*-hexane afforded enantiopure product (15.6 mg, >99%ee, $[\alpha]_D^{22}$ = –1810 (c = 0.050, $CHCl_3$)).

(*rac*)-**6N** was obtained as a pale yellow solid (139 mg, 0.34 mmol, 34%) from (*rac*)-**6SO$_2$** (504 mg, 1.0 mmol) using LiHMDS (1.0 g, 6.0 mmol), KHMDS (1.2 g, 6.0 mmol), and 1,4-dioxane (8.0 mL).

^{1}H NMR (Acetone-d_6): δ 11.37 (s, 2H), 8.13 (d, J = 8.2 Hz, 2H), 7.94 (d, J = 8.2 Hz, 2H), 7.89 (d, J = 8.2 Hz, 2H), 7.73 (d, J = 8.2 Hz, 2H), 7.48 (d, J = 8.2 Hz, 2H), 7.28 (d, J = 8.2 Hz, 2H), 6.76 (t, J = 8.2 Hz, 2H), 6.20 (t, J = 8.2 Hz, 2H).

^{13}C NMR (Acetone-d_6): δ 139.0, 136.4, 130.9, 129.5, 127.8, 127.3, 126.9, 126.6, 125.6, 125.1, 123.0, 122.5, 121.3, 117.7, 113.4, 110.6.

HRMS (APCI-MS, positive): m/z $[M]^+$ Calcd for $C_{30}H_{18}N_2$: 406.1465; Found: 406.1473.

Synthesis of 6CX: Atom-substitution by xanthene via sequential 4-fold S_NAr reactions

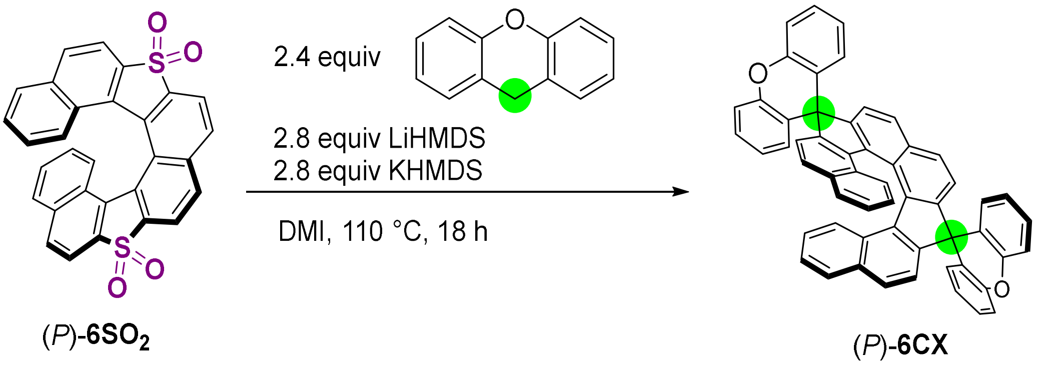

Synthesis of (P)-**6CX** is representative. A Schlenk flask was charged with LiHMDS (149 mg, 0.90 mmol) and DMI (10 mL). To the flask, xanthene (163 mg, 0.90 mmol) and (P)-**6SO$_2$** (162 mg, 0.32 mmol) were added sequentially, and the resulting mixture was stirred at 110 °C for 4 h. The reaction mixture was cooled to room temperature, KHMDS (179 mg, 0.90 mmol) was added, and the resulting mixture was again stirred for 14 h at 110 °C. The reaction was quenched with sat. aqueous NH_4Cl (10 mL) at room temperature, and the resulting biphasic solution was extracted with CH_2Cl_2 (20 mL × 3). The combined organic layer was washed with brine, dried over Na_2SO_4, passed through a pad of silica gel (eluent: CH_2Cl_2), and concentrated under reduced pressure. The crude mixture was purified by column chromatography on silica gel (eluent: CH_2Cl_2: n-hexane = 5:1) to give (P)-**6CX** as a yellow solid (137 mg, 0.19 mmol, 58%, 85%ee). Recrystallization from CH_2Cl_2/ n-hexane afforded the enantiopure product (88.7 mg, >99%ee, $[\alpha]_D^{22}$ = –83.2 (c = 0.10, $CHCl_3$)).

(M)-**6CX** and (rac)-**6CX** were synthesized according to the same procedure.

(M)-**6CX**: Obtained as a yellow solid (138 mg, 0.19 mmol, 59%, 87%ee) from (M)-**6SO$_2$** (162 mg, 0.32 mmol). Recrystallization from CH_2Cl_2/ n-hexane afforded the enantiopure product (66.0 mg, >99%ee, $[\alpha]_D^{24}$ = +67.0 (c = 0.10, $CHCl_3$)).

(*rac*)-**6CX** was obtained as a yellow solid (136 mg, 0.18 mmol, 56%) from (*rac*)-**6SO₂** (162 mg, 0.32 mmol).

^{1}H NMR ($CDCl_3$): δ 7.90 (d, *J* = 8.2 Hz, 2H), 7.86 (d, *J* = 8.2 Hz, 2H), 7.49 (d, *J* = 8.2 Hz, 2H), 7.40 (d, *J* = 8.2 Hz, 2H), 7.38 (dd, *J* = 8.2, 1.6 Hz, 2H), 7.33 (td, *J* = 8.2, 1.6 Hz, 2H), 7.27–7.22 (m, 6H), 7.19 (dd, *J* = 8.2, 1.6 Hz, 2H), 7.14 (td, *J* = 8.2, 1.6 Hz, 2H), 6.99 (t, *J* = 8.2 Hz, 2H), 6.82 (t, *J* = 8.2 Hz, 2H), 6.59 (t, *J* = 8.2 Hz, 2H), 6.40 (td, *J* = 8.2, 1.6 Hz, 2H), 6.11 (dd, *J* = 8.2, 1.6 Hz, 2H).
^{13}C NMR ($CDCl_3$): δ 155.1, 152.6, 152.2, 152.1, 138.7, 137.2, 135.4, 132.9, 130.0, 128.9, 128.5, 127.5, 127.3, 127.0, 125.4, 124.5, 124.2, 123.9, 123.7, 123.5, 123.0, 122.3, 122.2, 117.6, 117.1 (three sp_2-hydbridized carbons were missing probably due to overlapping), 55.1.
HRMS (APCI-MS, positive): *m/z* $[M]^+$ Calcd for $C_{56}H_{32}O_2$: 736.2397; Found: 736.2407.

Synthesis of 6CF: Atom-substitution by fluorene via sequential 4-fold S_NAr reactions

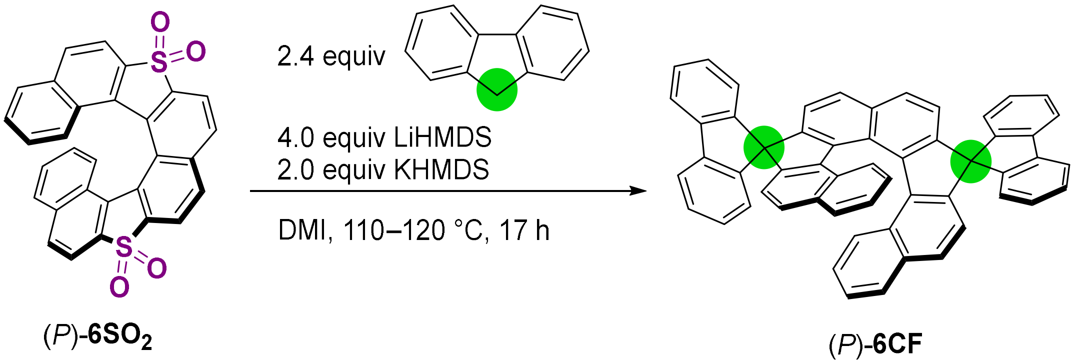

Synthesis of (*P*)-**6CF** is representative. A Schlenk flask was charged with LiHMDS (200 mg, 1.2 mmol) and DMI (3 mL). To the flask, fluorene (122 mg, 0.72 mmol) and (*P*)-**6SO₂** (151 mg, 0.30 mmol) were added sequentially, and the resulting mixture was stirred at 110 °C for 5 h. The reaction mixture was cooled to room temperature, KHMDS (120 mg, 1.2 mmol) was added, and the resulting mixture was again stirred for 10 h at 110 °C and 2 h at 120 °C. The reaction was quenched with sat. aqueous NH_4Cl (3 mL) at room temperature, and the resulting biphasic solution was extracted with CH_2Cl_2 (10 mL × 3). The combined organic layer was washed with brine, dried over Na_2SO_4, passed through a pad of silica gel (eluent: CH_2Cl_2), and concentrated under reduced pressure. The crude mixture purified by column chromatography on silica gel (eluent: CH_2Cl_2: *n*-hexane = 5:1) to (*P*)-**6CF** as a yellow solid (84.2 mg, 0.12 mmol, 40%, 99%ee). Recrystallization from CH_2Cl_2/ *n*-hexane afforded the enantiopure product (46.8 mg, >99%ee, $[\alpha]_D^{24}$ = –29.8 (c =0.099, $CHCl_3$)).

(*M*)-**6CF** and (*rac*)-**6CF** were synthesized according to the same procedure.

(*M*)-**6CF** was obtained as a yellow solid (83.0 mg, 0.12 mmol, 39%, 98%ee) from (*M*)-**6SO₂** (151 mg, 0.30 mmol). Recrystallization from CH_2Cl_2/ *n*-hexane afforded the almost enantiopure product (56.4 mg, >99%ee, $[\alpha]_D^{23}$ = +30.0 (c =0.098, $CHCl_3$)).

(*rac*)-**6CF** was obtained as a yellow solid (21.2 mg, 0.030 mmol, 30%) from (*rac*)-**6SO₂** (50.4 mg, 0.10 mmol).

^{1}H NMR ($CDCl_3$): δ 8.13 (d, *J* = 8.2 Hz, 2H), 7.99 (d, *J* = 7.5 Hz, 2H), 7.92 (d, *J* = 7.5 Hz, 2H), 7.74 (d, *J* = 8.2 Hz, 2H), 7.51 (t, *J* = 6.9 Hz, 2H), 7.38 (t, *J* = 7.5 Hz, 2H), 7.34–7.30 (m, 6H), 7.25–7.24 (m, 2H), 7.03 (t, *J* = 6.9 Hz, 2H), 7.00 (d, *J* = 8.2 Hz, 2H), 6.85 (t, *J* = 7.9 Hz, 2H), 6.80 (d, *J* = 8.2 Hz, 2H), 6.55–6.51 (m, 4H).

^{13}C NMR ($CDCl_3$): δ 148.5, 147.6, 147.3, 145.2, 142.9, 142.5, 141.4, 138.9, 135.4, 133.0, 129.1, 129.0, 128.3, 128.2, 128.0, 126.8, 125.1, 124.8, 124.3, 124.1, 124.0, 123.8, 122.4, 121.3, 120.9, 120.6, 120.2 (one sp_2-hybridized carbon was missing probably due to overlapping), 66.9.

HRMS (APCI-MS, positive): *m/z* $[M]^+$ Calcd for $C_{56}H_{32}$: 704.2499; Found: 704.2483.

3. HPLC trace

Compound 4

Column: DAICEL CHIRALPAK IA (4.6 mm × 250 mm), Eluent: *n*-hexane/*i*PrOH= 1:1, Flow rate: 1.4 mL/min, Detection: 300 nm, Retention time: t_1=8.9 min, t_2=10.7 min.

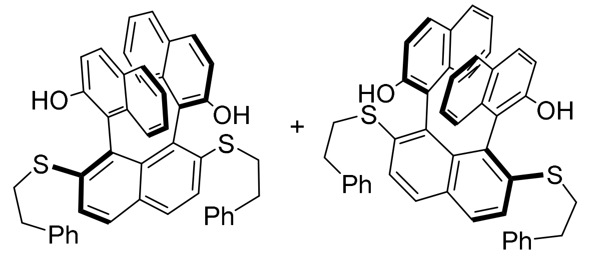

(R_a,R_a)-**4** (S_a,S_a)-**4**

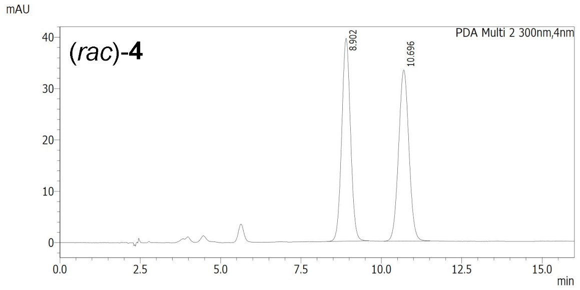

Peak#	Retention time	Area	%
1	8.902	748159	50.169
2	10.696	743131	49.831

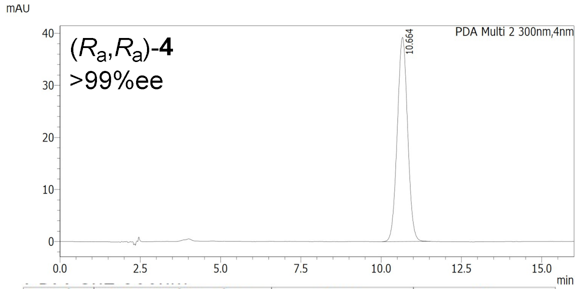

Peak#	Retention time	Area	%
1	10.664	881675	100.000

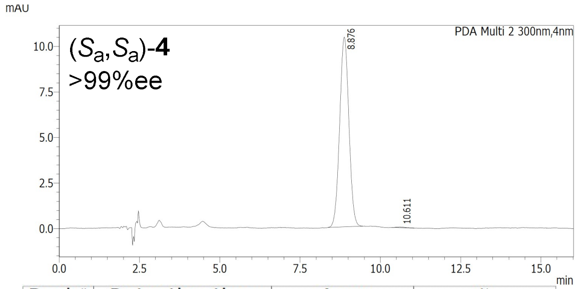

Peak#	Retention time	Area	%
1	8.876	208052	99.661
2	10.611	707	0.339

Fig. S1 HPLC chromatograms of **4**.

Compound 6O

Column: DAICEL CHIRALPAK IA (4.6 mm × 250 mm), Eluent: *n*-hexane/*i*PrOH= 100:1, Flow rate: 1.5 ml/min, Detection: 350 nm, Retention time: t_1=4.1 min, t_2=5.1 min.

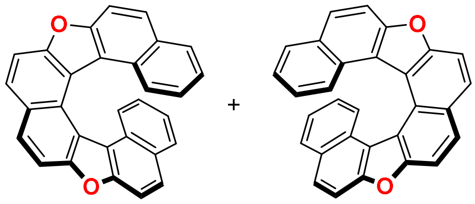

(*M*)-**6O** from (*R*,*R*)-**5**

(*P*)-**6O** from (*S*,*S*)-**5**

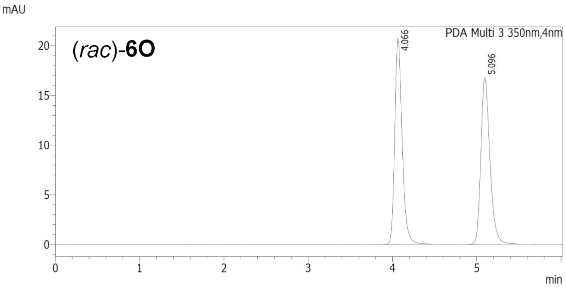

Peak#	Retention time	Area	%
1	4.066	125812	50.086
2	5.096	125381	49.914

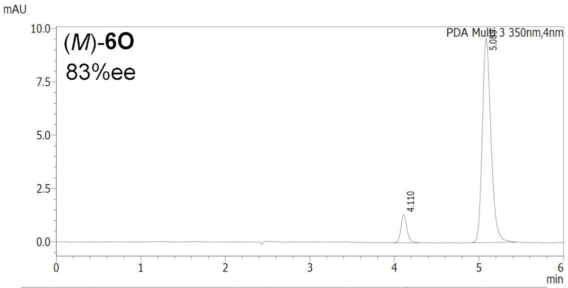

Peak#	Retention time	Area	%
1	4.110	6491	8.658
2	5.085	68483	91.342

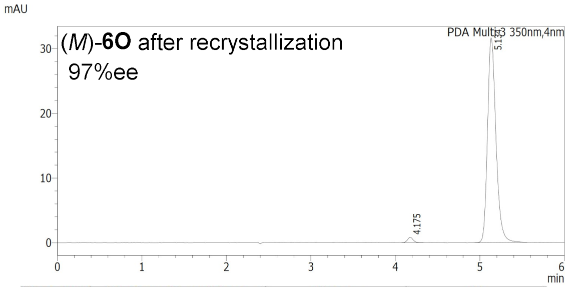

Peak#	Retention time	Area	%
1	4.175	3715	1.660
2	5.134	219998	98.340

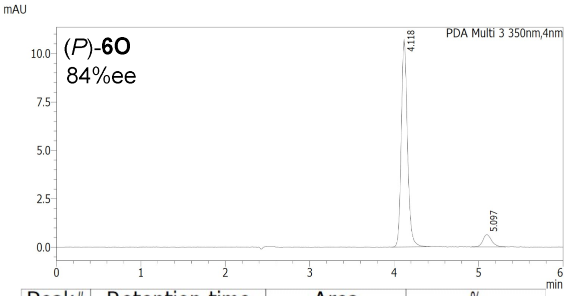

Peak#	Retention time	Area	%
1	4.118	54326	92.146
2	5.097	4631	7.854

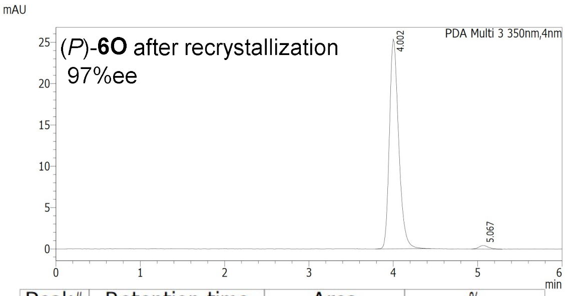

Peak#	Retention time	Area	%
1	4.002	194567	98.266
2	5.067	3434	1.734

Fig. S2 HPLC chromatograms of **6O**.

Compound 6S

Column: DAICEL CHIRALPAK IA (4.6 mm × 250 mm), Eluent: *n*-hexane/*i*PrOH= 100:5, Flow rate: 1.0 ml/min, Detection: 300 nm, Retention time: t_1=4.6 min, t_2=5.1 min.

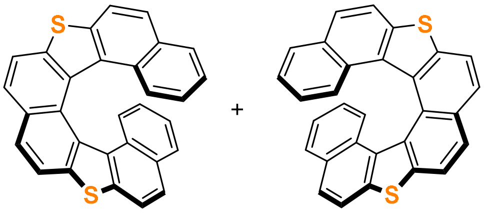

(*M*)-**6S** from (*R*,*R*)-**4**

(*P*)-**6S** from (*S*,*S*)-**4**

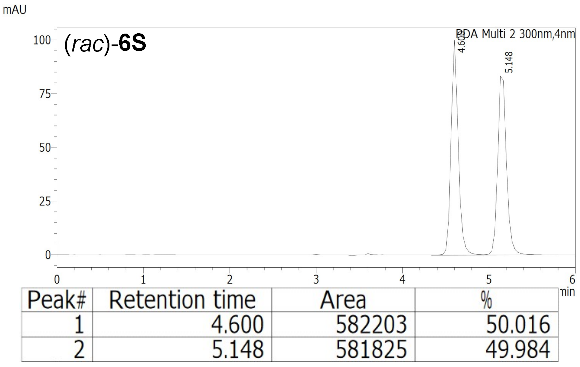

Peak#	Retention time	Area	%
1	4.600	582203	50.016
2	5.148	581825	49.984

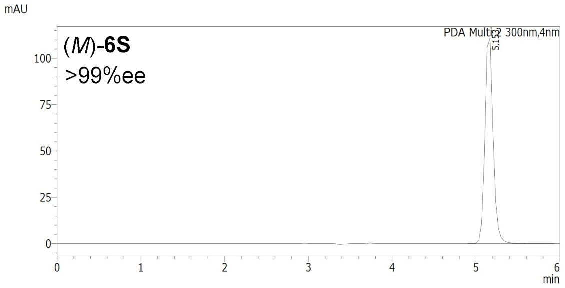

Peak#	Retention time	Area	%
1	5.153	753808	100.000

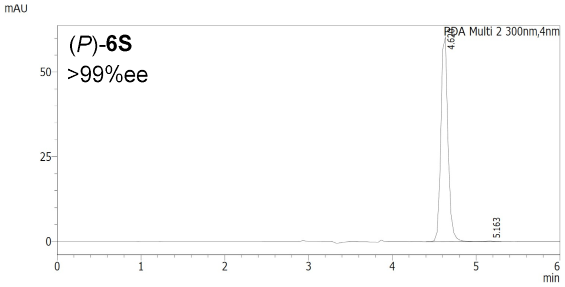

Peak#	Retention time	Area	%
1	4.620	358658	99.683
2	5.163	1142	0.317

Fig. S3 HPLC chromatograms of **6S**.

Compound $6SO_2$

Column: DAICEL CHIRALPAK IA (4.6 mm × 250 mm), Eluent: *n*-hexane/*i*PrOH = 1:1, Flow rate: 1.0 mL/min, Detection: 300 nm, Retention time: t_1=8.3 min, t_2=9.5 min.

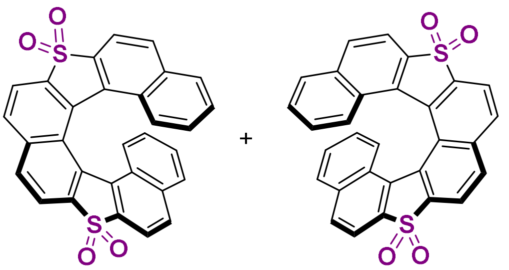

(*M*)-**$6SO_2$** from (*M*)-**6S** + (*P*)-**$6SO_2$** from (*P*)-**6S**

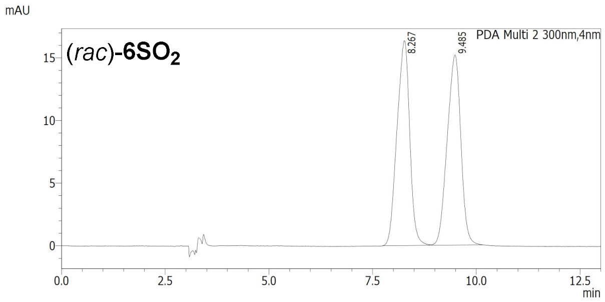

Peak#	Retention time	Area	%
1	8.267	349181	50.078
2	9.485	348091	49.922

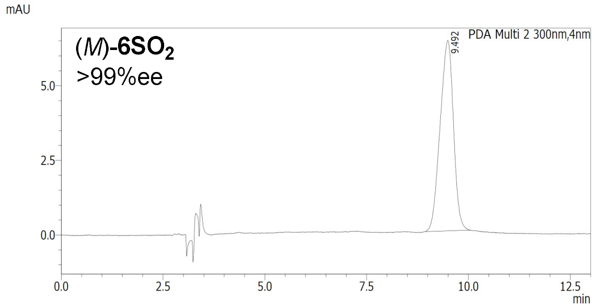

Peak#	Retention time	Area	%
1	9.492	145734	100.000

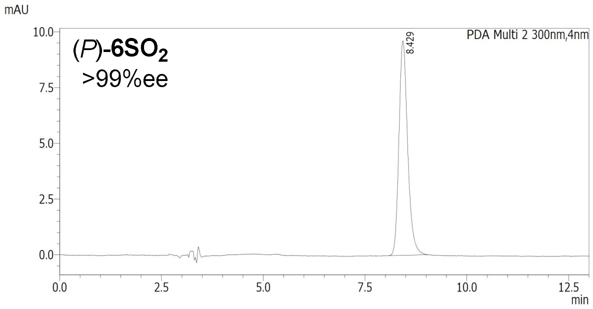

Peak#	Retention time	Area	%
1	8.429	143656	100.000

Fig. S4 HPLC chromatograms of **$6SO_2$**.

Compound 6N

Column: DAICEL CHIRALPAK IC-3 (4.6 mm × 150 mm), Eluent: *n*-hexane:*i*PrOH = 85:15, Flow rate: 1.0 mL/min, Detection: 300 nm, Retention time: t_1=6.5 min, t_2=11.9 min.

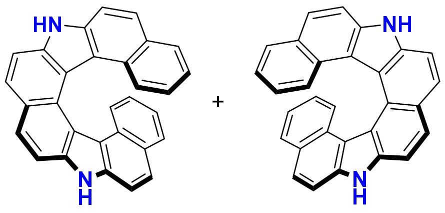

(*M*)-**6N**
from (*M*)-**6SO$_2$**

(*P*)-**6N**
from (*P*)-**6SO$_2$**

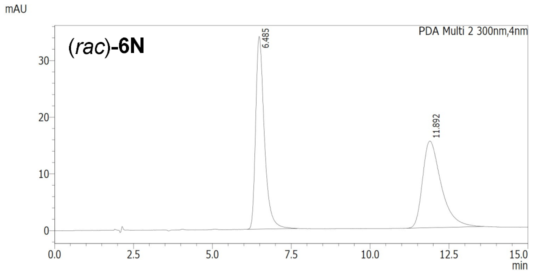

Peak#	Retention time	Area	%
1	6.485	651623	50.893
2	11.892	628753	49.107

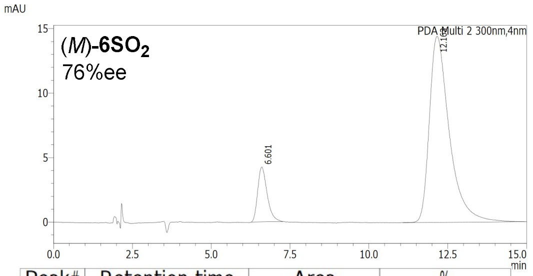

Peak#	Retention time	Area	%
1	6.601	88510	12.204
2	12.163	636740	87.796

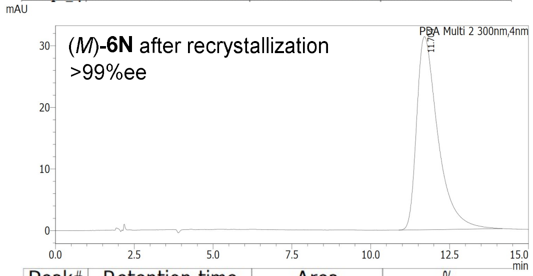

Peak#	Retention time	Area	%
1	11.704	1477489	100.000

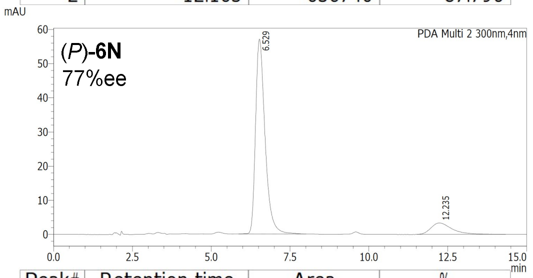

Peak#	Retention time	Area	%
1	6.529	1197371	88.327
2	12.235	158233	11.673

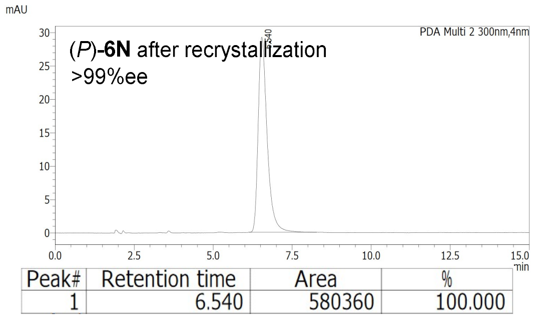

Peak#	Retention time	Area	%
1	6.540	580360	100.000

Fig. S5 HPLC chromatograms of **6N**.

Compound 6CX

Column: DAICEL CHIRALPAK AD-H (4.6 mm ×250 mm), Eluent: *n*-hexane:*i*PrOH = 100:3,
Flow rate: 1.0 mL/min, Detection: 300 nm, Retention time: t_1=3.9 min, t_2=4.2 min.

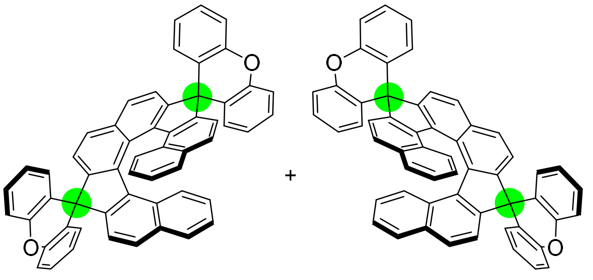

(*M*)-**6CX** from (*M*)-**6SO$_2$**

(*P*)-**6CX** from (*P*)-**6SO$_2$**

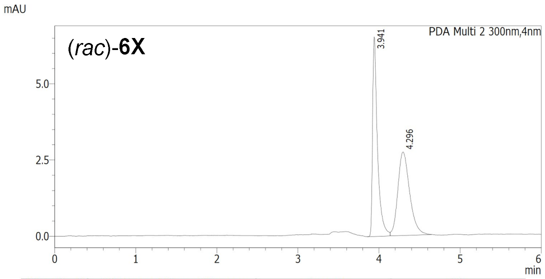

Peak#	Retention time	Area	%
1	3.941	27426	49.737
2	4.296	27715	50.263

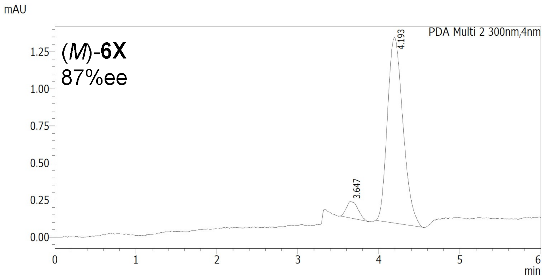

Peak#	Retention time	Area	%
1	3.647	1127	6.299
2	4.193	16761	93.701

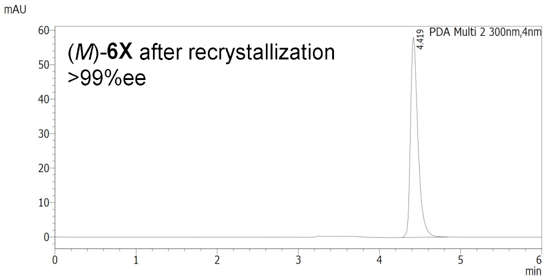

Peak#	Retention time	Area	%
1	4.419	372164	100.000

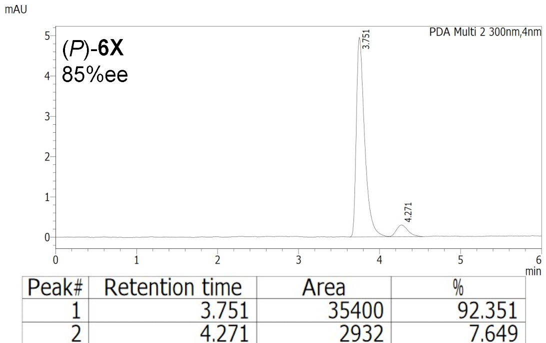

Peak#	Retention time	Area	%
1	3.751	35400	92.351
2	4.271	2932	7.649

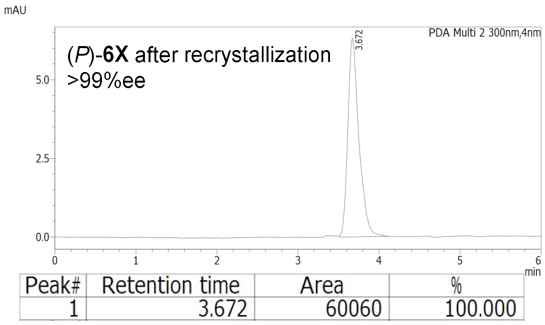

Peak#	Retention time	Area	%
1	3.672	60060	100.000

Fig. S6 HPLC chromatograms of **6CX**.

Compound 6CF

Column: DAICEL CHIRALPAK AD-H (4.6 mm × 250 mm), Eluent: *n*-hexane:*i*PrOH = 100:3, Flow rate: 1.0 mL/min, Detection: 300 nm, Retention time: t_1=4.2 min, t_2=5.0 min.

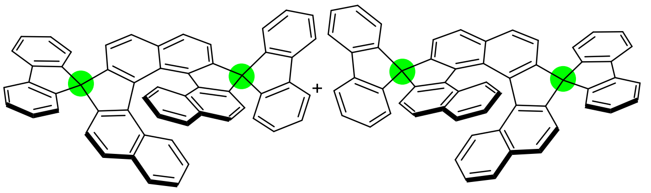

(*M*)-**6CF** from (*M*)-**6SO$_2$**

(*P*)-**6CF** from (*P*)-**6SO$_2$**

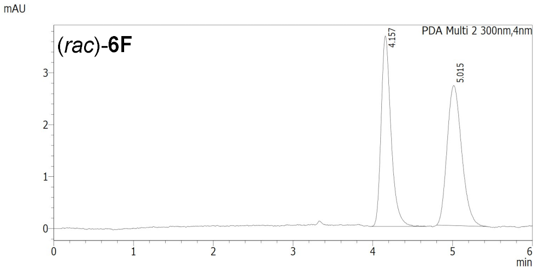

Peak#	Retention time	Area	%
1	4.157	31596	47.813
2	5.015	34485	52.187

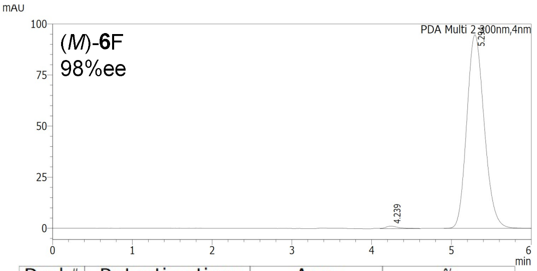

Peak#	Retention time	Area	%
1	4.239	13445	0.923
2	5.294	1443130	99.077

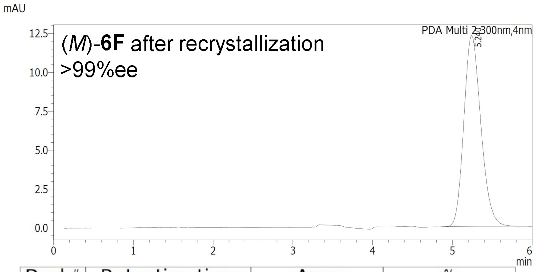

Peak#	Retention time	Area	%
1	5.240	181704	100.000

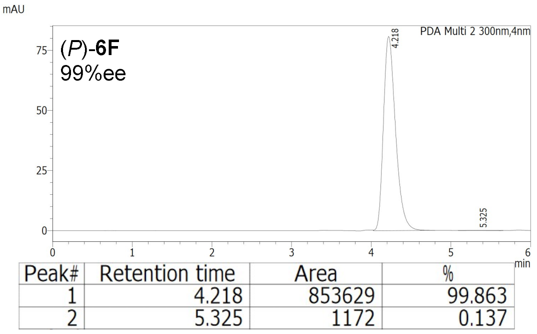

Peak#	Retention time	Area	%
1	4.218	853629	99.863
2	5.325	1172	0.137

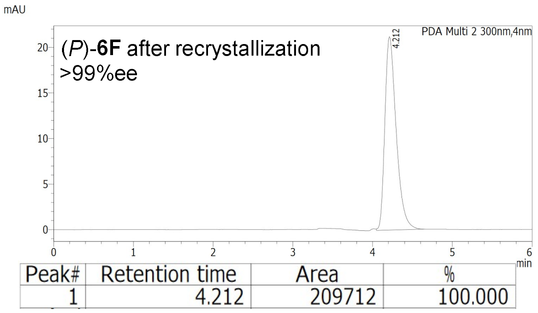

Peak#	Retention time	Area	%
1	4.212	209712	100.000

Fig. S7 HPLC chromatograms of **6CF**.

4. X-Ray Crystallographic Details

All thermal ellipsoids were scaled to 50% probability.

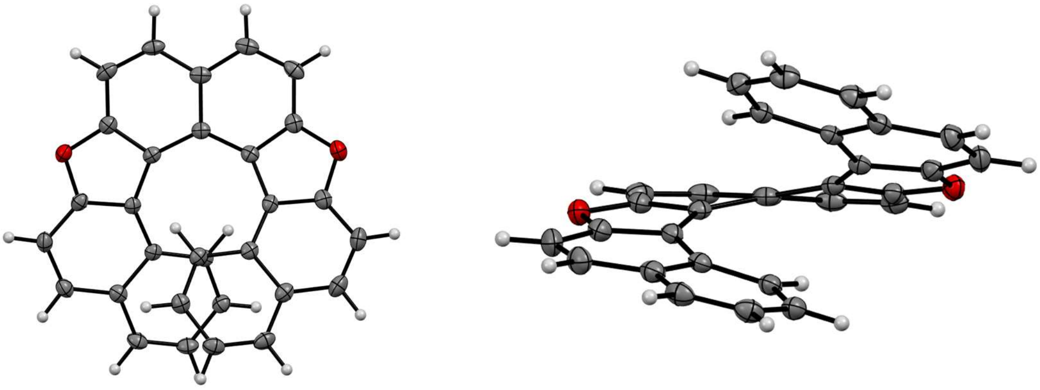

Fig. S8 ORTEP representation of the crystal structure of (*rac*)-**6O**.

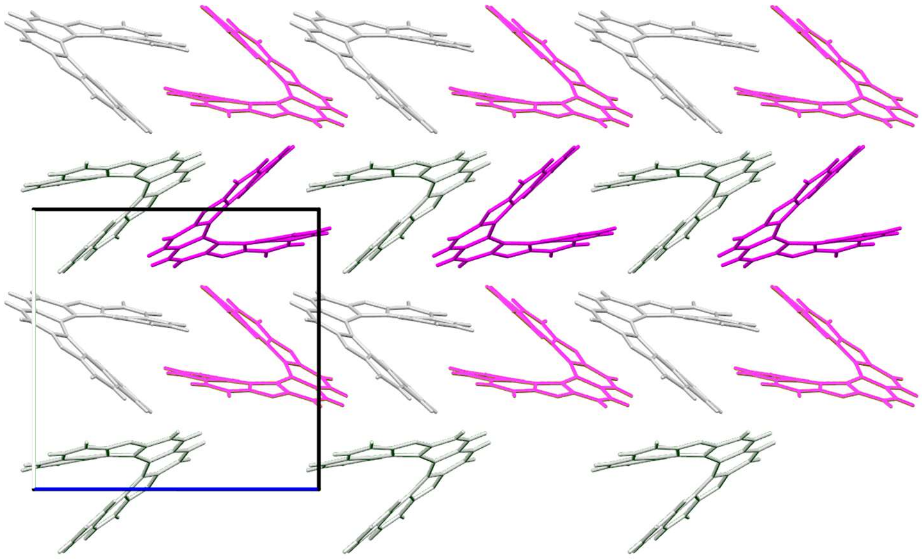

Fig. S9 Packing structure of (*rac*)-**6O**.

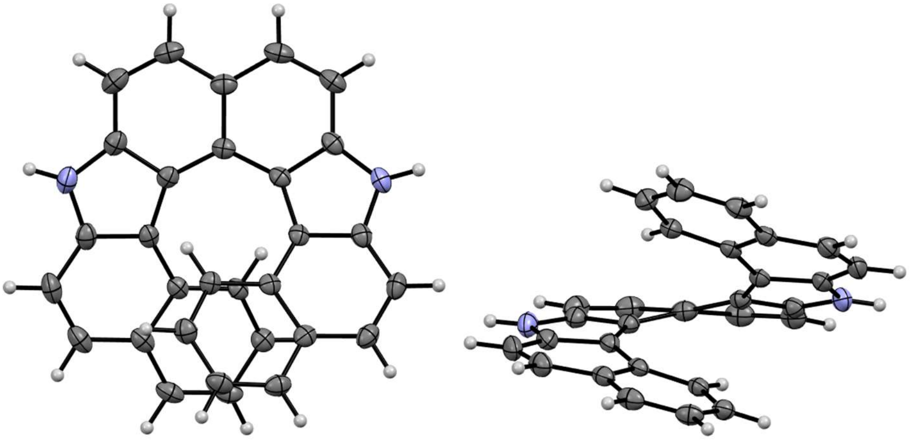

Fig. S10 ORTEP representation of the crystal structure of (*rac*)-**6N**.

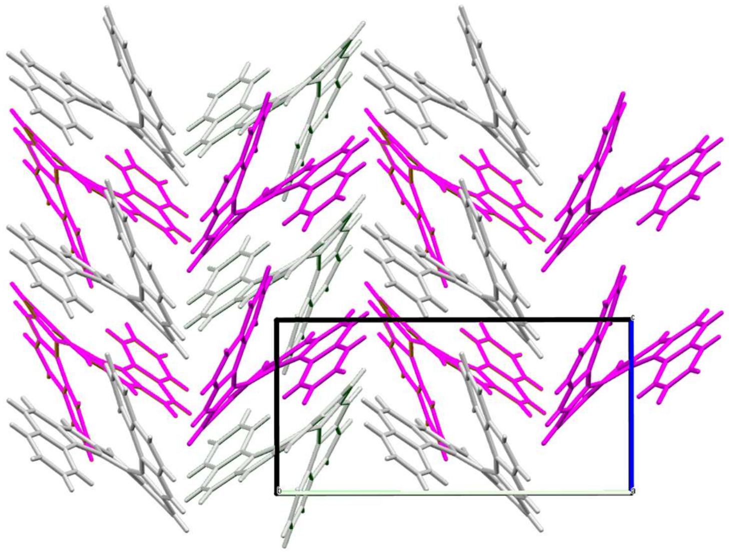

Fig. S11 Packing structure of (*rac*)-**6N**.

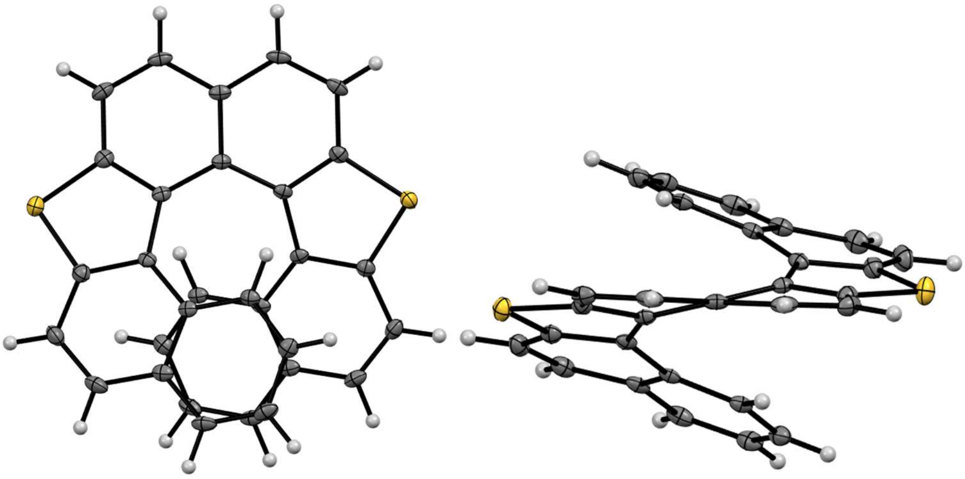

Fig. S12 ORTEP representation of the crystal structure of (*rac*)-**6S**.

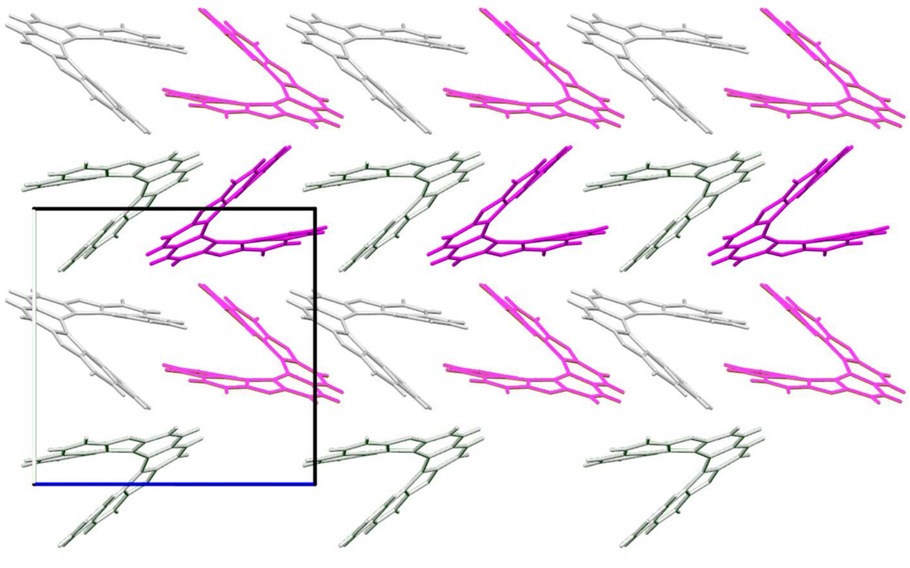

Fig. S13 Packing structure of (*rac*)-**6S**.

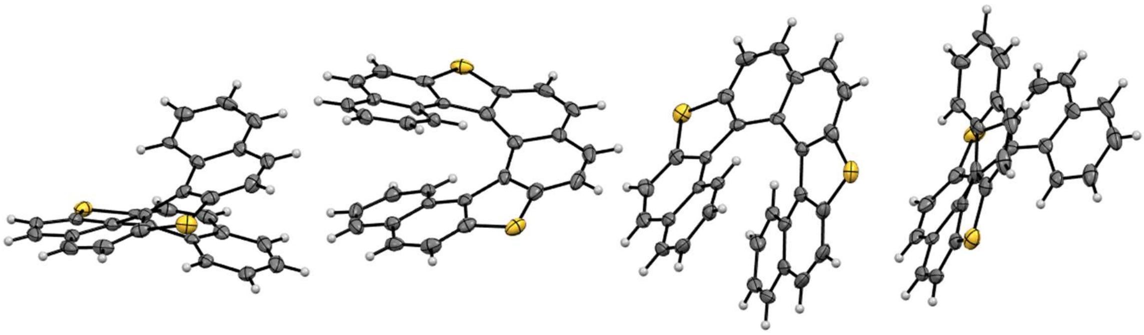

Fig. S14 ORTEP representation of the crystal structure of (*P*)-**6S** derived from 2nd fraction of **4'**. The unit cell contained four independent molecules.

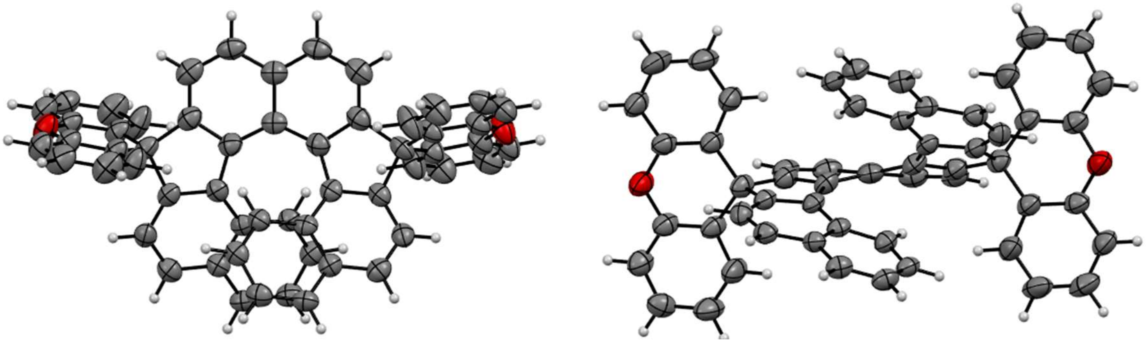

Fig. S15 ORTEP representation of the crystal structure of **6CX** obtained from a solution of (*rac*)-**6CX** in $CHCl_3$/ *n*-hexane. Due to spontaneous resolution, the crystal consisted of only one isomer, but the absolute configuration was not determined. Solvent molecules were omitted for clarity.

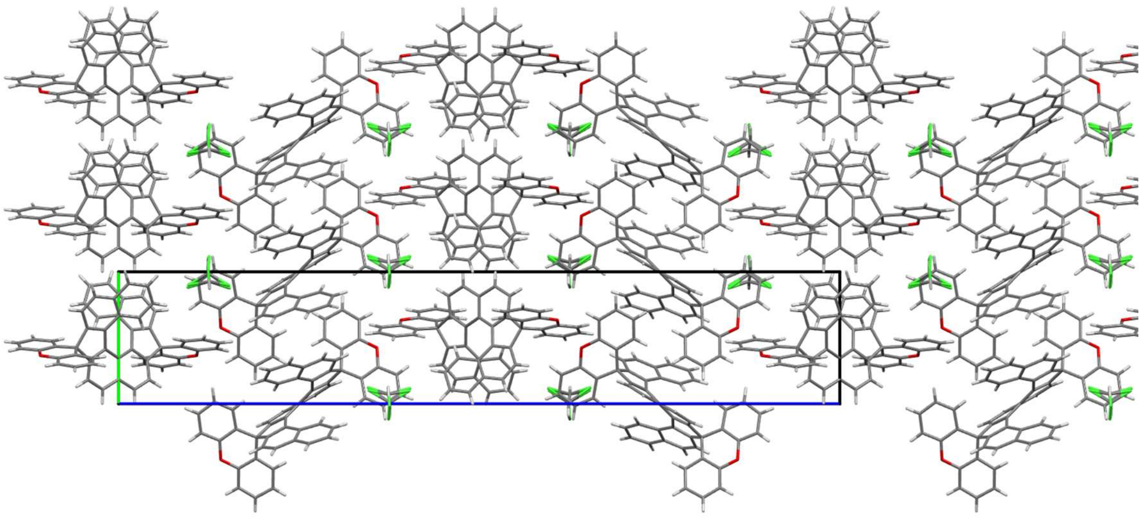

Fig. S16 Packing structure of **6CX**.

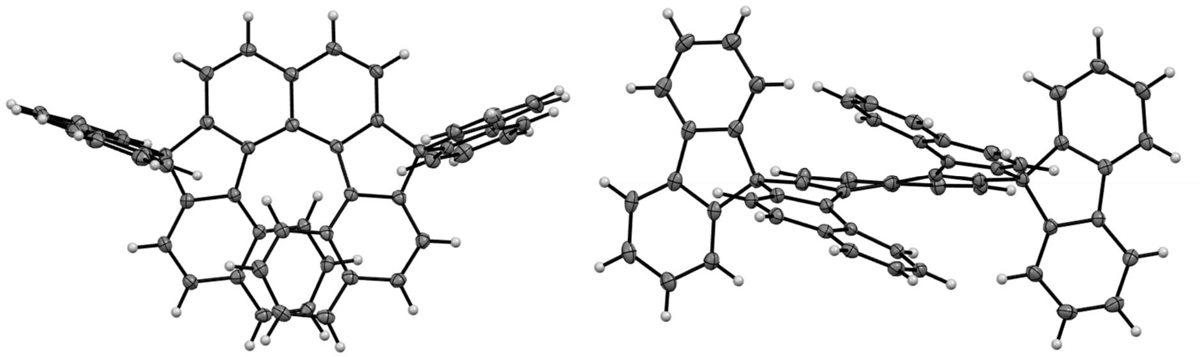

Fig. S17 ORTEP representation of the crystal structure of (*M*)-**6CF** obtained from a solution of (*rac*)-**6CF** in CH_2Cl_2/ *n*-hexane. Due to spontaneous resolution, the crystal consisted of only (*M*)-isomer. Solvent molecules were omitted for clarity.

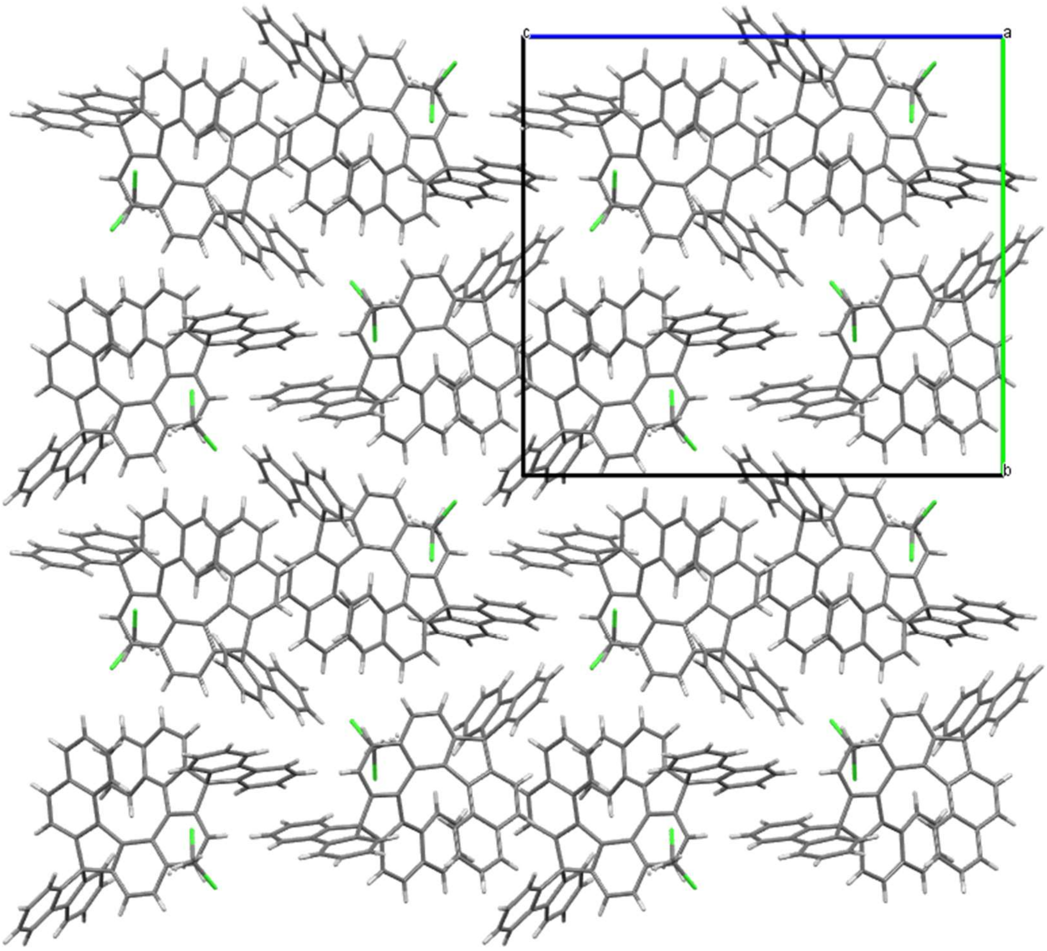

Fig. S18 Packing structure of (*M*)**-6CF**.

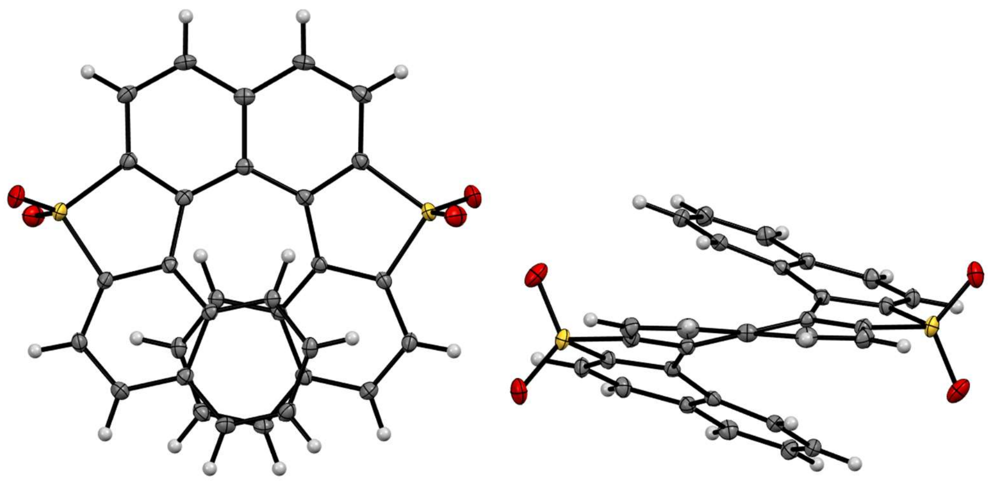

Fig. S19 ORTEP representation of the crystal structure of (*rac*)-**6SO₂**.

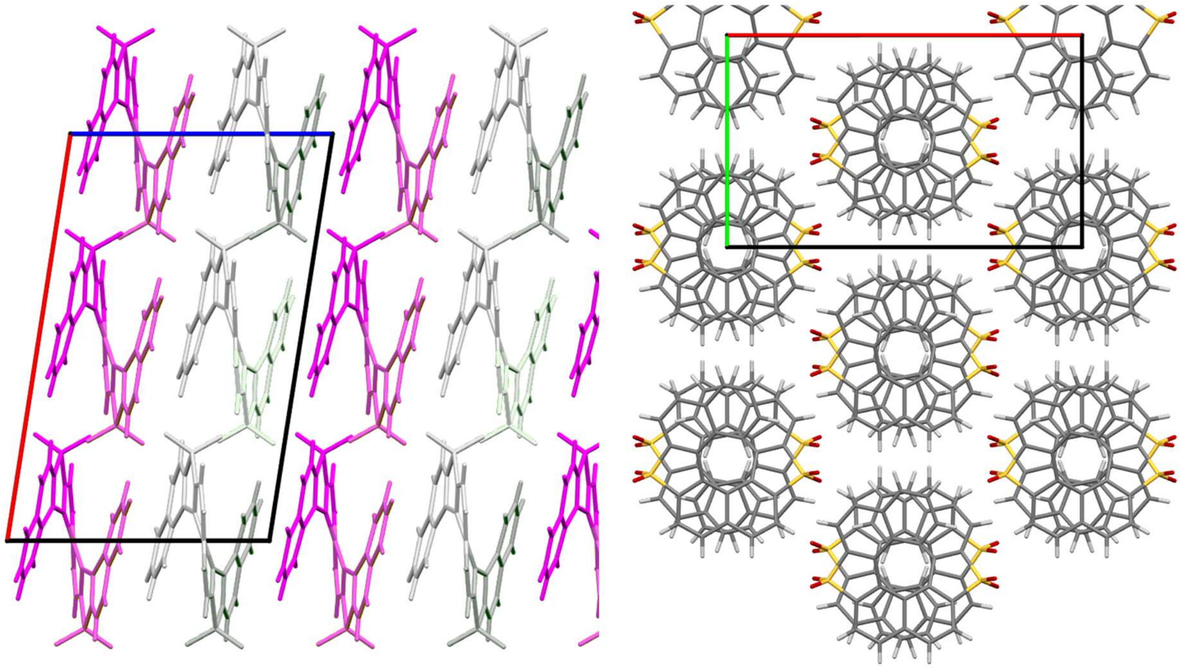

Fig. S20 Packing structure of (*rac*)-**6SO₂**.

Table S1 Crystal parameters and structural refinement data.

Compound	(*rac*)-**6O**	(*rac*)-**6S**	(*rac*)-**6N**
Empirical Formula	$C_{30}H_{16}O_2$	$C_{30}H_{16}S_2$	$C_{30}H_{18}N_2$
Mw	408.46	440.55	406.46
Crystal System	Monoclinic	Monoclinic	Monoclinic
Space Group	$P2_{1/c}$ (No.14)	$P2_{1/n}$ (No.11)	$P2_1/c$ (No.14)
a [Å]	8.1656(12)	9.338(3)	15.9958(1)
b [Å]	15.1358(14)	13.622(3)	15.9492(1)
c [Å]	15.7979(16)	16.299(4)	7.8148(1)
α [deg]	90	90	90
β [deg]	102.46682(19)	102.204(7)	91.516(1)
γ [deg]	90	90	90
Volume / $Å^3$	1906.5(4)	2026.4(9)	1993.02(3)
Z	4	4	4
Density [$g\cdot cm^{-3}$]	1.423	1.444	1.355
Flack parameter	--	--	--
Completeness	0.969	0.994	0.989
Goodness-of-fit	1.046	1.071	1.049
R_1 [$I > 2\sigma(I)$]	0.0313	0.0311	0.0380
wR_2 (all data)	0.0844	0.0797	0.0948
Solvent system	$CHCl_3$	CH_2Cl_2/*n*-hexane	CH_2Cl_2/acetone / *n*-hexane
CCDC number	1504747	2047440	2047442

Compound	(*M*)-**6CX**[a,b]	(*M*)-**6CF**[a]	(*rac*)-**6SO₂**
Empirical Formula	$0.5(C_{56}H_{32}O_2)\cdot 0.5(CHCl_3)$	$(C_{56}H_{32})\cdot(CH_2Cl_2)$	$0.5(C_{30}H_{16}O_4S_2)$
Mw	428.09	789.74	252.27
Crystal System	Tetragonal	Orthorhombic	Monoclinic
Space Group	$P4_1$ 2 2 (No.91)	$P2_1$ 2_1 2_1 (No.19)	*C*2/*c* (No.15)
a [Å]	9.0201(9)	8.57192(4)	17.7976(2)
b [Å]	9.0201(9)	20.53361(10)	10.4721(1)
c [Å]	49.382(7)	22.43166(11)	11.7388(1)
α [deg]	90	90	90
β [deg]	90	90	99.158(1)
γ [deg]	90	90	90
Volume / $Å^3$	4017.8(10)	3948.25(3)	2159.97(4)
Z	8	4	8
Density [$g\cdot cm^{-3}$]	1.415	1.329	1.552
Flack parameter	0.478(5)	0.001(4)	--
Completeness	1.61/1.00	1.77/0.99	0.989
Goodness-of-fit	1.002	1.030	1.093
R_1 [$I > 2\sigma(I)$]	0.0853	0.0366	0.0360
wR_2 (all data)	0.2449	0.1004	0.0975
Solvent system	$CHCl_3$/ *n*-hexane	CH_2Cl_2/ *n*-hexane	CH_2Cl_2/EtOAc
CCDC number	2047444	2047445	2047446

[a] The crystal was obtained from a solution of racemate by spontaneous resolution. [b] The accuracy of the absolute configuration is low.

Compound	(*P*)-**6S**
Empirical Formula	$4(C_{30}H_{16}S_2)$
Mw	1762.19
Crystal System	Monoclinic
Space Group	$P2_1$ (No.4)
a [Å]	17.272(2)
b [Å]	14.9852(13)
c [Å]	17.311(2)
α [deg]	90
β [deg]	114.511(3)
γ [deg]	90
Volume / $Å^3$	4076.7(8)
Z	2
Density [$g \cdot cm^{-3}$]	1.436
Flack parameter	0.050(3)
Completeness	1.89/0.99
Goodness-of-fit	1.083
R_1 [$I > 2\sigma(I)$]	0.0505
wR_2 (all data)	0.1379
Solvent system	CH_2Cl_2/*n*-hexane
CCDC number	2047441

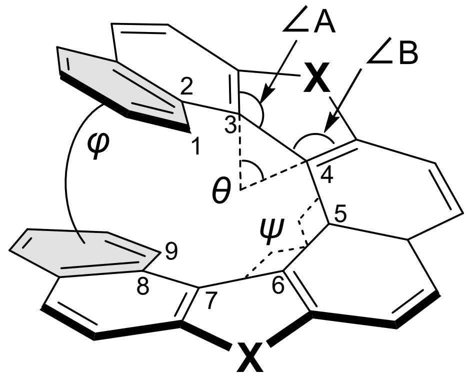

$$\theta = 180-(180-\angle A)-(180-\angle B) = \angle A+\angle B-180$$

Fig. S21. Definition of structural parameters.

Table S2 Summary of structural features of helicenes based on X-ray crystal structures.

Compound	Bond length /Å (Inner helix)	Wedge angle	Torsional angle	Interplanar angle
---	C1–C2, C2–C3, ·· C7–C8, C8–C9	θ	ψ: ∠C1-C2-C3-C4, ··· , ∠C6-C7-C8-C9	φ
(*rac*)-**6O**	1.41, 1.44, 1.46, 1.44, 1.44, 1.47, 1.44, 1.41	30°	4.1°, 12.7°, 20.4°, 18.5, 9.1°, 9.0° (73.8° total)	34.0°
(*rac*)-**6N**	1.42, 1.44, 1.45, 1.44, 1.43, 1.45, 1.43, 1.42	32°	7.3°, 14.0°, 23.1°, 18.2°, 12.9°, 9.5° (85.0° total)	38.5°
(*rac*)-**6S**	1.41, 1.44, 1.45, 1.44, 1.44, 1.46, 1.44, 1.41	42°	11.9°, 17.8°, 20.7°, 18.0°, 16.1°, 15.4° (99.9° total)	20.6°
(*M*)-**6CX**	1.41, 1.42, 1.48, 1.43, 1.43, 1.48, 1.42, 1.41	36°	4.8°, 15.3°, 25.0°, 25.0°, 15.3°, 4.8° (90.2° total)	51.2°
(*M*)-**6CF**	1.42, 1.43, 1.49, 1.44, 1.44, 1.48, 1.43, 1.42	36°	14.9°, 16.7°, 14.5°, 19.3°, 18.3°, 11.7° (95.5° total)	21.0°
(*rac*)-**6SO₂**	1.42, 1.43, 1.49, 1.43, 1.43, 1.49, 1.43, 1.42	44°	11.6°, 18.8°, 19.4°, 19.4°, 18.8°, 11.6° (99.6° total)	21.1°

Table S3 Summary of Structural features of helicenes based on DFT calculation.

Compound	Bond length /Å (Inner helix)	Wedge angle	Torsional angle	Interplanar angle
**6O**	1.41, 1.43, 1.46, 1.43, 1.43, 1.46, 1.43, 1.41	30°	7.2°, 12.5°, 20.0°, 20.0°, 12.5°, 7.3° (79.5° total)	35.4°
**6N**	1.41, 1.43, 1.45, 1.43, 1.443, 1.45, 1.43, 1.41	33°	9.6°, 15.7°, 18.5°, 18.5°, 15.7°, 9.6° (87.6° total)	29.5°
**6S**	1.41, 1.44, 1.46, 1.44, 1.44, 1.46, 1.44, 1.41.	42°	14.6°, 17.7°, 18.2°, 18.2°, 17.7°, 14.6° (101.0° total)	15.8°
**6CF**	1.42, 1.43, 1.49, 1.44, 1.44, 1.49, 1.43, 1.42	36°	12.4°, 17.3°, 17.9°, 17.9°, 17.2°, 12.4° (95.1° total)	19.7°
**6SO$_2$**	1.42, 1.43, 1.49, 1.43, 1.43, 1.49, 1.43, 1.42	45°	12.3°, 19.3°, 19.3°, 19.3°, 19.3°, 12.3° (101.8° total)	18.1°
carbo[8]helicene	1.42, 1.45, 1.44, 1.45, 1.45, 1.44, 1.45, 1.42	56°	19.1°, 27.2°, 22.9°, 22.9°, 27.2°, 19.2 °(138.5° total)	7.1°

All geometries were optimized and characterized at the B3LYP-D3(BJ)/6-311G(d,p) level in the gas phase.

5. Racemization Experiments

A 20-mL Schlenk tube was charged with an enantio-enriched helicene (1.5 mg) and anhydrous tetraglyme (4.0 mL). The solution was heated at the indicated temperature under a nitrogen atmosphere, and the time-course of enantiomeric ratio was monitored by HPLC.

(*M*)-**6O**, **6S**, **6CF**, and (*P*)-**6N** were used for the experiments. Unfortunately, **$6SO_2$** was unstable under heating conditions and completely decomposed to form a complex mixture within 10 min at 270 °C.

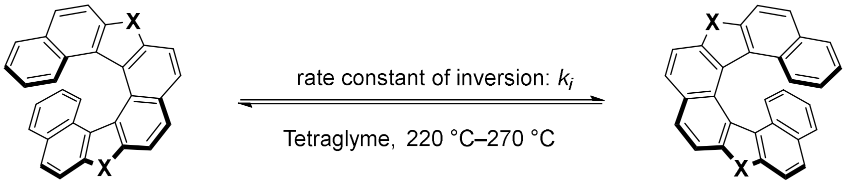

(*P*)-**6** (*M*)-**6**

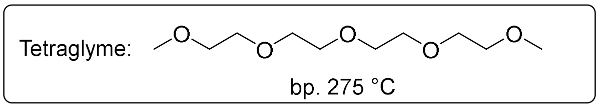

We determined the thermodynamic parameters of the racemization of the series of helicenes. Since the racemization is a reversible unimolecular process, the differential equation for the racemization is given below:

$$\frac{d[M]}{dt} = -k_i[M] + k_i\,[P] \qquad (1)$$

$$[P] + [M] = 100 \qquad (2)$$

$[M]$: the ratio of (*P*)-**6**, $[P]$: the ratio of (*M*)-**6**

Rearrange to give:

$$\frac{d[M]}{dt} = -k_i[M] + k_i\,(100 - [M]) = -k_i(2[M] - 100) \qquad (3)$$

Integral representation of eq. 3 is given below:

$$[M] = 50 + ([M_0] - 50) \times e^{-2k_i t} \quad (4)$$

$[M_0]$: the initial ratio of the (*M*)-isomer

According to eq. 2 and eq. 4, the relationship between [*P*] and time is given below:

$$[P] = 50 - ([M_0] - 50) \times e^{-2k_i t} \quad (5)$$

According to eq. 4 and eq. 5, ee can be represented as a function of time given below:

$$ee(t) = [M] - [P]$$
$$= 2 \times ([M_0] - 50) \times e^{-2k_i t}$$
$$= ee_0 \times e^{-2k_i t} \quad (ee_0\text{: the initial ee}) \quad (5)$$

Experimental values of $[M_0]$ and k_i were obtained by curve fitting of the time-courses of ee to eq. 5 using SciPy (ver. 1.5.2).

According to eq 5., the rate constant of racemization can be given as $k_r = 2k_i$.

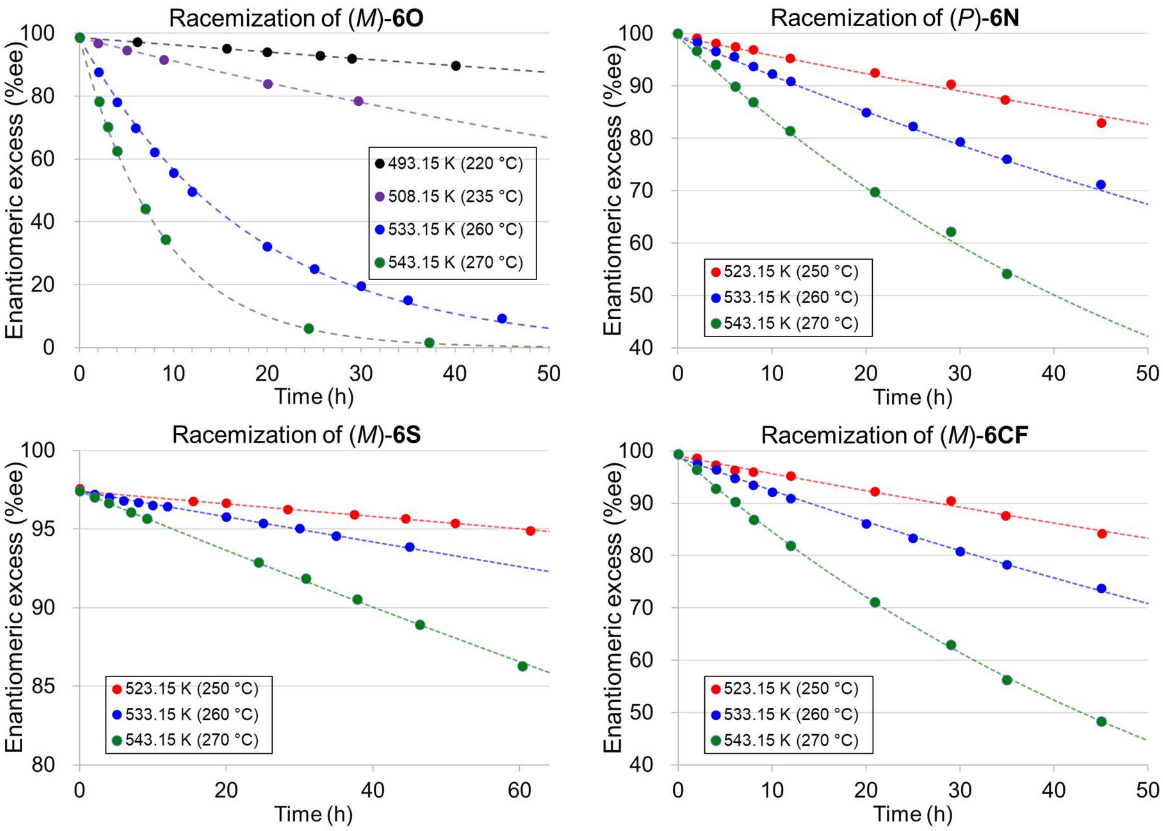

Fig. S22 Time-courses of ee over the reaction time at various temperatures.

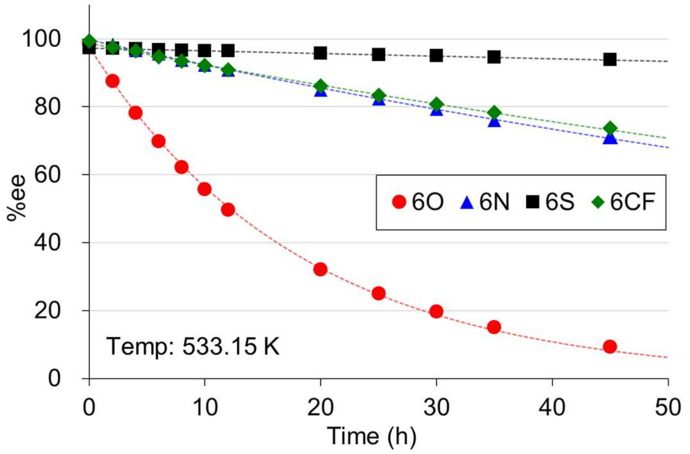

Fig. S23 Time-courses of ee of a series of helicenes over the reaction time at 533.15 K.

Table S4 Rate constants of racemization of a series of helicenes at various temperatures.

compound	rate constant of racemization: k_r (10^{-7}/s)				
	493 K / 220 °C	508 K / 235 °C	523 K / 250 °C	533 K / 260 °C	543 K / 270 °C
(*M*)-**6O**	6.58	21.5	–	153	318
(*P*)-**6N**	–	–	10.9	21.2	48.1
(*M*)-**6S**	–	–	1.17	2.27	5.50
(*M*)-**6CF**	–	–	9.65	18.5	44.5

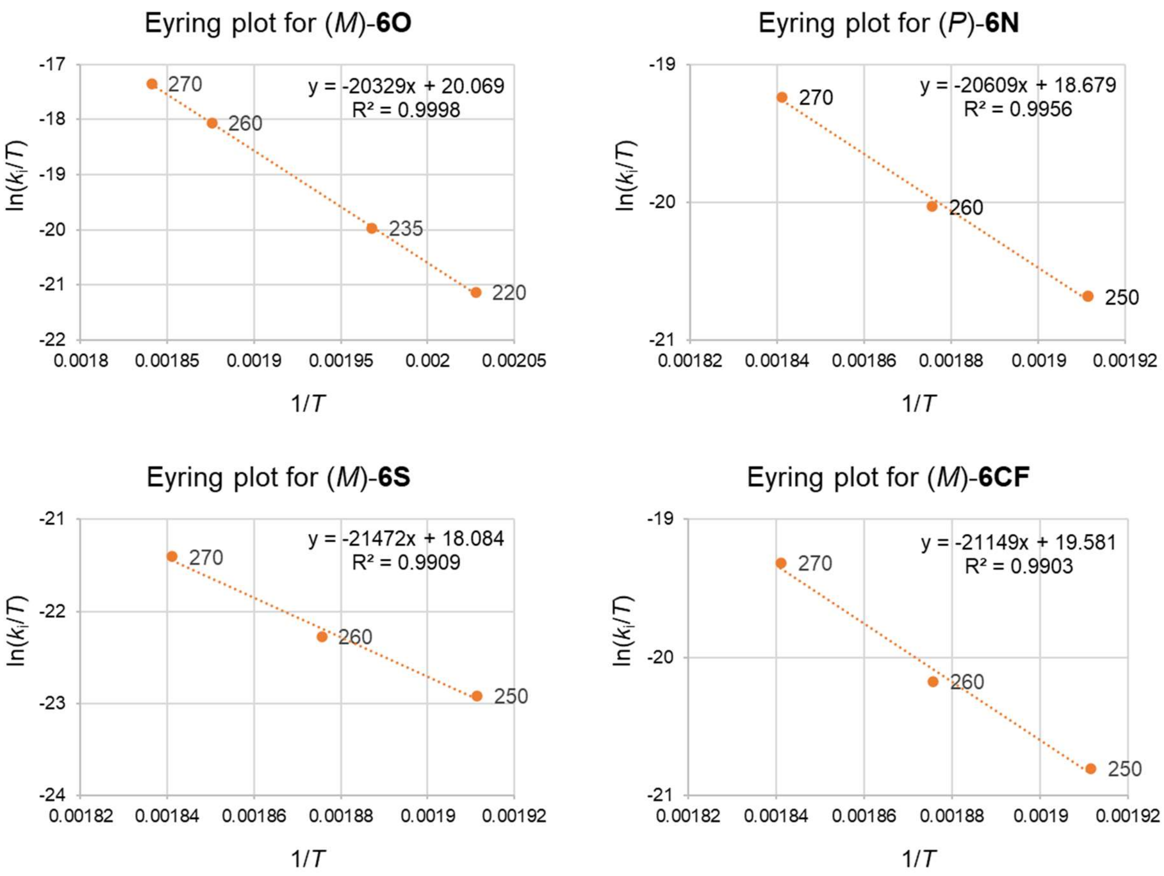

Fig. S24 Eyring plot.

Table S5 Thermodynamic parameters of racemization of a series of helicenes obtained by Eyring plots and DFT calculations.

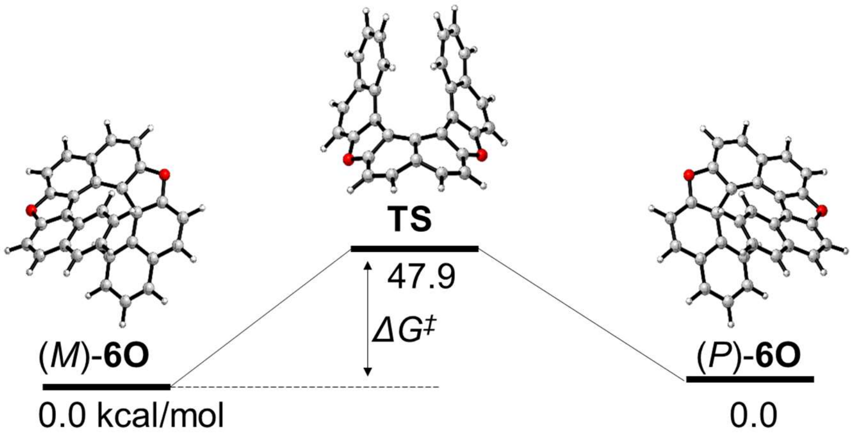

compound	$\Delta H^{\ddagger}$ (kcal/mol) exp (calc.)	$\Delta S^{\ddagger}$ (cal/mol·K) exp (calc.)	$\Delta G^{\ddagger}$ (kcal/mol) exp (calc.)
**6O**	40.4 (44.9)	–7.3 (–5.7)	44.3 (47.9)
**6N**	41.0 (47.1)	–11.4 (–5.2)	46.3 (49.8)
**6S**	42.7 (49.9)	–12.7 (–3.5)	48.7 (51.7)
**6CF**	42.0 (46.6)	–9.7 (0.38)	46.2 (46.4)
**6SO$_2$**	--(46.1)	-- (–5.9)	--(49.3)
carbo[8]helicene	41.0 (43.9)	–6.1(–3.9)	43.5 (45.8)[a]

All geometries were optimized and characterized at the B3LYP-D3(BJ)/6-311G(d,p) level in the gas phase (533.15 K, 1 atm). [a] The experimental values were reported in ref. 8. Racemization is a multi-step process. The shown value is the activation free energy from the minimum structure to the highest TS (See Fig. S25).

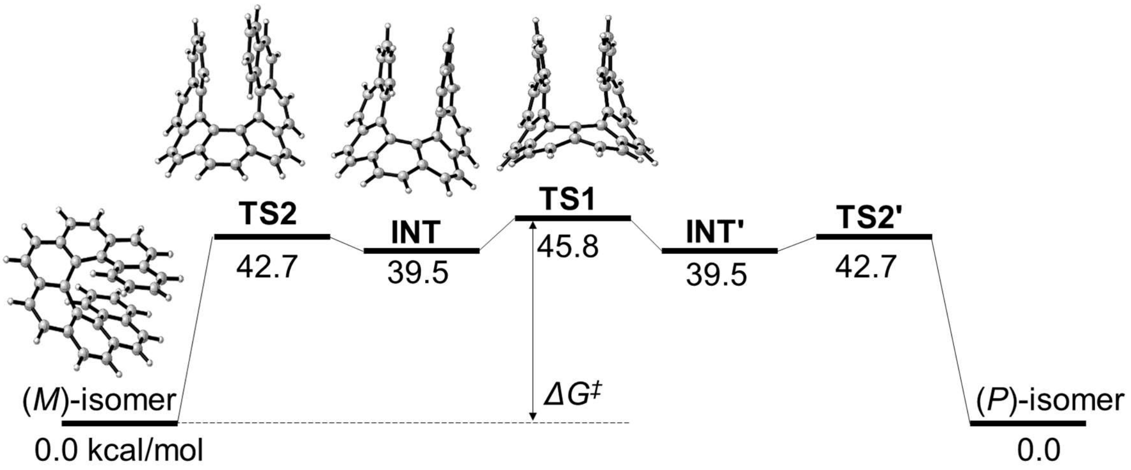

Fig. S25 Energy diagram of racemization of carbo[8]helicene. The pathway from **TS1** to (*P*)-isomer is omitted because it is the mirror-symmetric with the shown pathway.

Table S6 Dependence of the activation free energy of racemization on DFT functionals.

DFT functional	$\Delta G^{\ddagger}$ (kcal/mol)		$\Delta\Delta G^{\ddagger}$ (kcal/mol)
	**6O**	carbo[8]helicene	
experimental values	44.3	43.5	0.8
B3LYP	45.1	42.3	2.8
B3LYP-D3	47.4	45.0	2.4
B3LYP-D3(BJ)	47.9	45.8	2.1
B3LYP-D3(BJ)[a]	47.5	45.6	2.0
PBE0	45.6	43.4	2.2
PBE0-D3	47.2	45.5	1.7
M06-2X	47.5	46.6	0.9
ωB97X-D	48.6	46.1	2.4

Geometry optimization and frequency calculations were conducted using the indicated DFT functional with 6-311G(d,p) basis set in the gas phase (533.15 K, 1 atm). [a] Def2-TZVP was used as a basis set.

Racemization Dynamics in the course of cyclization reaction into 6O

Geometry optimization and frequency calculations were conducted with implicit solvation (SMD model for DMF) at the B3LYP-D3(BJ)/6-31+G(d) level of theory employing SDD as an ECP for Cs. Single point energies were calculated at the B3LYP-D3(BJ)/6-311+G(2df,p) level of theory employing SDD as an ECP for Cs, and SMD solvation model for DMF. Energies of all structures were corrected to a reference state of 1 mol/L at 403.15 K through addition of RTln(0.08206×T) = 2.80 kcal/mol to every species. We have determined DMI to be the optimal solvent for the synthesis of **6O** experimentally, but we have observed almost the same yield and enantioselectivity in the case of using DMF as a solvent.

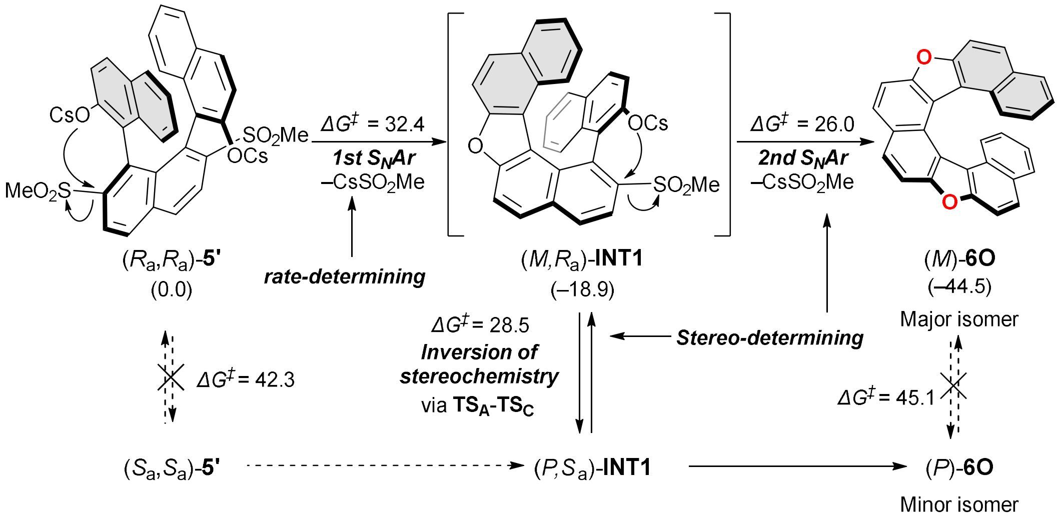

Scheme S1 Outline of the cyclization of **5'** and racemization dynamics.

$\Delta G^{\ddagger}$ values represent the activation free energies (kcal/mol) of the forward reaction. The numbers in parentheses are the relative free energies (kcal/mol).

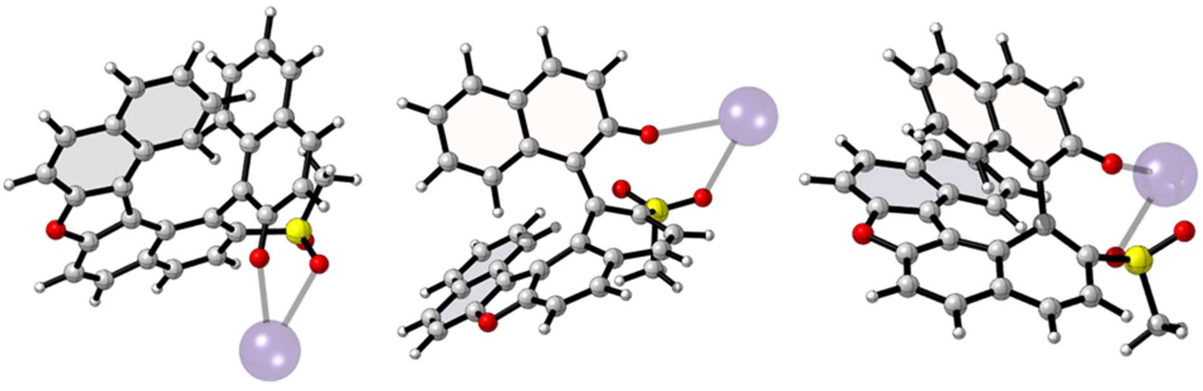

Fig. S26. The optimized structure of transition states.

Experimental verification

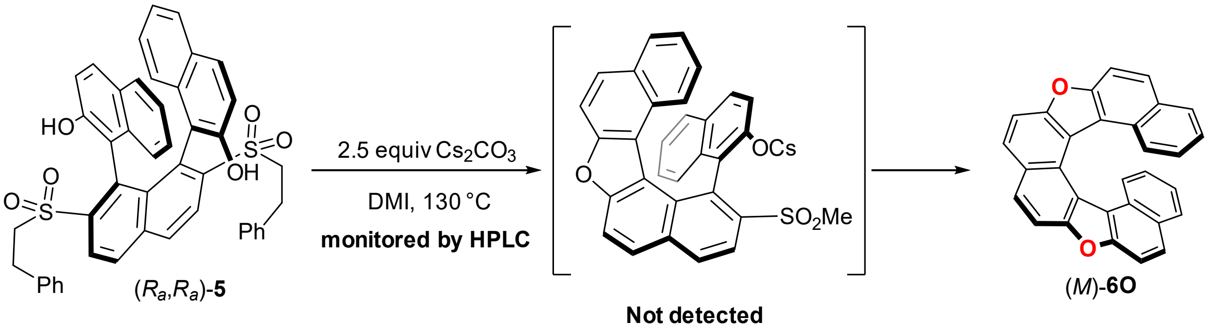

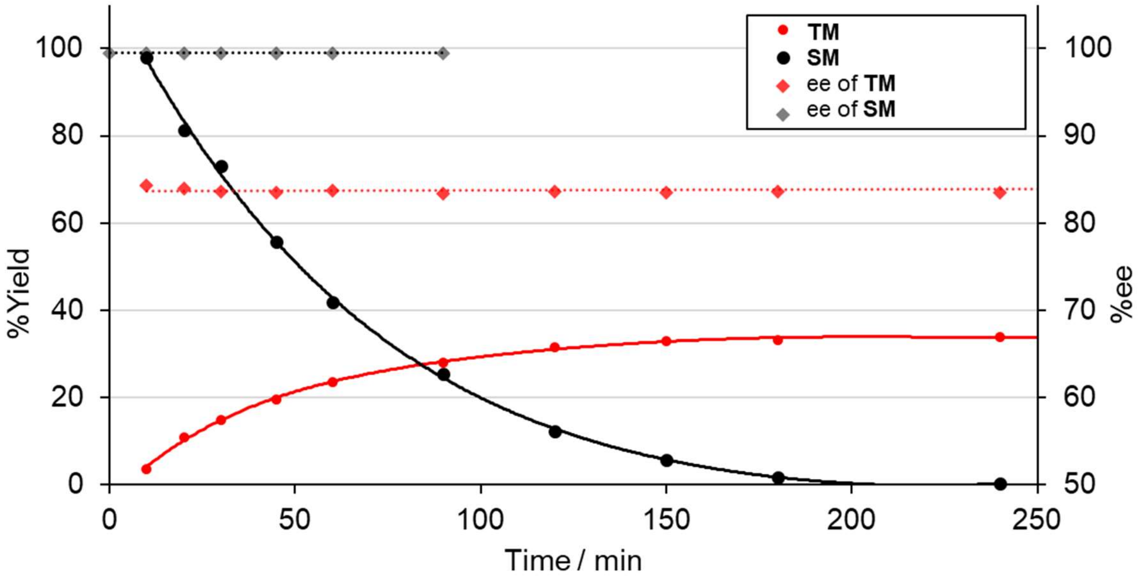

Scheme S2 Time-course of yield and ee of the reaction components.

A 10-mL Schlenk tube was charged with (R_a,R_a)-**5** (74.8 mg, 0.10 mmol), Cs_2CO_3 (81.0 mg, 0.25 mmol), dibenzofuran (16.4 mg, 0.10 mmol for an internal standard), and DMI (3 mL). The resulting solution was stirred at room temperature for 10 min and at 130 °C for 6 h. *ca*. 0.1 mL of the reaction mixture was taken from the Schlenk tube at the indicated time. The aliquot was neutralized by CH_2Cl_2/AcOH (1 mL, 20:1), filtered, and then analyzed by HPLC using dibenzofuran as an internal standard. During the reaction, racemization of (R_a,R_a)-**5** could not be detected, and ee of **6O** was almost constant (84%ee). According to the conversion of the starting material and the product yield, noticeable accumulation of a reaction intermediate was unlikely although mass-balance was not good.

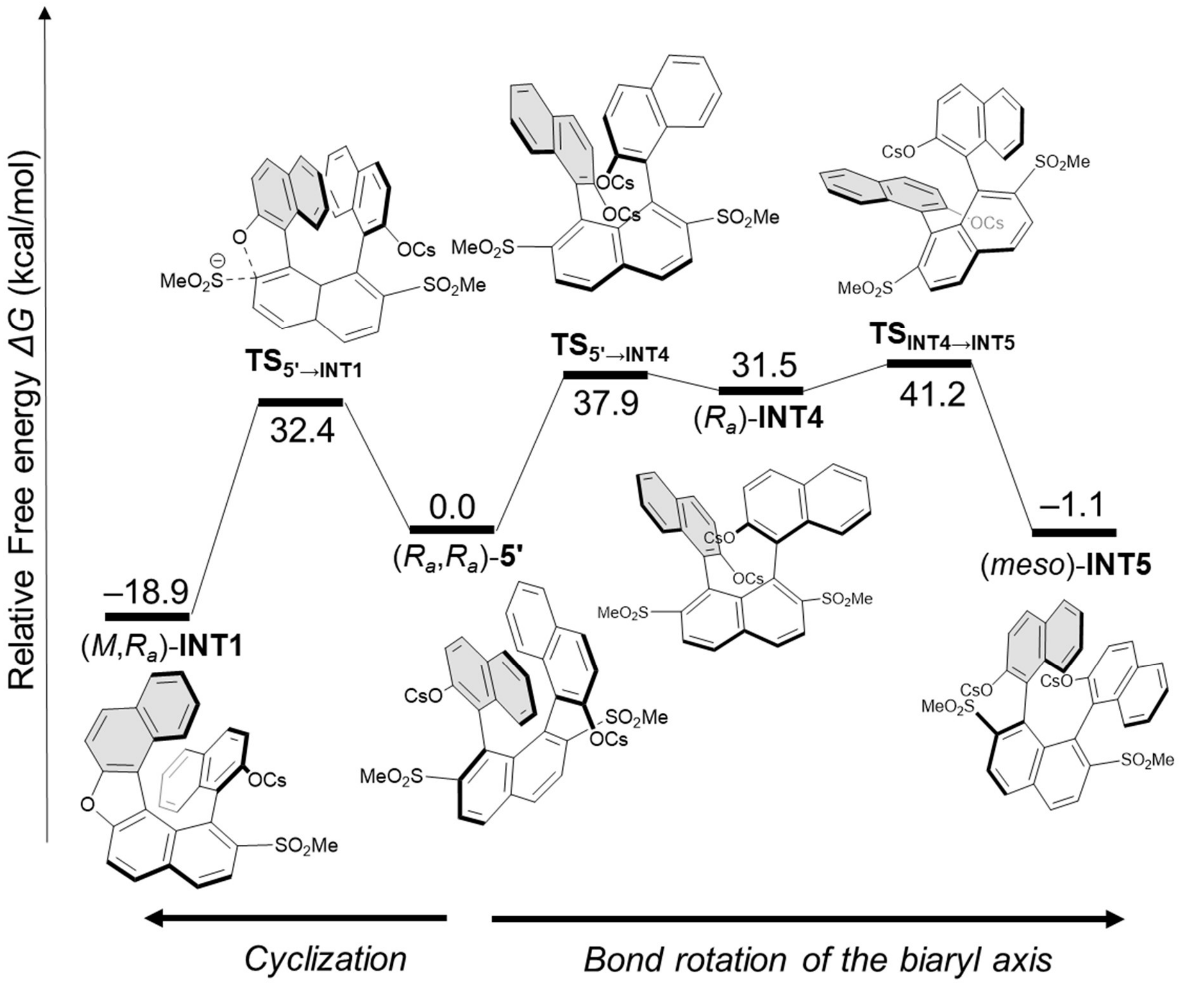

Scheme S3 Free energy profile of the stereo-inversion of (R,R)-**5'**. The pathway from (*meso*)-**INT5** to (P,S)-**INT1** is omitted because it is the mirror symmetric with the shown pathway.

6. Optical Measurement

TD-DFT calculations were also conducted at B3LYP-D3(BJ)/6-311G(d,p) level in the gas phase.

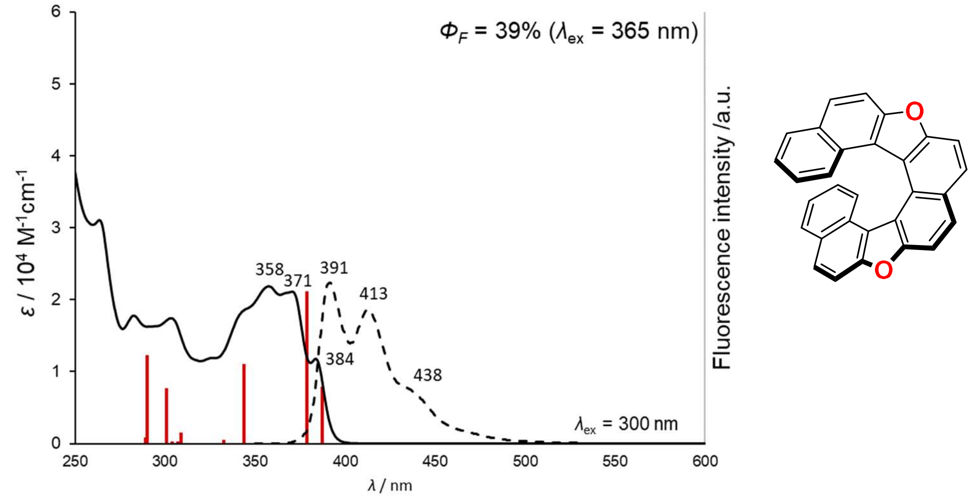

Fig. S27 UV/Vis absorption spectra in CH_2Cl_2 (9.0×10^{-6} M, solid line), the calculated oscillator strengths *f* (red bars), and fluorescence spectra in CH_2Cl_2 (2.4×10^{-6} M, dashed line) of (*rac*)-**6O**.

Table S7 Calculated electronic transition of (*M*)-**6O**.

No.	Wavelength (nm)	coefficients	Electronic Transition	*f*	*R*
1	387.48	–0.15906	105 HOMO–1 → 107 LUMO	0.0790	–211.5630
		0.68078	106 HOMO → 108 LUMO+1		
2	378.94	0.68892	106 HOMO → 107 LUMO	0.2112	151.3496
		0.13425	104 HOMO–2 → 108 LUMO+1		
3	343.78	0.67345	105 HOMO–1 → 107 LUMO	0.1106	–274.7852
		0.15861	106 HOMO → 108 LUMO+1		

R: Rotatory Strength ($\times10^{-40}$ esu·cm·erg/Gauss).

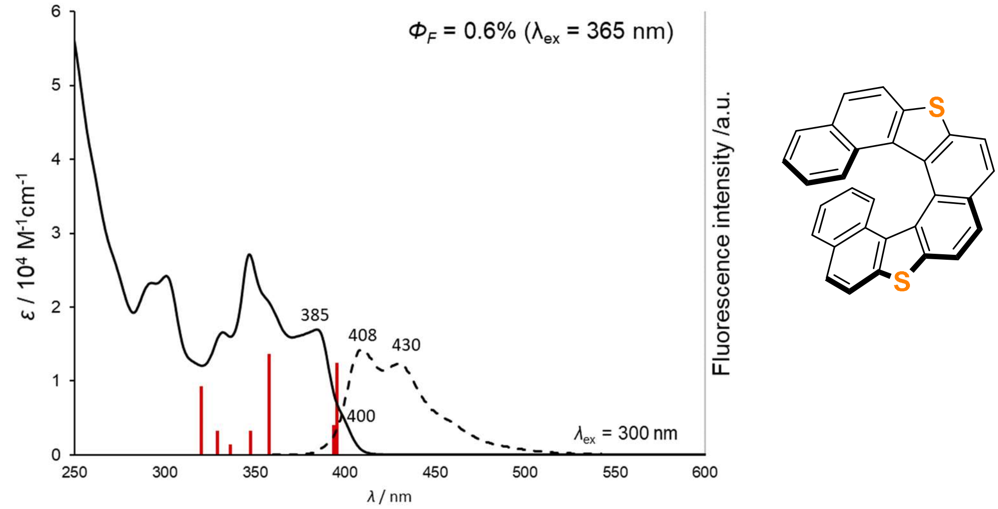

Fig. S28 UV/Vis absorption spectra in CH_2Cl_2 (1.4×10^{-5} M, solid line), the calculated oscillator strengths *f* (red bars), and fluorescence spectra in CH_2Cl_2 (2.4×10^{-6} M, dashed line) of (*rac*)-**6S**.

Table S8 Calculated electronic transition of (*M*)-**6S**.

No.	Wavelength (nm)	coefficients	Electronic Transition	*f*	*R*
1	395.52	0.11088	112 HOMO–2 → 116 LUMO+1	0.1247	62.5373
		–0.13792	113 HOMO–1 → 116 LUMO+1		
		0.67704	114 HOMO → 115 LUMO		
2	394.24	0.21798	113 HOMO–1 → 115 LUMO	0.0397	–162.8200
		0.66518	114 HOMO → 116 LUMO+1		
3	357.99	0.18371	112 HOMO–2 → 115 LUMO	0.1360	–194.5801
		0.61055	113 HOMO–1 → 115 LUMO		
		-0.22301	114 HOMO → 116 LUMO+1		
		0.12777	114 HOMO → 118 LUMO+3		
		0.10262	114 HOMO → 120 LUMO+5		

R: Rotatory Strength (×10^{-40} esu·cm·erg/Gauss).

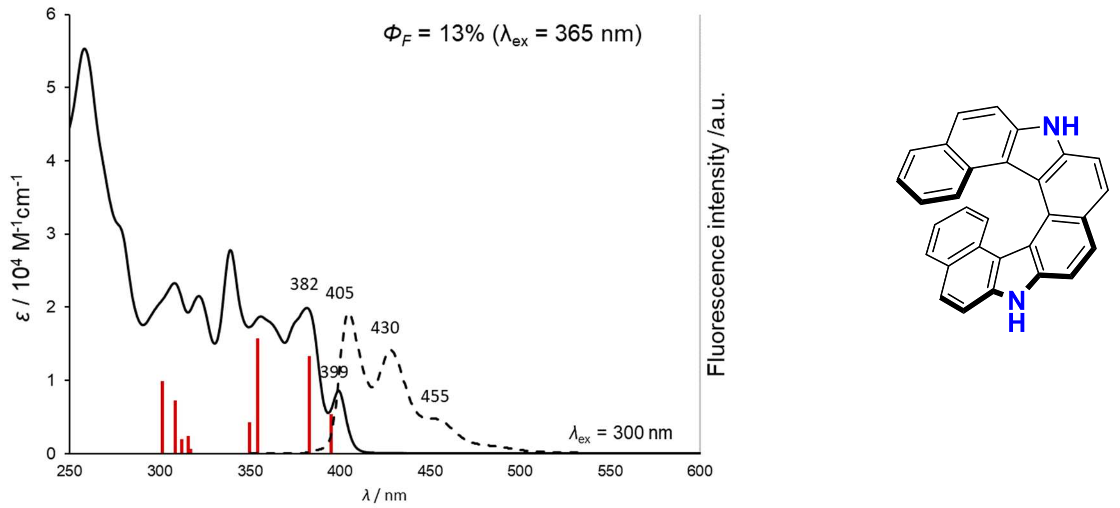

Fig. S29 UV/Vis absorption spectra in CH_2Cl_2 (1.0×10^{-5} M, solid line), the calculated oscillator strengths *f* (red bars), and fluorescence spectra in CH_2Cl_2 (2.4×10^{-6} M, dashed line) of (*rac*)-**6N**.

Table S9 Calculated electronic transition of (*M*)-**6N**.

No.	Wavelength (nm)	coefficients	Electronic Transition	*f*	*R*
1	395.20	0.25318	105 HOMO–1 → 107 LUMO	0.0540	–171.8732
		0.65387	106 HOMO → 108 LUMO+1		
2	383.05	–0.14156	104 HOMO–2 → 108 LUMO+1	0.1337	92.3011
		–0.22678	105 HOMO–1 → 108 LUMO+1		
		0.64616	106 HOMO → 107 LUMO		
3	354.70	0.63770	105 HOMO–1 → 107 LUMO	0.1576	–319.5628
		–0.24928	106 HOMO → 108 LUMO+1		

R: Rotatory Strength (×10^{-40} esu·cm·erg/Gauss)

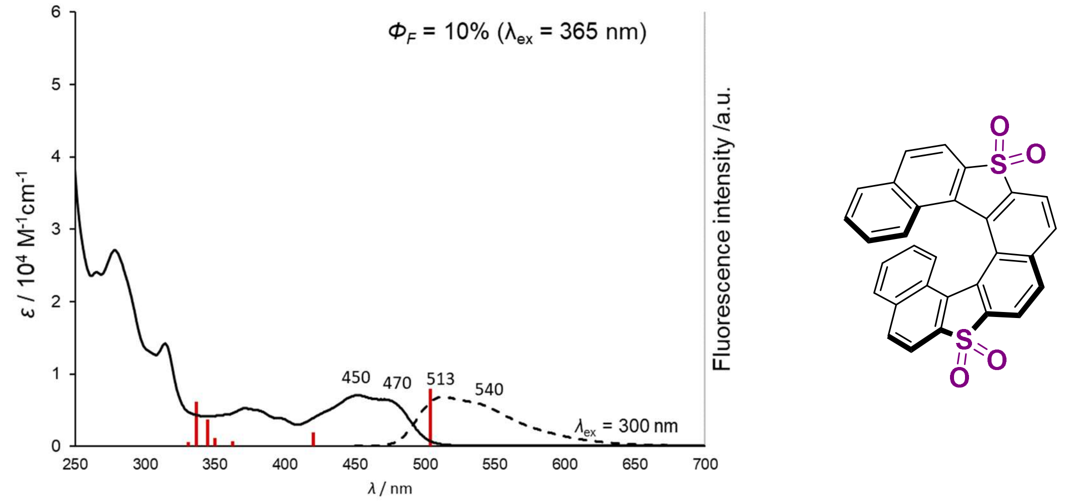

Fig. S30 UV/Vis absorption spectra in CH_2Cl_2 (4.0×10^{-5} M, solid line), the calculated oscillator strengths *f* (red bars), and fluorescence spectra in CH_2Cl_2 (2.6×10^{-6} M, dashed line) of (*rac*)-**6SO₂**.

Table S10 Calculated electronic transition of (*M*)-**6SO₂**.

No.	Wavelength (nm)	coefficients	Electronic Transition	*f*	*R*
1	503.87	0.0797	130 HOMO → 131 LUMO	0.0797	–11.5777
2	419.08	0.21926	129 HOMO–1 → 131 LUMO	0.0344	28.4925
		0.66959	130 HOMO → 132 LUMO+1		
3	400.22	0.66668	129 HOMO–1 → 131 LUMO	0.0197	–48.1803
		–0.21768	130 HOMO → 132 LUMO+1		

R: Rotatory Strength ($\times10^{-40}$ esu·cm·erg/Gauss).

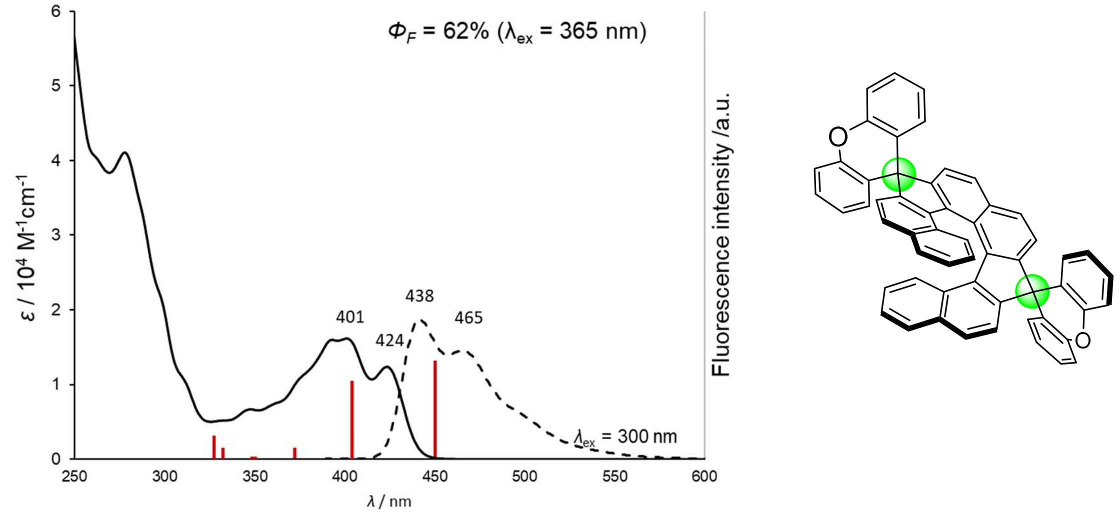

Fig. S31 UV/Vis absorption spectra in CH_2Cl_2 (1.1×10^{-5} M, solid line), the calculated oscillator strengths *f* (red bars), and fluorescence spectra in CH_2Cl_2 (2.4×10^{-6} M, dashed line) of (*rac*)-**6CX**.

Table S11 Calculated electronic transition of (*M*)-**6CX**.

No.	Wavelength (nm)	coefficients	Electronic Transition	*f*	*R*
1	450.34	0.70072	192 HOMO → 193 LUMO	0.1316	55.1049
2	404.19	0.10549	191 HOMO–1 → 193 LUMO	0.1049	–26.4094
		0.69363	192 HOMO → 194 LUMO+1		
3	372.41	0.68233	191 HOMO–1 → 193 LUMO	0.0152	–65.1929
		0.12996	192 HOMO → 195 LUMO+2		

R: Rotatory Strength (×10^{-40} esu·cm·erg/Gauss).

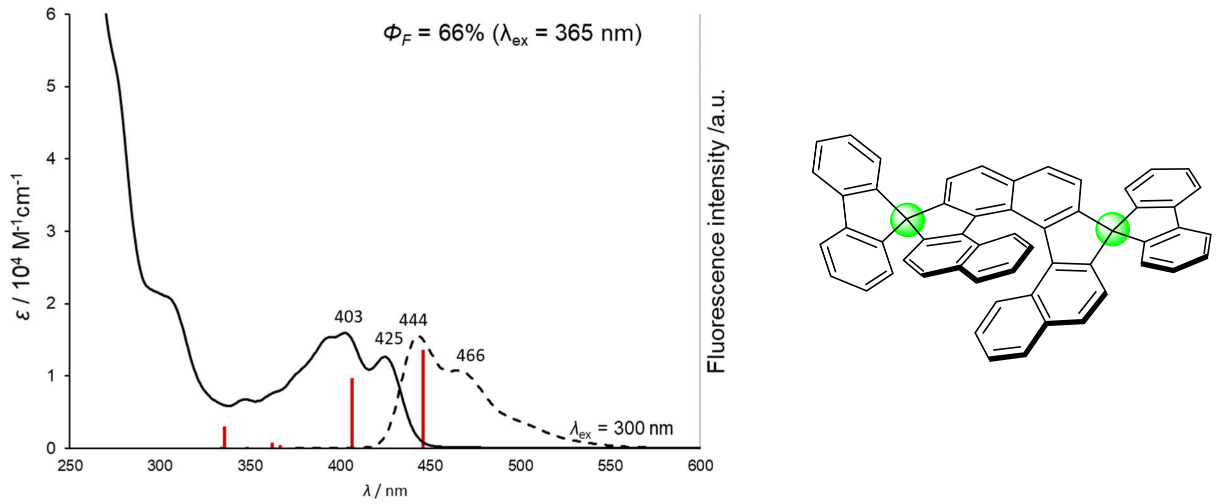

Fig. S32 UV/Vis absorption spectra in CH_2Cl_2 (1.0×10^{-5} M, solid line), the calculated oscillator strengths *f* (red bars), and fluorescence spectra in CH_2Cl_2 (2.4×10^{-6} M, dashed line) of (*rac*)-**6CF**.

Table S12 Calculated electronic transition of (*M*)-**6CF**.

No.	Wavelength (nm)	coefficients	Electronic Transition	*f*	*R*
1	446.99	0.70061	184 HOMO → 185 LUMO	0.1358	25.1358
2	406.27	0.69784	184 HOMO → 186 LUMO+1	0.0970	–31.3872
3	366.68	0.65582	183 HOMO–1 → 185 LUMO	0.0049	–39.2482
		0.15942	184 HOMO → 188 LUMO+3		
		0.17591	184 HOMO → 189 LUMO+4		

R: Rotatory Strength (×10^{-40} esu·cm·erg/Gauss).

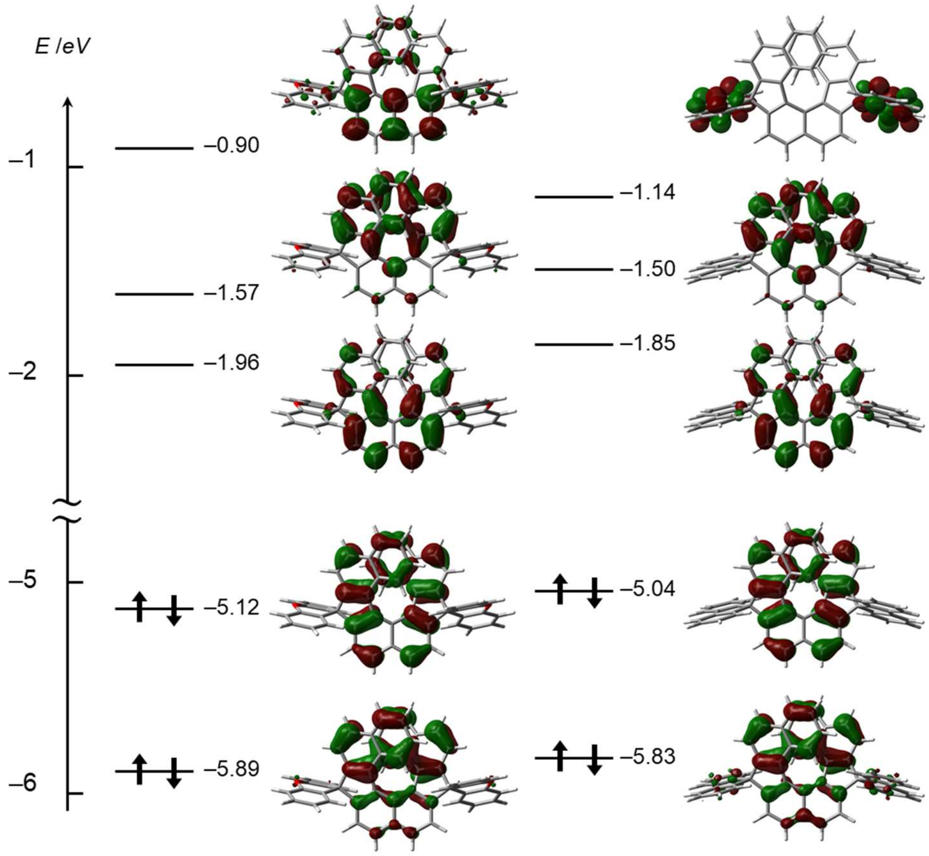

Fig. S33 Kohn-Sham orbital representations of **6CX** and **6CF** (isovalue:0.03).

Table S13 Summary of calculated electric and magnetic transition dipole moments and relative angle (θ) of the lowest energy electronic transitions.[9]

Compound	Wavelength (*f*)	$\|\mu_e\|$	$\|\mu_m\|$	θ	*R*
(*M*)-**6N**	395 nm (0.054)	213.04	1.29	129°	–171.88
(*M*)-**6O**	387 nm (0.079)	255.19	1.64	120°	–211.59
(*M*)-**6S**[a]	394 nm (0.040)	182.46	1.20	138°	–162.84
(*M*)-**6CF**	447 nm (0.136)	359.32	0.07	0.0°	25.12
(*M*)-**6CX**	450 nm (0.132)	355.06	0.16	0.0°	55.09
(*M*)-**6SO₂**	504 nm (0.020)	292.20	0.04	180°	–11.57

μ_e: transition electronic dipole moment ($\times 10^{-20}$ esu·cm), μ_m: transition magnetic dipole moment ($\times 10^{-20}$ erg/Gauss), θ: the angles of the transition electronic dipole moment and the transition magnetic dipole moment.

The rotatory strength *R* ($\times 10^{-40}$ esu·cm·erg/Gauss) is defined as the imaginary part of scalar product of μ_e and μ_m.

$$R = \mathrm{Im}(\mu_e \bullet \mu_m) = |\mu_e||\mu_m|\cos\theta$$

[a] According to TD-DFT calculation, the two lowest energy electronic transitions of **6S** were energetically degenerated, and the second lowest energy transition would be mainly responsible for the CD response. Thus, the parameters of the second lowest energy transition are shown here.

Fluorescence life-time measurement

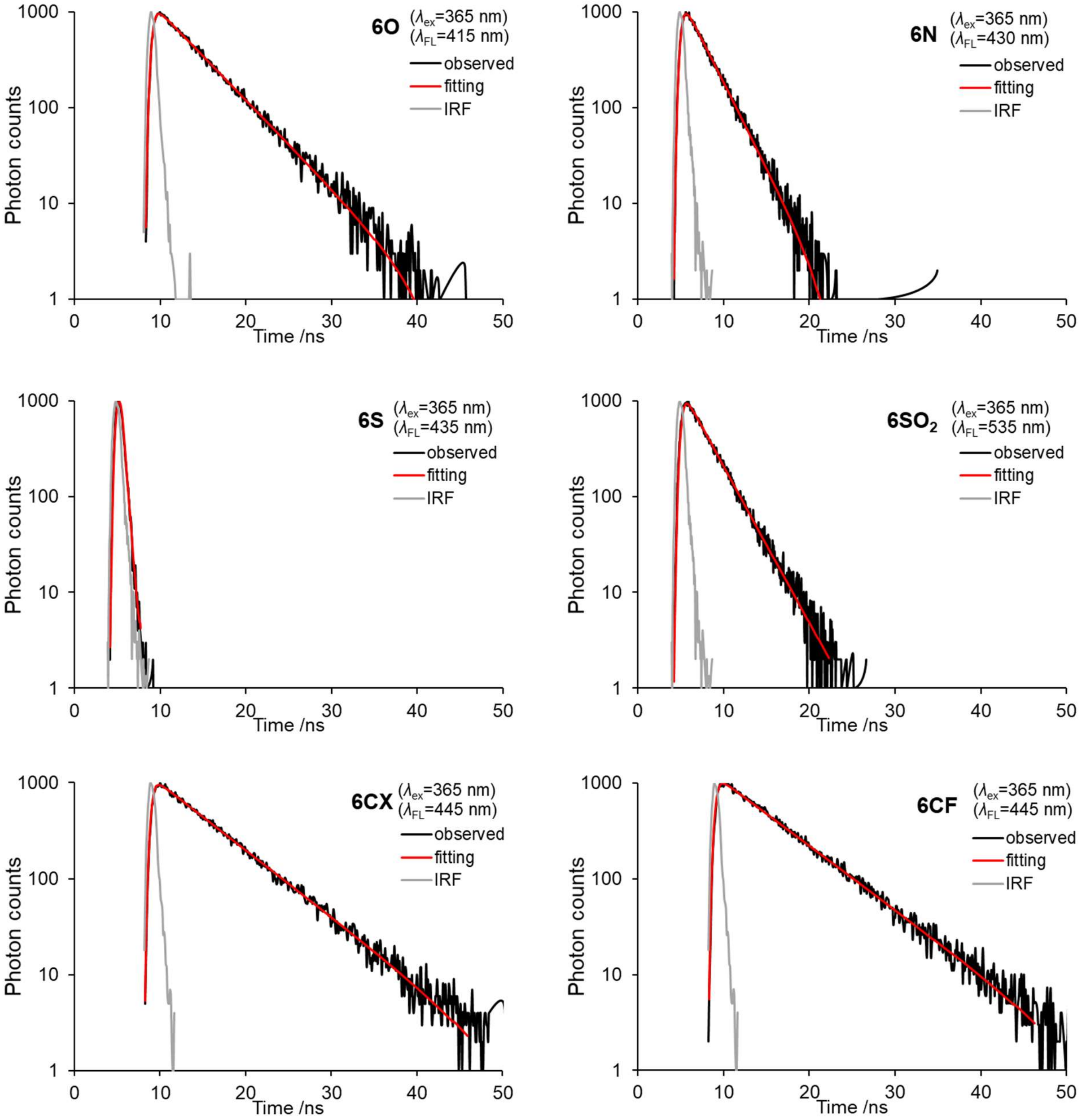

Fig. S34 Fluorescence decay profiles of helicenes in CH_2Cl_2 (IRF = instrumental response function). The concentration of samples was as same as that of fluorescence measurement.

Circular Dichroism (CD) Spectra

|g_{abs}| values shown in Table 1 are the average of the values of two isomers.

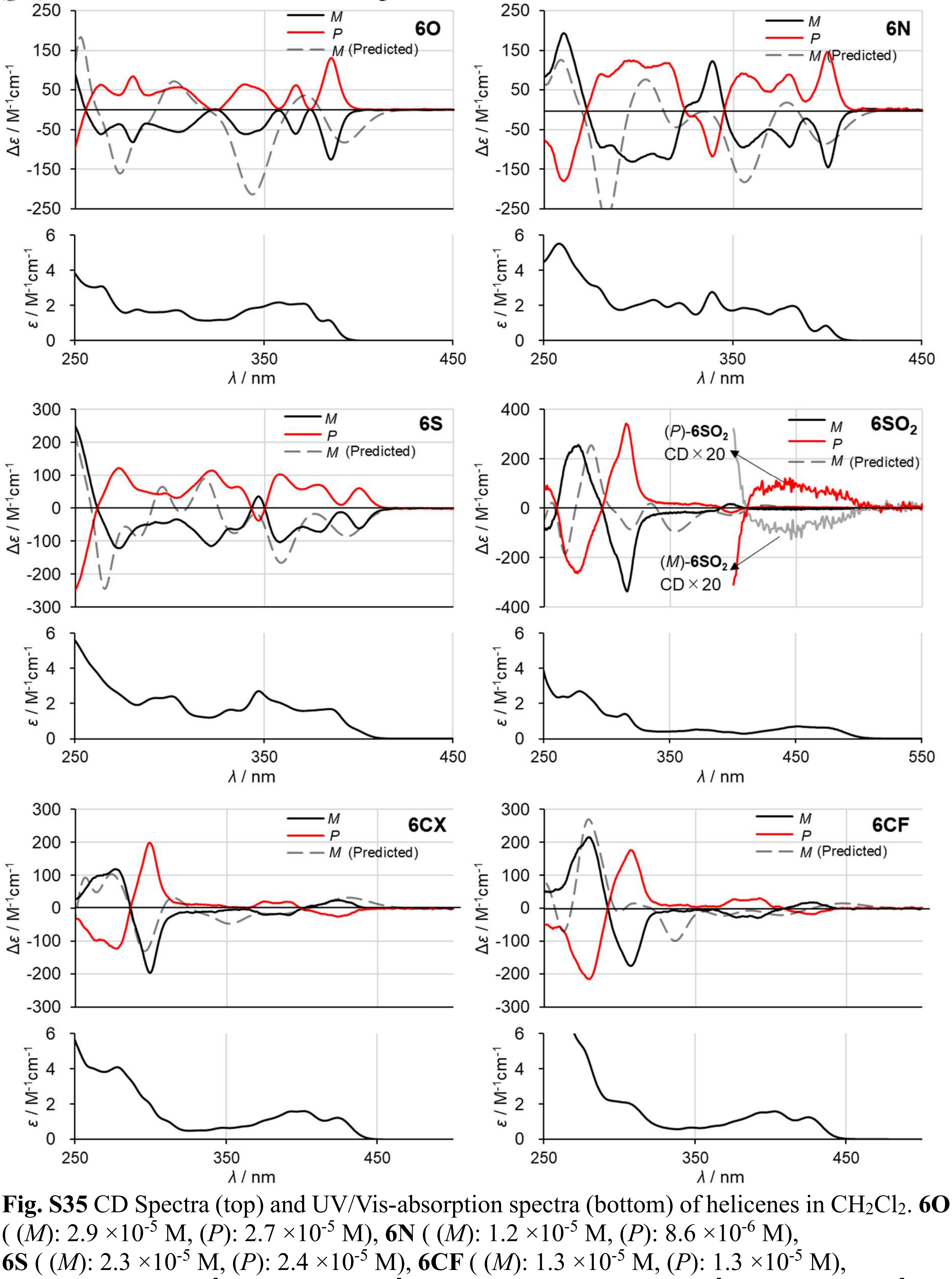

Fig. S35 CD Spectra (top) and UV/Vis-absorption spectra (bottom) of helicenes in CH_2Cl_2. **6O** ((*M*): 2.9 ×10^{-5} M, (*P*): 2.7 ×10^{-5} M), **6N** ((*M*): 1.2 ×10^{-5} M, (*P*): 8.6 ×10^{-6} M), **6S** ((*M*): 2.3 ×10^{-5} M, (*P*): 2.4 ×10^{-5} M), **6CF** ((*M*): 1.3 ×10^{-5} M, (*P*): 1.3 ×10^{-5} M), **6CX** ((*M*): 1.7 ×10^{-5} M, (*P*): 1.7 ×10^{-5} M), and **6SO$_2$** ((*M*): 2.6 ×10^{-5} M, (*P*): 2.9 ×10^{-5} M). Simulated CD spectra are shown as gray dashed lines (peak half-width: 0.1 eV).

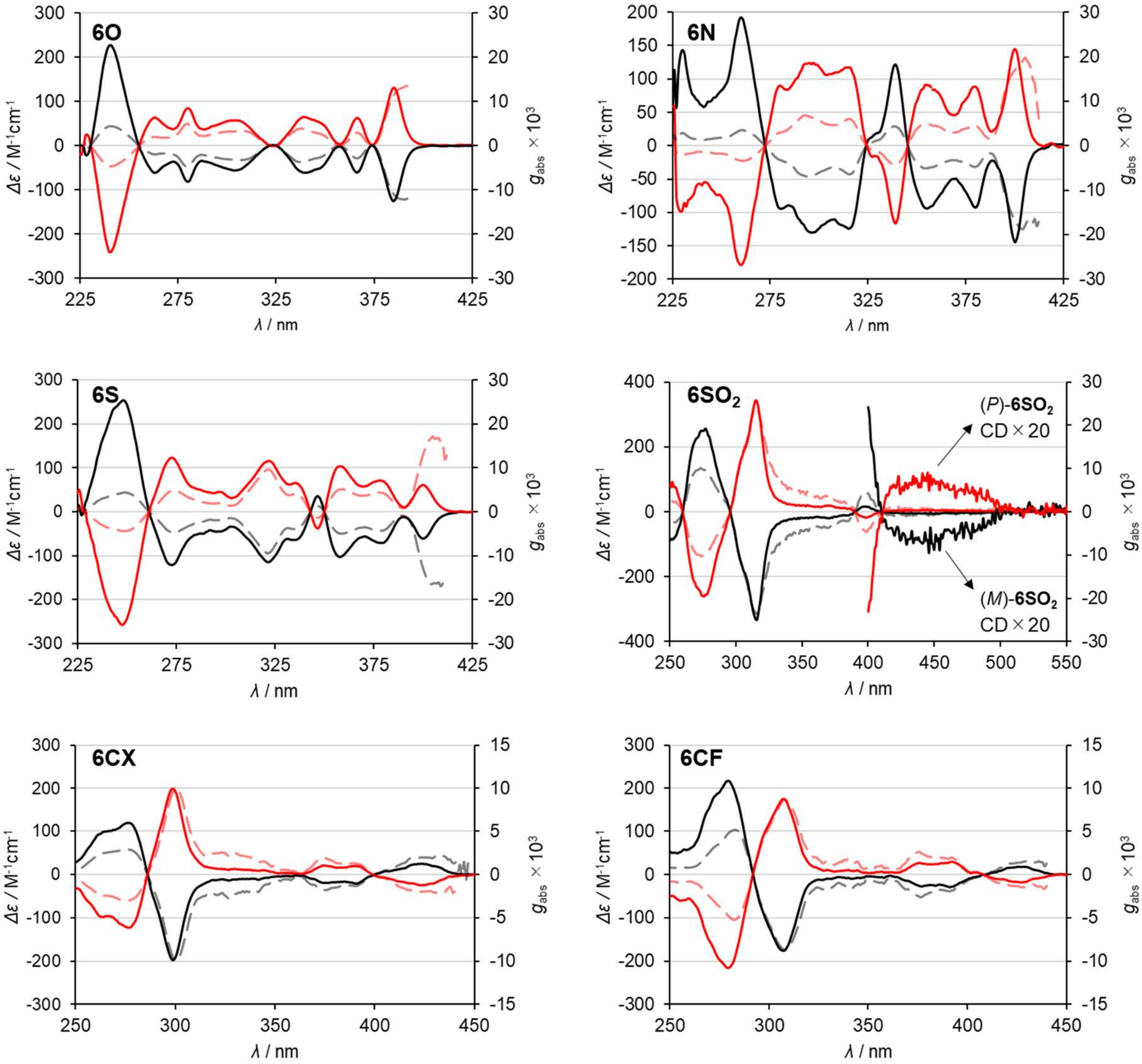

Fig. S36 CD Spectra (solid lines) and g_{abs} values (dashed lines) of helicenes. The red lines were spectra from the (*P*)-isomers, and the black lines are the spectra of (*M*)-isomers.

Circularly Polarized Luminescence (CPL) Spectra

|g_{lum}| values shown in Table 1 are the average of the values of two isomers.

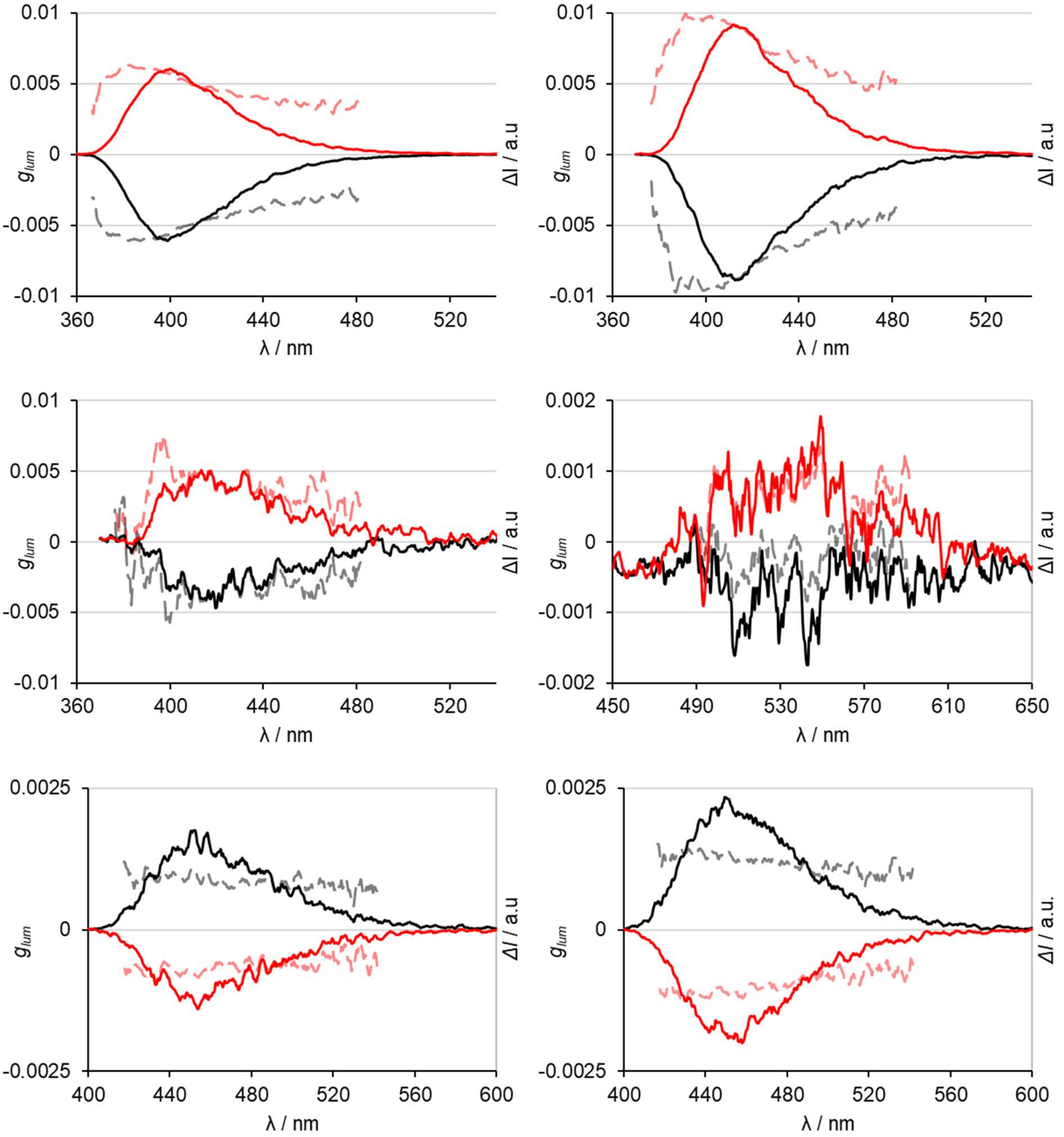

Fig. S37 CPL Spectra (solid lines) and g_{lum} values (dashed lines) of helicenes in CH_2Cl_2 (2.5×10^{-6} M, excited at 300 nm). The red lines were spectra from the (*P*)-isomers, and the black lines are the spectra of (*M*)-isomers.

Reference

1 (a) A. Altomare, M. C. Burla, M. Camalli, G. Cascarano, C. Giacovazzo, A. Guagliardi, A. G. G. Moliterni, G. Polidori, R. Spagna, R. *J. Appl. Cryst.*, 1999, **32**, 115; (b) G. M. Sheldrick, *Acta Cryst*., 2008, **A64**, 112.

2 M. J. Frisch, G. W. Trucks, H. B. Schlegel, G. E. Scuseria, M. A. Robb, J. R. Cheeseman, G. Scalmani, V. Barone, G. A. Petersson, H. Nakatsuji, X. Li, M. Caricato, A. V. Marenich, J. Bloino, B. G. Janesko, R. Gomperts, B. Mennucci, H. P. Hratchian, J. V. Ortiz, A. F. Izmaylov, J. L. Sonnenberg, D. Williams-Young, F. Ding, F. Lipparini, F. Egidi, J. Goings, B. Peng, A. Petrone, T. Henderson, D. Ranasinghe, V. G. Zakrzewski, J. Gao, N. Rega, G. Zheng, W. Liang, M. Hada, M. Ehara, K. Toyota, R. Fukuda, J. Hasegawa, M. Ishida, T. Nakajima, Y. Honda, O. Kitao, H. Nakai, T. Vreven, K. Throssell, J. A. Montgomery, Jr., J. E. Peralta, F. Ogliaro, M. J. Bearpark, J. J. Heyd, E. N. Brothers, K. N. Kudin, V. N. Staroverov, T. A. Keith, R. Kobayashi, J. Normand, K. Raghavachari, A. P. Rendell, J. C. Burant, S. S. Iyengar, J. Tomasi, M. Cossi, J. M. Millam, M. Klene, C. Adamo, R. Cammi, J. W. Ochterski, R. L. Martin, K. Morokuma, O. Farkas, J. B. Foresman, and D. J. Fox, Gaussian, Inc., Wallingford CT, 2016.

3 (a) S. Maeda, Y. Harabuchi, Y. Sumiya, M. Takagi, K. Suzuki, M. Hatanaka, Y. Osada, T. Taketsugu, K. Morokuma, K. Ohno, GRRM17, see http://iqce.jp/GRRM/index_e.shtml (accessed date 17 Oct, 2020); (b) S. Maeda, K. Ohno, K. Morokuma, *Phys. Chem. Chem. Phys.*, 2013, **15**, 3683.

4 C. Y. Legault, CYLview, Version 1.0b, 2009, Université de Sherbrooke, (http://www.cylview.org).

5 T. Yanagi, S. Otsuka, Y. Kasuga, K. Fujimoto, K. Mukarami, K. Nogi, H. Yorimitsu, A. Osuka *J. Am. Chem. Soc.*, 2016, **138**, 14582.

6 H. Ghosh, R. Vavilala, A. M. Szpilman, *Tetrahedron Asymmetry*, 2015, **26**, 79.

7 A. Kaga, K. Nogi, H. Yorimitsu, *Chem. Eur. J.*, 2019, **25**, 14780.

8 R. H. Martin, M. J. Marchant, *Tetrahedron*, 1974, **30**, 347.

9 (a) H. Kubo, T. Hirose, T. Nakashima, T. Kawai, J. Hasegawa, K. Matsuda, *J. Phys. Chem. Lett.* 2021, **12**, 6868; (b) H. Tanaka, Y. Inoue, T. Mori, *ChemPhotoChem*, 2018, **2**, 386–402; (c) T. Mori, *Chem. Rev.* 2021, *in press* (DOI: 10.1021/acs.chemrev.0c01017).

XYZ Coordinates and Energies

• Structures of helicenes

6O

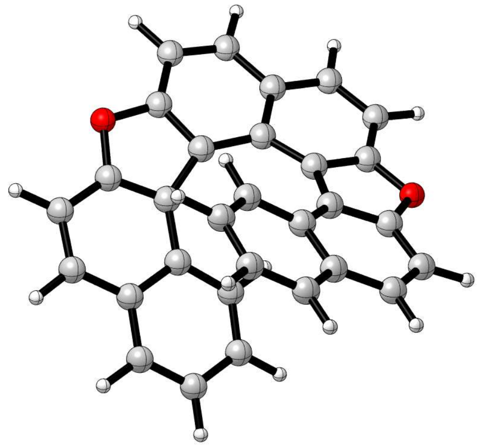

C	3.63061300	2.33777500	0.71831500
C	4.27414700	1.17234700	0.37770800
C	2.24420200	2.35910500	0.54404800
C	3.56033300	-0.00000300	-0.00000300
C	1.48557200	1.27635500	0.08329600
C	2.12036700	-0.00000200	-0.00000200
O	1.47397700	3.48066200	0.70251300
C	0.22194300	3.14903700	0.24784200
C	0.17687800	1.83625100	-0.21940900
H	4.16036200	3.22179900	1.04717800
H	5.35621000	1.12288700	0.41164700
C	-0.86861300	4.02692200	0.25599200
C	-0.98843700	1.41257600	-0.93075400
C	-2.12561700	2.28493500	-0.90864300
C	-2.04210900	3.56457900	-0.28656000
H	-0.77213900	5.02031100	0.67403500
H	-2.92224100	4.19718600	-0.29102600
C	3.63060800	-2.33778300	-0.71831500
C	4.27414500	-1.17235600	-0.37771100
C	2.24419700	-2.35911000	-0.54404400
C	1.48556900	-1.27635700	-0.08329500
O	1.47397000	-3.48066500	-0.70250900
C	0.22193600	-3.14903600	-0.24783900
C	0.17687400	-1.83625000	0.21941200
H	4.16035300	-3.22181000	-1.04717600
H	5.35620800	-1.12289800	-0.41165100
C	-0.86862100	-4.02692000	-0.25599100
C	-0.98844000	-1.41257500	0.93075500
C	-2.12562300	-2.28493100	0.90864000
C	-2.04211800	-3.56457400	0.28655700
H	-0.77214700	-5.02030900	-0.67403500
H	-2.92225000	-4.19718000	0.29102200
C	-3.31372600	-1.87378000	1.55998300
C	-1.06017300	-0.23248000	1.70334300
C	-3.37033000	-0.68757000	2.25151300
C	-2.22303900	0.12491400	2.34480200
H	-4.17923900	-2.52613800	1.51801400
H	-4.28654100	-0.38775500	2.74637600
H	-0.18303000	0.38836000	1.79840600
H	-2.25502200	1.03875500	2.92614600
C	-1.06017100	0.23248000	-1.70333900
H	-0.18303100	-0.38836300	-1.79839900
C	-3.31372000	1.87378800	-1.55998800
H	-4.17923200	2.52614600	-1.51801900
C	-3.37032600	0.68757700	-2.25151600
C	-2.22303700	-0.12491100	-2.34480000
H	-2.25502200	-1.03875100	-2.92614600
H	-4.28653700	0.38776300	-2.74638100

E(RB3LYP) = -1303.74580389
Zero-point correction=0.367404 (Hartree/Particle)
Thermal correction to Energy= 0.435525
Thermal correction to Enthalpy= 0.437213
Thermal correction to Gibbs Free Energy= 0.249813
Sum of electronic and zero-point Energies
= -1303.378400
Sum of electronic and thermal Energies
= -1303.310279
Sum of electronic and thermal Enthalpies
= -1303.308591
Sum of electronic and thermal Free Energies
= -1303.495991 (unit: a.u.)

6O-TS

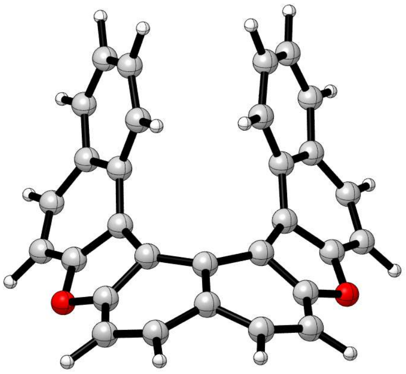

C	-0.39852200	3.74824300	2.32994000
C	-1.01145800	4.18938600	1.19232700
C	0.05983400	2.43285900	2.29780800
C	-0.97215700	3.41027500	0.00000000
C	-0.23707600	1.47453800	1.30895800
C	-0.58924900	2.01227400	0.00000000
O	0.85950000	1.93326300	3.28598200
C	1.00169600	0.60210400	3.01780800
C	0.23554300	0.22092900	1.91674100
H	-0.23749200	4.36948400	3.20038800
H	-1.37742000	5.20673000	1.12662400
C	1.79691800	-0.25747100	3.78915000
C	-0.12778800	-1.16866100	1.89851700
C	0.67267200	-2.08602300	2.66066300
C	1.69163400	-1.59888300	3.53119300
H	2.44644200	0.14312500	4.55625100
H	2.29413500	-2.31288900	4.08026100
C	-0.39852200	3.74824300	-2.32994000
C	-1.01145800	4.18938600	-1.19232700
C	0.05983400	2.43285900	-2.29780800
C	-0.23707600	1.47453800	-1.30895800
O	0.85950000	1.93326300	-3.28598200
C	1.00169600	0.60210400	-3.01780800
C	0.23554300	0.22092900	-1.91674100
H	-0.23749200	4.36948400	-3.20038800
H	-1.37742000	5.20673000	-1.12662400
C	1.79691800	-0.25747100	-3.78915000
C	-0.12778800	-1.16866100	-1.89851700
C	0.67267200	-2.08602300	-2.66066300
C	1.69163400	-1.59888300	-3.53119300
H	2.44644200	0.14312500	-4.55625100
H	2.29413500	-2.31288900	-4.08026100

C	0.30332100	-3.45075800	-2.69616800
C	-1.37487100	-1.62229500	-1.42981100
C	-0.89316900	-3.87321800	-2.16428500
C	-1.76311100	-2.93394200	-1.58547000
H	0.94423300	-4.14656400	-3.22669700
H	-1.18922600	-4.91264200	-2.24074100
H	-2.05984500	-0.89625600	-1.01784700
H	-2.74635900	-3.24558000	-1.25559800
C	-1.37487100	-1.62229500	1.42981100
H	-2.05984500	-0.89625600	1.01784700
C	0.30332100	-3.45075800	2.69616800
H	0.94423300	-4.14656400	3.22669700
C	-0.89316900	-3.87321800	2.16428500
C	-1.76311100	-2.93394200	1.58547000
H	-2.74635900	-3.24558000	1.25559800
H	-1.18922600	-4.91264200	2.24074100

Imaginary frequency: –41.16 cm^{-1}
E(RB3LYP) = -1303.67164007
Zero-point correction= 0.366304 (Hartree/Particle)
Thermal correction to Energy= 0.433006
Thermal correction to Enthalpy= 0.434695
Thermal correction to Gibbs Free Energy= 0.252105
Sum of electronic and zero-point Energies
= -1303.305336
Sum of electronic and thermal Energies
= -1303.238634
Sum of electronic and thermal Enthalpies
= -1303.236945
Sum of electronic and thermal Free Energies
= -1303.419535 (unit: a.u.)

6S

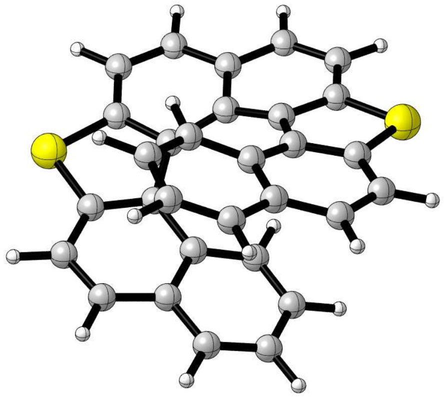

C	-3.56362100	2.41075500	-0.29822400
C	-4.21578400	1.21568200	-0.15652500
C	-2.17090100	2.42680400	-0.11587400
C	-3.49882700	-0.00013500	-0.00001100
C	-1.41602700	1.27458100	0.18190600
C	-2.06131000	-0.00007900	-0.00000600
S	-1.19505100	3.87812000	-0.19936300
C	0.22126700	2.97660000	0.29805700
C	-0.05895800	1.63165400	0.57542600
H	-4.10373200	3.32885200	-0.49281800
H	-5.29799200	1.17360800	-0.19782000
C	1.51588900	3.52392600	0.38024700
C	0.96848500	0.86180400	1.22090200
C	2.29716000	1.39102200	1.24156100
C	2.54316200	2.71457400	0.78234300
H	1.69095600	4.55639400	0.10367200
H	3.55864000	3.09279200	0.80980300

C	-3.56343000	-2.41103200	0.29820800
C	-4.21568500	-1.21600900	0.15649800
C	-2.17070800	-2.42697400	0.11587700
C	-1.41592700	-1.27469000	-0.18190000
S	-1.19473400	-3.87820700	0.19940000
C	0.22151400	-2.97657300	-0.29802600
C	-0.05882100	-1.63165300	-0.57540500
H	-4.10348000	-3.32916500	0.49280800
H	-5.29789700	-1.17402300	0.19778900
C	1.51617900	-3.52379500	-0.38021400
C	0.96855300	-0.86172600	-1.22090200
C	2.29727400	-1.39083700	-1.24155500
C	2.54338400	-2.71436200	-0.78232100
H	1.69133800	-4.55624400	-0.10362800
H	3.55889300	-3.09249900	-0.80977800
C	3.34605000	-0.60202700	-1.76994800
C	0.73381400	0.36948100	-1.87418200
C	3.09482500	0.62946500	-2.32567800
C	1.77095000	1.10024500	-2.40772300
H	4.35446200	-1.00012800	-1.74641100
H	3.90648400	1.22517400	-2.72636700
H	-0.27763700	0.73616700	-1.96253300
H	1.56365800	2.04730400	-2.89112200
C	0.73384800	-0.36944800	1.87413900
H	-0.27757600	-0.73621900	1.96246900
C	3.34600000	0.60228900	1.76994400
H	4.35437800	1.00047700	1.74642000
C	3.09487700	-0.62923600	2.32564700
C	1.77104200	-1.10013800	2.40766500
H	1.56383000	-2.04723100	2.89103100
H	3.90658500	-1.22488300	2.72632900

E(RB3LYP) = -1949.71950091
Zero-point correction= 0.361192 (Hartree/Particle)
Thermal correction to Energy= 0.431607
Thermal correction to Enthalpy= 0.433295
Thermal correction to Gibbs Free Energy= 0.241375
Sum of electronic and zero-point Energies
= -1949.358309
Sum of electronic and thermal Energies
= -1949.287894
Sum of electronic and thermal Enthalpies
= -1949.286206
Sum of electronic and thermal Free Energies
= -1949.478126 (unit: a.u.)

6S-TS

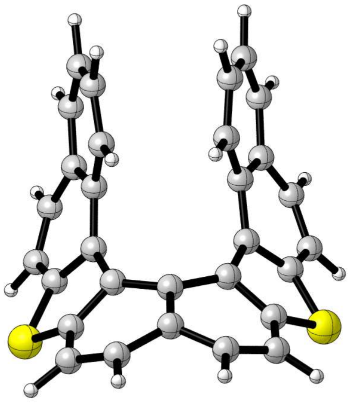

C	3.57644700	2.30393800	-0.75614700
C	3.92044100	1.20192800	-1.48495300
C	2.34319100	2.27783200	-0.08538800

C	3.16646200	0.00159300	-1.35202900
C	1.35898000	1.29367200	-0.30774500
C	1.84738700	0.00086900	-0.77551900
S	1.91830600	3.39647400	1.19999400
C	0.30984000	2.70402800	1.28685500
C	0.10097600	1.74129200	0.29249600
H	4.23836000	3.15594400	-0.66628300
H	4.87049300	1.16174300	-2.00446500
C	-0.72738800	3.17483100	2.12246200
C	-1.24322900	1.66727900	-0.21058300
C	-2.31238600	2.16156700	0.60189900
C	-2.01535400	2.81596200	1.83287100
H	-0.50036600	3.82597000	2.95766100
H	-2.83297600	3.15021100	2.46117900
C	3.57856500	-2.30061500	-0.75709600
C	3.92153200	-1.19799700	-1.48546200
C	2.34534700	-2.27587700	-0.08621400
C	1.36021600	-1.29252400	-0.30808600
S	1.92167800	-3.39535700	1.19882900
C	0.31262700	-2.70435600	1.28623500
C	0.10272100	-1.74144000	0.29227000
H	4.24125000	-3.15206100	-0.66761300
H	4.87152900	-1.15673800	-2.00499100
C	-0.72399500	-3.17634600	2.12192200
C	-1.24168600	-1.66829000	-0.21039500
C	-2.31022200	-2.16378600	0.60217400
C	-2.01233200	-2.81844400	1.83279600
H	-0.49621400	-3.82760800	2.95681900
H	-2.82951700	-3.15361900	2.46118000
C	-3.62234500	-2.17939100	0.07651700
C	-1.50683800	-1.41343600	-1.57198800
C	-3.85334100	-1.88633600	-1.24797100
C	-2.77458800	-1.55772200	-2.08684500
H	-4.43435600	-2.50676200	0.71663800
H	-4.85564000	-1.95080700	-1.65447600
H	-0.67899800	-1.16128400	-2.22126100
H	-2.94569700	-1.39566200	-3.14431900
C	-1.50777600	1.41287700	-1.57238200
H	-0.67954100	1.16170600	-2.22153800
C	-3.62437200	2.17635800	0.07587300
H	-4.43682900	2.50278300	0.71590900
C	-3.85474700	1.88375800	-1.24882400
C	-2.77548400	1.55642500	-2.08754700
H	-2.94614700	1.39479000	-3.14516000
H	-4.85697900	1.94763200	-1.65559100

Imaginary frequency: –36.36 cm^{-1}
E(RB3LYP) = -1949.63724540
Zero-point correction= 0.359641 (Hartree/Particle)
Thermal correction to Energy= 0.428879
Thermal correction to Enthalpy= 0.430567
Thermal correction to Gibbs Free Energy= 0.241582
Sum of electronic and zero-point Energies
= -1949.277604
Sum of electronic and thermal Energies
= -1949.208366
Sum of electronic and thermal Enthalpies
= -1949.206678
Sum of electronic and thermal Free Energies
= -1949.395664 (unit: a.u.)

6N

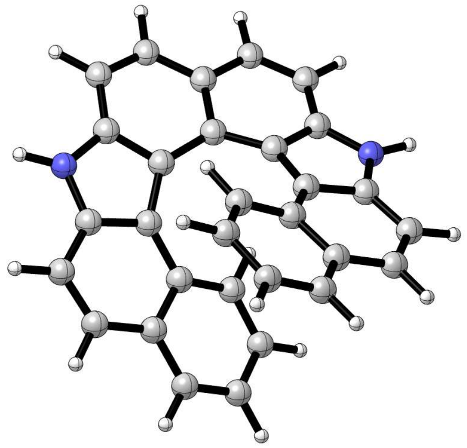

C	3.63966900	-2.36534700	-0.61838200
C	4.28803100	-1.19097400	-0.32423400
C	2.24599100	-2.39668400	-0.44068500
C	3.57928100	-0.00001000	0.00000100
C	1.50020300	-1.27554900	-0.00328800
C	2.14162800	-0.00000600	0.00000100
N	1.38850300	-3.47087000	-0.56069500
C	0.13178700	-3.10803000	-0.11222400
C	0.17548300	-1.76692200	0.31765100
H	4.18501100	-3.25125800	-0.92189300
H	5.37057000	-1.14670800	-0.36458700
C	-1.04432200	-3.88154400	-0.09236400
C	-0.95311400	-1.24285400	1.02357700
C	-2.16108800	-2.01195900	1.01265700
C	-2.17941900	-3.30923700	0.42094200
H	-1.05014900	-4.89319300	-0.48188800
H	-3.11014200	-3.86518700	0.42946100
C	3.63968300	2.36532700	0.61838200
C	4.28803800	1.19095000	0.32423600
C	2.24600500	2.39667300	0.44068400
C	1.50021100	1.27554100	0.00328900
N	1.38852400	3.47086500	0.56069000
C	0.13180700	3.10803200	0.11221900
C	0.17549500	1.76692300	-0.31765300
H	4.18503000	3.25123600	0.92189200
H	5.37057700	1.14667800	0.36458700
C	-1.04429800	3.88155300	0.09235600
C	-0.95310600	1.24285900	-1.02357600
C	-2.16107500	2.01197200	-1.01266000
C	-2.17939700	3.30925300	-0.42095000
H	-1.05011700	4.89320300	0.48187800
H	-3.11011700	3.86520900	-0.42947100
C	-3.31312700	1.48824800	-1.64528200
C	-0.92528700	0.05348800	-1.78613100
C	-3.27156400	0.29016700	-2.31918300
C	-2.05674100	-0.41514800	-2.41409000
H	-4.23231700	2.06302200	-1.60456600
H	-4.16241600	-0.09769700	-2.79925200
H	0.00411500	-0.48572000	-1.88273400
H	-2.01025700	-1.33539200	-2.98451400
C	-0.92528500	-0.05348900	1.78614000
H	0.00412100	0.48571100	1.88274700
C	-3.31313700	-1.48823000	1.64528100
H	-4.23233000	-2.06299600	1.60456100
C	-3.27156400	-0.29015200	2.31919000
C	-2.05673600	0.41515200	2.41410200
H	-2.01024400	1.33539100	2.98453300
H	-4.16241300	0.09771500	2.79926100
H	1.66376700	-4.40527600	-0.81131100
H	1.66379400	4.40526900	0.81130600

E(RB3LYP) = -1264.02961283
Zero-point correction= 0.392111 (Hartree/Particle)
Thermal correction to Energy= 0.462469
Thermal correction to Enthalpy= 0.464158
Thermal correction to Gibbs Free Energy= 0.272929
Sum of electronic and zero-point Energies
= -1263.637502
Sum of electronic and thermal Energies
= -1263.567143
Sum of electronic and thermal Enthalpies
= -1263.565455
Sum of electronic and thermal Free Energies
= -1263.756684 (unit: a.u.)

6N-TS

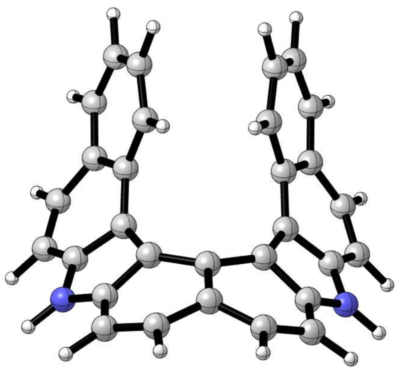

C	3.75973100	2.31960400	-0.42654500
C	4.17789100	1.19289700	-1.07140000
C	2.45352000	2.30070800	0.08207100
C	3.39884900	0.00000000	-1.02275400
C	1.48217400	1.30587400	-0.22754700
C	2.01572600	0.00000000	-0.60143500
N	1.90005800	3.23380500	0.92688000
C	0.56355700	2.96173200	1.09550400
C	0.22640700	1.86374500	0.27654100
H	4.40386700	3.17956700	-0.28766000
H	5.18297300	1.13191200	-1.47180800
C	-0.36642400	3.65325800	1.89988100
C	-1.15060400	1.83060400	-0.13301900
C	-2.12011900	2.52414300	0.66627900
C	-1.69227900	3.35435800	1.74550900
H	-0.03164800	4.40294100	2.60760900
H	-2.44165800	3.84653000	2.35469700
C	3.75973100	-2.31960400	-0.42654500
C	4.17789100	-1.19289700	-1.07140000
C	2.45352000	-2.30070800	0.08207100
C	1.48217400	-1.30587400	-0.22754700
N	1.90005800	-3.23380500	0.92688000
C	0.56355700	-2.96173200	1.09550400
C	0.22640700	-1.86374500	0.27654100
H	4.40386700	-3.17956700	-0.28766000
H	5.18297300	-1.13191200	-1.47180800
C	-0.36642400	-3.65325800	1.89988100
C	-1.15060400	-1.83060400	-0.13301900
C	-2.12011900	-2.52414300	0.66627900
C	-1.69227900	-3.35435800	1.74550900
H	-0.03164800	-4.40294100	2.60760900
H	-2.44165800	-3.84653000	2.35469700
C	-3.46843000	-2.54985000	0.24389800
C	-1.54375900	-1.41923000	-1.42120300
C	-3.83016000	-2.07645800	-0.99736800
C	-2.84218100	-1.56755200	-1.85583200
H	-4.20234800	-3.02843300	0.88368300
H	-4.85854300	-2.14565600	-1.33165200
H	-0.78215600	-1.05284100	-2.09448800
H	-3.10538500	-1.27975700	-2.86640600
C	-1.54375900	1.41923000	-1.42120300
H	-0.78215600	1.05284100	-2.09448800
C	-3.46843000	2.54985000	0.24389800
H	-4.20234800	3.02843300	0.88368300
C	-3.83016000	2.07645800	-0.99736800
C	-2.84218100	1.56755200	-1.85583200
H	-3.10538500	1.27975700	-2.86640600
H	-4.85854300	2.14565600	-1.33165200
H	2.39483400	-4.01121800	1.32983400
H	2.39483400	4.01121800	1.32983400

Imaginary frequency: –40.08 cm^{-1}
E(RB3LYP) = -1263.95187076
Zero-point correction= 0.390747 (Hartree/Particle)
Thermal correction to Energy= 0.459835
Thermal correction to Enthalpy= 0.461523
Thermal correction to Gibbs Free Energy= 0.274685
Sum of electronic and zero-point Energies
= -1263.561124
Sum of electronic and thermal Energies
= -1263.492036
Sum of electronic and thermal Enthalpies
= -1263.490348
Sum of electronic and thermal Free Energies
= -1263.677186 (unit: a.u.)

6SO₂

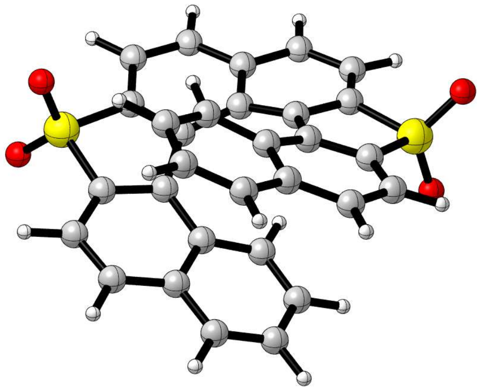

C	2.42336500	3.40111100	-0.15570700
C	1.22382100	4.06680500	-0.08401700
C	2.40191500	2.02005800	0.04757800
C	0.00004200	3.35441100	-0.00004400
C	1.25506800	1.26807200	0.25673500
C	0.00002700	1.90773000	-0.00001700
S	3.83977000	0.94929100	-0.07221700
C	2.89209200	-0.46233900	0.47327600
C	1.57009000	-0.11806300	0.70073400
H	3.36112500	3.92268700	-0.29755400
H	1.19148300	5.14901600	-0.11831200
C	3.41355900	-1.74767400	0.63565600
C	0.73396800	-1.08133100	1.34078800
C	1.22319200	-2.42771200	1.43624200
C	2.55412800	-2.73418400	1.05626900
H	4.45235600	-1.95297700	0.41137700
H	2.89788700	-3.75794900	1.14564600
C	-2.42327900	3.40116700	0.15563400
C	-1.22372200	4.06683400	0.08391000
C	-2.40186000	2.02010700	-0.04760800
C	-1.25503000	1.26809000	-0.25674100
S	-3.83973700	0.94936600	0.07224800
C	-2.89207900	-0.46230800	-0.47318400

C	-1.57008100	-0.11805600	-0.70069000
H	-3.36102700	3.92276800	0.29747300
H	-1.19136200	5.14904500	0.11816400
C	-3.41360900	-1.74760700	-0.63563100
C	-0.73399400	-1.08133600	-1.34076300
C	-1.22327400	-2.42769800	-1.43626100
C	-2.55422200	-2.73413100	-1.05630400
H	-4.45241000	-1.95287700	-0.41134100
H	-2.89802900	-3.75787400	-1.14575400
C	-0.36674400	-3.43194200	-1.95411200
C	0.53250200	-0.78632500	-1.90196900
C	0.88761400	-3.12112600	-2.41500500
C	1.32726300	-1.78006300	-2.41762900
H	-0.73249600	-4.45152400	-1.99604600
H	1.53316800	-3.89828300	-2.80625400
H	0.87522700	0.23654500	-1.93869400
H	2.29939600	-1.52565300	-2.82169800
C	-0.53250100	-0.78627200	1.90202500
H	-0.87518500	0.23661100	1.93876000
C	0.36662100	-3.43193300	1.95406600
H	0.73231700	-4.45153800	1.99594800
C	-0.88771200	-3.12107000	2.41499600
C	-1.32729900	-1.77998800	2.41767300
H	-2.29941200	-1.52555000	2.82177300
H	-1.53328900	-3.89820800	2.80624500
O	-4.19463000	0.82301800	1.48971500
O	-4.87558400	1.29069700	-0.90069500
O	4.19466300	0.82282700	-1.48967400
O	4.87562100	1.29069100	0.90069800

E(RB3LYP) = -2250.53152046
Zero-point correction= 0.377815 (Hartree/Particle)
Thermal correction to Energy= 0.458528
Thermal correction to Enthalpy= 0.460216
Thermal correction to Gibbs Free Energy= 0.242382
Sum of electronic and zero-point Energies
= -2250.153706
Sum of electronic and thermal Energies
= -2250.072993
Sum of electronic and thermal Enthalpies
= -2250.071304
Sum of electronic and thermal Free Energies
= -2250.289139 (unit: a.u.)

6SO$_2$-TS

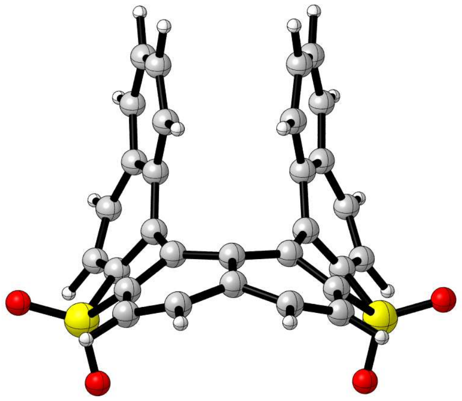

C	-2.32444200	-3.15558400	1.34155900
C	-1.20371800	-3.45735800	2.06875900
C	-2.29636300	-1.97937500	0.58985800
C	-0.00019200	-2.73803500	1.84132300
C	-1.28286100	-1.03264700	0.63297600
C	-0.00010900	-1.47279300	1.13319200
S	-3.32028400	-1.71959700	-0.87873700
C	-2.53818300	-0.13138600	-1.19982800
C	-1.69878200	0.17563800	-0.14243800
H	-3.18750400	-3.80828900	1.31787000
H	-1.16300400	-4.35845100	2.66839500
C	-3.03500400	0.81865900	-2.10402500
C	-1.65580100	1.54192400	0.26090700
C	-2.13349100	2.53581900	-0.65712300
C	-2.72922600	2.13906900	-1.88352300
H	-3.65328900	0.50995800	-2.93703300
H	-3.06322900	2.90289300	-2.57586900
C	2.32400900	-3.15588000	1.34158200
C	1.20324100	-3.45751000	2.06877200
C	2.29608200	-1.97967200	0.58987200
C	1.28270300	-1.03281200	0.63298100
S	3.32003200	-1.72005200	-0.87872900
C	2.53814200	-0.13174200	-1.19983900
C	1.69878600	0.17541300	-0.14244900
H	3.18698700	-3.80869700	1.31789700
H	1.16240700	-4.35859600	2.66841100
C	3.03510600	0.81822300	-2.10404100
C	1.65601400	1.54170900	0.26088700
C	2.13384500	2.53552600	-0.65715600
C	2.72952000	2.13867900	-1.88355300
H	3.65334500	0.50942400	-2.93704700
H	3.06363200	2.90244700	-2.57590700
C	2.16516800	3.88876800	-0.23955600
C	1.42344800	1.91955500	1.60309300
C	1.90572300	4.22481300	1.06652700
C	1.58677000	3.22187800	2.00410700
H	2.47464600	4.64532500	-0.95155000
H	1.98392000	5.25633200	1.38831300
H	1.17160800	1.15230900	2.32302800
H	1.44767500	3.48614600	3.04530700
C	-1.42315900	1.91972300	1.60311400
H	-1.17142300	1.15243300	2.32303900
C	-2.16460900	3.88906100	-0.23951000
H	-2.47398300	4.64567100	-0.95149300
C	-1.90509800	4.22505600	1.06657300
C	-1.58628000	3.22206700	2.00414100
H	-1.44712300	3.48630700	3.04534000
H	-1.98313600	5.25658500	1.38836800
O	4.72585300	-1.46209300	-0.56717000
O	2.97806600	-2.77457300	-1.83161300
O	-2.97846900	-2.77415000	-1.83164100
O	-4.72606700	-1.46145000	-0.56716400

Imaginary frequency: –32.84 cm^{-1}
E(RB3LYP) = -2250.45558332
Zero-point correction= 0.377024 (Hartree/Particle)
Thermal correction to Energy= 0.456176
Thermal correction to Enthalpy= 0.457864
Thermal correction to Gibbs Free Energy= 0.245068
Sum of electronic and zero-point Energies
= -2250.078559
Sum of electronic and thermal Energies
= -2249.999407
Sum of electronic and thermal Enthalpies
= -2249.997719
Sum of electronic and thermal Free Energies
= -2250.210515 (unit: a.u.)

6CF

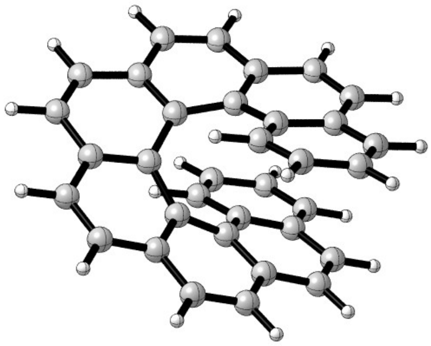

C	-2.43411500	-2.89930400	-0.08399000
C	-1.23193900	-3.56791600	-0.04933000
C	-2.43054400	-1.51053600	0.09120500
C	-0.00001700	-2.86386800	0.00019000
C	-1.25218200	-0.78538900	0.26288900
C	-0.00001000	-1.42082000	0.00011100
C	-2.98642700	0.74656500	0.48348400
C	-1.61943800	0.58428200	0.67933200
H	-3.37144600	-3.43256300	-0.18923100
H	-1.20419000	-4.65117600	-0.08412500
C	-3.61088400	1.99330300	0.60730100
C	-0.86530000	1.64519900	1.26086400
C	-1.48648400	2.93471700	1.33026800
C	-2.84974600	3.08335200	0.96471600
H	-4.67057900	2.09267500	0.40305400
H	-3.29677000	4.06948700	1.02280200
C	2.43407900	-2.89931300	0.08437400
C	1.23189800	-3.56791900	0.04979100
C	2.43052100	-1.51056500	-0.09098400
C	1.25216400	-0.78542600	-0.26274200
C	2.98642700	0.74648200	-0.48353200
C	1.61943200	0.58419300	-0.67934600
H	3.37140400	-3.43257200	0.18967500
H	1.20414200	-4.65117600	0.08471500
C	3.61089500	1.99319900	-0.60749400
C	0.86530400	1.64505800	-1.26099000
C	1.48649900	2.93456100	-1.33053700
C	2.84976700	3.08321900	-0.96501700
H	4.67059400	2.09258400	-0.40327000
H	3.29680600	4.06934100	-1.02321200
C	0.72442500	4.03313300	-1.80320800
C	-0.43318900	1.49264400	-1.80306900
C	-0.56116900	3.86208800	-2.25355500
C	-1.13352600	2.57258200	-2.28276200
H	1.18639900	5.01421800	-1.81906800
H	-1.13157800	4.71171500	-2.61025200
H	-0.86588800	0.50457900	-1.84196800
H	-2.13316100	2.43516300	-2.67723600
C	0.43318500	1.49283200	1.80297400
H	0.86588500	0.50477000	1.84197800
C	-0.72440700	4.03333100	1.80283700
H	-1.18637500	5.01442100	1.81858900
C	0.56117600	3.86232200	2.25322400
C	1.13352100	2.57281400	2.28257300
H	2.13314500	2.43542100	2.67708300
H	1.13158700	4.71198300	2.60983800
C	3.63634900	-0.58378700	-0.13444600
C	-3.63636400	-0.58373200	0.13454500
C	4.71466200	-0.98998500	-1.13478200
C	4.41986900	-0.57291500	1.17696700
C	5.76502400	-0.91014100	0.94859500
C	5.94685800	-1.17340800	-0.48511800
C	3.96630800	-0.27898400	2.45255200
C	6.66810800	-0.94745100	2.00911600
C	4.59317700	-1.17328000	-2.50203000
C	7.07396400	-1.54780900	-1.21372400
C	-4.71472300	-0.98981600	1.13486000
C	-4.41981600	-0.57302000	-1.17692400
C	-5.76497600	-0.91024400	-0.94858000
C	-5.94688200	-1.17334000	0.48515500
C	-3.96619500	-0.27924000	-2.45252200
C	-6.66800300	-0.94771200	-2.00914400
C	-4.59331000	-1.17294000	2.50213800
C	-7.07402000	-1.54767100	1.21374600
H	-2.92658400	-0.02988200	-2.62627900
C	-4.87290700	-0.31807800	-3.51382700
H	-4.53436100	-0.09227200	-4.51821700
H	-7.70789900	-1.20542400	-1.84344900
C	-6.21166600	-0.64819900	-3.29186900
H	-6.90216700	-0.67355500	-4.12689100
H	-3.63890100	-1.02481800	2.99422400
C	-5.72418000	-1.54865200	3.22988200
H	-5.64970700	-1.69695300	4.30080500
H	-8.02984600	-1.69282900	0.72323000
C	-6.95197600	-1.73375500	2.58992800
H	-7.81937100	-2.02505100	3.17092000
H	2.92670000	-0.02962900	2.62633000
C	4.87307700	-0.31766800	3.51381600
H	4.53457700	-0.09174400	4.51819400
H	7.70800100	-1.20516000	1.84339900
C	6.21183100	-0.64778400	3.29182700
H	6.90237800	-0.67301700	4.12681600
H	3.63873900	-1.02523700	-2.99408200
C	5.72401500	-1.54906200	-3.22978800
H	5.64948800	-1.69749900	-4.30068800
H	8.02981800	-1.69289200	-0.72324100
C	6.95184800	-1.73406400	-2.58987600
H	7.81921700	-2.02541700	-3.17088000

E(RB3LYP) = -2154.05465992
Zero-point correction= 0.693669 (Hartree/Particle)
Thermal correction to Energy= 0.817972
Thermal correction to Enthalpy= 0.819660
Thermal correction to Gibbs Free Energy= 0.513063
Sum of electronic and zero-point Energies
= -2153.360991
Sum of electronic and thermal Energies
= -2153.236688
Sum of electronic and thermal Enthalpies
= -2153.235000
Sum of electronic and thermal Free Energies
= -2153.541597 (unit: a.u.)

6CF-TS

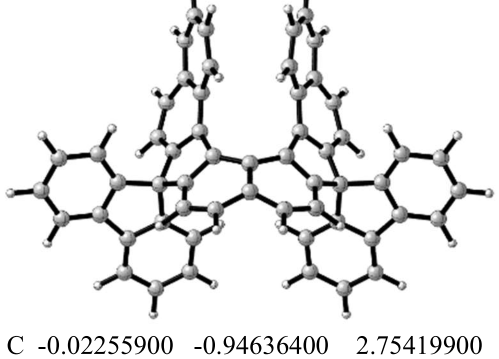

C	-0.02255900	-0.94636400	2.75419900
C	-0.01102000	0.04380000	1.69381600

C	2.29474200	-1.53010600	2.40866600
C	1.17265400	-1.57708700	3.19320300
C	2.28489700	-0.66713700	1.31038200
C	1.28773700	0.29053100	1.09821900
C	2.77524900	0.46316600	-0.69587100
C	1.80728600	1.16776400	0.01099000
H	3.14784600	-2.17147000	2.59432900
H	1.11793900	-2.25053900	4.04058700
C	3.36052100	0.96452600	-1.86897900
C	1.77797800	2.58278100	-0.17694400
C	2.37845900	3.11721500	-1.36642100
C	3.07731000	2.25360200	-2.25252100
H	4.04907200	0.34842300	-2.43539300
H	3.50035200	2.66669700	-3.16122500
C	2.42707100	4.52319200	-1.53270500
C	1.47039100	3.47633400	0.87290600
C	2.07766100	5.36543300	-0.50452600
C	1.65537700	4.83001800	0.72805200
H	2.82584400	4.92252000	-2.45891800
H	2.16899900	6.43863200	-0.62393600
H	1.14952800	3.06568800	1.82070800
H	1.45516100	5.49331800	1.56093100
C	3.22837500	-0.73553000	0.11747200
C	3.13338700	-2.09120900	-0.57779400
C	4.71487300	-0.66364300	0.45188600
C	5.37678300	-1.82147800	0.00851900
C	4.39509800	-2.70652900	-0.63408100
C	5.40259400	0.35328600	1.09276100
C	6.74684100	-1.96647600	0.21600500
C	2.01081200	-2.71136600	-1.09808400
C	4.53539300	-3.95813300	-1.23008900
H	4.88313800	1.24601600	1.42192400
C	6.77565600	0.20511600	1.29900600
H	7.33230400	0.98952600	1.79824600
H	7.26878400	-2.85523500	-0.12010400
C	7.43920300	-0.94484500	0.86480200
H	8.50546800	-1.04260200	1.03314900
H	1.04277600	-2.22998100	-1.03489200
C	2.15294900	-3.96622400	-1.69263500
H	1.28519100	-4.46947800	-2.10259400
H	5.50334300	-4.44357500	-1.28193100
C	3.40529600	-4.58135300	-1.75849700
H	3.49865600	-5.55598500	-2.22339500
C	-2.34121500	-1.50384300	2.38043300
C	-1.22906100	-1.56550900	3.17775300
C	-2.31039900	-0.63740000	1.28531000
C	-1.30037900	0.30906000	1.08341500
C	-2.79612200	0.52497500	-0.70123800
C	-1.80449900	1.20197400	0.00030300
H	-3.20498400	-2.13355100	2.55629400
H	-1.19195800	-2.24049200	4.02486100
C	-3.37638000	1.04045700	-1.87038700
C	-1.72762200	2.61400100	-0.19813300
C	-2.32194300	3.16220600	-1.38497300
C	-3.05900200	2.31946900	-2.25960700
H	-4.08504900	0.44179500	-2.43062300
H	-3.47746900	2.74308200	-3.16558300
C	-2.31928800	4.56743400	-1.56285300
C	-1.36763800	3.50385100	0.83718900
C	-1.91986900	5.40406500	-0.54855100
C	-1.49802200	4.86271000	0.68125600
H	-2.71596900	4.97388500	-2.48686100
H	-1.96947400	6.47900600	-0.67650600
H	-1.04828100	3.08933900	1.78353900
H	-1.25440500	5.52481300	1.50324900
C	-3.25933600	-0.67770900	0.09536700
C	-3.17674100	-2.01994400	-0.62743300
C	-4.73994500	-0.61125200	0.45454900
C	-5.40388700	-1.77382900	0.02644800
C	-4.43380200	-2.64656900	-0.64980900
C	-5.41920700	0.39888300	1.11486400
C	-6.76696000	-1.93017800	0.26877700
C	-2.07150100	-2.61093500	-1.21439400
C	-4.58352800	-3.88880100	-1.26301300
H	-4.89827900	1.29438200	1.43397600
C	-6.78569400	0.24014300	1.35440000
H	-7.33589900	1.01992000	1.86784400
H	-7.29020200	-2.82260200	-0.05538400
C	-7.45083900	-0.91470400	0.93595400
H	-8.51174300	-1.02100200	1.13087000
H	-1.11174900	-2.10911900	-1.19914200
C	-2.22389500	-3.85453700	-1.82981700
H	-1.37132300	-4.33178100	-2.29858600
H	-5.54795100	-4.38326600	-1.28858600
C	-3.46910100	-4.48720000	-1.85011200
H	-3.57048300	-5.45299900	-2.33144800

Imaginary frequency: -33.04 cm^{-1}
E(RB3LYP) = -2153.97745482
Zero-point correction= 0.691850 (Hartree/Particle)
Thermal correction to Energy= 815110
Thermal correction to Enthalpy= 0.816798
Thermal correction to Gibbs Free Energy= 0.509876
Sum of electronic and zero-point Energies=
-2153.285605
Sum of electronic and thermal Energies=
-2153.162345
Sum of electronic and thermal Enthalpies=
-2153.160657
Sum of electronic and thermal Free Energies=
-2153.467578

6CX

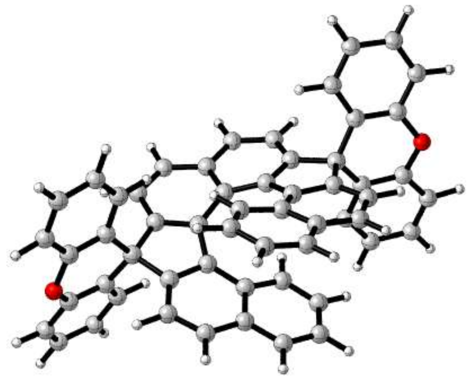

C	-2.43181700	-2.98886700	-0.12834800
C	-1.23155900	-3.66018400	-0.07220700
C	-2.43206000	-1.60041900	0.05091800
C	0.00002500	-2.95708900	0.00033300
C	-1.25591200	-0.87790800	0.24348000
C	0.00001100	-1.51442000	0.00021300
C	-2.98492500	0.66188800	0.42877000
C	-1.62431600	0.49318400	0.65212900
H	-3.36721100	-3.52111000	-0.25324400
H	-1.20480900	-4.74339200	-0.10796100
C	-3.60644600	1.91127300	0.53978100
C	-0.87595100	1.54969000	1.24867600
C	-1.49156700	2.84227200	1.30531000
C	-2.84654900	2.99714400	0.91247900
H	-4.66108900	2.01968600	0.31537400
H	-3.28998800	3.98538700	0.96119900
C	2.43187100	-2.98880200	0.12897600
C	1.23162400	-3.66014900	0.07297800

C	2.43208500	-1.60038500	-0.05053000
C	1.25591800	-0.87792700	-0.24318000
C	2.98489000	0.66187300	-0.42875300
C	1.62428300	0.49310300	-0.65206600
H	3.36728000	-3.52100800	0.25392500
H	1.20489400	-4.74335200	0.10890600
C	3.60637800	1.91125600	-0.53996500
C	0.87588000	1.54949600	-1.24876500
C	1.49146200	2.84208600	-1.30559900
C	2.84644600	2.99705200	-0.91281000
H	4.66102600	2.01972500	-0.31560800
H	3.28986100	3.98529800	-0.96169300
C	0.73333700	3.93617000	-1.79466800
C	-0.40921400	1.39025400	-1.81836800
C	-0.54126400	3.75747900	-2.27337400
C	-1.10567600	2.46474000	-2.31513500
H	1.19015000	4.91976100	-1.80147200
H	-1.10787800	4.60390800	-2.64352400
H	-0.83894800	0.40258100	-1.86844200
H	-2.09358600	2.31544100	-2.73403000
C	0.40913900	1.39056600	1.81831600
H	0.83889500	0.40291100	1.86853100
C	-0.73347600	3.93644800	1.79422400
H	-1.19031200	4.92002900	1.80087400
C	0.54112400	3.75786100	2.27297200
C	1.10556900	2.46514300	2.31493100
H	2.09347900	2.31593200	2.73385800
H	1.10771300	4.60436000	2.64300100
C	3.63930100	-0.67344600	-0.06194200
C	-3.63928000	-0.67349200	0.06207300
C	4.67526600	-1.03392400	-1.11766500
C	4.30172500	-0.58924400	1.30896000
C	5.66732000	-0.32303200	1.40236600
C	6.02309500	-0.76103000	-0.89308800
O	6.47921700	-0.23844900	0.29565700
C	3.56856700	-0.67206700	2.49629000
C	6.28635300	-0.11747800	2.63451400
C	4.30783500	-1.55944900	-2.35893400
C	6.98612200	-0.99591000	-1.87279800
C	-4.67536700	-1.03383400	1.11772200
C	-4.30154600	-0.58946600	-1.30891600
C	-5.66711700	-0.32318500	-1.40249800
C	-6.02315700	-0.76088700	0.89297100
O	-6.47911100	-0.23838200	-0.29587400
C	-3.56826600	-0.67247700	-2.49615700
C	-6.28601200	-0.11776100	-2.63473500
C	-4.30810300	-1.55928400	2.35907200
C	-6.98630500	-0.99562500	1.87259300
H	-2.51212300	-0.90146300	-2.44176400
C	-4.16920300	-0.46824200	-3.73150800
H	-3.57772700	-0.53191000	-4.63657800
H	-7.34958200	0.08424300	-2.65171400
C	-5.53371400	-0.18272400	-3.79864500
H	-6.01277100	-0.02224500	-4.75708600
H	-3.26193000	-1.77023600	2.54466000
C	-5.25500800	-1.79820000	3.34622100
H	-4.94856000	-2.20223600	4.30328800
H	-8.02114300	-0.77128300	1.64676200
C	-6.59883400	-1.51106300	3.10123700
H	-7.34503800	-1.69433600	3.86513700
H	2.51241100	-0.90102100	2.44203800
C	4.16963900	-0.46769900	3.73155100
H	3.57825300	-0.53122200	4.63669100
H	7.34993700	0.08446400	2.65135600
C	5.53417300	-0.18224200	3.79851000
H	6.01333800	-0.02166600	4.75688000
H	3.26162800	-1.77035100	-2.54438900
C	5.25461400	-1.79850500	-3.34616800
H	4.94803500	-2.20259900	-4.30316800
H	8.02099700	-0.77160900	-1.64710000
C	6.59848500	-1.51143100	-3.10135400
H	7.34459500	-1.69481600	-3.86531900

E(RB3LYP) = -2304.52694748
Zero-point correction= 0.702831 (Hartree/Particle)
Thermal correction to Energy= 0.831671
Thermal correction to Enthalpy= 0.833360
Thermal correction to Gibbs Free Energy= 0.516402
Sum of electronic and zero-point Energies
= -2303.824116
Sum of electronic and thermal Energies
= -2303.695276
Sum of electronic and thermal Enthalpies
= -2303.693588
Sum of electronic and thermal Free Energies
= -2304.010545 (unit: a.u.)

---

carbo[8]helicene

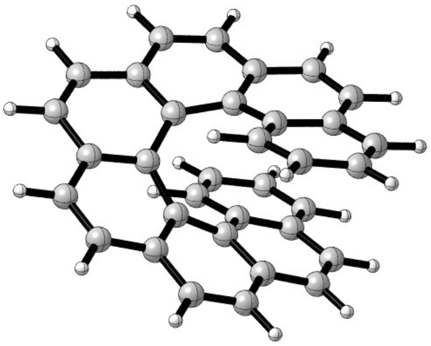

C	-3.39550500	2.38058600	0.44250000
C	-4.08007300	1.22033400	0.23620100
C	-1.98192600	2.36662400	0.62843800
C	-3.38018000	0.00100200	0.00013600
C	-1.28503500	1.13184900	0.59370600
C	-1.96333200	0.00062700	-0.00016500
C	-1.25596900	3.57494700	0.82664200
C	0.09894700	3.55021600	0.97419500
C	0.78687000	2.31488900	1.13836600
C	0.06506500	1.10094300	1.09979900
H	-3.91814300	3.32755600	0.51920300
H	-5.16342200	1.21372000	0.19178700
H	-1.79666100	4.51465600	0.80648600
H	0.66753500	4.47155200	1.03208800
C	2.19914800	2.29287700	1.35207100
C	0.71532400	-0.09093100	1.60403500
C	2.12846000	-0.08787300	1.77507800
C	2.85513200	1.12688900	1.58875800
H	2.73968400	3.23112500	1.29763900
H	3.93313700	1.11058900	1.70446400
C	-3.39692300	-2.37866200	-0.44166600
C	-4.08082000	-1.21799000	-0.23551100
C	-1.98338800	-2.36551700	-0.62801800
C	-1.28583700	-1.13109700	-0.59401500
C	-1.25814500	-3.57429900	-0.82603200
C	0.09671900	-3.55036800	-0.97424300
C	0.78523700	-2.31546500	-1.13906200
C	0.06411300	-1.10112200	-1.10046400
H	-3.92007700	-3.32538300	-0.51792200
H	-5.16414800	-1.21080300	-0.19070800
H	-1.79934300	-4.51370600	-0.80531400
H	0.66476400	-4.47203700	-1.03214700
C	2.19748500	-2.29426300	-1.35317000
C	0.71499200	0.09036900	-1.60471500
C	2.12820200	0.08674200	-1.77507700
C	2.85414600	-1.12854900	-1.58932100
H	2.73745700	-3.23286100	-1.29917100
H	3.93216300	-1.11281600	-1.70499000

C	2.78171000	1.26811400	-2.19376400
C	0.00412400	1.24113700	-2.01980700
C	2.06619500	2.39886000	-2.51025400
C	0.66008200	2.36892800	-2.45567100
H	3.86248900	1.25721500	-2.28399900
H	2.57792600	3.29788400	-2.83357100
H	0.08802000	3.23695400	-2.76077500
H	-1.07533600	1.22982400	-2.00247600
C	2.78125200	-1.26924900	2.19488300
C	0.00381700	-1.24149800	2.01870900
C	2.06514500	-2.39957400	2.51147200
C	0.65909100	-2.36932400	2.45548800
H	3.86197400	-1.25869700	2.28585400
H	2.57634100	-3.29855700	2.83575000
H	0.08653000	-3.23712400	2.76031100
H	-1.07561800	-1.23000200	2.00034500

E(RB3LYP) = -1308.17349560
Zero-point correction= 0.426811 (Hartree/Particle)
Thermal correction to Energy= 0.501228
Thermal correction to Enthalpy= 0.502916
Thermal correction to Gibbs Free Energy= 0.305336
Sum of electronic and zero-point Energies
= -1307.746684
Sum of electronic and thermal Energies
= -1307.672268
Sum of electronic and thermal Enthalpies
= -1307.670580
Sum of electronic and thermal Free Energies
= -1307.868160 (unit: a.u.)

---

carbo[8]helicene-TS1

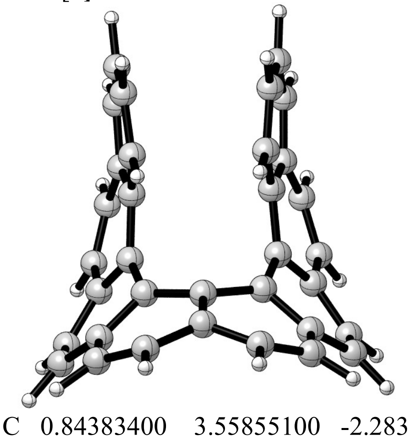

C	0.84383400	3.55855100	-2.28336300
C	1.63780600	3.81444900	-1.20970500
C	-0.02539000	2.43257900	-2.27405500
C	1.41590600	3.09275500	0.00000000
C	0.13936000	1.42882900	-1.28224500
C	0.69644300	1.86078500	0.00000000
C	-1.11981500	2.35158100	-3.18587400
C	-2.00947400	1.32060800	-3.10810800
C	-1.65438900	0.15581600	-2.36942700
C	-0.45537700	0.15231400	-1.62007400
H	0.82314600	4.22689400	-3.13684600
H	2.28086800	4.68667500	-1.18651500
H	-1.27428900	3.17397000	-3.87515900
H	-2.91393600	1.31157300	-3.70548400
C	-2.38574100	-1.06450700	-2.51970300
C	0.24515600	-1.10523400	-1.53498100
C	-0.45185000	-2.31808100	-1.80921900
C	-1.82959200	-2.25870400	-2.18075800
H	-3.37545000	-1.01878700	-2.96081500
H	-2.37849100	-3.18451600	-2.31231000
C	0.84383400	3.55855100	2.28336300
C	1.63780600	3.81444900	1.20970500
C	-0.02539000	2.43257900	2.27405500
C	0.13936000	1.42882900	1.28224500
C	-1.11981500	2.35158100	3.18587400
C	-2.00947400	1.32060800	3.10810800
C	-1.65438900	0.15581600	2.36942700
C	-0.45537700	0.15231400	1.62007400
H	0.82314600	4.22689400	3.13684600
H	2.28086800	4.68667500	1.18651500
H	-1.27428900	3.17397000	3.87515900
H	-2.91393600	1.31157300	3.70548400
C	-2.38574100	-1.06450700	2.51970300
C	0.24515600	-1.10523400	1.53498100
C	-0.45185000	-2.31808100	1.80921900
C	-1.82959200	-2.25870400	2.18075800
H	-3.37545000	-1.01878700	2.96081500
H	-2.37849100	-3.18451600	2.31231000
C	0.26562300	-3.53169400	1.86067600
C	1.65423000	-1.14993300	1.45396200
C	1.63901800	-3.54399800	1.76525100
C	2.33691900	-2.33644800	1.59561500
H	-0.28130600	-4.45185400	2.03581700
H	2.18311300	-4.47779700	1.84444100
H	2.19743000	-0.22314800	1.32209500
H	3.41995700	-2.34039600	1.55880100
C	1.65423000	-1.14993300	-1.45396200
H	2.19743000	-0.22314800	-1.32209500
C	0.26562300	-3.53169400	-1.86067600
H	-0.28130600	-4.45185400	-2.03581700
C	1.63901800	-3.54399800	-1.76525100
C	2.33691900	-2.33644800	-1.59561500
H	3.41995700	-2.34039600	-1.55880100
H	2.18311300	-4.47779700	-1.84444100

Imaginary frequency: –37.79 cm^{-1}
E(RB3LYP) = -1308.10062050
Zero-point correction= 0.424988 (Hartree/Particle)
Thermal correction to Energy= 0.498315
Thermal correction to Enthalpy= 0.500004
Thermal correction to Gibbs Free Energy= 0.305543
Sum of electronic and zero-point Energies=
-1307.675632
Sum of electronic and thermal Energies
= -1307.602305
Sum of electronic and thermal Enthalpies
= -1307.600617
Sum of electronic and thermal Free Energies
= -1307.795078 (unit: a.u.)

---

carbo[8]helicene-INT

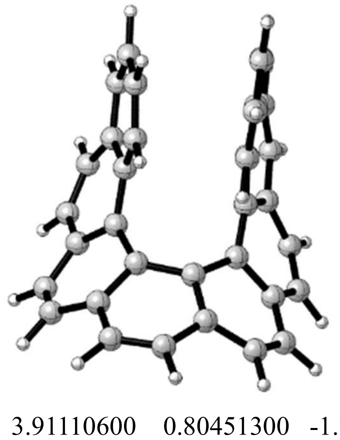

C	3.91110600	0.80451300	-1.70719400

C	3.87147800	-0.54880100	-1.82952600
C	2.95446400	1.46844300	-0.89081700
C	2.92944800	-1.28649800	-1.05814100
C	1.74504500	0.80977500	-0.53009200
C	1.76767200	-0.65335000	-0.52964900
C	3.31437500	2.70260600	-0.26998300
C	2.56435000	3.19497200	0.75522600
C	1.24718400	2.69244400	0.96213600
C	0.76300500	1.66168100	0.12510500
H	4.73260700	1.37830000	-2.12125400
H	4.65796600	-1.08473200	-2.34859000
H	4.26304800	3.15387700	-0.53709800
H	2.90698900	4.03313900	1.35123700
C	0.35700400	3.30358900	1.89989400
C	-0.64928300	1.69342000	-0.15425400
C	-1.53260500	2.35679600	0.74649300
C	-0.98227100	3.06209100	1.85871200
H	0.77506300	3.98697300	2.63097800
H	-1.65292700	3.51903000	2.57777300
C	2.81683400	-3.05300800	0.57314500
C	3.34749600	-2.56600000	-0.58064700
C	1.64131900	-2.46240800	1.12000200
C	0.95748900	-1.46756000	0.37495100
C	1.12752400	-2.85848100	2.38906900
C	-0.04879900	-2.34495200	2.85339000
C	-0.91115900	-1.66208500	1.95042000
C	-0.45241700	-1.36652200	0.64448400
H	3.25682100	-3.90504900	1.07882200
H	4.21327500	-3.03126700	-1.03755200
H	1.71610900	-3.54111500	2.99167400
H	-0.40259700	-2.56909900	3.85322300
C	-2.28020100	-1.42335800	2.28092900
C	-1.44091600	-1.31140700	-0.40810700
C	-2.81908700	-1.20957300	-0.06410500
C	-3.19443900	-1.14948600	1.31201100
H	-2.58342100	-1.51804100	3.31783900
H	-4.23685900	-0.98776000	1.56186900
C	-3.79243600	-1.26564100	-1.08409000
C	-1.10712600	-1.55853400	-1.76006100
C	-3.43635400	-1.49621200	-2.39437700
C	-2.08104700	-1.66356600	-2.72848700
H	-4.83648300	-1.16097500	-0.80976400
H	-4.19773500	-1.56676400	-3.16253500
H	-0.06798600	-1.69693700	-2.02671900
H	-1.79946800	-1.88016100	-3.75250100
C	-1.13837400	1.35178800	-1.43545000
H	-0.44314600	0.96345000	-2.16612100
C	-2.90123900	2.46987500	0.41331000
H	-3.57341100	2.93429500	1.12671800
C	-3.35353700	2.09578700	-0.83147600
C	-2.45102200	1.57931200	-1.77847700
H	-2.79347800	1.34261100	-2.77694300
H	-4.39526300	2.23232000	-1.09711000

E(RB3LYP) = -1308.10838284
Zero-point correction= 0.425601 (Hartree/Particle)
Thermal correction to Energy= 0.500284
Thermal correction to Enthalpy= 0.501972
Thermal correction to Gibbs Free Energy= 0.303129
Sum of electronic and zero-point Energies
= -1307.682782
Sum of electronic and thermal Energies
= -1307.608099
Sum of electronic and thermal Enthalpies
= -1307.606411
Sum of electronic and thermal Free Energies
= -1307.805254 (unit: a.u.)

carbo[8]helicene-TS2

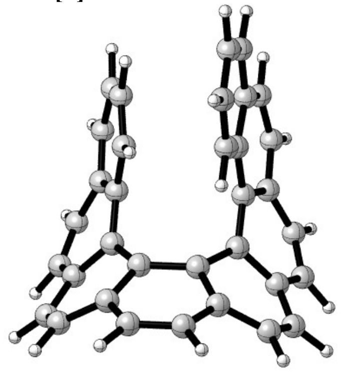

C	3.82113200	-0.53062300	-1.85064500
C	3.50157800	-1.81266000	-1.53389200
C	3.13313500	0.53726800	-1.21244700
C	2.48233500	-2.05999200	-0.57426100
C	1.85744900	0.32267100	-0.60986400
C	1.51086100	-1.06142700	-0.27524100
C	3.86465500	1.73818100	-0.96999400
C	3.43751000	2.60822400	-0.01513700
C	2.10502500	2.51085700	0.47667000
C	1.24099300	1.52049600	-0.04532200
H	4.69428300	-0.30500200	-2.45225900
H	4.11330200	-2.64106200	-1.87280200
H	4.83494600	1.85270100	-1.43919200
H	4.06295200	3.42701800	0.32185500
C	1.59491600	3.46854600	1.40958500
C	-0.15617000	1.88112300	-0.07786900
C	-0.65786900	2.85996700	0.83197600
C	0.26222100	3.56407100	1.66549000
H	2.30360500	4.12528400	1.90216900
H	-0.11809700	4.27395600	2.39144500
C	1.98048600	-3.26095100	1.45205400
C	2.62678100	-3.21286700	0.25762100
C	0.89545100	-2.36990500	1.70195200
C	0.47901500	-1.47692300	0.67826700
C	0.16359100	-2.42799900	2.92005300
C	-1.00804600	-1.74261800	3.05734400
C	-1.62918200	-1.19621600	1.90252000
C	-0.92701200	-1.16902000	0.67028400
H	2.21970300	-4.01301900	2.19506000
H	3.38385100	-3.94368500	-0.00190300
H	0.56230800	-3.02426600	3.73309800
H	-1.54518600	-1.73335100	3.99882200
C	-3.00663300	-0.82237500	1.94489300
C	-1.71295600	-1.14144100	-0.54650800
C	-3.11330500	-0.88510500	-0.46670600
C	-3.71764400	-0.64338800	0.80222700
H	-3.48465000	-0.73653900	2.91448100
H	-4.76860900	-0.37952100	0.83693900
C	-3.89286200	-0.89632500	-1.64331800
C	-1.17861800	-1.48260500	-1.81054400
C	-3.33313900	-1.18955000	-2.86606700
C	-1.96420500	-1.50330400	-2.94216500
H	-4.95250400	-0.67885900	-1.56250800
H	-3.94488500	-1.20200800	-3.76059400
H	-0.13081500	-1.73372500	-1.89082700
H	-1.52400200	-1.77410400	-3.89485000
C	-0.96296100	1.53399800	-1.18008600
H	-0.53651500	0.92345700	-1.96075900
C	-2.01033300	3.25490700	0.74330200
H	-2.39523000	3.95997100	1.47219900
C	-2.79609600	2.83900500	-0.30919300
C	-2.24462600	2.02177400	-1.31020900
H	-2.82763400	1.76309100	-2.18407700
H	-3.81967100	3.18490300	-0.39490800

Imaginary frequency: –24.71 cm^{-1}
E(RB3LYP) = -1308.10669621
Zero-point correction= 0.425558 (Hartree/Particle)
Thermal correction to Energy= 0.498724
Thermal correction to Enthalpy= 0.500413
Thermal correction to Gibbs Free Energy= 0.306635
Sum of electronic and zero-point Energies
= 1307.681138
Sum of electronic and thermal Energies
= -1307.607972
Sum of electronic and thermal Enthalpies
= -1307.606284
Sum of electronic and thermal Free Energies
= -1307.800061 (unit: a.u.)

• Cyclization of (R_a,R_a)-5 into (M)-6O

(R_a,R_a)**-5'**

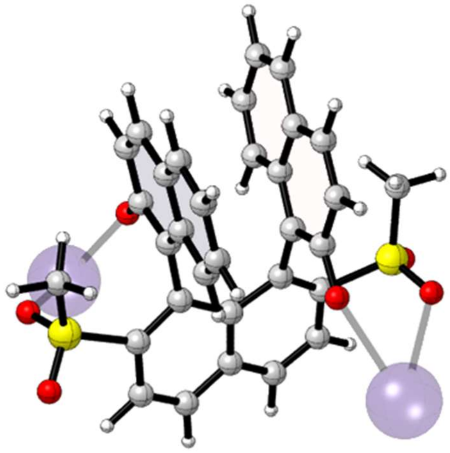

C	0.72887000	4.33363300	-2.27126600
H	1.01688900	5.37394200	-2.12735300
C	-0.07989000	3.97195400	-3.33464800
H	-0.44057700	4.72049300	-4.03566300
C	-0.43310400	2.60973800	-3.49972300
H	-1.07253600	2.31687300	-4.32994300
C	0.00530100	1.65141100	-2.60757700
H	-0.30316600	0.61922400	-2.73518200
C	0.84093500	1.98880100	-1.50256200
C	1.21248400	3.36903300	-1.35384400
C	2.09272800	3.71923700	-0.28655800
H	2.36981700	4.76568800	-0.16454700
C	2.59242800	2.76829700	0.56284100
H	3.26764400	3.04636000	1.36987000
C	2.24871700	1.36203700	0.45367800
C	1.34388500	1.02155700	-0.59457300
C	1.04355900	-0.43057800	-0.74937800
C	1.88202800	-1.20857400	-1.54242900
C	1.78745600	-2.62220200	-1.63142100
H	2.45230400	-3.17375600	-2.28671400
C	0.88324100	-3.27694000	-0.84075600
H	0.82737200	-4.36216400	-0.84755600
C	0.00014600	-2.55077500	0.00000500
C	-0.00010400	-1.11158400	-0.00005300
C	-1.04392600	-0.43087500	0.74927600
C	-1.88213900	-1.20910300	1.54241300
C	-1.78705100	-2.62267000	1.63153300
H	-2.45164400	-3.17439200	2.28694000
C	-0.88264800	-3.27716700	0.84086500
H	-0.82638800	-4.36237000	0.84779000
C	-1.34438500	1.02125600	0.59473700
C	-2.24903300	1.36210300	-0.45354000
C	-2.59280000	2.76839100	-0.56220700
H	-3.26797000	3.04671300	-1.36918400
C	-2.09316600	3.71905200	0.28753900
H	-2.37027100	4.76553900	0.16587200
C	-1.21295200	3.36850800	1.35474100
C	-0.84144400	1.98822300	1.50305000
C	-0.00577800	1.65050100	2.60793900
H	0.30267400	0.61827400	2.73525700
C	0.43270800	2.60856800	3.50033100
H	1.07218400	2.31544800	4.33042800
C	0.07951200	3.97083500	3.33566600
H	0.44025900	4.71916900	4.03687000
C	-0.72928900	4.33283300	2.27242300
H	-1.01728800	5.37318900	2.12881600
O	2.75978600	0.49687900	1.25417600
O	-2.75991600	0.49725000	-1.25449500
S	-3.40692200	-0.64873800	2.38214700
C	-3.40776200	1.07470600	2.88294000
H	-2.51354600	1.29042500	3.46681900
H	-3.49598400	1.72534900	2.01629900
H	-4.29949900	1.14345900	3.51513200
O	-4.52831100	-0.83431400	1.42213100
O	-3.47379900	-1.43583200	3.64205800
Cs	-4.44509200	-1.88918400	-1.62082400
S	3.40659300	-0.64780800	-2.38228500
O	4.52822400	-0.83343900	-1.42258100
O	3.47329600	-1.43463300	-3.64236700
C	3.40718300	1.07574600	-2.88270500
H	2.51290200	1.29148000	-3.46647400
H	3.49539200	1.72623300	-2.01594400
H	4.29885600	1.14471600	-3.51496300
Cs	4.44580700	-1.88914700	1.62016700

E(RB3LYP) = -2521.044395
Zero-point correction= 0.462143 (Hartree/Particle)
Thermal correction to Energy= 0.527039
Thermal correction to Enthalpy= 0.528316
Thermal correction to Gibbs Free Energy= 0.346211
Sum of electronic and zero-point Energies
= -2520.582252
Sum of electronic and thermal Energies
= -2520.517356
Sum of electronic and thermal Enthalpies
= -2520.516079
Sum of electronic and thermal Free Energies
= -2520.698184
E(RB3LYP/Single point) = -2521.669067
G =-2521.322856 (unit: a.u.)

TS$_{5'\rightarrow INT1}$

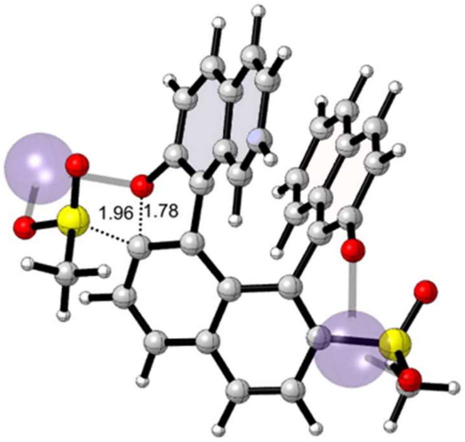

C	0.17959100	4.10763200	2.06781400
H	0.13536900	5.18167700	1.89243300
C	1.09061600	3.58368200	2.96924700
H	1.77459600	4.23410800	3.50875800
C	1.11040000	2.18414200	3.18747800
H	1.81151500	1.76092800	3.90425700
C	0.25311800	1.34644400	2.49992200
H	0.29557700	0.27792100	2.68620800
C	-0.67925100	1.84729800	1.53901900
C	-0.71642200	3.27503800	1.35498500
C	-1.67586600	3.81161200	0.44516700
H	-1.69730600	4.88965600	0.28840000
C	-2.55218800	2.99719000	-0.21870100
H	-3.28242300	3.41569700	-0.90880600
C	-2.55510400	1.55482300	-0.06461200
C	-1.55749100	1.00994800	0.79791400
C	-1.47729400	-0.46220100	0.96056200
C	-2.51548800	-1.18495200	1.54660000
C	-2.45943800	-2.58509400	1.79107400
H	-3.32373500	-3.12475000	2.16116600
C	-1.25284200	-3.22280600	1.63835100
H	-1.13986000	-4.26431900	1.93146500
C	-0.12962200	-2.53118100	1.12848700
C	-0.27060400	-1.19644600	0.58524400
C	0.82585600	-0.67806800	-0.17065500
C	2.09621500	-1.33627800	-0.10487600
C	2.26949200	-2.52307800	0.67571300
H	3.24929600	-2.98264800	0.75012700
C	1.15767500	-3.12836400	1.19980200
H	1.25334200	-4.08923000	1.70149200
C	1.11423800	0.67755500	-0.67712300
C	2.39821000	1.03754400	-0.23087400
C	2.86992600	2.36341600	-0.37657300
H	3.81207300	2.65539300	0.08031300
C	2.14939500	3.24812300	-1.15726800
H	2.51662300	4.26066200	-1.31116900
C	0.96258100	2.84157300	-1.82950000
C	0.43295500	1.52504200	-1.58622600
C	-0.68851800	1.09605800	-2.34558200
H	-1.09080600	0.10481300	-2.16539900
C	-1.26687700	1.91861500	-3.28842300
H	-2.12085300	1.56625200	-3.86180100
C	-0.77912800	3.23545400	-3.49263000
H	-1.25902900	3.88474200	-4.22091600
C	0.30809500	3.68451200	-2.76939000
H	0.69920900	4.68796200	-2.92730000
O	-3.40852500	0.83815700	-0.69945300
O	3.14271000	0.04964200	0.29420200
S	3.04853100	-1.61589900	-1.79415200
C	1.98324900	-2.85645800	-2.56264800
H	1.90232400	-3.72550100	-1.90757400
H	1.00153300	-2.40934200	-2.73703000
H	2.45565700	-3.12801000	-3.51094300
O	3.11573700	-0.46435100	-2.73463700
O	4.35361300	-2.27832600	-1.46785000
Cs	6.17984200	-0.67239500	0.60843700
S	-4.01861200	-0.45322700	2.26093000
O	-3.99973800	1.01794800	2.37087100
O	-5.18555800	-1.09373800	1.58898600
C	-3.98823900	-1.05559300	3.96449200
H	-4.08738400	-2.14033800	3.99863100
H	-3.05292400	-0.73424100	4.42903300
H	-4.84107800	-0.57938000	4.45728900
Cs	-5.15183800	-1.30400100	-1.61543300

Imaginary frequency: –389.85 cm^{-1}
E(RB3LYP) = -2520.990202
Zero-point correction= 0.459066 (Hartree/Particle)
Thermal correction to Energy= 0.523848
Thermal correction to Enthalpy= 0.525125
Thermal correction to Gibbs Free Energy= 0.341587
Sum of electronic and zero-point Energies= -2520.531136
Sum of electronic and thermal Energies= -2520.466354
Sum of electronic and thermal Enthalpies= -2520.465077
Sum of electronic and thermal Free Energies= -2520.648615E(RB3LYP/Single point) = -2521.612753
G = -2521.271166 (unit: a.u.)

(*M*, *R$_a$*)-**INT1**

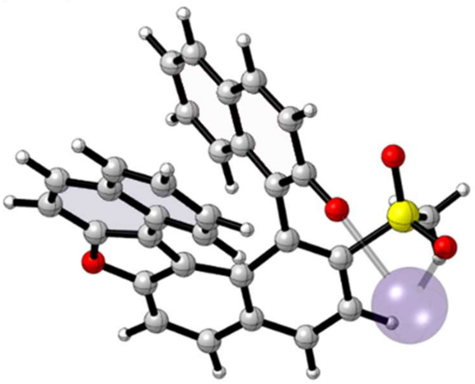

C	-3.18182600	-3.79077500	-0.43446500
H	-4.02694000	-4.45247700	-0.25887200
C	-1.89400600	-4.29024800	-0.46761800
H	-1.71409400	-5.34879900	-0.29816800
C	-0.81174100	-3.42701700	-0.75242700
H	0.19791400	-3.82416900	-0.80933800
C	-1.02163500	-2.07364000	-0.93665100
H	-0.18057000	-1.42583700	-1.14584200
C	-2.32364200	-1.52513500	-0.87095300
C	-3.43727800	-2.41468600	-0.67767600
C	-4.77885900	-1.94039700	-0.82167300
H	-5.59563300	-2.63786300	-0.65566200
C	-5.04332700	-0.65613000	-1.24527900
H	-6.05222400	-0.30677300	-1.44165900
C	-3.93727400	0.18973800	-1.42823400
C	-2.61383200	-0.14517200	-1.12832900
C	-1.83663800	1.05861900	-1.38942400
C	-2.74286500	1.93832700	-1.99905500
C	-2.39576400	3.12866900	-2.65342300
H	-3.14626400	3.73502800	-3.15074000
C	-1.05532500	3.44166900	-2.67902900

H	-0.70374600	4.31061400	-3.22818300
C	-0.10593500	2.69153600	-1.91974600
C	-0.50620500	1.55440600	-1.13205400
C	0.33937400	1.12837500	-0.03664800
C	1.62090800	1.68649200	0.02463100
C	2.11027900	2.59196200	-0.95015900
H	3.13977900	2.93195300	-0.92200300
C	1.24520100	3.11949300	-1.87661000
H	1.57469200	3.90168700	-2.55524500
C	-0.17704300	0.21408500	1.00573100
C	0.54518300	-0.99134000	1.27081600
C	-0.03151900	-1.89079300	2.25094000
H	0.49998500	-2.82345100	2.42894500
C	-1.20173900	-1.61833900	2.90224100
H	-1.61698900	-2.33154400	3.61336400
C	-1.90446100	-0.39499500	2.67825200
C	-1.35629200	0.54629100	1.73838100
C	-2.03115400	1.80087900	1.62095900
H	-1.62094300	2.56581100	0.97033700
C	-3.19445200	2.07019700	2.31747600
H	-3.67944600	3.03615900	2.19285900
C	-3.75613000	1.11147800	3.19455600
H	-4.67557100	1.32949800	3.73176400
C	-3.10070900	-0.09440900	3.37387100
H	-3.49504700	-0.83755700	4.06517200
O	-4.02554800	1.44974700	-1.96708700
O	1.61991500	-1.31505900	0.65468200
S	2.75536200	1.53360200	1.43701800
C	3.08683600	3.25822800	1.86061400
H	3.62796000	3.76036900	1.05876200
H	2.13573800	3.75627500	2.06386600
H	3.69862400	3.22225200	2.76679600
O	2.13399100	0.95098300	2.64118400
O	4.03209300	0.95253000	0.93789200
Cs	4.00753800	-1.58306600	-0.99247100

E(RB3LYP) = -1912.261639
Zero-point correction= 0.415312 (Hartree/Particle)
Thermal correction to Energy= 0.467359
Thermal correction to Enthalpy= 0.468636
Thermal correction to Gibbs Free Energy= 0.317677
Sum of electronic and zero-point Energies
= -1911.846328
Sum of electronic and thermal Energies
= -1911.794280
Sum of electronic and thermal Enthalpies
= -1911.793004
Sum of electronic and thermal Free Energies
= -1911.943962
E(RB3LYP/Single point) = -1912.759717
G = -1912.44204 (unit: a.u.)

$TS_{INT1\rightarrow 6O}$

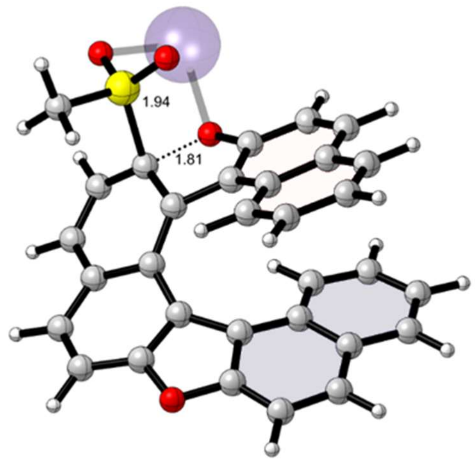

C	-4.43935700	-2.68880200	-1.43173400
H	-5.36736200	-3.25110800	-1.35462500
C	-3.30146000	-3.28201400	-1.94144700
H	-3.32066400	-4.32393800	-2.25073900
C	-2.11695000	-2.52285100	-2.09515600
H	-1.23849600	-2.97717700	-2.54449700
C	-2.07011400	-1.20762200	-1.67517700
H	-1.16425600	-0.62893500	-1.80404600
C	-3.20365800	-0.58875000	-1.09238000
C	-4.43989900	-1.32197100	-1.03803500
C	-5.65313000	-0.66628600	-0.66650300
H	-6.56876400	-1.25067000	-0.62990100
C	-5.69293300	0.69657600	-0.43750900
H	-6.62292900	1.22003600	-0.23684000
C	-4.47152000	1.38184600	-0.47692900
C	-3.22387700	0.77195600	-0.64897400
C	-2.24262200	1.82920500	-0.48164600
C	-2.99352400	3.01344700	-0.42520300
C	-2.46532800	4.30775600	-0.55298800
H	-3.10319500	5.18547100	-0.54763700
C	-1.10041600	4.37521200	-0.77975900
H	-0.63333700	5.33359700	-0.99526700
C	-0.26644800	3.22744500	-0.69880500
C	-0.80976000	1.93222200	-0.33468000
C	0.07735200	0.95124500	0.16816500
C	1.49243000	1.15377500	0.06125200
C	1.99753700	2.28854400	-0.65141700
H	3.06597300	2.39882200	-0.80511600
C	1.13196800	3.31832500	-0.93965100
H	1.52900100	4.25087300	-1.33593600
C	-0.14360800	-0.45228500	0.52617400
C	0.93227100	-1.20804900	0.01695700
C	0.88362700	-2.62003300	0.02060000
H	1.68330200	-3.18844700	-0.44729400
C	-0.17158900	-3.25750800	0.64966900
H	-0.21057900	-4.34440300	0.67395900
C	-1.20002600	-2.52605000	1.30359700
C	-1.17747100	-1.08732600	1.25948400
C	-2.17321700	-0.37215500	1.98178400
H	-2.13535600	0.71208200	1.99371300
C	-3.17296200	-1.03698700	2.66207600
H	-3.92565800	-0.47057900	3.20516700
C	-3.22985200	-2.45468500	2.65640500
H	-4.03238300	-2.96647600	3.18138200
C	-2.25651400	-3.17786400	1.99633200
H	-2.27922700	-4.26588800	2.00215300
O	-4.34289200	2.74629000	-0.35159500
O	1.97446000	-0.51355400	-0.46307900

S 2.54649400 0.95149800 1.67429900
C 2.10104800 2.46579400 2.55259400
H 2.39557600 3.33334200 1.96065700
H 1.02236000 2.46179300 2.72713900
H 2.64261700 2.44230500 3.50243500
O 2.16632100 -0.18484400 2.55468400
O 3.99156500 1.05485700 1.29702800
Cs 5.04734300 -0.85762400 -0.98731600

Imaginary frequency: –388.69 cm^{-1}
E(RB3LYP) = -1912.22275
Zero-point correction= 0.413143 (Hartree/Particle)
Thermal correction to Energy= 0.464538
Thermal correction to Enthalpy= 0.465815
Thermal correction to Gibbs Free Energy= 0.317362
Sum of electronic and zero-point Energies
= -1911.809608
Sum of electronic and thermal Energies
= -1911.758213
Sum of electronic and thermal Enthalpies
= -1911.756936
Sum of electronic and thermal Free Energies
= -1911.905388
E(RB3LYP/Single point) = -1912.717925
G = -1912.400563 (unit: a.u.)

• Stereo-inversion of (*M*, *R*$_a$)-INT1

TS$_A$

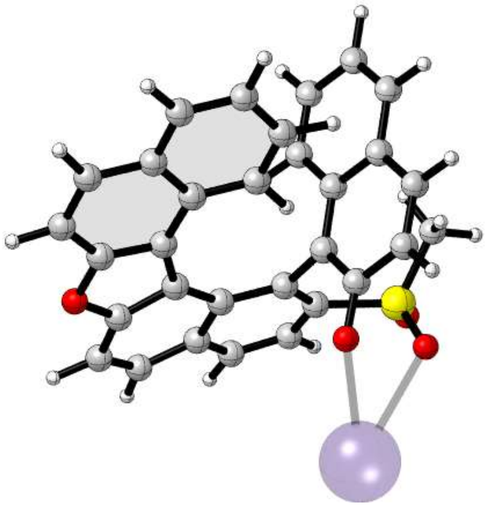

C -5.60520000 -0.27642700 -1.45693400
H -6.65570300 -0.06896800 -1.64649400
C -5.05125500 -1.48459200 -1.82175400
H -5.65602400 -2.25602300 -2.29099500
C -3.67640800 -1.68479900 -1.60043100
H -3.20580500 -2.60886300 -1.91754100
C -2.90549200 -0.71233500 -0.98704500
H -1.85842800 -0.87610900 -0.85244800
C -3.42831300 0.52011200 -0.54658100
C -4.82867900 0.74576500 -0.85087800
C -5.46355900 1.99646000 -0.60576800
H -6.51292500 2.11439500 -0.86065500
C -4.74083900 3.03011400 -0.08495100
H -5.15914900 4.01379500 0.10343100
C -3.40172300 2.77059200 0.24928300
C -2.66760700 1.57970500 0.12068300
C -1.33061200 1.87973700 0.78715400
C -1.49311400 3.23017000 1.17617700
C -0.64746800 4.06659300 1.91329700
H -0.93685900 5.09391700 2.10852500
C 0.48251800 3.49420200 2.39899700
H 1.17399700 4.05089200 3.02327700
C 0.78593100 2.13615900 2.09179000
C -0.03043900 1.28396100 1.22696300
C 0.57405300 -0.00017200 0.91287700
C 1.80564200 -0.34890200 1.49682800
C 2.49567900 0.44510000 2.42669500
H 3.41926200 0.10251200 2.87448700
C 1.98050700 1.68094700 2.69572100
H 2.50066800 2.35379100 3.37139000
C 0.04180900 -1.04974000 0.00509000
C 0.37119200 -0.97918300 -1.38305900
C 0.04337300 -2.16029500 -2.16618800
H 0.32180900 -2.14467300 -3.21821800
C -0.59800500 -3.24643000 -1.63274000
H -0.83353700 -4.10562300 -2.25930200
C -0.99307700 -3.28064500 -0.26095600
C -0.65486900 -2.15699400 0.56779100
C -1.06050800 -2.20276900 1.93425500
H -0.82976500 -1.36302600 2.58348800
C -1.74438500 -3.29076600 2.44487400
H -2.04284700 -3.29339000 3.49086700
C -2.06386600 -4.39898800 1.62327700
H -2.60107500 -5.24889200 2.03588000
C -1.68821600 -4.38199100 0.29078700
H -1.92815700 -5.22145800 -0.35960800
O -2.69236700 3.76104900 0.83686200
O 0.91874900 0.04042900 -1.93012800
S 2.88358400 -1.73726000 0.94236900
C 2.08025500 -3.34278300 0.89993100
H 1.46459800 -3.47079500 1.79115900
H 1.50170900 -3.45328600 -0.01232200
H 2.91869600 -4.04622700 0.91316700
O 3.36493900 -1.42198000 -0.42706400
O 3.92704700 -1.86544600 1.99545900
Cs 3.54747800 1.47165400 -1.79905700

Imaginary frequency: –37.71 cm^{-1}
E(RB3LYP) = -1912.236333
Zero-point correction= 0.415625 (Hartree/Particle)
Thermal correction to Energy= 0.466546
Thermal correction to Enthalpy= 0.467823
Thermal correction to Gibbs Free Energy= 0.319425
Sum of electronic and zero-point Energies
= -1911.820708
Sum of electronic and thermal Energies=
-1911.769786
Sum of electronic and thermal Enthalpies
= -1911.768510
Sum of electronic and thermal Free Energies
= -1911.916908
E(RB3LYP/Single point) = -1912.733841
G = -1912.414416 (unit: a.u.)

(*P*, *R*)-**INT2**

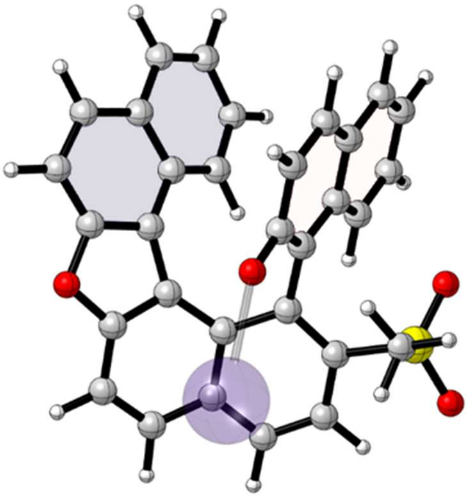

C	4.79811500	-2.21802800	-0.42065500
H	5.46837500	-2.84780600	-1.00129000
C	5.27448700	-1.09278200	0.22377900
H	6.32132400	-0.81388000	0.13584900
C	4.40594400	-0.32497900	1.03232000
H	4.79012000	0.53188800	1.57707100
C	3.06461900	-0.64363200	1.11921900
H	2.40613400	-0.05671400	1.74570600
C	2.53500500	-1.76105900	0.43342800
C	3.43943800	-2.61244500	-0.29152500
C	3.00492900	-3.88611500	-0.77521300
H	3.71019600	-4.49854300	-1.33080100
C	1.75615700	-4.37884600	-0.46440900
H	1.44466900	-5.38219300	-0.73809200
C	0.89593700	-3.52299900	0.24318900
C	1.17290400	-2.19022900	0.56282400
C	-0.04080300	-1.67391100	1.18432400
C	-0.84800400	-2.80825000	1.36105600
C	-2.01324300	-2.86298500	2.13913900
H	-2.54629800	-3.79741400	2.28478300
C	-2.40505200	-1.68600100	2.73366900
H	-3.26788700	-1.66232800	3.39338600
C	-1.76377800	-0.44917300	2.41193400
C	-0.62351400	-0.39479500	1.52836000
C	-0.31158200	0.85918200	0.87778800
C	-1.06441800	1.97916000	1.25258100
C	-2.01455000	1.95829000	2.30059800
H	-2.52619100	2.87119900	2.57982000
C	-2.35827200	0.75555700	2.86590800
H	-3.15430100	0.70311000	3.60423800
C	0.63194700	0.91151700	-0.27131400
C	0.26740400	0.19100400	-1.45112500
C	1.21587800	0.22208700	-2.54780200
H	0.95428700	-0.34773500	-3.43753500
C	2.38059000	0.93917700	-2.49244300
H	3.06572800	0.94254300	-3.33943900
C	2.72625100	1.69819000	-1.33582700
C	1.83353600	1.67907700	-0.21206200
C	2.23027600	2.41900400	0.94160900
H	1.58458500	2.41928100	1.81396300
C	3.41192500	3.12881000	0.97823600
H	3.67965700	3.67845700	1.87833600
C	4.28133400	3.15105200	-0.14107400
H	5.21112900	3.71301900	-0.10284000
C	3.93239900	2.44030100	-1.27439300
H	4.58834200	2.43166700	-2.14345000
O	-0.32243600	-3.91337100	0.73955700
O	-0.85319600	-0.42573200	-1.58466300
S	-1.21376800	3.45763900	0.19457600
C	-2.07259400	2.80134200	-1.24440700
H	-1.48451700	1.99492300	-1.68396000
H	-3.05010400	2.44117700	-0.91655100
H	-2.18506800	3.63456900	-1.94477800
O	-2.15174000	4.38017300	0.88814400
O	0.07260600	4.03546500	-0.24391300
Cs-	3.74566100	-0.90281500	-1.29976200

E(RB3LYP) = -1912.261631
Zero-point correction= 0.415413 (Hartree/Particle)
Thermal correction to Energy= 0.467295
Thermal correction to Enthalpy= 0.468572
Thermal correction to Gibbs Free Energy= 0.319411
Sum of electronic and zero-point Energies
= -1911.846218
Sum of electronic and thermal Energies
= -1911.794336
Sum of electronic and thermal Enthalpies
= -1911.793059
Sum of electronic and thermal Free Energies
= -1911.942220
E(RB3LYP/Single point) = -1912.759795
G = -1912.440384 (unit: a.u.)

TS$_B$

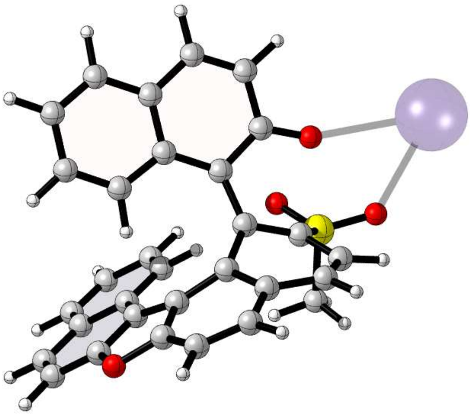

C	4.61152800	0.51408400	3.18617900
H	5.57097500	0.64068700	3.68256900
C	3.44636400	0.93383200	3.79695100
H	3.47906100	1.39747400	4.77938500
C	2.20814000	0.75519600	3.13865600
H	1.28497200	1.07693200	3.61221700
C	2.15510700	0.17183300	1.88726400
H	1.19726800	0.05605200	1.40342900
C	3.32999800	-0.26874900	1.23062000
C	4.59142700	-0.09691800	1.90356100
C	5.80768600	-0.54435900	1.30179000
H	6.73879800	-0.39261900	1.84122800
C	5.81354700	-1.17264800	0.07676000
H	6.72663800	-1.53725100	-0.38352300
C	4.57324500	-1.33598100	-0.56022600
C	3.34555700	-0.88934500	-0.06379100
C	2.35012600	-1.26569700	-1.07608200
C	3.08287200	-1.99367100	-2.03133300
C	2.52712500	-2.74695100	-3.06943900
H	3.15123700	-3.27519100	-3.78313500
C	1.15394000	-2.87277400	-3.04739100
H	0.65835900	-3.55083000	-3.73738000
C	0.34923300	-2.19290500	-2.08547900
C	0.92348700	-1.18341400	-1.22506800
C	-0.03510000	-0.31732500	-0.50918500

C	-1.12609700	-1.09973400	-0.01588100
C	-1.62723200	-2.21576500	-0.71943800
H	-2.53734000	-2.69818400	-0.37373900
C	-0.99200700	-2.62639900	-1.87155300
H	-1.42197300	-3.38319800	-2.52141700
C	-0.16618100	1.13900200	-0.52475100
C	-1.55028000	1.60590200	-0.41452400
C	-1.87481400	2.96405200	-0.80402200
H	-2.92788600	3.22986800	-0.75925900
C	-0.92649500	3.87715800	-1.13218900
H	-1.19421400	4.90675200	-1.36191200
C	0.44821900	3.49471400	-1.19697800
C	0.82955600	2.13140700	-0.92765200
C	2.20871800	1.86043800	-1.09394000
H	2.57276000	0.87149500	-0.92492700
C	3.14012600	2.81934300	-1.46231500
H	4.18299300	2.52648700	-1.56155600
C	2.74983700	4.14916600	-1.69769500
H	3.47745500	4.90516200	-1.98034000
C	1.40892400	4.46535100	-1.56483700
H	1.06114400	5.47950600	-1.75068800
O	4.42982600	-1.98940100	-1.75537200
O	-2.52362600	0.88113100	-0.03691500
S	-1.69295500	-0.94289800	1.68716400
C	-0.81747700	-2.33323900	2.43857200
H	0.25699000	-2.17832100	2.31708400
H	-1.08563700	-2.33892000	3.49943800
H	-1.13151800	-3.26242200	1.95840600
O	-1.17223600	0.27186300	2.35417300
O	-3.14225400	-1.26763100	1.80660700
Cs	-5.36983500	-0.08924400	-0.01996300

Imaginary frequency: –24.39 cm^{-1}
E(RB3LYP) = -1912.218765 Zero-point correction= 0.414178 (Hartree/Particle)
Thermal correction to Energy= 0.465266
Thermal correction to Enthalpy= 0.466542
Thermal correction to Gibbs Free Energy= 0.319219
Sum of electronic and zero-point Energies
= -1911.804587
Sum of electronic and thermal Energies
= -1911.753499
Sum of electronic and thermal Enthalpies
= -1911.752223
Sum of electronic and thermal Free Energies
= -1911.899546
E(RB3LYP/Single point) = -1912.715892
G = -1912.396673 (unit: a.u.)

(*P*)-**INT3**

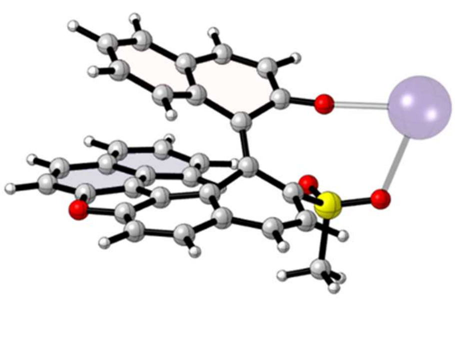

C	-4.25358500	-2.56675000	1.78482600
H	-5.18809300	-3.12035900	1.84190500
C	-3.08833600	-3.10387100	2.29536000
H	-3.09205600	-4.09346500	2.74501700
C	-1.89459500	-2.34765600	2.25758400
H	-0.98232100	-2.74806000	2.69070900
C	-1.87065100	-1.09647100	1.67174800
H	-0.94626800	-0.53987500	1.67218100
C	-3.03386000	-0.54151500	1.08179400
C	-4.26860300	-1.27530100	1.19270100
C	-5.50120700	-0.70224900	0.75256900
H	-6.41109500	-1.28695800	0.85928300
C	-5.55621100	0.57414400	0.23824600
H	-6.48995000	1.03801400	-0.06445500
C	-4.33698900	1.25539900	0.10333600
C	-3.07996600	0.73297900	0.42541500
C	-2.11771000	1.75261800	0.01234300
C	-2.90487600	2.83432000	-0.41673800
C	-2.41148200	4.08560100	-0.80193000
H	-3.07913000	4.87865000	-1.12420100
C	-1.04908200	4.26777200	-0.68705100
H	-0.61079200	5.24205400	-0.88709000
C	-0.17964300	3.20769800	-0.28842600
C	-0.69522000	1.87767200	-0.09245900
C	0.26746400	0.77155200	-0.03236000
C	1.45144300	1.16457200	0.68681600
C	1.93007100	2.48943700	0.61361500
H	2.89714400	2.72941700	1.04849400
C	1.19280800	3.47128700	-0.01836700
H	1.59647500	4.46586000	-0.18362000
C	0.05129000	-0.50373600	-0.63297900
C	1.05654800	-1.56258400	-0.52180900
C	0.58136800	-2.93678400	-0.64973200
H	1.27362200	-3.71755000	-0.34377600
C	-0.66028100	-3.22850600	-1.11696400
H	-1.00740100	-4.25826200	-1.18022300
C	-1.49882000	-2.18800500	-1.64992000
C	-1.09379700	-0.82426900	-1.49418600
C	-1.77871200	0.12536400	-2.29258200
H	-1.42652200	1.14766000	-2.32842700
C	-2.88441800	-0.21841000	-3.06008900
H	-3.37793700	0.54848000	-3.65253600
C	-3.36260300	-1.54088600	-3.08081900
H	-4.24625200	-1.80148900	-3.65731000
C	-2.64877800	-2.51608300	-2.39827700
H	-2.94689300	-3.56093300	-2.45725100
O	-4.24438700	2.52985900	-0.39399500
O	2.28474500	-1.35228600	-0.32973500
S	2.19952900	0.19200500	1.99936200
C	1.95111900	1.27687200	3.42579100
H	2.51722300	2.20119600	3.30293000
H	0.88366100	1.48511700	3.52740900
H	2.31714600	0.71790800	4.29251300
O	1.40262500	-1.02079100	2.29679500
O	3.67753300	0.07358600	1.84683800
Cs	5.05975900	-0.37541200	-0.89627800

E(RB3LYP) = -1912.231396
Zero-point correction= 0.414415 (Hartree/Particle)
Thermal correction to Energy= 0.466536
Thermal correction to Enthalpy= 0.467813
Thermal correction to Gibbs Free Energy= 0.317295
Sum of electronic and zero-point Energies
= -1911.816981
Sum of electronic and thermal Energies
= -1911.764860
Sum of electronic and thermal Enthalpies

= -1911.763584
Sum of electronic and thermal Free Energies
= -1911.914102
E(RB3LYP/Single point) = -1912.728753
G = -1912.411458 (unit: a.u.)

TSc

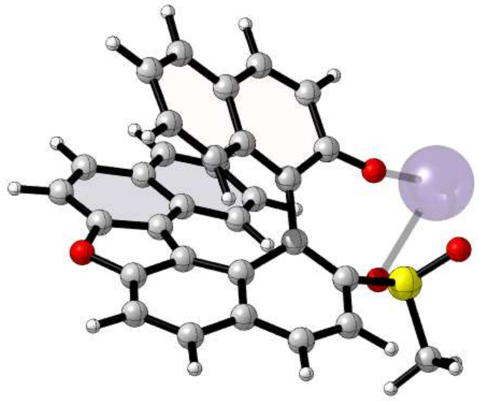

C	0.96415500	-3.92500900	-1.77349700
H	1.30877300	-4.94629400	-1.91890600
C	-0.38351500	-3.62625000	-1.81896300
H	-1.11369300	-4.41410300	-1.98557200
C	-0.80953500	-2.28608500	-1.67710300
H	-1.86554700	-2.04315600	-1.75295200
C	0.10288200	-1.27618300	-1.43584200
H	-0.25337600	-0.26003600	-1.33586000
C	1.49102700	-1.55450800	-1.34608400
C	1.93342900	-2.90545800	-1.57397200
C	3.32760300	-3.21006800	-1.66538300
H	3.62062900	-4.24297100	-1.83402800
C	4.28689700	-2.22287800	-1.60352300
H	5.34427900	-2.43445300	-1.72891800
C	3.83446000	-0.91566400	-1.36415900
C	2.50197500	-0.55956000	-1.13658900
C	2.52966700	0.86530000	-0.83531700
C	3.85956700	1.25592200	-1.04026600
C	4.32086100	2.57732700	-0.98169500
H	5.36444000	2.81752100	-1.15968600
C	3.37233200	3.54890700	-0.73662000
H	3.65152100	4.59911800	-0.74477300
C	2.01634000	3.21267400	-0.44055700
C	1.60709800	1.84249800	-0.34591000
C	0.35458400	1.49234800	0.32540300
C	-0.66293100	2.48250400	0.10867900
C	-0.27431300	3.83621700	-0.06900300
H	-1.02903100	4.61179800	-0.05474600
C	1.04162200	4.21579500	-0.21078000
H	1.31992000	5.26533000	-0.23543000
C	0.37798700	0.30749600	1.16526700
C	-0.81053400	-0.45750700	1.45820800
C	-0.64254900	-1.80405500	1.99366600
H	-1.54463600	-2.40793300	2.06511000
C	0.57158400	-2.30090900	2.34470900
H	0.67580300	-3.32284100	2.70563400
C	1.73480300	-1.46133600	2.32107700
C	1.62313200	-0.12994700	1.80207500
C	2.74101900	0.71867800	2.03903100
H	2.68121500	1.76732400	1.77600200
C	3.91496700	0.25712300	2.61226000
H	4.73761600	0.95178800	2.76576800
C	4.04813100	-1.09064700	3.00382100
H	4.97834400	-1.45482800	3.43185400
C	2.95037700	-1.92478000	2.87596700
H	2.99508700	-2.95420000	3.22638700
O	4.66436700	0.17862900	-1.32804500
O	-1.97765200	-0.03775600	1.22098100
S	-2.43677700	2.22125100	-0.21300100
C	-2.89613200	3.63846800	-1.25326000
H	-2.98209100	4.55737800	-0.67269100
H	-2.19831200	3.74857200	-2.08500700
H	-3.88289200	3.35361300	-1.62976200
O	-2.58726100	1.07181900	-1.14869100
O	-3.33797400	2.33530100	0.96325000
Cs	-4.56497100	-1.06760200	0.04483300

Imaginary frequency: –45.54 cm^{-1}
E(RB3LYP) = -1912.228993
Zero-point correction= 0.415232 (Hartree/Particle)
Thermal correction to Energy= 0.465797
Thermal correction to Enthalpy= 0.467074
Thermal correction to Gibbs Free Energy= 0.322899
Sum of electronic and zero-point Energies
= -1911.813761
Sum of electronic and thermal Energies
= -1911.763196
Sum of electronic and thermal Enthalpies
= -1911.761920
Sum of electronic and thermal Free Energies
= -1911.906095
E(RB3LYP/Single point) = -1912.725761
G = -1912.402862 (unit: a.u.)

TS$_{5'\rightarrow INT4}$

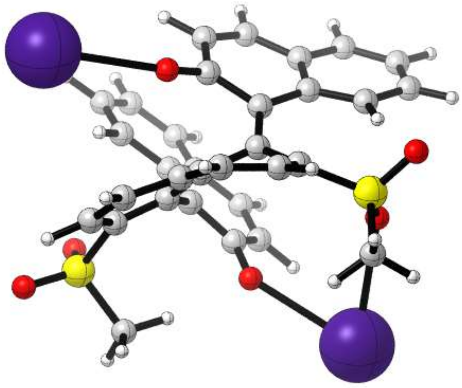

C	-1.04188100	4.95910100	1.16904300
H	-0.57847800	5.64126600	1.87911600
C	-2.36080000	5.14012900	0.77865200
H	-2.96116300	5.94455000	1.19527400
C	-2.87745600	4.29066700	-0.21323200
H	-3.87813000	4.45208700	-0.60782300
C	-2.11823300	3.24066200	-0.71425000
H	-2.55178400	2.66950000	-1.50772900
C	-0.80649500	2.95641100	-0.27462000
C	-0.24880600	3.92352700	0.63092700
C	1.15737100	3.90193900	0.91281400
H	1.56226300	4.64730500	1.59470600
C	1.96762500	3.02573100	0.27454300
H	3.04382500	3.04610800	0.41024700
C	1.45937900	1.96496900	-0.58609300
C	-0.00427300	1.81190400	-0.71689400
C	-0.42812700	0.61616500	-1.40409100
C	-1.44308400	0.36382800	-2.39862700
C	-1.07689400	-0.37874200	-3.55817900
H	-1.74083600	-0.35911400	-4.41738400
C	0.08359500	-1.10648400	-3.63087000

H	0.37300300	-1.62243500	-4.54143600
C	0.77035300	-1.35641100	-2.40871100
C	0.44090400	-0.58235500	-1.25211900
C	0.83154500	-1.06627500	0.04562600
C	1.61803400	-2.22708500	0.09068900
C	2.10299400	-2.87257800	-1.07066600
H	2.74129600	-3.74302900	-0.97399300
C	1.68409200	-2.43309400	-2.30498400
H	1.98397600	-2.95978000	-3.20786900
C	0.32615800	-0.42059300	1.29693200
C	-1.04329600	-0.62477200	1.63518600
C	-1.55260500	0.13723500	2.76110200
H	-2.61136900	0.02646600	2.99091200
C	-0.75990300	0.96137400	3.51548000
H	-1.18150300	1.52361900	4.34805700
C	0.63381400	1.08536200	3.24336900
C	1.17971600	0.36114800	2.12745300
C	2.58568900	0.46685300	1.91737600
H	3.02768600	-0.07761900	1.09236600
C	3.38953200	1.23709000	2.73130100
H	4.45890400	1.28841000	2.53515300
C	2.83698900	1.97745500	3.80563600
H	3.47423500	2.59658300	4.43230400
C	1.47728600	1.89363600	4.04695700
H	1.02947200	2.44458300	4.87272700
O	2.31318400	1.27066000	-1.20865500
O	-1.81283400	-1.45283100	1.01694300
S	1.82651500	-3.19829100	1.60705900
C	0.18174000	-3.88279600	1.85564000
H	-0.55835700	-3.07700400	1.82602300
H	-0.00641400	-4.59401800	1.04724400
H	0.19241700	-4.39303900	2.82363000
O	2.74011200	-4.32605200	1.27342700
O	2.19030800	-2.39278000	2.79295100
Cs	5.01737400	0.03559300	-1.58178500
S	-3.21552700	0.64110800	-2.34723000
O	-3.67989100	0.72194900	-0.93924200
O	-3.65587800	1.72399800	-3.27101500
C	-3.91895100	-0.89518600	-2.99538300
H	-3.76412400	-0.97385600	-4.07133500
H	-3.47862000	-1.74708400	-2.47395500
H	-4.98922700	-0.81925300	-2.78063300
Cs	-4.77008200	-1.71144300	0.71272800

E(RB3LYP) = -2520.980692
Zero-point correction= 0.460068 (Hartree/Particle)
Thermal correction to Energy= 0.524424
Thermal correction to Enthalpy= 0.525701
Thermal correction to Gibbs Free Energy= 0.344968
Sum of electronic and zero-point Energies
= -2520.520624
Sum of electronic and thermal Energies
= -2520.456268
Sum of electronic and thermal Enthalpies
= -2520.454991
Sum of electronic and thermal Free Energies
= -2520.635724
E(RB3LYP/Single point) = -2521.607367
G = -2521.262399 (unit: a.u.)

(R_a)-**INT4**

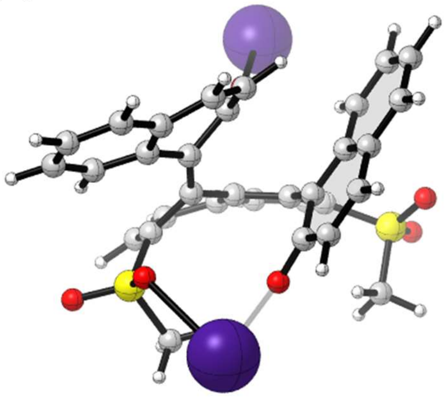

C	-2.64171100	3.27398400	3.07600200
H	-2.78175600	3.20201100	4.15265200
C	-3.43014900	4.13341500	2.32062500
H	-4.21272800	4.72251300	2.79163300
C	-3.16850800	4.25910700	0.94583300
H	-3.73290100	4.96928200	0.34603900
C	-2.19693300	3.47466800	0.33566700
H	-2.02492600	3.59204200	-0.72676100
C	-1.41974500	2.54950900	1.06427700
C	-1.61142600	2.51909300	2.47845900
C	-0.67265800	1.79366500	3.30820100
H	-0.88707200	1.70986800	4.37222400
C	0.50009800	1.34180400	2.80122600
H	1.27197200	0.90733800	3.42853600
C	0.80300300	1.50570700	1.37863700
C	-0.35194700	1.71653000	0.47564300
C	-0.29467700	1.25840300	-0.83879100
C	-1.20914300	1.63465800	-1.90795700
C	-0.66780900	1.96371300	-3.17156900
H	-1.32111500	2.41656400	-3.91383000
C	0.65169900	1.69627900	-3.47399600
H	1.08938500	1.99721500	-4.42129300
C	1.32480500	0.73825900	-2.65158500
C	0.79072000	0.38157200	-1.36610800
C	1.19013500	-0.85296800	-0.75639000
C	2.14744300	-1.62990200	-1.44125400
C	2.81815100	-1.16996600	-2.59430900
H	3.58122500	-1.78763100	-3.05330000
C	2.42634900	0.01601300	-3.17366800
H	2.88083800	0.34639300	-4.10480400
C	0.60711400	-1.36353400	0.52244600
C	-0.75644700	-1.79081900	0.53459100
C	-1.26290600	-2.28996000	1.80274000
H	-2.29877900	-2.62115200	1.82662200
C	-0.49413800	-2.37740200	2.93115700
H	-0.91859500	-2.75960200	3.85898900
C	0.87524400	-1.98765200	2.91582100
C	1.42944200	-1.49011300	1.68728900
C	2.80959200	-1.13034900	1.71333300
H	3.26923200	-0.76557100	0.80445300
C	3.57161400	-1.23293300	2.85814300
H	4.62021900	-0.94216000	2.83029700
C	3.00944000	-1.71366500	4.06805000
H	3.61762600	-1.78949500	4.96607500
C	1.67877400	-2.08706800	4.08052700
H	1.21930200	-2.46561600	4.99240900
O	1.98711900	1.55174400	0.98620600
O	-1.52102000	-1.79072400	-0.50152100
S	2.35399500	-3.40530200	-1.13931100
C	0.75722000	-4.06274400	-1.64861700
H	-0.03838600	-3.57506600	-1.08123500
H	0.63783200	-3.86376100	-2.71654000
H	0.78363900	-5.13977900	-1.45642700

O	3.36724200	-3.89065700	-2.11695900
O	2.58461600	-3.77962700	0.27316100
Cs	4.67871600	2.03962900	-0.28845300
S	-2.94118800	1.31366800	-1.93610400
O	-3.49247100	1.02329600	-0.58850400
O	-3.62860400	2.36152400	-2.74352200
C	-3.04528900	-0.22625400	-2.87326000
H	-2.60438400	-0.06310700	-3.85937800
H	-2.48664600	-0.97997900	-2.31010500
H	-4.10437800	-0.48572900	-2.97378100
Cs	-4.77951100	-1.67725000	0.11711300

E(RB3LYP) = -2520.98903
Zero-point correction= 0.460283 (Hartree/Particle)
Thermal correction to Energy= 0.525682
Thermal correction to Enthalpy= 0.526958
Thermal correction to Gibbs Free Energy= 0.342838
Sum of electronic and zero-point Energies
= -2520.528747
Sum of electronic and thermal Energies
= -2520.463348
Sum of electronic and thermal Enthalpies
= -2520.462071
Sum of electronic and thermal Free Energies
= -2520.646191
E(RB3LYP/Single point) = -2521.615531
G = -2521.272693 (unit: a.u.)

$TS_{INT4 \rightarrow INT5}$

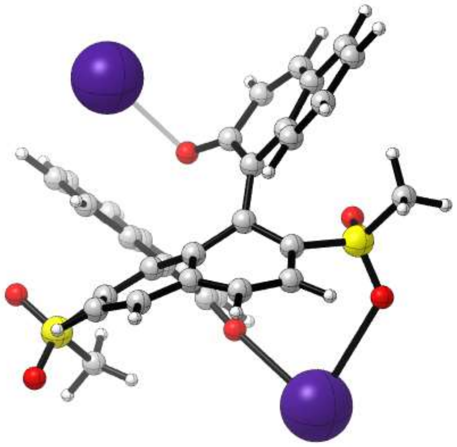

C	0.16180300	5.78742400	0.47623600
H	0.44297200	6.55660600	1.19327500
C	-0.36976300	6.13634100	-0.75385500
H	-0.53096200	7.17923400	-1.01374600
C	-0.65344200	5.11122200	-1.68406300
H	-1.01244300	5.36585100	-2.67874400
C	-0.47367500	3.78109800	-1.34877300
H	-0.68991300	3.02538600	-2.09464300
C	-0.00211200	3.37820800	-0.06335600
C	0.39167600	4.43371600	0.82202600
C	1.08633300	4.08881000	2.02782600
H	1.31993200	4.87765700	2.74147000
C	1.52326000	2.81504700	2.22455000
H	2.12624400	2.55058600	3.09095000
C	1.27264300	1.73596400	1.26737800
C	0.23305400	1.97493200	0.28671400
C	-0.59238000	0.99512400	-0.41050200
C	-1.94734200	1.39460800	-0.59312100
C	-2.64858400	1.19891300	-1.80461500
H	-3.64806100	1.60336700	-1.93249100
C	-2.00452700	0.55339500	-2.83081200
H	-2.44173800	0.47965600	-3.82241700
C	-0.83629100	-0.21221300	-2.52862700
C	-0.24489400	-0.19979500	-1.21480100
C	0.47115200	-1.38204700	-0.79195200
C	0.76607100	-2.34544300	-1.77355300
C	0.46949200	-2.15710100	-3.14302800
H	0.76709300	-2.90023800	-3.87271800
C	-0.34762700	-1.11645700	-3.50495900
H	-0.71807100	-1.03766600	-4.52390300
C	0.58976600	-1.71841500	0.65041300
C	-0.64885200	-1.91681600	1.34244000
C	-0.55363100	-2.32907900	2.73116000
H	-1.49289600	-2.45459300	3.26726700
C	0.64107300	-2.60126800	3.34154700
H	0.66749500	-2.93189800	4.37945600
C	1.87486500	-2.46610900	2.63754300
C	1.83840300	-2.01549500	1.27433400
C	3.09128100	-1.86856000	0.61317200
H	3.09696400	-1.52786300	-0.41612300
C	4.28695100	-2.13801000	1.24431700
H	5.22180900	-2.01066200	0.70129100
C	4.31346000	-2.58148400	2.59150400
H	5.26048000	-2.79227800	3.08251900
C	3.11712900	-2.73966100	3.26693300
H	3.10828400	-3.07472400	4.30310000
O	1.95806500	0.68289200	1.34586600
O	-1.80341900	-1.82024800	0.78807200
S	1.29389300	-4.06277900	-1.44205600
C	0.38138400	-4.68152700	-0.01524500
H	0.86608200	-4.38276300	0.91105100
H	-0.64750000	-4.31978600	-0.06625000
H	0.41002500	-5.76923400	-0.13515100
O	0.79805400	-4.85597900	-2.60098600
O	2.74760500	-4.16112100	-1.16900800
Cs	4.19968900	1.62790400	-0.78568600
S	-2.91521100	2.11286900	0.73571100
O	-4.25686500	1.46657400	0.60585600
O	-2.22934700	1.98527500	2.03983800
C	-3.22033400	3.86357500	0.43082100
H	-2.31881000	4.43878400	0.63230300
H	-3.54981700	3.99205800	-0.60212600
H	-4.01981600	4.14479500	1.12299800
Cs	-4.66854500	-1.68001800	0.24661700

Imaginary frequency: –26.85 cm^{-1}
E(RB3LYP) = -2520.97390296
Zero-point correction= 0.459556 (Hartree/Particle)
Thermal correction to Energy= 0.523976
Thermal correction to Enthalpy= 0.525253
Thermal correction to Gibbs Free Energy= 0.342441
Sum of electronic and zero-point Energies
= -2520.514347
Sum of electronic and thermal Energies
= -2520.449927
Sum of electronic and thermal Enthalpies
= -2520.448650
Sum of electronic and thermal Free Energies
= -2520.631462
E(RB3LYP/Single point) = -2521.59967783
G = -2521.257237 (unit: a.u.)

(*meso*)-**INT5**

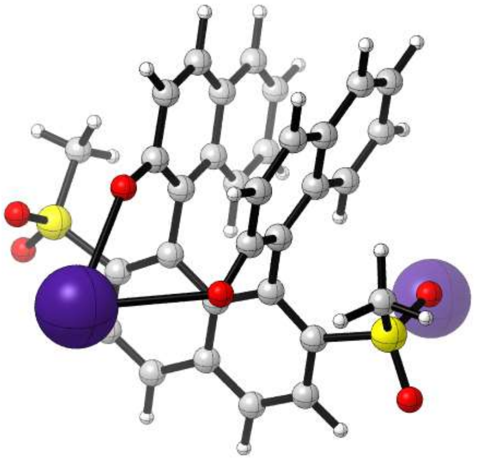

C	0.58767700	4.40162500	1.46192400
H	0.61144400	5.03919300	2.34441300
C	1.42953600	4.65803300	0.39309100
H	2.12135800	5.49602900	0.42118000
C	1.37195800	3.81547700	-0.74483500
H	2.02271200	4.01291500	-1.59444600
C	0.50375100	2.74232000	-0.78775600
H	0.48706400	2.11199300	-1.67176900
C	-0.36680000	2.43605700	0.30606200
C	-0.31761000	3.31417800	1.44556600
C	-1.20010400	3.05462200	2.53466600
H	-1.15828300	3.71032800	3.40371100
C	-2.08074800	2.00578100	2.49955500
H	-2.74909600	1.81532900	3.33737200
C	-2.18276900	1.09824400	1.36774900
C	-1.28013800	1.34840700	0.28788100
C	-1.34639900	0.53198700	-0.95927700
C	-2.24494000	0.89427000	-1.96197000
C	-2.24325900	0.31745400	-3.25585000
H	-2.96451100	0.64749500	-3.99380500
C	-1.28706600	-0.60969500	-3.57327600
H	-1.23579900	-1.02999300	-4.57403900
C	-0.34259700	-1.03292200	-2.60381500
C	-0.39189300	-0.52029800	-1.25946700
C	0.51380900	-1.08607500	-0.27597400
C	1.45849700	-2.00640200	-0.72502700
C	1.55557500	-2.43826800	-2.07065500
H	2.31084100	-3.16220900	-2.35256900
C	0.64949800	-1.97250300	-2.98527800
H	0.67205500	-2.32285800	-4.01360900
C	0.33056900	-0.88151500	1.19006000
C	-0.62214900	-1.75219600	1.80582600
C	-0.79527800	-1.58934800	3.23589300
H	-1.54474500	-2.21901100	3.71234200
C	-0.05678100	-0.70415400	3.97603000
H	-0.21610200	-0.61792500	5.05049300
C	0.94133500	0.11639800	3.36920400
C	1.13045700	0.01473200	1.94756100
C	2.13439600	0.84068700	1.36411500
H	2.28295900	0.78947200	0.29195400
C	2.90467500	1.69806600	2.12356700
H	3.66050500	2.31318800	1.63994900
C	2.70412700	1.80076000	3.52300800
H	3.30422400	2.48848700	4.11369000
C	1.73232900	1.01879800	4.12365100
H	1.55958200	1.08418600	5.19693100
O	-3.03426600	0.14891700	1.37079500
O	-1.25929100	-2.66844000	1.16776500
S	2.73353500	-2.76406000	0.33690200
C	2.03980500	-3.32533600	1.89686900
H	1.96295800	-2.49521800	2.59495000
H	1.06654200	-3.78144100	1.70518400
H	2.75519500	-4.07342600	2.25329900
O	3.17998000	-3.98978400	-0.37648600
O	3.78165500	-1.74637300	0.61579800
Cs	4.89265400	0.31064400	-1.61174700
S	-3.59385700	2.11429800	-1.76444800
O	-3.92642600	2.56039200	-3.14536200
O	-4.69135100	1.50076500	-0.98192100
C	-3.02671800	3.59447600	-0.91325500
H	-2.93674300	3.41320600	0.15474400
H	-2.08002200	3.91805100	-1.34832200
H	-3.81092200	4.32977800	-1.12119700
Cs	-4.12856200	-2.27822800	0.11484700

E(RB3LYP) = -2521.04457460
Zero-point correction= 0.461832 (Hartree/Particle)
Thermal correction to Energy= 0.526845
Thermal correction to Enthalpy= 0.528122
Thermal correction to Gibbs Free Energy= 0.344823
Sum of electronic and zero-point Energies
= -2520.582743
Sum of electronic and thermal Energie
s= -2520.517729
Sum of electronic and thermal Enthalpies
= -2520.516452
Sum of electronic and thermal Free Energies
= -2520.699752
E(RB3LYP/Single point) = -2521.66949398
G = -2521.324671 (unit: a.u.)

$MeSO_2Cs$

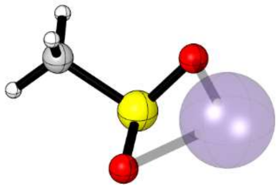

S	-2.20543500	0.00001100	-0.52558400
C	-3.08486600	-0.00003900	1.09252500
H	-3.70904600	0.89743400	1.16546500
H	-3.70902100	-0.89753300	1.16542200
H	-2.32403700	-0.00004800	1.88252800
O	-1.32637200	-1.27077100	-0.39959100
O	-1.32640100	1.27080700	-0.39952800
Cs	1.54109900	-0.00000100	0.07334100

E(RB3LYP) = -608.789286851
Zero-point correction= 0.044659 (Hartree/Particle)
Thermal correction to Energy= 0.055555
Thermal correction to Enthalpy= 0.056832
Thermal correction to Gibbs Free Energy
= -0.005859
Sum of electronic and zero-point Energies
= -608.744628
Sum of electronic and thermal Energies
= -608.733732
Sum of electronic and thermal Enthalpies
= -608.732455
Sum of electronic and thermal Free Energies
= -608.795146
E(RB3LYP/Single point) = -608.9094733
G = -608.9153323 (unit: a.u.)

NMR Spectra

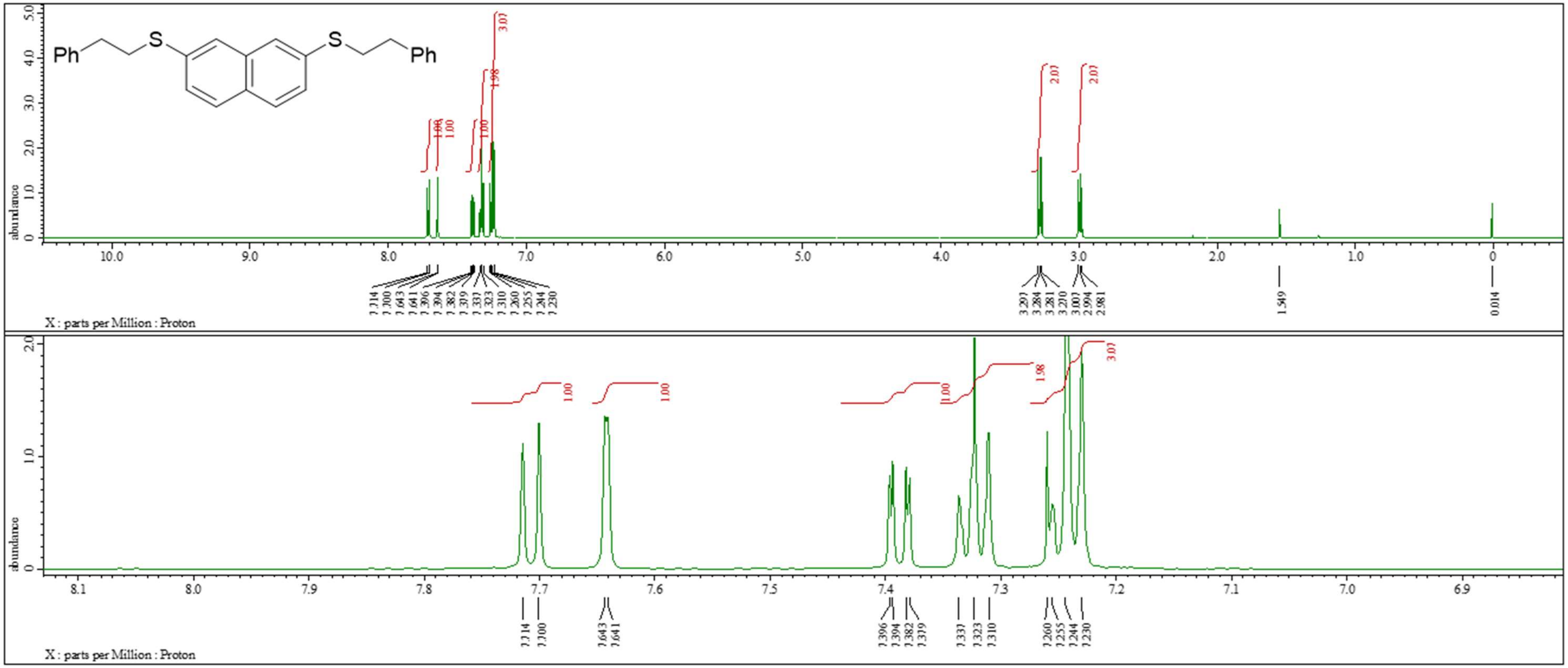

Fig. S38 ^{1}H NMR spectrum of **2** in $CDCl_3$.

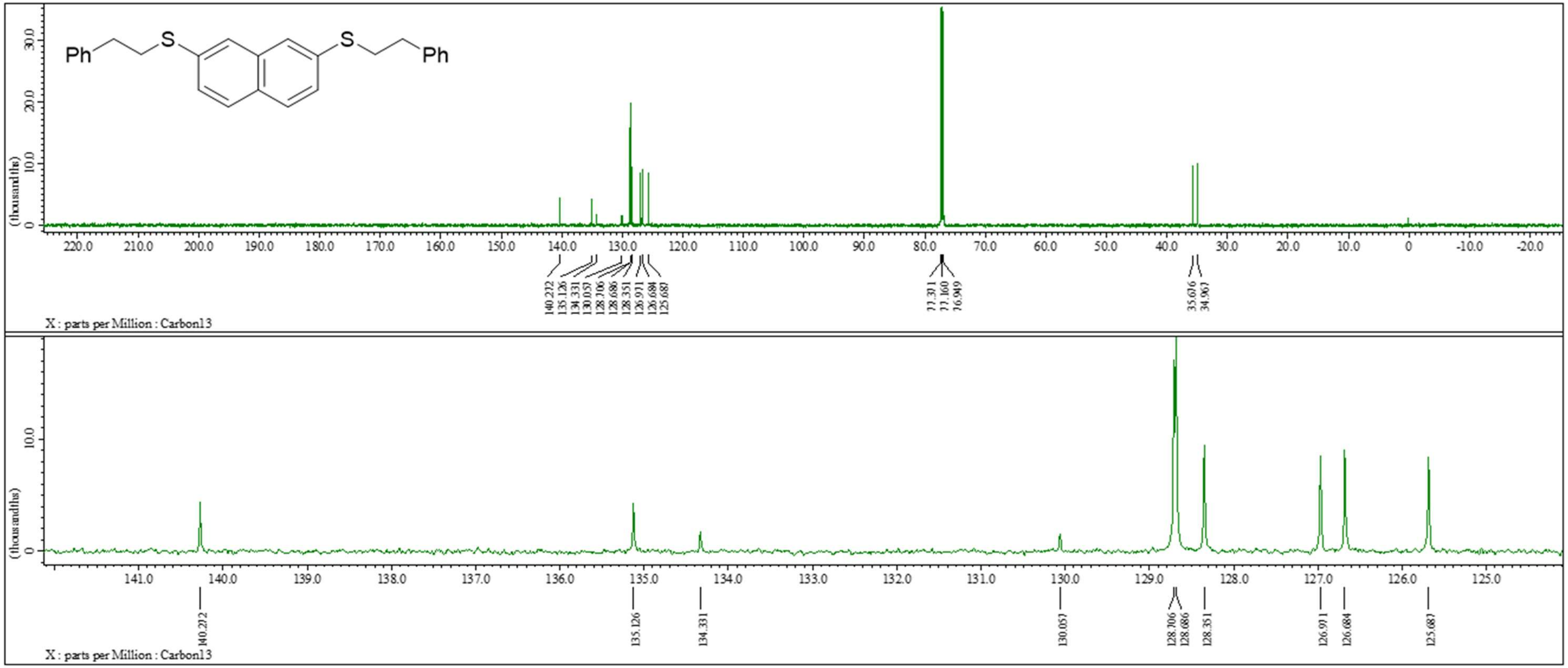

Fig. S39 ^{13}C NMR spectrum of **2** in $CDCl_3$.

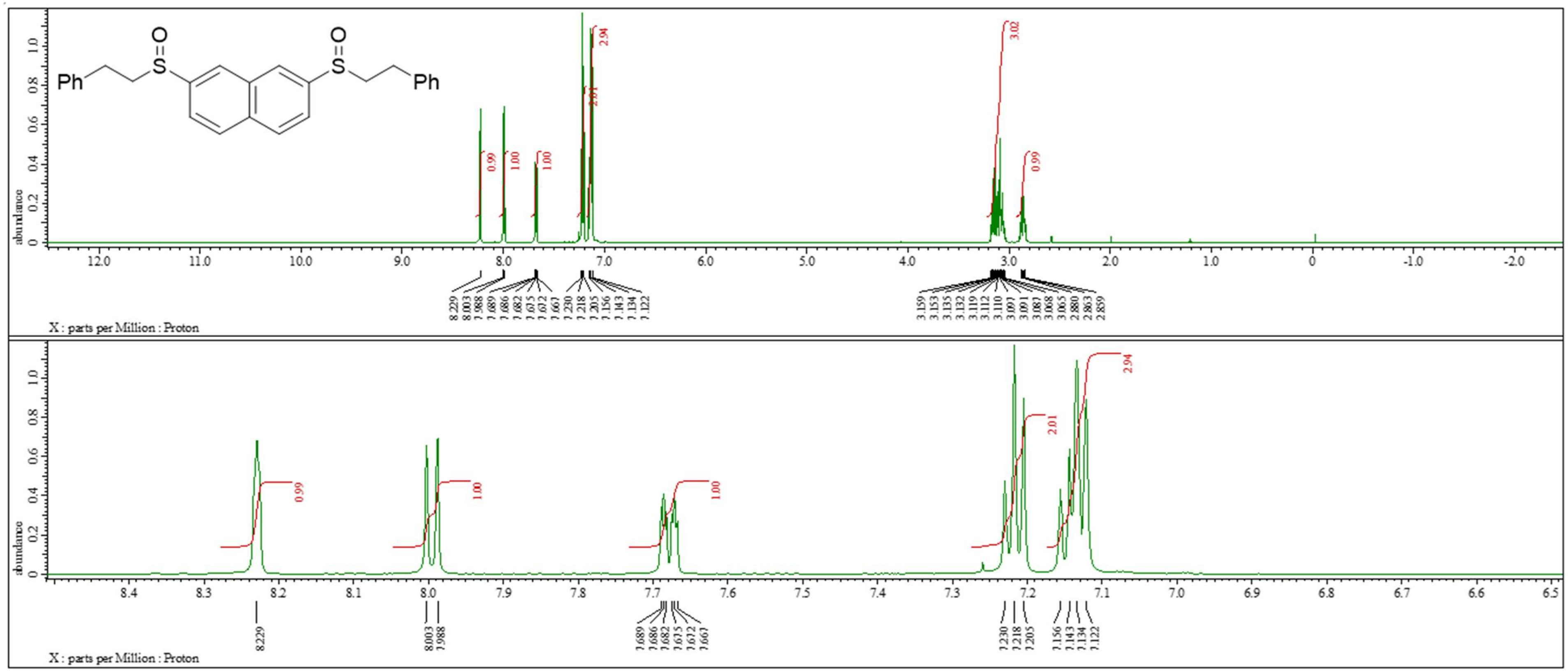

Fig. S40 ^{1}H NMR spectrum of **3** in $CDCl_3$.

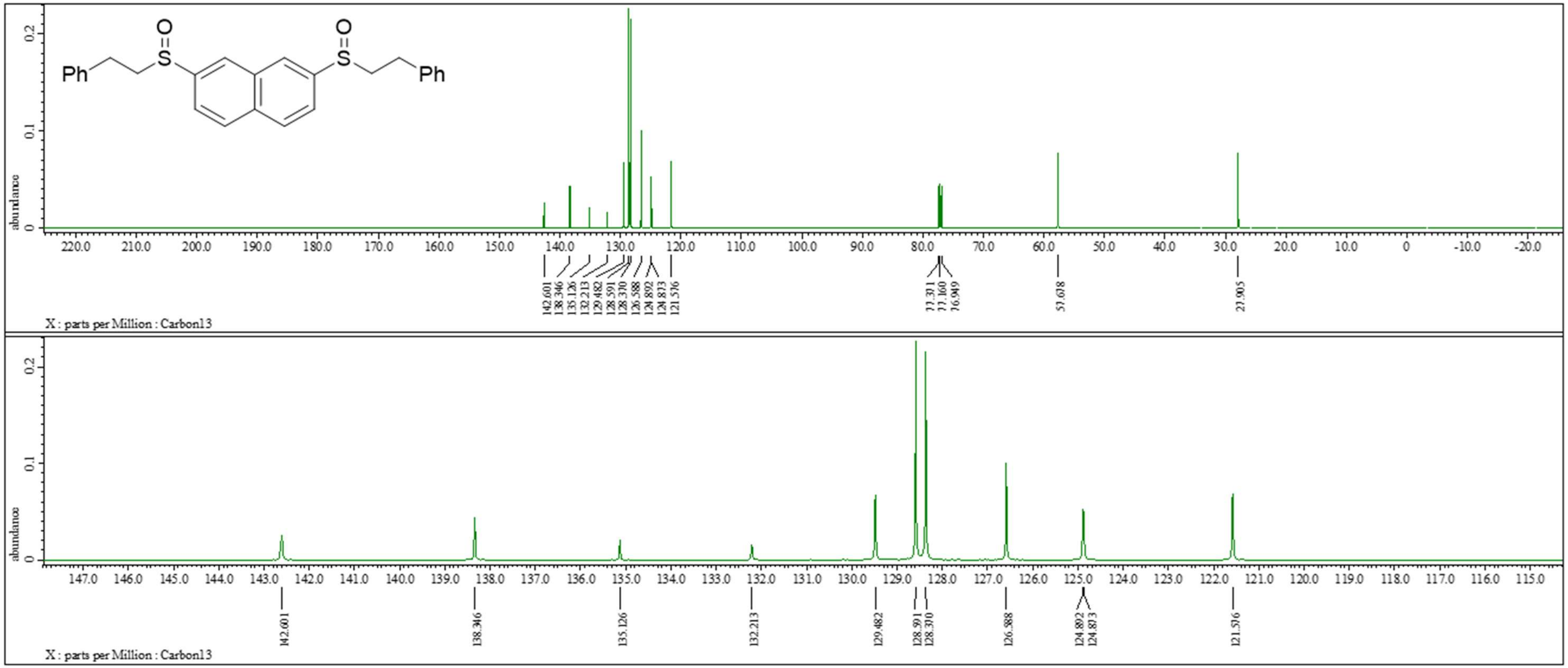

Fig. S41 ^{13}C NMR spectrum of **3** in $CDCl_3$.

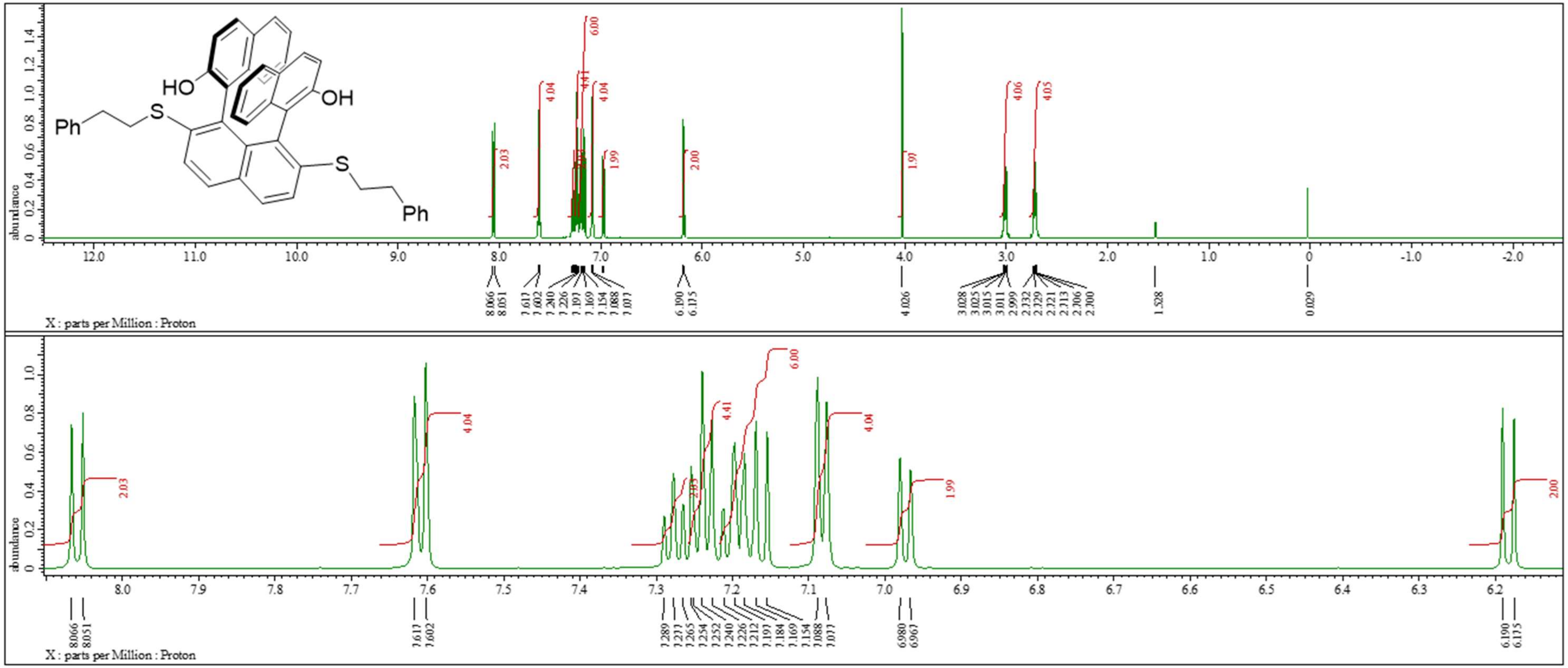

Fig. S42 ^{1}H NMR spectrum of (*rac*)-**4** in $CDCl_3$.

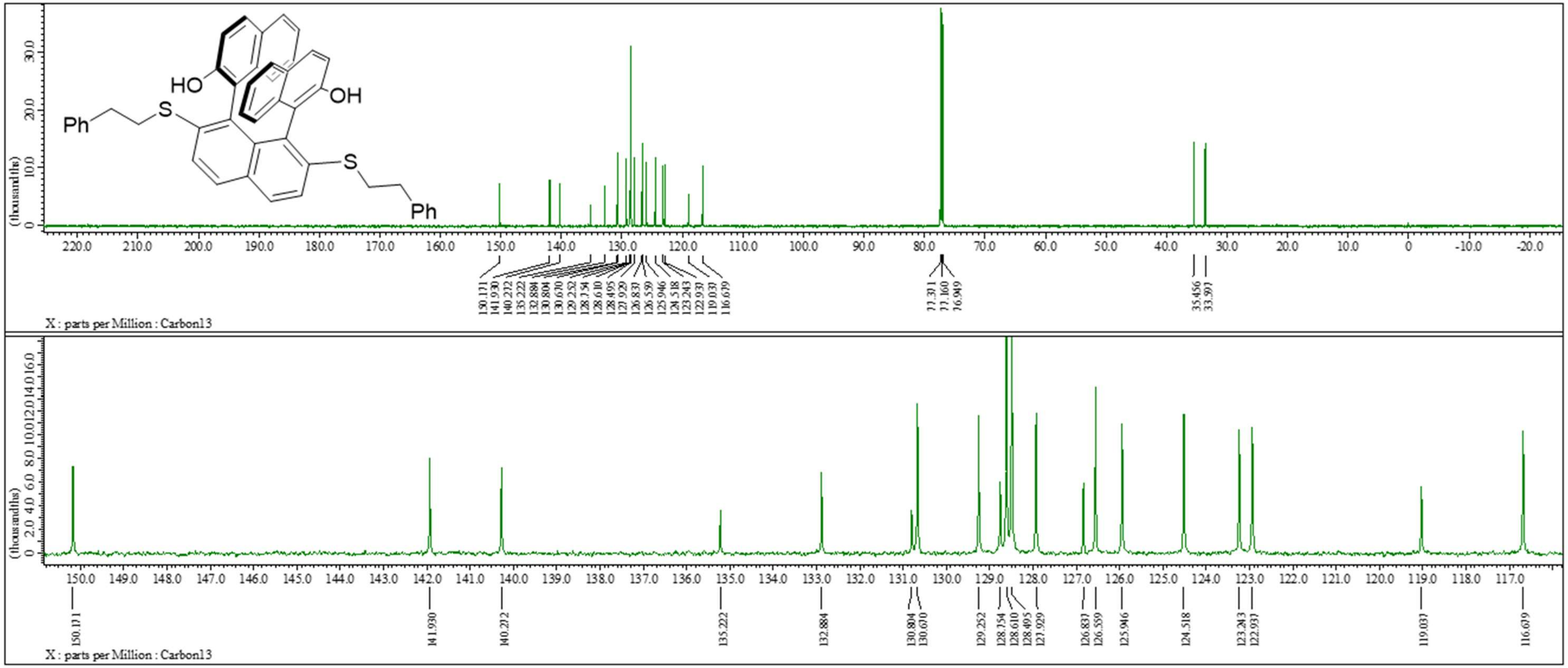

Fig. S43 ^{13}C NMR spectrum of (*rac*)-**4** in $CDCl_3$.

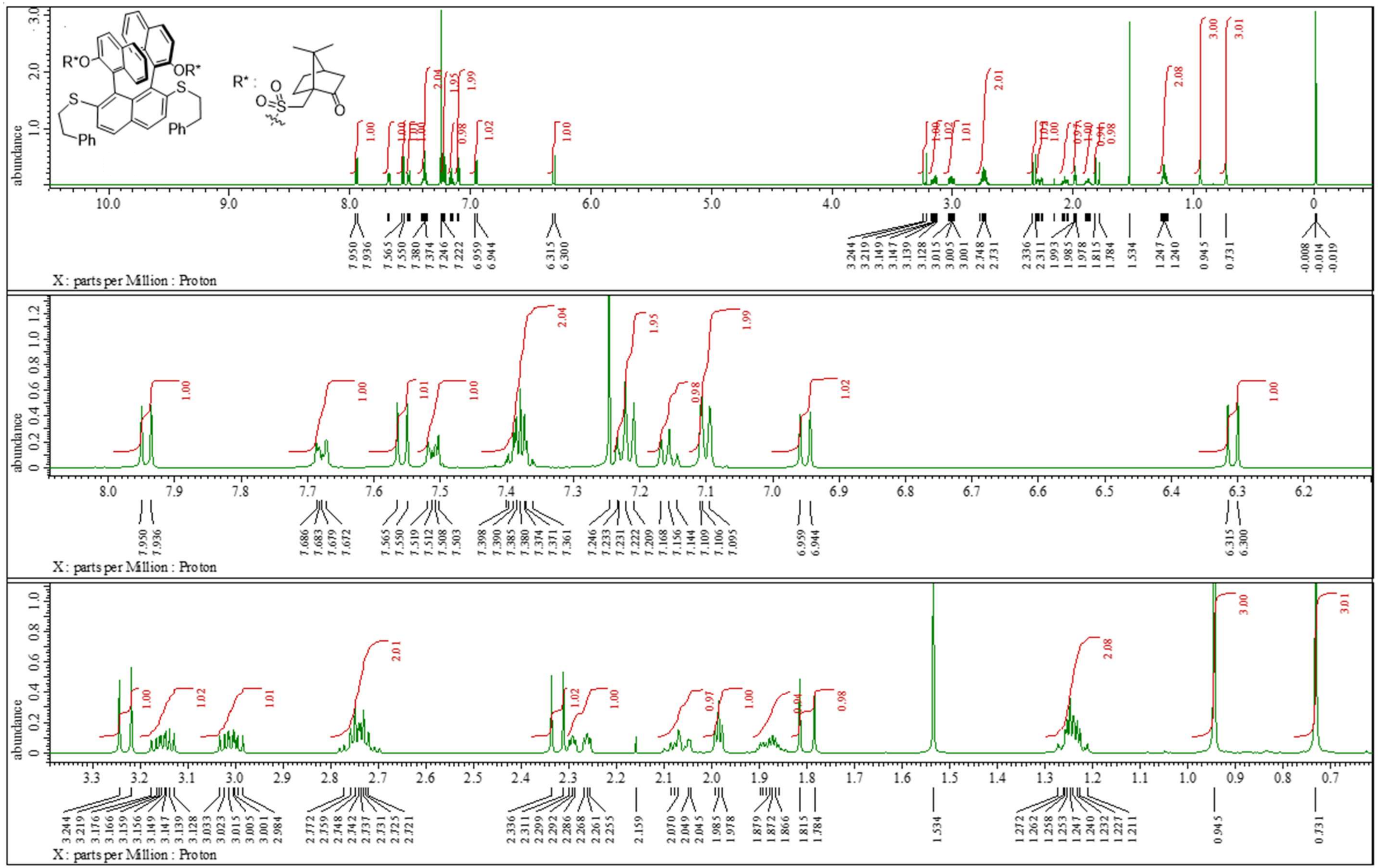

Fig. S44 ^{1}H NMR spectrum of (R_a,R_a)**-4'** in $CDCl_3$.

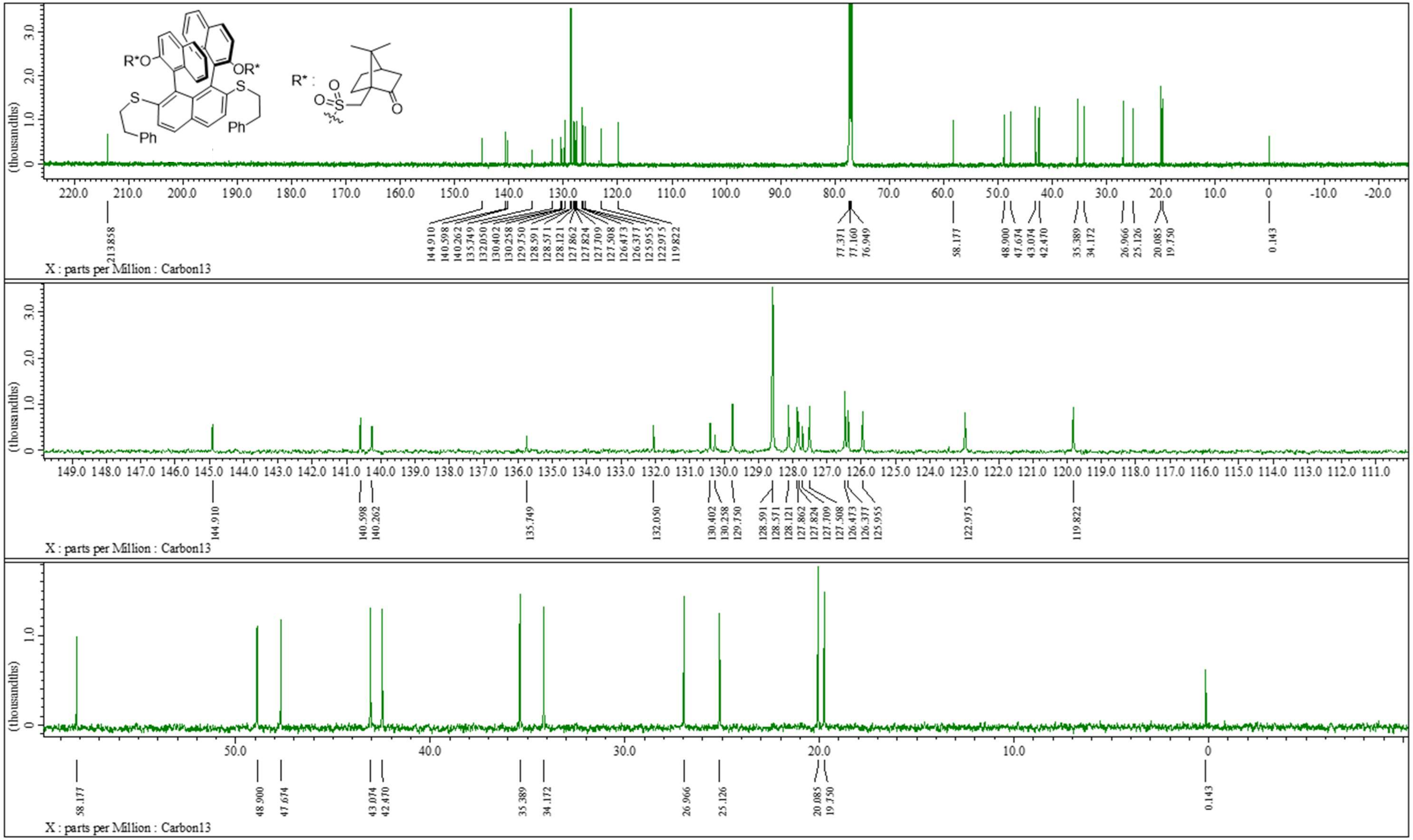

Fig. S45 ^{13}C NMR spectrum of (R_a,R_a)**-4'** in $CDCl_3$.

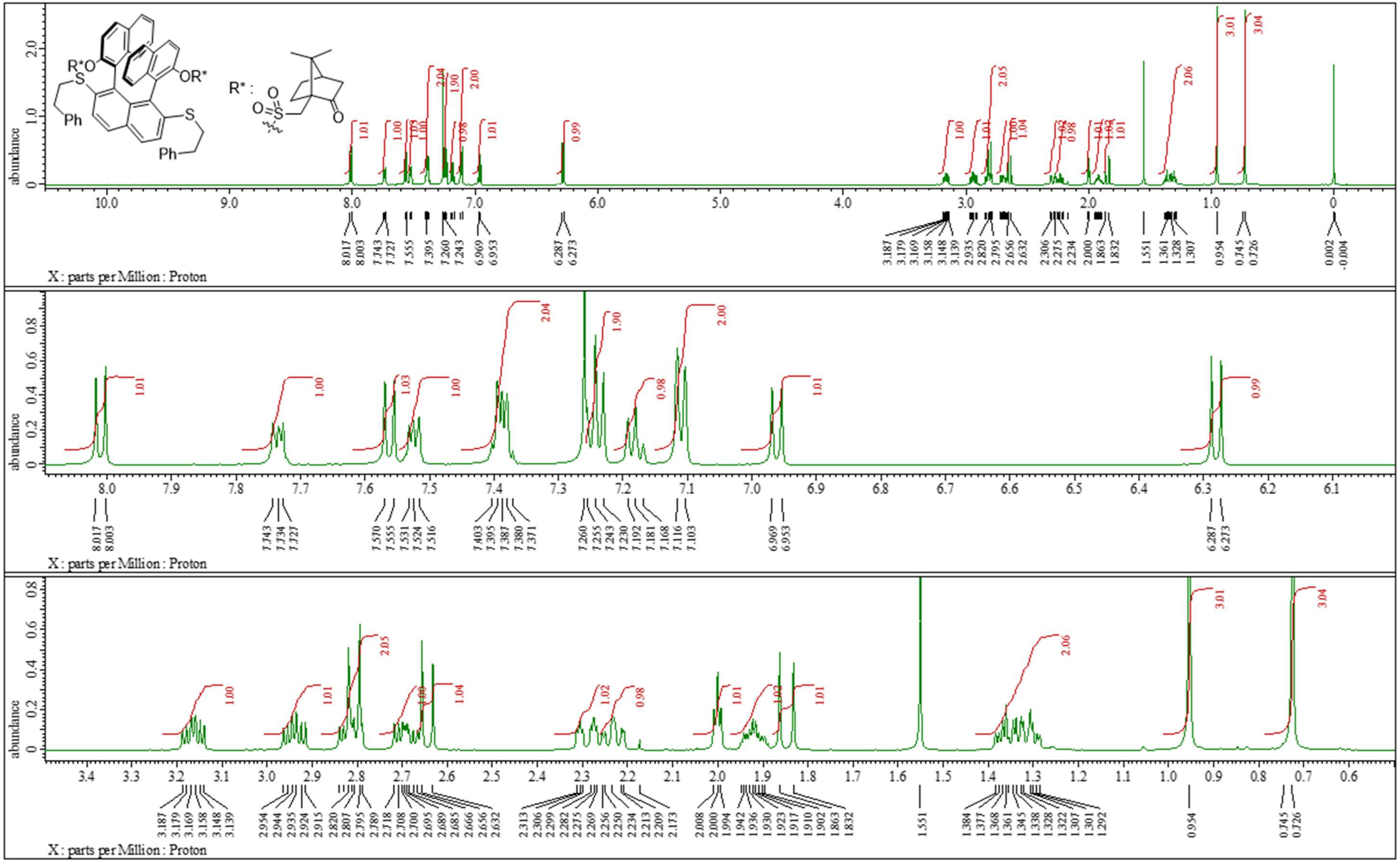

Fig. S46 ^{1}H NMR spectrum of (S_a,S_a)**-4'** in $CDCl_3$.

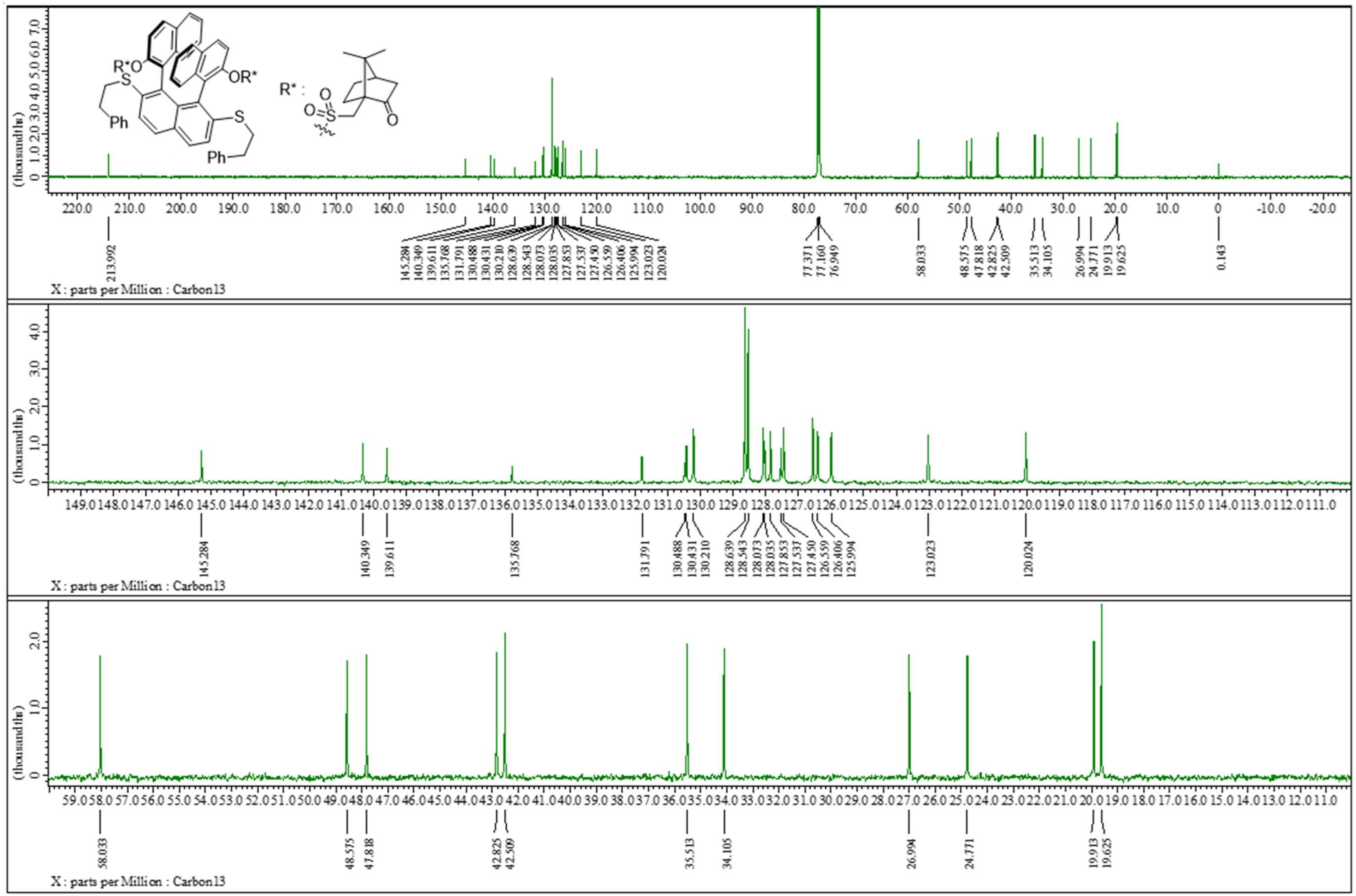

Fig. S47 ^{13}C NMR spectrum of (S_a,S_a)**-4'** in $CDCl_3$.

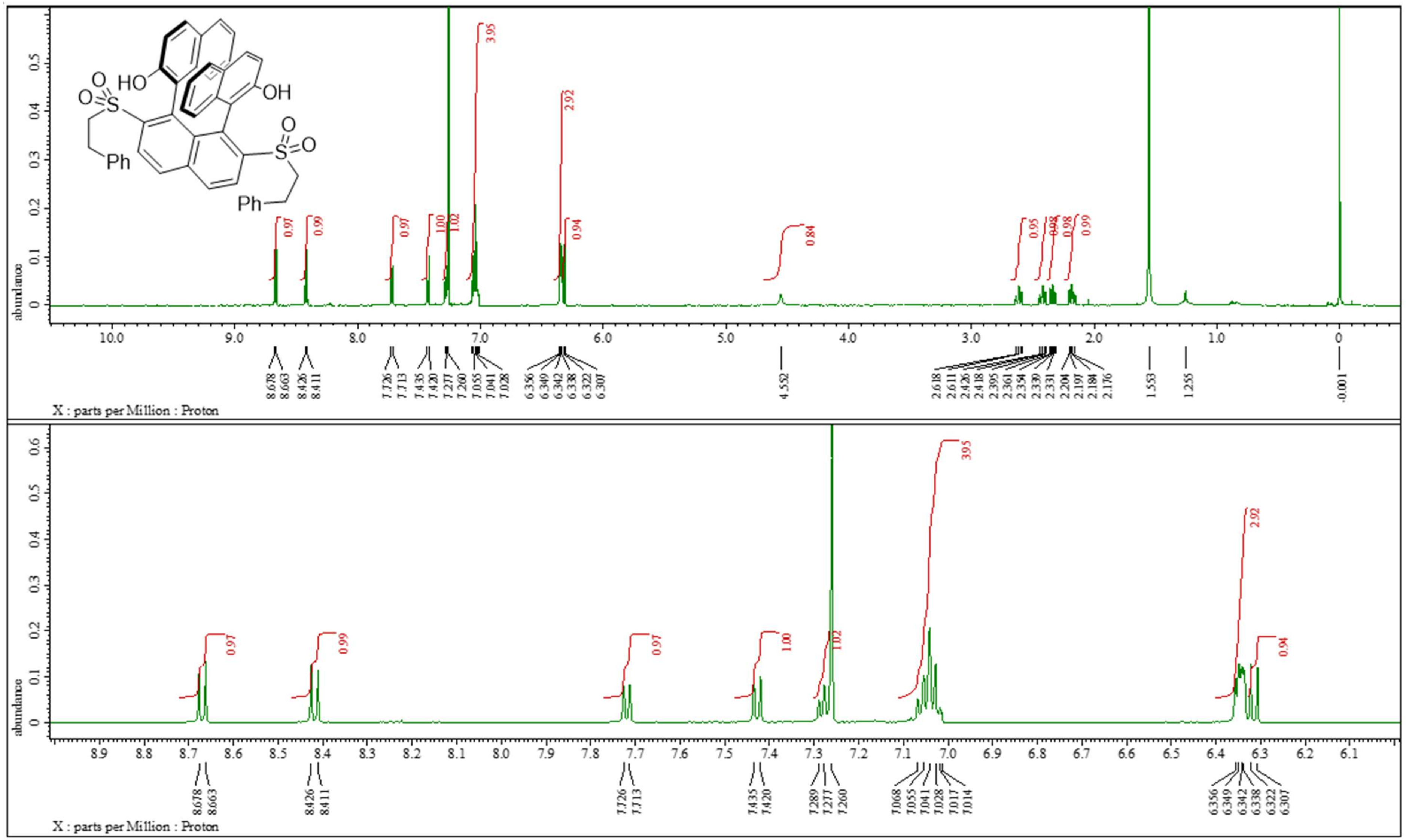

Fig. S48 ^{1}H NMR spectrum of (*rac*)-**5** in $CDCl_3$.

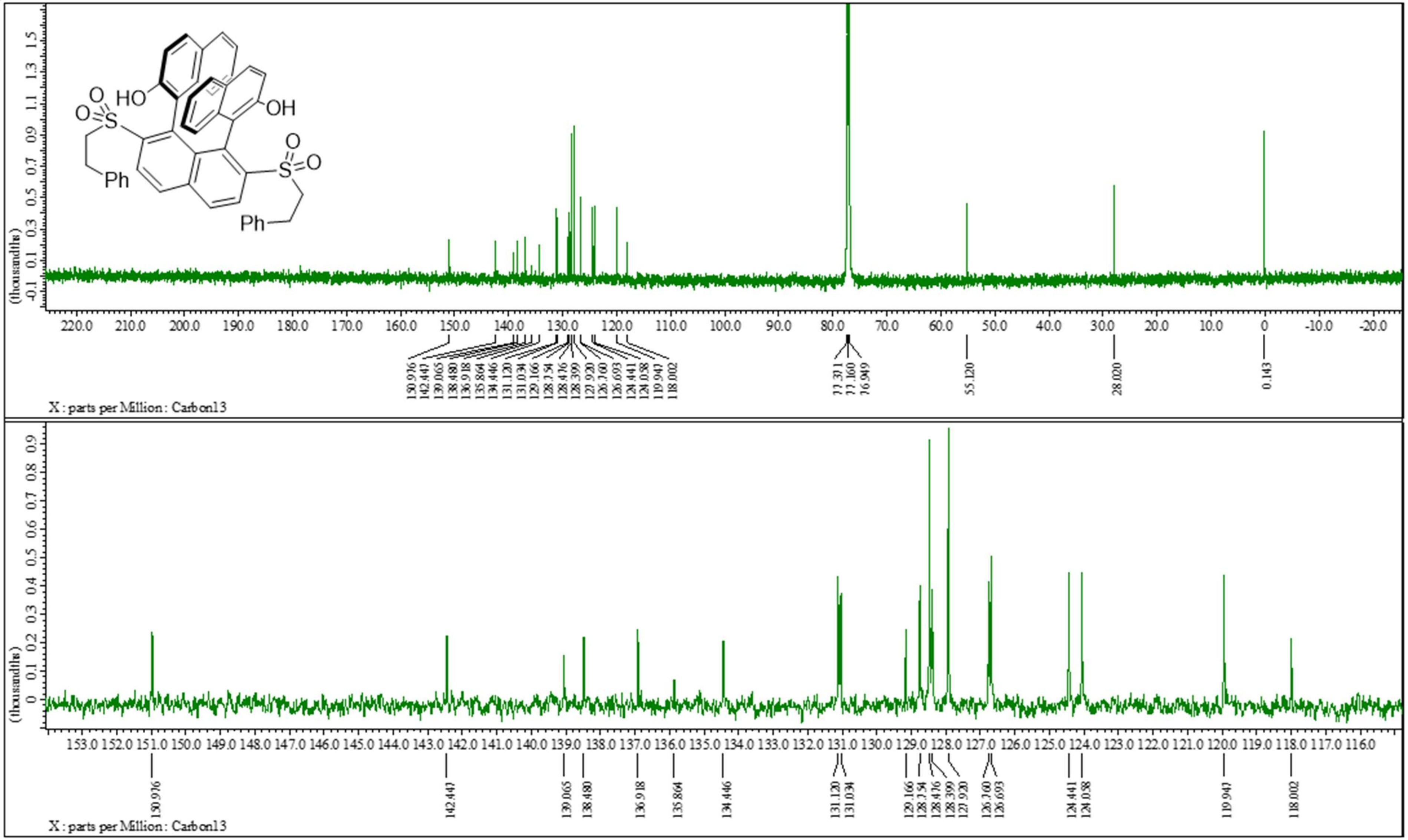

Fig. S49 ^{13}C NMR spectrum of (*rac*)-**5** in $CDCl_3$.

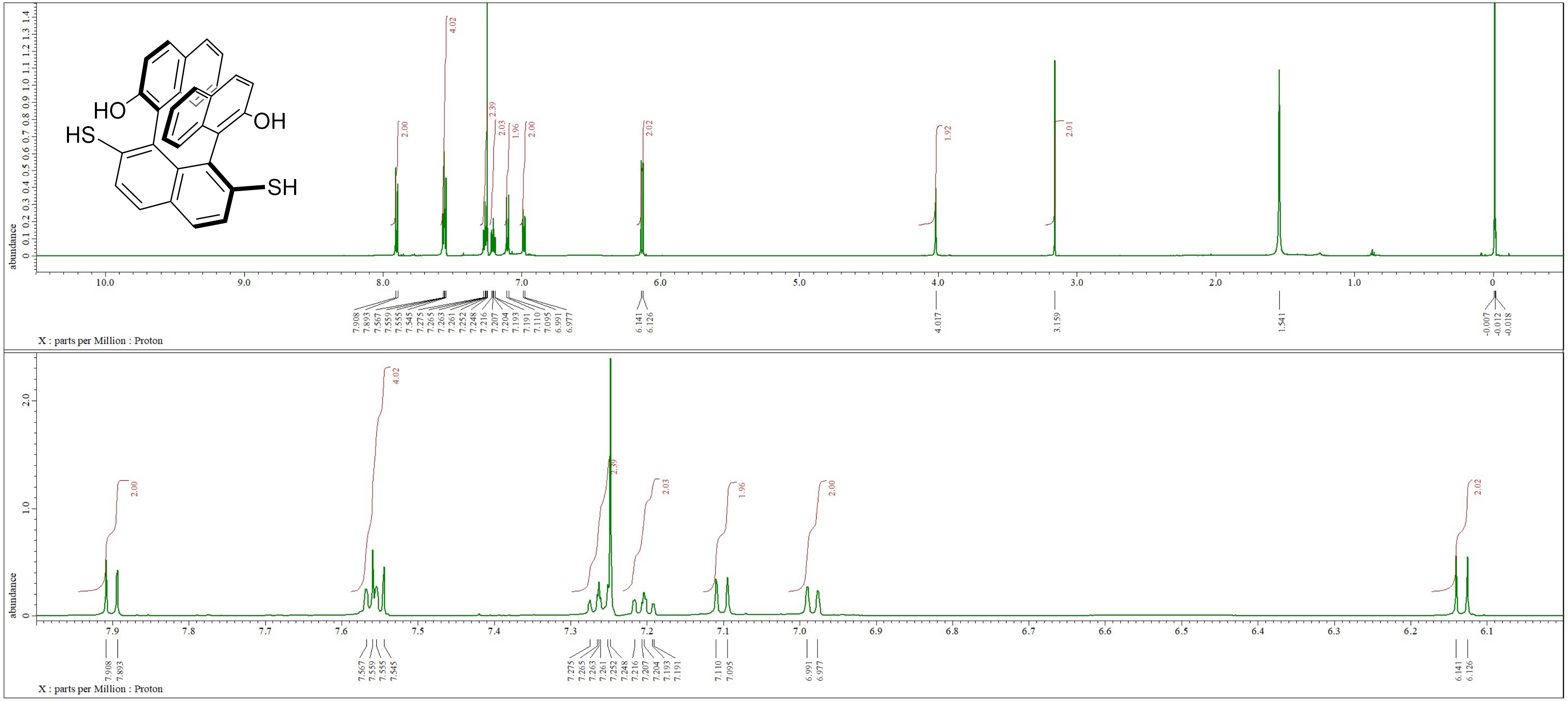

Fig. S50 ^{1}H NMR spectrum of (*rac*)-**7** in $CDCl_3$.

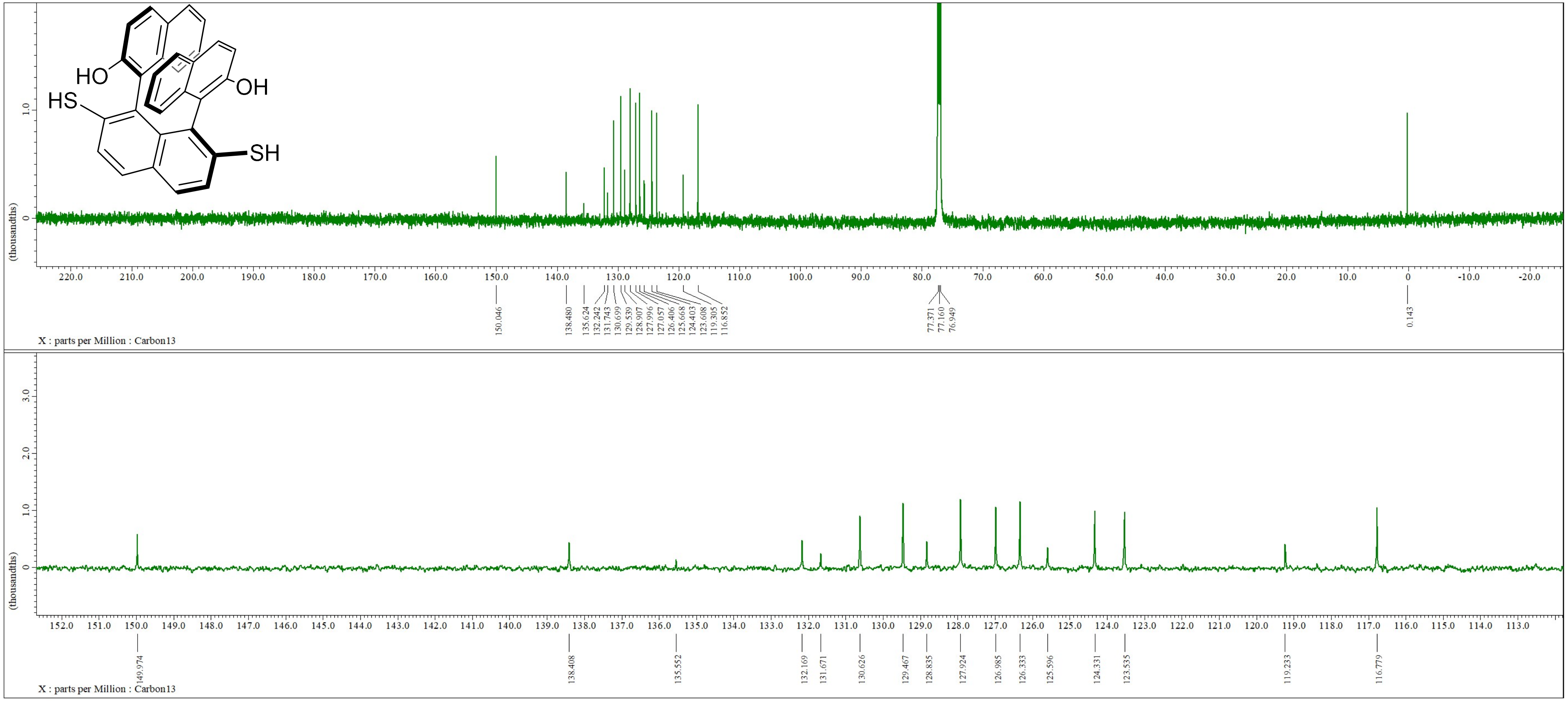

Fig. S51 ^{13}C NMR spectrum of (*rac*)-**7** in $CDCl_3$.

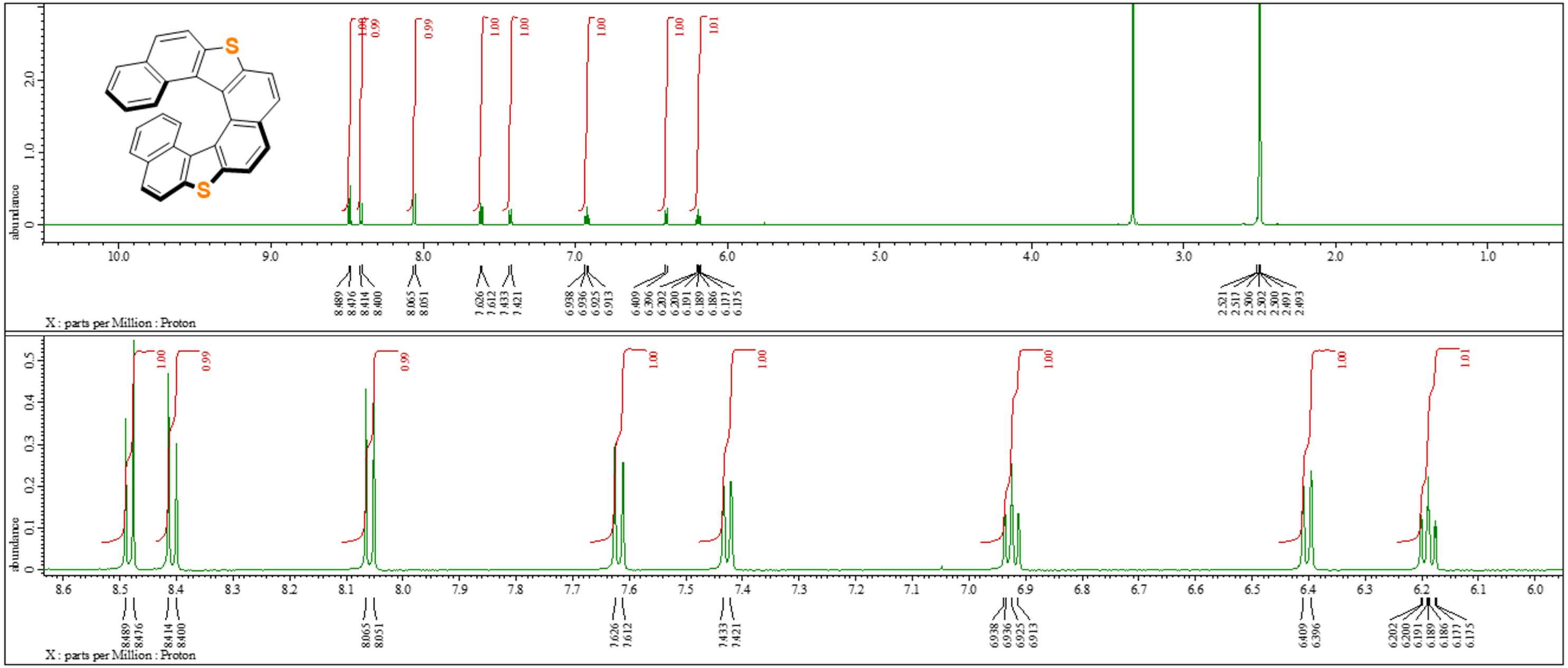

Fig. S52 ^{1}H NMR spectrum of (*rac*)-**6S** in DMSO-d_6.

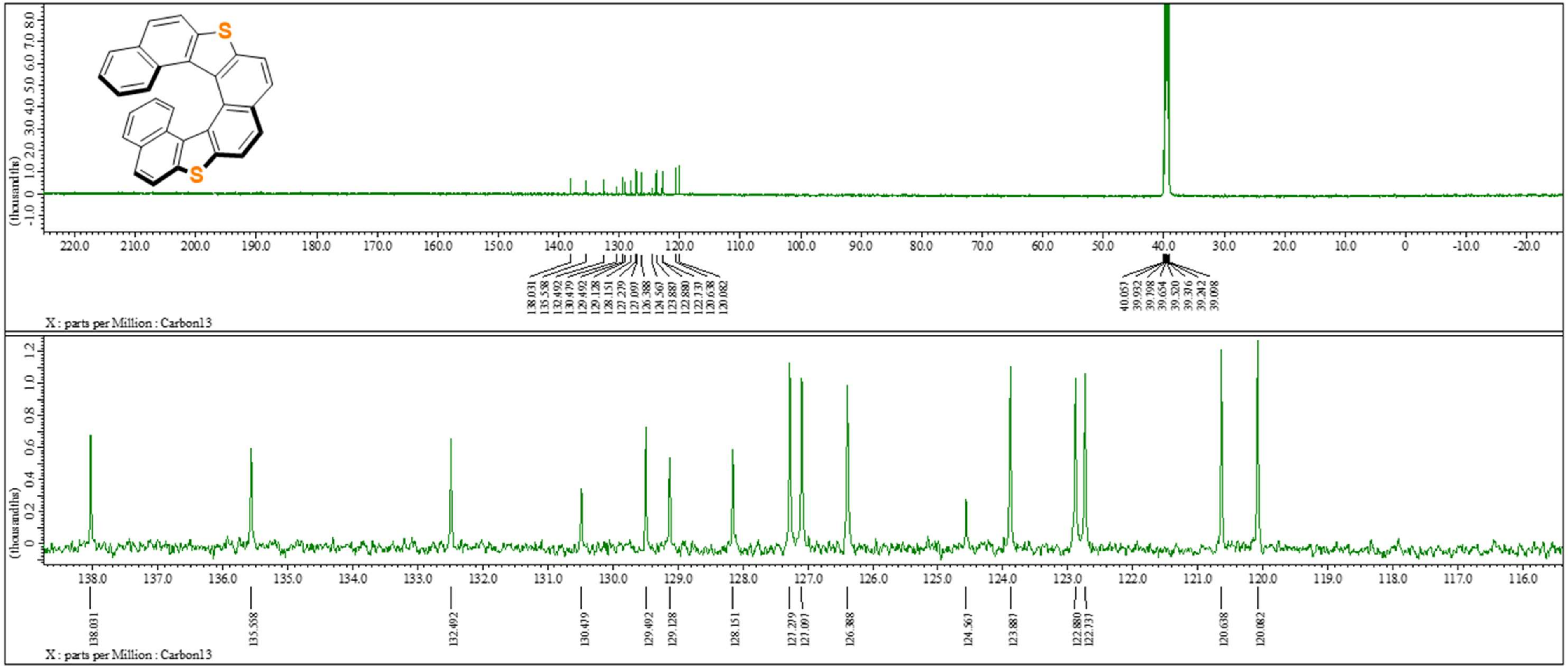

Fig. S53 ^{13}C NMR spectrum of (*rac*)-**6S** in DMSO-d_6.

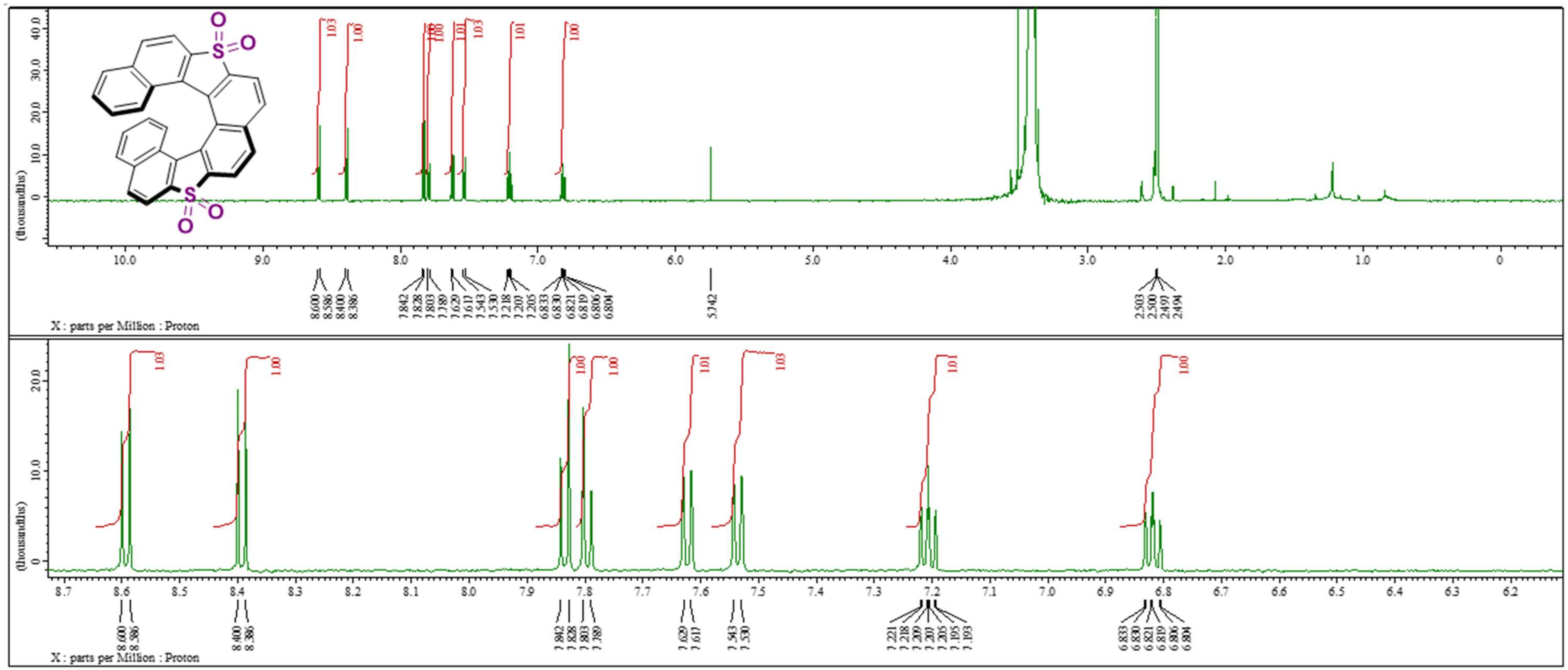

Fig. S54 ^{1}H NMR spectrum of (*rac*)-**6SO₂** in DMSO-d_6.

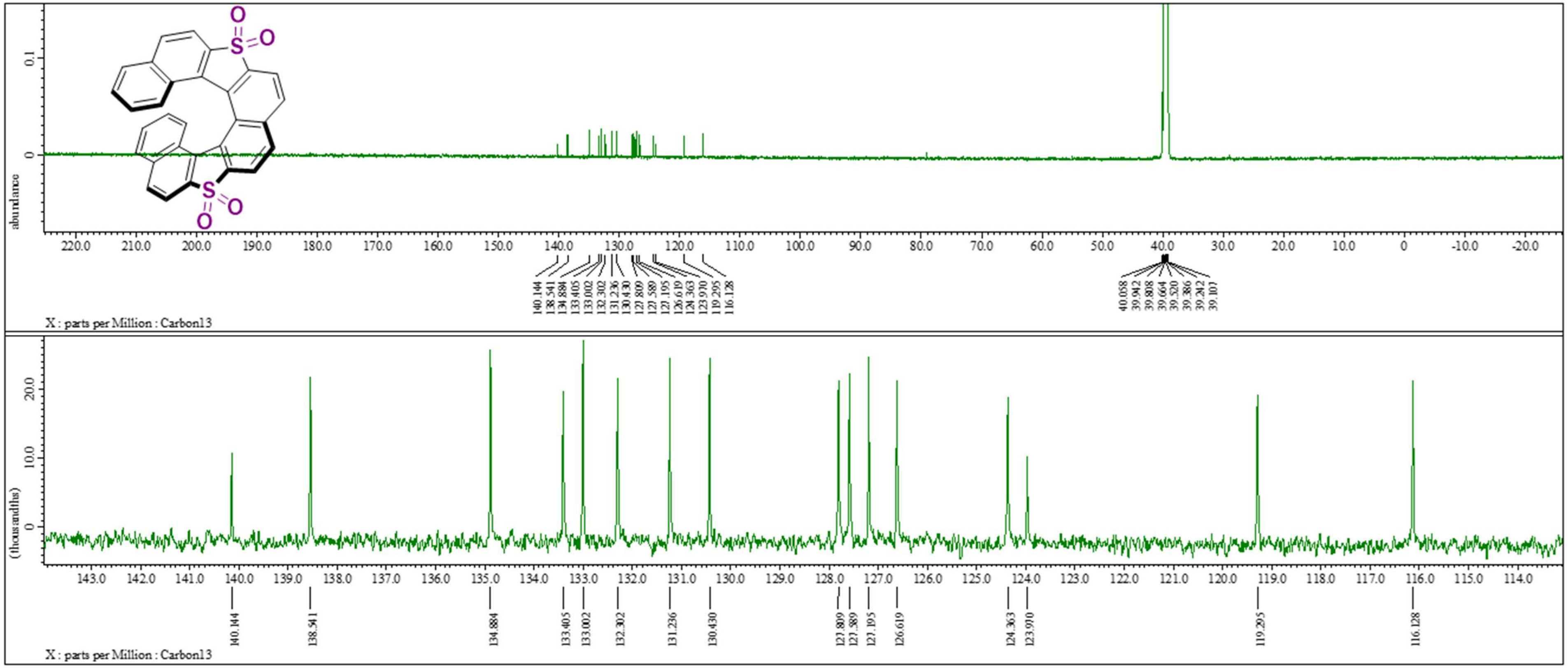

Fig. S55 ^{13}C NMR spectrum of (*rac*)-**6SO$_2$** in DMSO-d_6.

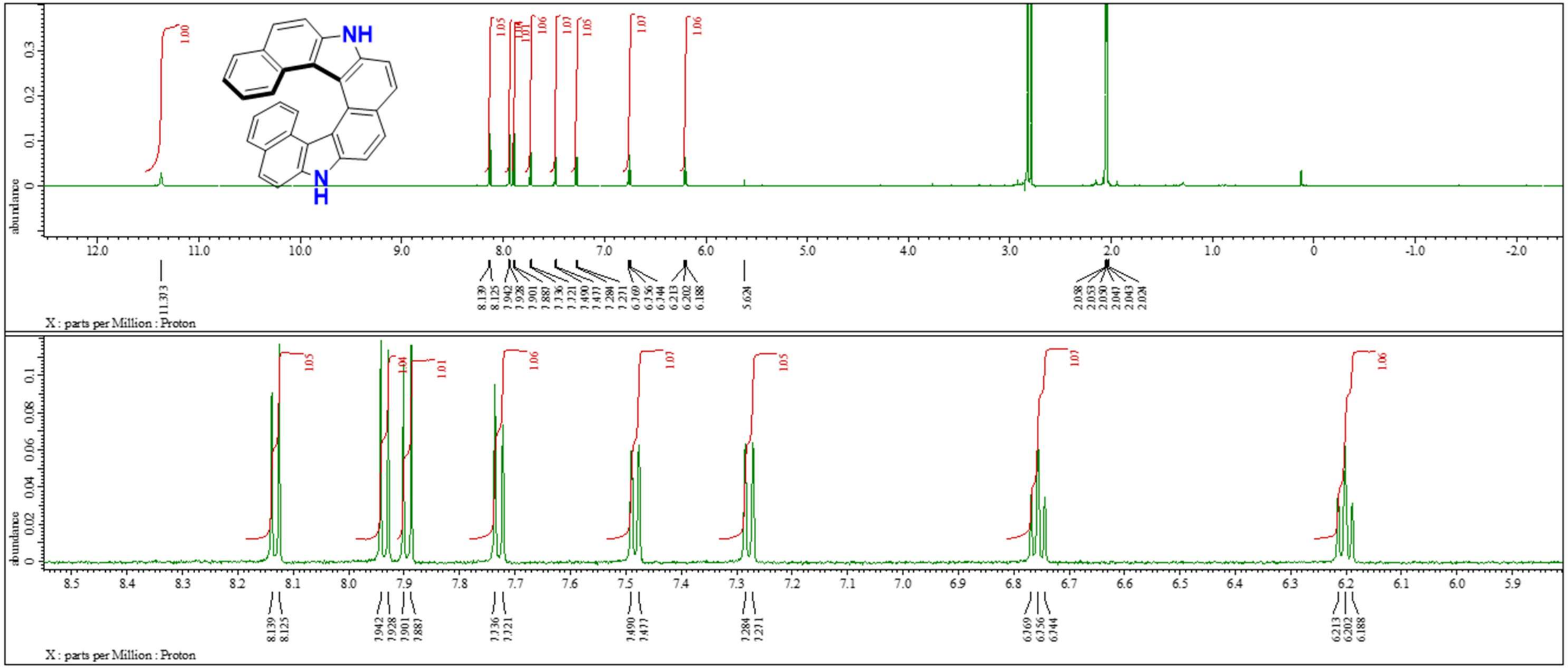

Fig. S56 ^{1}H NMR spectrum of (*rac*)-**6N** in aceton-d_6.

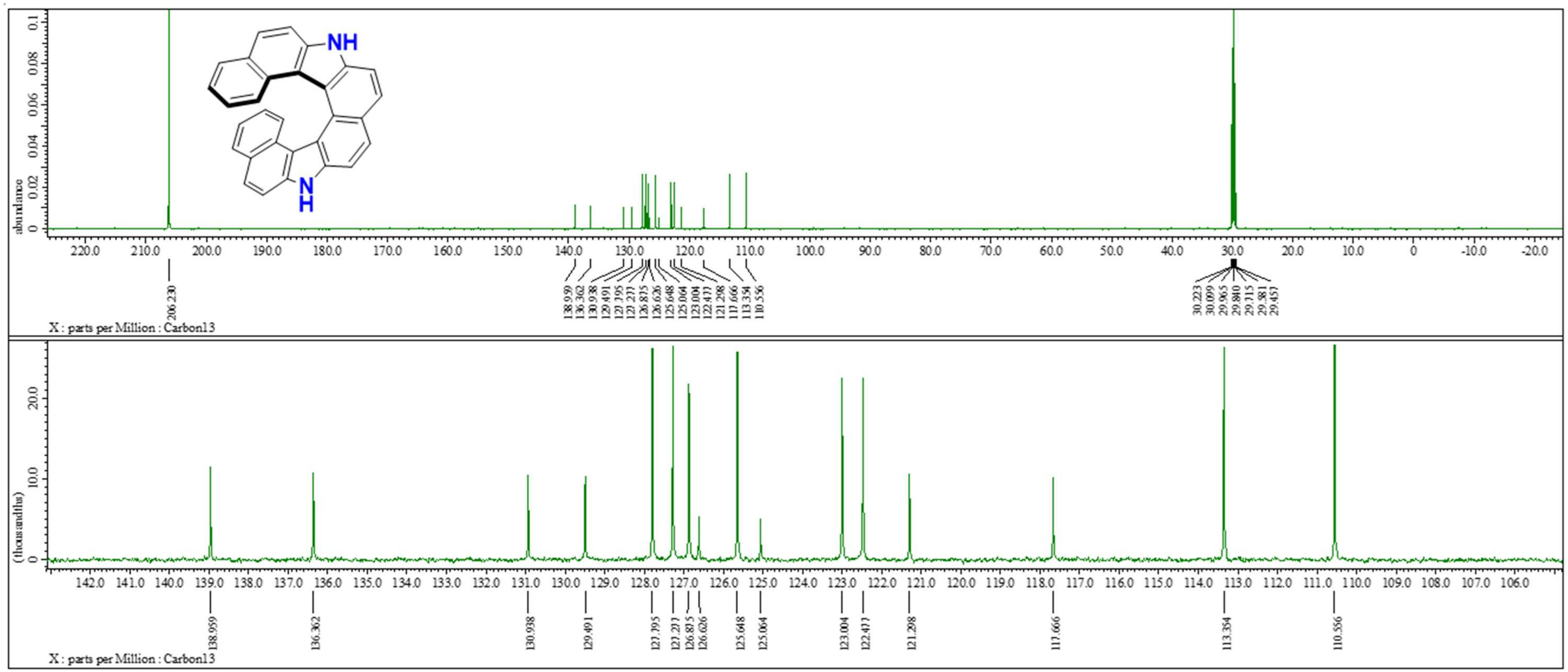

Fig. S57 ^{13}C NMR spectrum of (*rac*)-**6N** in aceton-d_6.

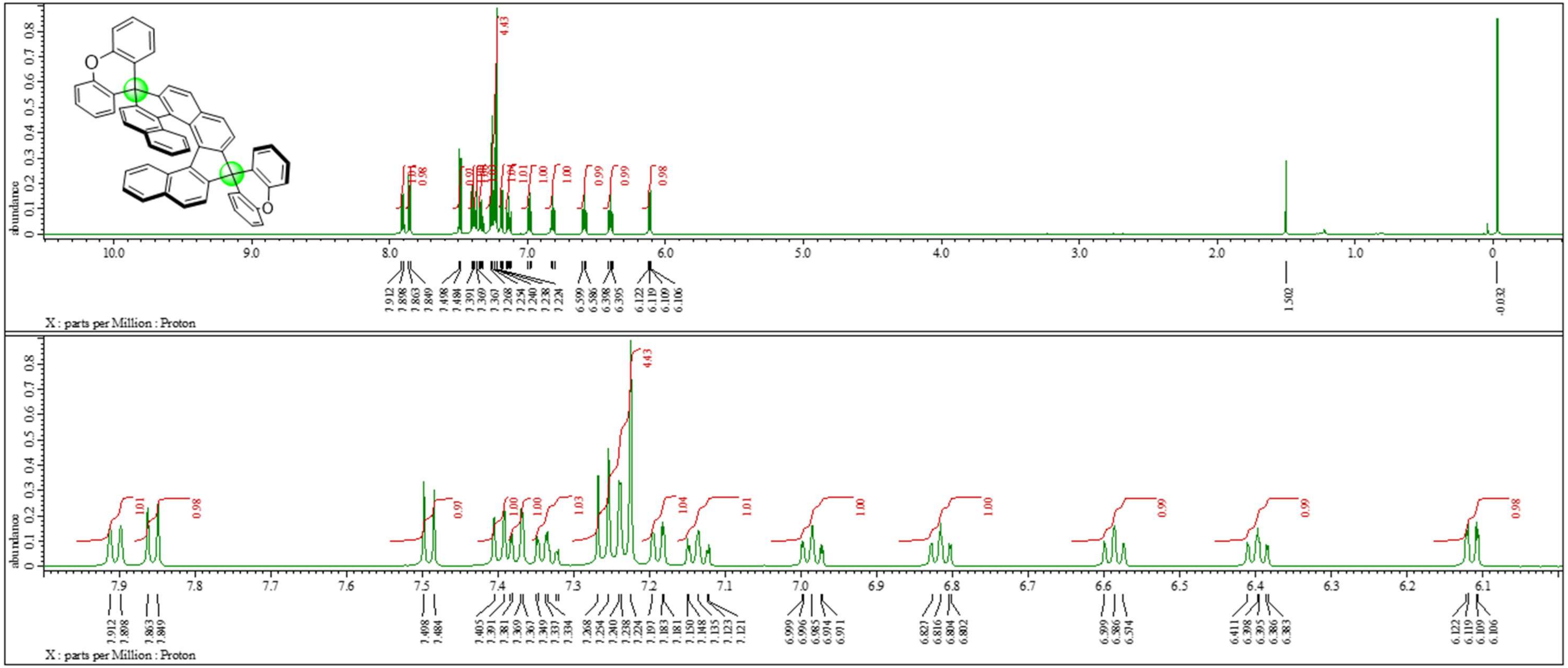

Fig. S58 ^{1}H NMR spectrum of (*rac*)-**6CX** in $CDCl_3$.

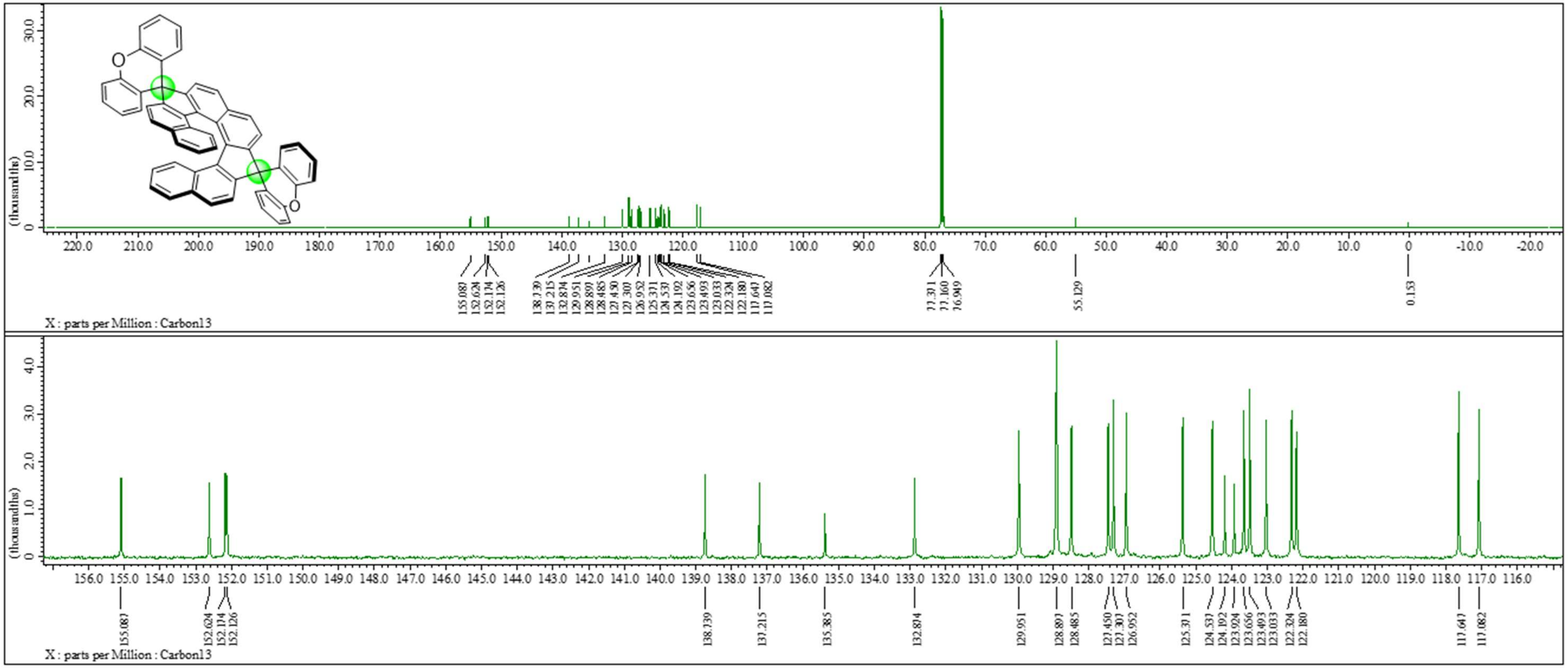

Fig. S59 ^{13}C NMR spectrum of (*rac*)-**6CX** in $CDCl_3$.

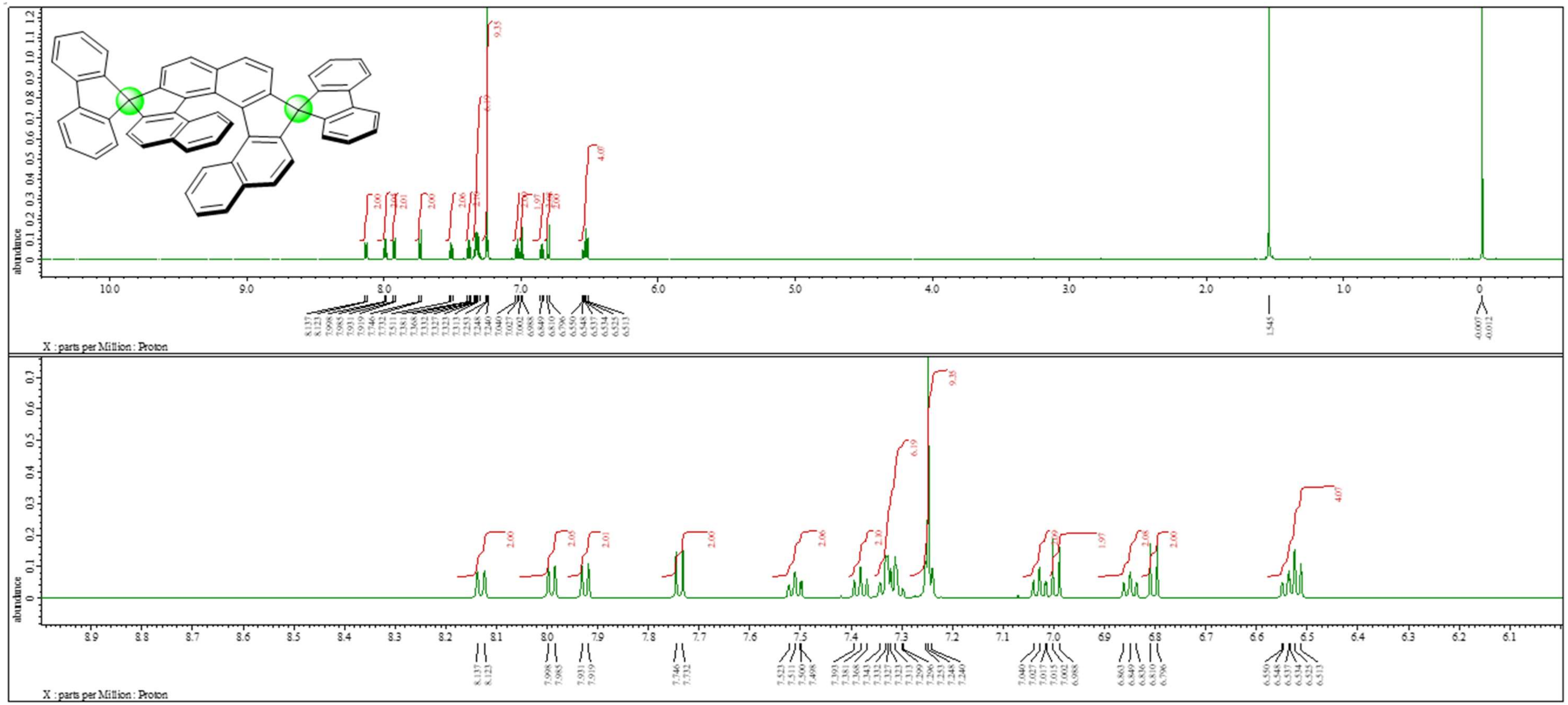

Fig. S60 ^{1}H NMR spectrum of (*rac*)-**6CF** in $CDCl_3$.

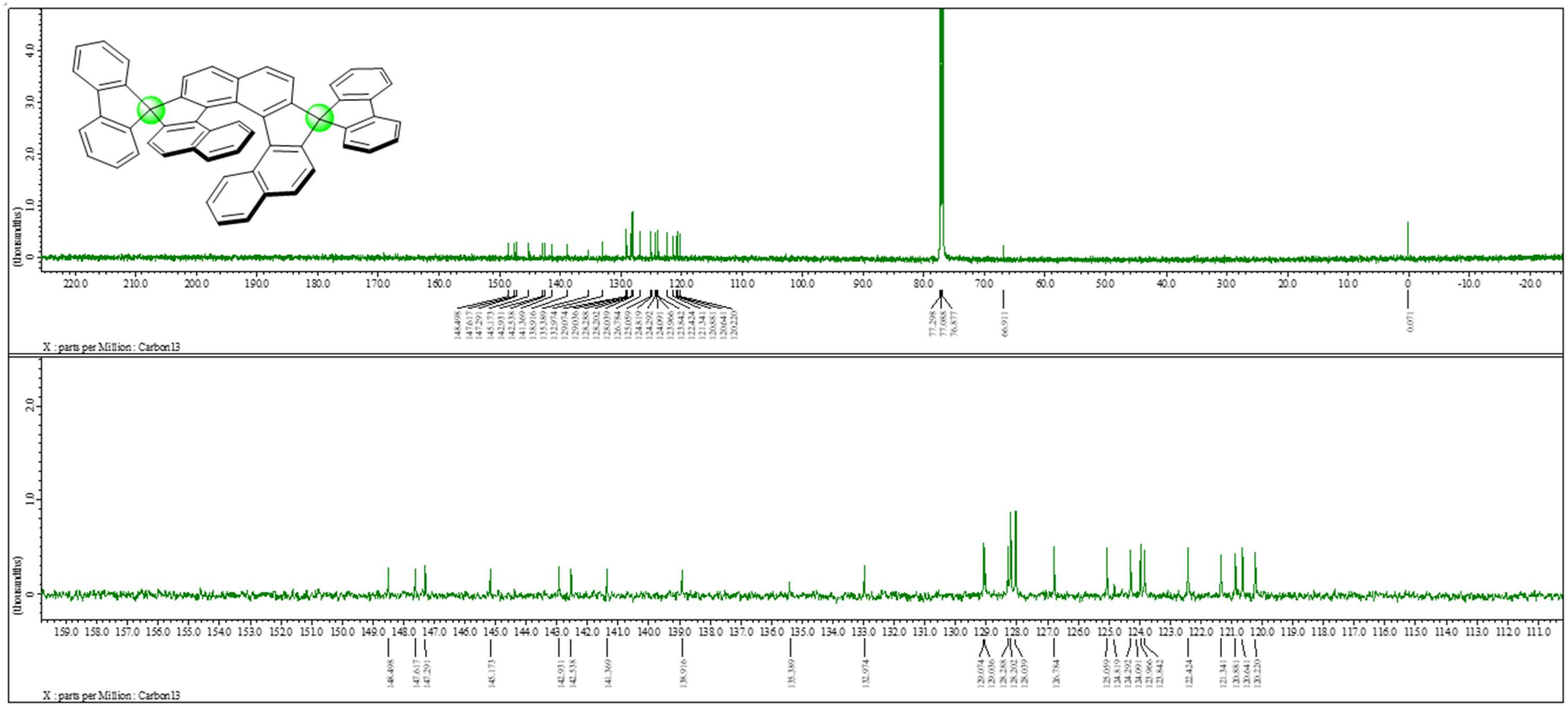

Fig. S61 ^{13}C NMR spectrum of (*rac*)-**6CF** in $CDCl_3$.
